# Identification of Novel Viruses and Their Microbial Hosts from Soils with Long-Term Nitrogen Fertilization and Cover Cropping Management

**DOI:** 10.1128/msystems.00571-22

**Published:** 2022-11-29

**Authors:** Ning Duan, Mark Radosevich, Jie Zhuang, Jennifer M. DeBruyn, Margaret Staton, Sean M. Schaeffer

**Affiliations:** a Department of Biosystems Engineering and Soil Science, The University of Tennessee, Knoxville, Tennessee, USA; b Center for Environmental Biotechnology, The University of Tennessee, Knoxville, Tennessee, USA; c Department of Entomology and Plant Pathology, University of Tennessee, Knoxville, Tennessee, USA; Los Alamos National Laboratory

**Keywords:** soil, viruses, phage, bacteria, archaea, metagenomics, N-fertilization, nitrogen fertilization, cover cropping, nitrogen, conservation

## Abstract

Soils are the largest organic carbon reservoir and are key to global biogeochemical cycling, and microbes are the major drivers of carbon and nitrogen transformations in the soil systems. Thus, virus infection-induced microbial mortality could impact soil microbial structure and functions. In this study, we recovered 260 viral operational taxonomic units (vOTUs) in samples collected from soil taken from four nitrogen fertilization (N-fertilization) and cover-cropping practices at an experimental site under continuous cotton production evaluating conservation agricultural management systems for more than 40 years. Only ~6% of the vOTUs identified were clustered with known viruses in the RefSeq database using a gene-sharing network. We found that 14% of 260 vOTUs could be linked to microbial hosts that cover key carbon and nitrogen cycling taxa, including *Acidobacteriota*, *Proteobacteria*, *Verrucomicrobiota*, *Firmicutes*, and ammonia-oxidizing archaea, i.e., *Nitrososphaeria* (phylum *Thermoproteota*). Viral diversity, community structure, and the positive correlation between abundance of a virus and its host indicate that viruses and microbes are more sensitive to N-fertilization than cover-cropping treatment. Viruses may influence key carbon and nitrogen cycling through control of microbial function and host populations (e.g., *Chthoniobacterales* and *Nitrososphaerales*). These findings provide an initial view of soil viral ecology and how it is influenced by long-term conservation agricultural management.

**IMPORTANCE** Bacterial viruses are extremely small and abundant particles that can control the microbial abundance and community composition through infection, which gradually showed their vital roles in the ecological process to influence the nutrient flow. Compared to the substrate control, less is known about the influence of soil viruses on microbial community function, and even less is known about microbial and viral diversity in the soil system. To obtain a more complete knowledge of microbial function dynamics, the interaction between microbes and viruses cannot be ignored. To fully understand this process, it is fundamental to get insight into the correlation between the diversity of viral communities and bacteria which could induce these changes.

## INTRODUCTION

Viruses are a major driving force that regulate the microbial biomass and influence aquatic food webs (1, 2). A bacteriophage is a virus which only infects and replicates within bacteria (3). It has been proposed that much of the soluble organic carbon resulting from bacterial lysis due to bacteriophage infection can be recycled to feed other microbes instead of being transferred to higher trophic levels. This repeating cycling is called the “microbial (bacterium–phage–DOC) loop” or “viral shunt” (4–6). Up to 40% of bacteria were lysed daily due to viral infection in oceans, which can release ~10^9^ tons of carbon per day (7–9). Comparable estimates do not exist for soil ecosystems, but a conceptual model has been proposed for the impact of virus-mediated cell impact and its impact on the distribution of soil labile carbon and recalcitrant carbon pools (10).

Soils is a complex, heterogeneous ecosystem that provides habitat for a vast array of diverse microbes and higher life forms. The microbial community is critical biological component of soil health and driver of nutrient cycling (11–16). Microbial community structure and function can be modulated by the changes of environmental factors which drove by different types of cover crops. For example, vetch, having a lower C/N ratio have higher decomposition rates by microbes compared to wheat with high C/N ratio (17, 18). Inorganic nitrogen fertilization can decrease the soil pH, leading to soil acidity, and in turn impact microbial enzyme activities, nutrient availability, and the solubility of metals and other toxic substances (19–21). These factors and others can combine to shape microbial diversity and function (22). Like microbial hosts, viruses also likely respond to the physicochemical changes brought about by long-term agricultural management practices. For example, cover cropping and nitrogen fertilization (N-fertilization) can increase soil organic matter and N input to soil. Organic matter can compete with viral adsorption by blocking adsorption sites on soil particles, which could prompt release of adsorbed viral particles that could subsequently increase host infection rates and produce more viruses (23). The balance between these mechanisms is likely dependent on chemical properties of soil such as pH and ionic strength of the soil solution (24, 25). N-fertilization is a supplemental N source in agricultural systems that is fundamental element for viral reproduction to synthesize amino acids, nucleotides, etc. (26). Therefore, studying the response of viral populations and virus-host linkage under agricultural management practices is important to regulate the capability of viruses to influence microbial communities and functions in soils (27, 28).

In recent years, research addressing the role of viruses in soil ecosystems has steadily gained momentum (29–32). Viruses can impact soil microbes through predation control (called “top-down”) in the soil environment (10, 33, 34). First of all, viral abundances in soil can exceed 10^9^ g^−1^ virus-like particles, which is often an order of magnitude or greater than microbial abundance in aquatic environments (34–37). Second, the reproductive mode of viruses falls into two general categories: the lysogenic or the lytic cycle, which have different impacts on microbial activity. A temperate phage displays a lysogenic lifestyle and can be engaged in lytic or lysogenic reproduction (38). In lysogenic cycles, nucleic acid usually integrates into the host genome or a plasmid, and the provirus replicates as the host grows and divides. The initiation of the lytic cycle can be triggered by environmental factors, such as UV light, toxins, temperature, and host cell density dependence (39, 40). A lytic (or virulent) phage exploits the biosynthetic machinery of the host to produce, assemble, and release daughter phages by lysing the host cells immediately after infection (41). Infection efficiencies of lytic phages is relevant to phages type and their host (42). Lytic viruses with broad a host spectrum (i.e., myoviruses) may infect more hosts than viruses with specific host (i.e., podoviruses) (43, 44). The activities of host cellular functions influence the viral growth rate and further affect viral fitness, lysis time, or even burst size (42, 45). During the lytic cycle, viruses can also directly mediate host function through virus-encoded auxiliary metabolic genes (AMGs), including photosynthesis, central carbon metabolism, and nutrient cycling (46).

Bulk soil metagenomes are commonly used to study soil microbes (47). Viral information from bulk-soil metagenome sequencing can be obtained from a variety of bioinformatics tools designed to search and identify viral from nonviral genetic information (30–32). Using bulk-soil metagenome sequencing to distill the soil virus information is one of the important approaches to investigating the soil virus community and its ecological roles (32). Most previous studies revealed that agricultural management practices influence microbial functional diversity, but the diversity of soil viruses, their dynamic interactions with host microbes, and their overall ecological role in soil food webs is not well understood. Here, we exploit the bulk-soil metagenomes prepared from 12 samples across four agricultural treatments to recover virus operational taxonomic units (vOTUs) from and investigate the effect of long-term cover crop (e.g., vetch) and N-fertilization management on viral community composition and identify how N-fertilization and cover cropping impact the link between viruses and their host. We hypothesize that (i) new viruses will be identified, (ii) greater diversity of viruses will be observed in N-fertilization with cover crops than in other treatment, and (iii) the relative abundances of viruses will be closely correlated with their host microbes. Our objective is to provide an initial view of viruses and virus-host interactions in these agricultural soils to obtain a better understanding of the ecological roles of the viruses may have in agroecosystems in relation to various conservation management practices.

## RESULTS

### Data set overview and agricultural soil viral population (vOTU) recovery.

The sequences of the double-stranded DNA (dsDNA) viruses were extracted as described above from 12 agricultural soil samples from four conservation management practices. To quantify dsDNA viral diversity in long-term managed agriculture soil, we collected 12 soil metagenomes from the cores gathered from the long-term tillage, N-fertilization, and cover-cropping plots in western Tennessee. Approximate sequencing depths of 20-Gbp paired reads per metagenome were obtained by Illumina NovaSeq 6000 platform. Over 99% of raw reads per sample were passed through the quality control steps. After quality filtering, metagenome sequencing yielded a mean of 69,652,934 paired reads per library for 12 samples collected from the four agricultural management treatments.

The total of 260 vOTUs were predicted by VirSorter, DeepVirfinder, and VIBRANT pipeline. A total number of high-quality paired end reads that were obtained ranged from 51,805,430 to 90,119,576 with an average of 1.15 to 1.91% were assembled into contigs greater than 1 kbp. The reads were assembled to a mean of 4,922 soil viral contigs and ranged from 27,152 to 86,666 bp (Table 1). All soil viral contigs from the 12 samples clustered into 56,780 unique viral contigs, but only 241 viral contigs were greater than 10 kb. No plasmids were identified in 241 vOTUs based upon manual screening. Finally, the viral OTU table with 260 of vOTUs was generated by mapping reads to the updated PIGEON database (including the 241 viruses found in this study). The 260 vOTUs recovered in this study included the 10 named “alaska_puertorico” contigs obtained from permafrost (48), only 1 viral contig named “EarthsVirome_48737,” and 14 of the viral contigs named “gary_all20” (IMG/VR) and published by Paez-Espino et al. (49). Two viral contigs similar to “SPRUCE_viral_seq” orginally discovered from Peatlands in northern Minnesota were also identified (29), and one viral contig known as “virsorter_curated” recovered from the publicly available microbial genomes (RefSeq and WGS databases) (50). The rest of 232 out of 260 vOTUs (89.23%) were without significant hits to the databases we examined (see Table S1 in the supplemental material). The results indicated that the soil virosphere is undersampled and that different soil types may harbor previously unrecognized viruses.

**TABLE 1:** Bulk-soil metagenome and viral read information

Sample	Sample ID	Total no.			Assembled (%)	Total no.	
		PE reads	Assembled contigs (>1 kbp)	Assembled reads		Viral contigs	Virus reads
NCNTN0_1	M35	78,713,410	223,037	2,599,218	1.65	6,881	77,034
NCNTN0_2	M63	58,385,517	131,301	1,339,845	1.15	3,539	34,772
NCNTN0_4	M107	61,062,926	130,169	1,422,914	1.17	4,606	49,419
NCNTN60_1	M56	67,481,448	186,806	2,109,378	1.56	4,621	49,061
NCNTN60_2	M9	81,710,978	195,748	2,328,660	1.42	5,415	60,388
NCNTN60_4	M77	67,072,458	197,580	2,161,159	1.61	4,534	47,917
VNTN0_1	M37	76,463,359	214,116	2,584,629	1.69	5,930	66,678
VNTN0_2	M62	67,539,582	180,686	2,028,163	1.50	5,423	57,828
VNTN0_4	M112	51,805,430	109,048	1,204,616	1.16	2,592	27,152
VNTN60_1	M51	90,119,576	284,218	3,441,075	1.91	7,305	86,666
VNTN60_2	M16	61,552,488	140,778	1,645,990	1.34	3,675	40,775
VNTN60_4	M79	73,928,033	167,628	1,882,959	1.27	4,539	48,176

10.1128/msystems.00571-22.3 TABLE S1 vOTUs and putative phage types and evidence. Download Table S1, DOCX file, 0.04 MB. Copyright © 2022 Duan et al. 2022 Duan et al. https://creativecommons.org/licenses/by/4.0/ This content is distributed under the terms of the Creative Commons Attribution 4.0 International license.

### Gene-sharing network for viral taxonomy assignment.

**FIG 1:**
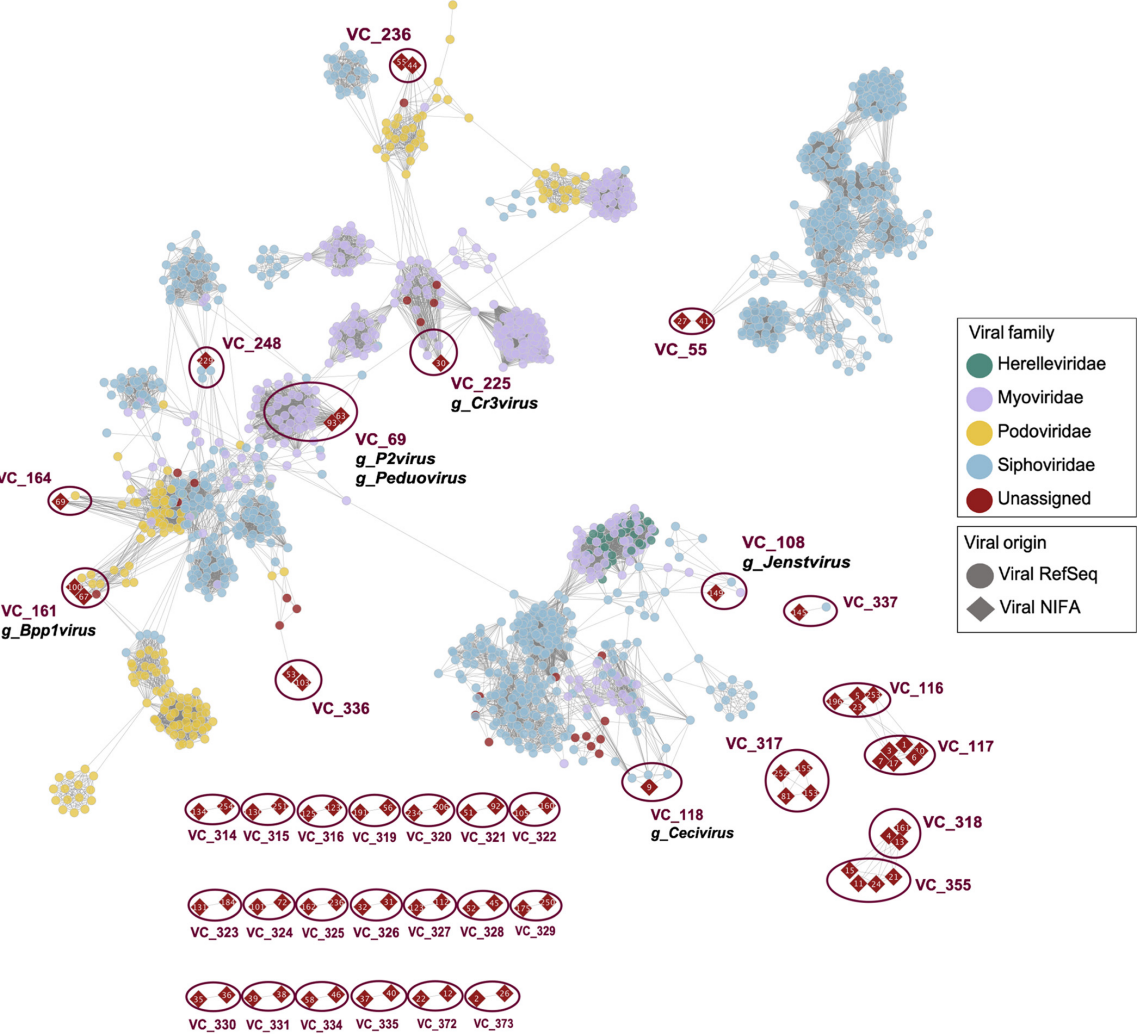
Gene-sharing network of vOTUs. Recovered vOTUs cluster with RefSeq (v94) viral genomes or fragments. The diamond shape with red colors represents the vOTUs recovered in our studies. Each round node is a virus obtained from the RefSeq database. Round shapes with different colors indicate major viral families in the database. Viral genome pairs with sufficiently similar (a significance score >1) were joined by an edge. The vOTUs classified as “outlier” are not shown in the network. “Viral Refseq” of viral origin represents viruses from the RefSeq (v94) database. “Viral NIFA” indicates the query viral candidates identified in this study (NIFA is the project name, which was founded by the National Institute of Food and Agriculture [USDA]).

**FIG 2:**
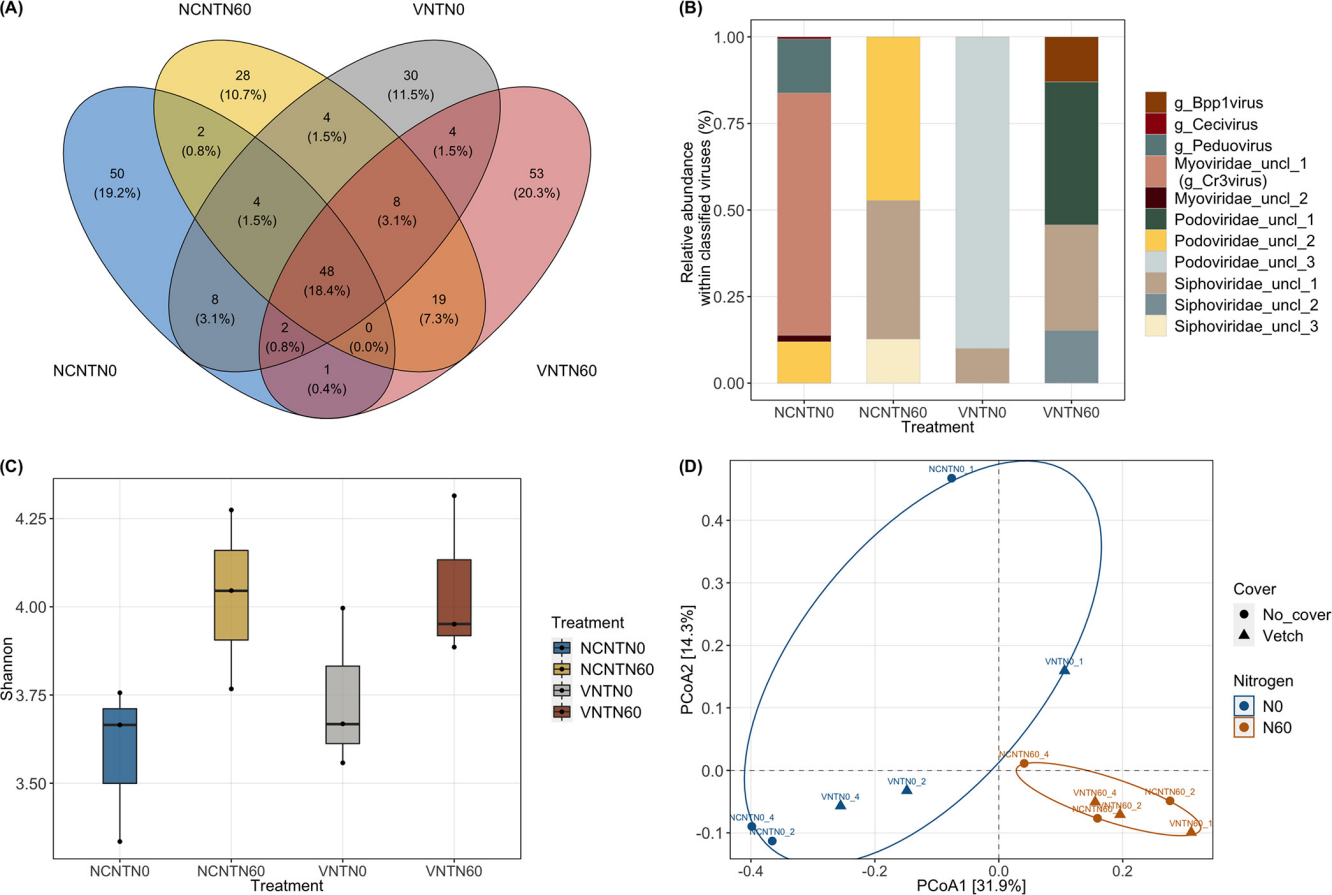
Distribution and diversity of vOTUs recovered in our study treatments. The difference was significant when results were grouped by N-fertilization management. NCNTN0 represent no cover, not tilled, with no N-fertilization; VNTN60_4 is vetch covered, not tilled, with N-fertilization. (A) Distribution of vOTUs across NCNTN0, NCNTN60, VNTN0, and VNTN60 treatment based on read mapping to the updated PIGEON database. (B) Stacked bar plot indicating the relative abundance of viral taxa in each treatment, considering that only 11 taxa can be clustered in gene-sharing networks (e.g., Podovirdae_uncl_1 and Podovirdae_uncl_2 have the same family but different genera). (C) Shannon index for 260 vOTUs across four treatments. (D) PCoA values for the viral community composition derived from read mapping to viral contigs with Bray-Cutis dissimilarities. Points represent samples. PERMANOVA with 2,000 permutations was applied. The ellipses in panel D are only used for better visualization.

The gene-sharing network was built to assign viral taxonomy due to the lack of universal phylogenetic marker genes in viruses (Fig. 1). The 260 vOTUs were clustered with 2,617 prokaryotic viral genomes in the RefSeq database (v94). The resulting network consisted of 1,658 nodes and 32,667 edges were left in the networks. Only 6% of the vOTUs identified in this study were similar to the viruses in the database, and 11 viral clusters (VCs) were related to previously curated clusters in the reference databases (Fig. 2B).

The vOTU_30 grouped in the same cluster (VC_225_1) as 5 Cronobacter_phage (VC_225_0) classified as genus *Cr3virus* (family *Myoviridae*), indicating that the two genomes were highly related at the subfamily level, which was *Vequintavirinae* (Fig. 1; see also Table S7). The vOTU_63 and vOTU_93 (VC_69) clustered with 28 viruses belonging to the genus *Peduovirus* affiliated with the *Myoviridae* family and were all P2-like viruses (Fig. 1; see also Table S7).

10.1128/msystems.00571-22.9 TABLE S7 Gene-sharing network information. Download Table S7, DOCX file, 0.4 MB. Copyright © 2022 Duan et al. 2022 Duan et al. https://creativecommons.org/licenses/by/4.0/ This content is distributed under the terms of the Creative Commons Attribution 4.0 International license.

With high probability, vOTU_229 was assigned to the *Siphoviridae*, which were grouped with Pseudomonas_phage_phiPSA1, Sinorhizobium_phage_phiLM21, and Vibrio_phage_SHOU24. vOTU_69 clustered with Ralstonia_phage_RSK1 in the same subcluster (VC_164_0), which belonging to the family *Podoviridae*, but the genus was unclassified due to the limitation on identification of genes. vOTU_100 and vOTU_67 connected with 2 viruses assigned to *Bpp1virus*, others were unclassified, but they grouped within the lineage of *Podoviridae* (Fig. 1; see also Table S7).

Most of the structural protein of vOTU_63 and vOTU_93 (e.g., baseplate protein, tail and head completion protein, tail tube protein, and phage major capsid protein) were similar to those of bacteriophage P2. Very late expression factor 1 (VLF-1), a member of the tyrosine recombinase family of proteins, serves the basic function during the late stage of DNA packaging and capsid assembly. This recombinase was detected in vOTU_63 and vOTU_93, indicating that the vOTU_63 and vOTU_93 might be temperate phages.

A portal protein was annotated in vOTU_100, which functions as a channel for passage of viral DNA bidirectionally, in tailed bacteriophages. DNA can move in and out of the virus head using portal protein which also provides an attachment point for the tail apparatus. vOTU_149 and *Brevibacillus* phage were in the same subcluster (VC_108_0), which was assigned to the genus *Jenstvirus* in the family *Siphoviridae*. vOTU_9 (Virsorter_curated_4650) clustered with *Bacillus* phage, were in the same subcluster (VC_118_0), which belongs to genus *Cecivirus* of the family *Siphoviridae*. vOTU_145 was in the VC_337_0 with *Thermoanaerobacterium*_phage, which is in the family *Siphoviridae*. The presence of recombinase, XerC, suggested that vOTU_145 may also be a temperate phage.

### Distribution and diversity of vOTUs across agricultural practices.

There were 8 vOTUs shared between no cover crops and vetch with no N-fertilization, and 19 vOTUs were shared by no-cover crops and vetch with N-fertilization (Fig. 2A). Only 4 vOTUs were shared by N-fertilization and no fertilization under vetch cover, and 3 were shared by the N-fertilization and no fertilization under no cover (Fig. 2A). These results suggest that N-fertilization may have a greater influence on shaping viral community than cover cropping and that there likely exists environmental specialization among soil viruses even at the small plot scale after long-term conservation management. A greater number of vOTUs that only exist in that treatment (i.e., that are not shared with other treatments) were found in no-cover with no N-fertilization and vetch with N-fertilization treatments compared to no-cover with N-fertilization and vetch without N-fertilization practices (Fig. 2A).

After annotating 260 vOTUs, only 8.5% (22 vOTUs) were possessed recombinase or recombinase-like protein, and 13.8% (36 vOTUs) were identified as prophage by BLAST and CRISPR arrays (see Table S1), and their putative hosts were found in Table S3. Here, the numbers of vOTUs that may be identified as temperate phage were 31.6, 28.6, 33.3, and 24.4% across no cover without fertilization, no cover with N-fertilization, vetch without N-fertilization, and vetch cover with N-fertilization, respectively. The estimated proportion of phages with integrase (31.6 and 33.3%) was greater in soil without N-fertilization than in N-fertilized soil (see Table S4).

10.1128/msystems.00571-22.5 TABLE S3 Coverage information of identified viral OTUs and potential bacterial and archaeal host. Download Table S3, DOCX file, 0.2 MB. Copyright © 2022 Duan et al. 2022 Duan et al. https://creativecommons.org/licenses/by/4.0/ This content is distributed under the terms of the Creative Commons Attribution 4.0 International license.

10.1128/msystems.00571-22.6 TABLE S4 Distribution of vOTUs across four treatments. Unique vOTUs were used in every treatment. “1” is present; “0” is absent. Download Table S4, DOCX file, 0.03 MB. Copyright © 2022 Duan et al. 2022 Duan et al. https://creativecommons.org/licenses/by/4.0/ This content is distributed under the terms of the Creative Commons Attribution 4.0 International license.

The alpha-diversity analyses revealed that soil with N-fertilization contained a significantly greater number of vOTUs than no N-fertilization soil (*P < *0.05; see Table S5). Also, the estimated diversity indices revealed that Shannon diversity (*P < *0.01), for example, was significantly greater in fertilized soil than soil without N-fertilization; however, cover crops had no apparent effects on alpha-diversity (Fig. 2C; see also Table S5). Principal coordinate analysis (PCoA) and permutational multivariate analysis of variance (PERMANOVA) were applied to the vOTU table (number of permutations = 2,000, *P < *0.01; see Table S6), resulting in 46.2% variation of the viral community, which could be explained by two axes of PCoA in total, suggesting that the N-fertilization-induced influence on the composition and structure of viral communities versus cover cropping was significant (Fig. 2D). No significant effect of POXC, inorganic N content, pH, or soil moisture content on viral community structure was observed (see Table S6).

10.1128/msystems.00571-22.7 TABLE S5 ANOVA on alpha diversity of vOTUs. Download Table S5, DOCX file, 0.04 MB. Copyright © 2022 Duan et al. 2022 Duan et al. https://creativecommons.org/licenses/by/4.0/ This content is distributed under the terms of the Creative Commons Attribution 4.0 International license.

10.1128/msystems.00571-22.8 TABLE S6 PERMANOVA results of the influence of treatments and environmental factor on vOTU community structure. Download Table S6, DOCX file, 0.04 MB. Copyright © 2022 Duan et al. 2022 Duan et al. https://creativecommons.org/licenses/by/4.0/ This content is distributed under the terms of the Creative Commons Attribution 4.0 International license.

### Identification of microbial host.

**FIG 3:**
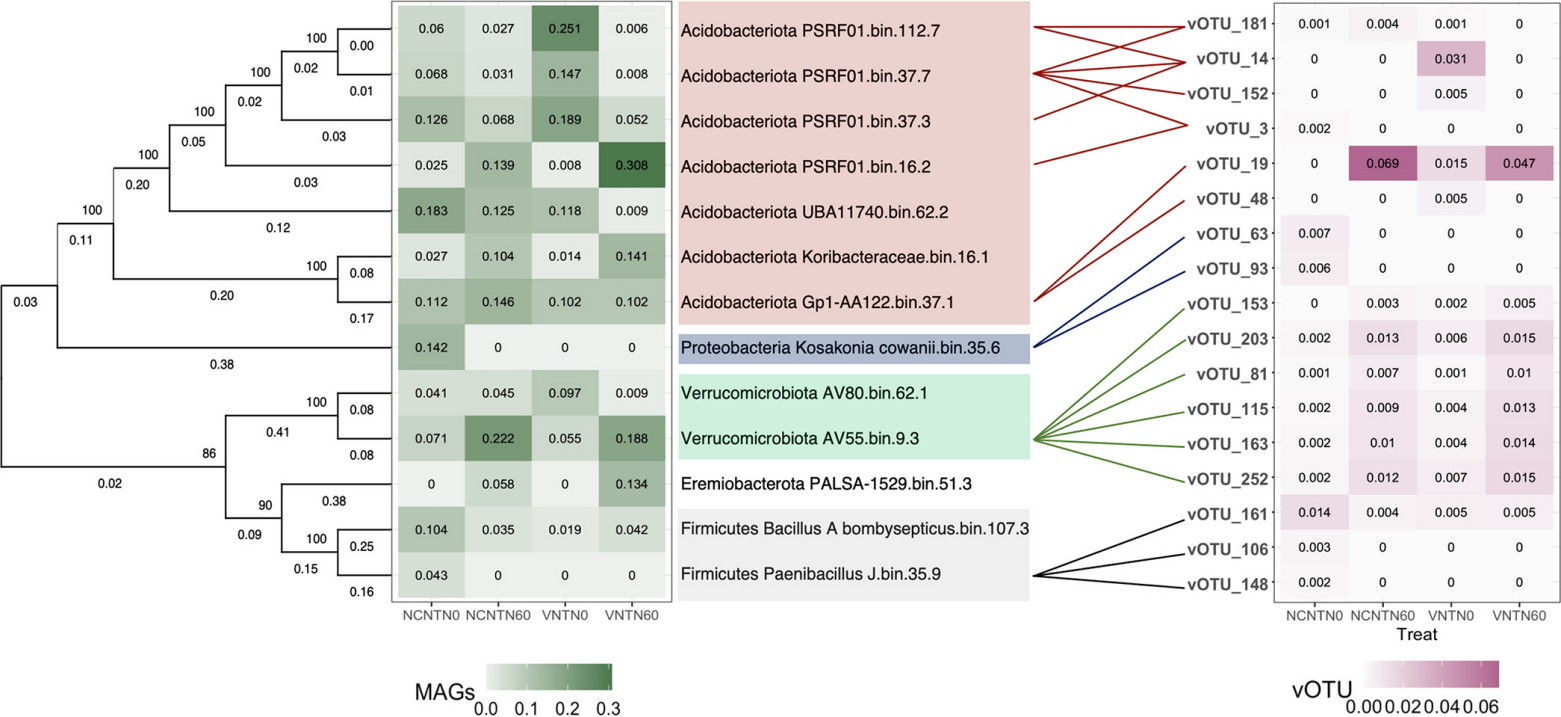
Predicated bacterial hosts for vOTUs. Unrooted phylogenetic tree of MAGs in the bacterial domain. The tree was built from concatenated protein sequences generated by alignment with single-copy genes defined by CheckM. Bootstrap values are shown as integer numbers near each node, representing the phylogenetic confidence of the tree topology. Branch lengths shown as decimals indicate the genetic change. The heatmaps represent the relative abundance of every MAG or vOTU in every treatment. The bacterial host and vOTU were connected according to CIRSPR and BLAST analysis (see Tables S2 and S3). Use of the same color indicates the same bacterial phyla.

**FIG 4:**
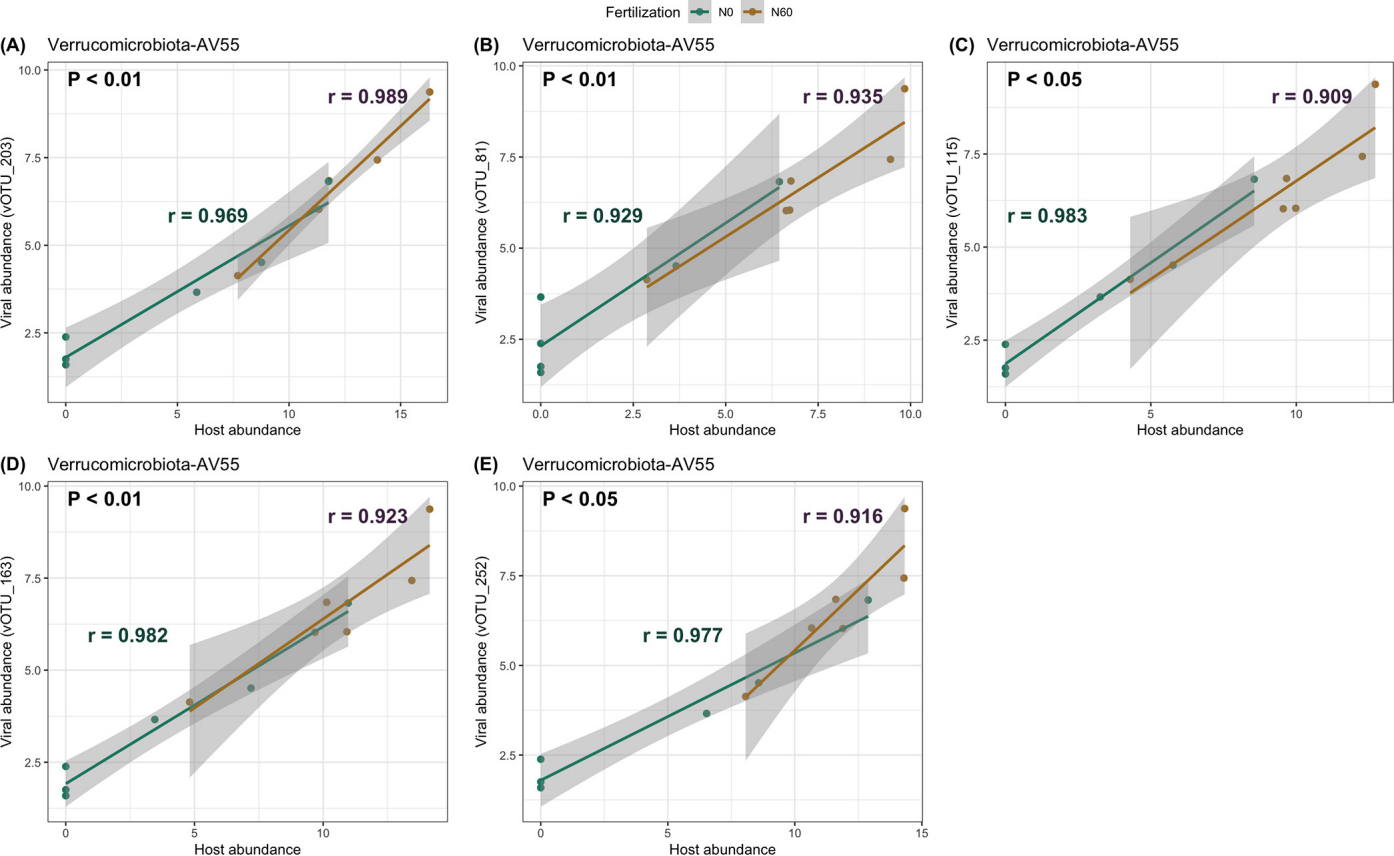
Abundance patterns of five vOTUs with host *Verrucomicrobiota AV55* (genus) for N-fertilization treatment. The five vOTUs are vOTU_203 (A), vOTU_81 (B), vOTU_115 (C), vOTU_163 (D), and vOTU_252 (E), respectively. Green and yellow represent no N-fertilization and N-fertilization, respectively. Viral and host abundance were calculated as the normalized mean coverage depth. Correlation coefficients (*r*) were calculated by using Pearson’s product-moment correlation. The normality of the data was checked by using the Shapiro-Wilk normality test before correlation analysis.

**FIG 5:**
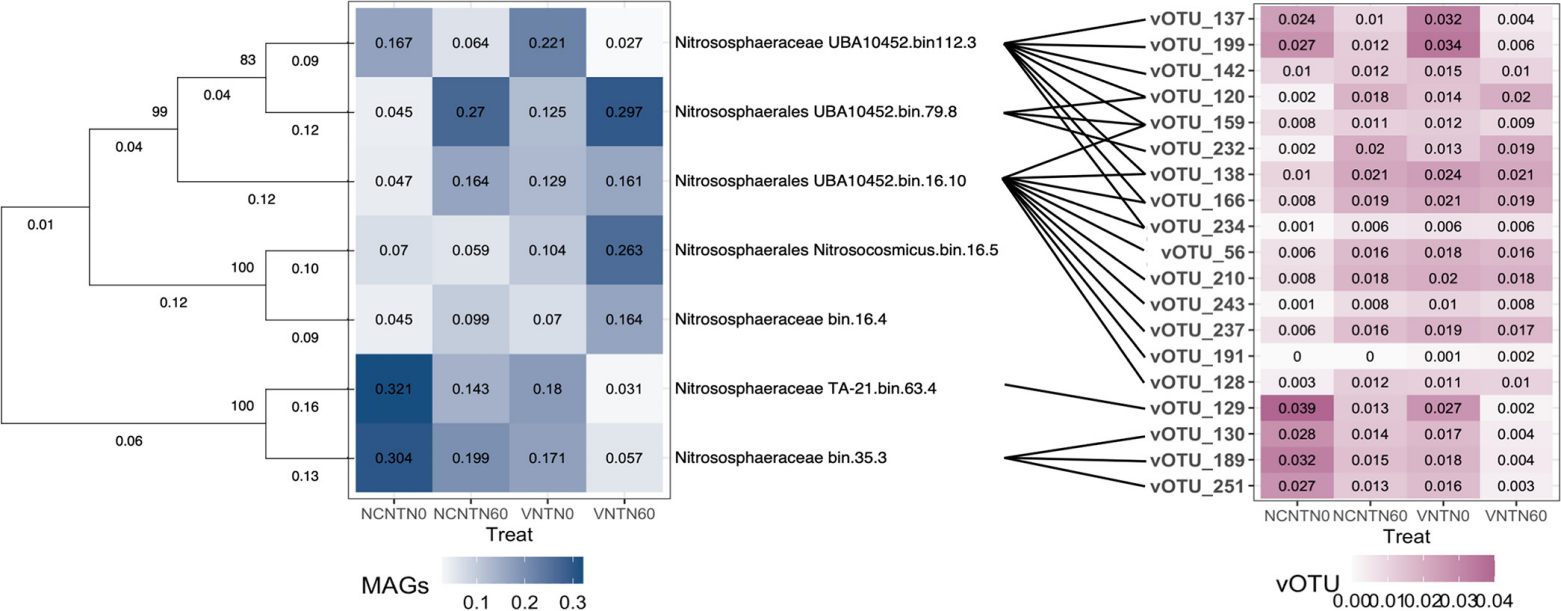
Predicated archaeal hosts for vOTUs. All archaeal genomes affiliated with *Thermoproteota*. An unrooted phylogenetic tree of MAGs under archaeal domain is shown. The tree was built from concatenated protein sequences generated by alignment with a single-copy gene defined by CheckM. Bootstrap values are shown as integer numbers near each node and represent the phylogenetic confidence of the tree topology. Branch lengths shown as decimals indicate the genetic change. The heatmaps represent the average relative abundance of every MAG or vOTU in every treatment.

We employed CRISPR and BLAST to link virus with potential microbial hosts. Fifty-two spacers within 39 spacer groups were identified from all 12 samples matched to our 260 vOTUs, and 39 direct repeats were blasted with the 13 bacterial and 7 archaeal genomes. In our study, more linkages between the virus and its host were detected by BLASTn with criteria described in Materials and Methods than by CRISPR-cas patterns. The bacterial hosts covered four bacterial phyla, including *Acidobacteriota*, *Proteobacteria*, *Verrucomicrobiota*, and *Firmicutes*, and five genera, including *PSRF01*, *Gp1-AA122*, *Kosakonia*, *AV55*, and *Paenibacillus*_*J* (Fig. 3; see also Table S3). The most abundant are genus *Kosakonia* (bin35.6, 14.21%, phylum *Proteobacteria*), *AV55* (bin9.3, 22.23%, phylum *Verrucomicrobiota*) (Fig. 4), *PSRF01* (bin112.7, 25.10%, phylum *Acidobacteriota*), and *PSRF01* (bin16.2, 30.83%, phylum *Acidobacteriota*) in no cover without fertilization, no cover with fertilization, vetch without fertilization, and vetch with fertilization treatments, respectively (see Fig. S1). All seven archaeal hosts are all in the lineage of *Nitrososphaeraceae* (phylum *Thermoproteota*) (Fig. 5). The most abundant are the genera *TA-21* (bin63.4, 32.11%), *UBA10452* (bin79.8, 27.02%), *UBA10452* (bin112.3, 22.11%), and *UBA10452* (bin79.8, 29.70%) in no cover without fertilization, no cover with fertilization, vetch without fertilization, and vetch with fertilization treatment, respectively (see Fig. S2).

10.1128/msystems.00571-22.1 FIG S1 Stacked bar plot of bacterial host MAGs. Bin 16.2 and bin 9.3 contain significantly greater abundance under fertilization treatment than under no fertilization (*P* < 0.05). Under no fertilization, the relative abundance of bin 37.7 decreased under vetch-covered soil (*P* < 0.05) and also significantly decreased in fertilized soil than in non-fertilized soil under vetch-covered treatment (*P* < 0.05) or no cover crop soil (*P* < 0.01). The relative abundance of bin 112.7 significantly decreased under fertilization compared to under no fertilization with no cover cropping (*P* < 0.05) and vetch cover cropping (*P* < 0.001), respectively. The relative abundance of bin112.7 decreased with vetch treatment under fertilized soil (*P* < 0.05) but increased with vetch treatment under nonfertilized soil (*P* < 0.05). Download FIG S1, DOCX file, 1.9 MB. Copyright © 2022 Duan et al. 2022 Duan et al. https://creativecommons.org/licenses/by/4.0/ This content is distributed under the terms of the Creative Commons Attribution 4.0 International license.

10.1128/msystems.00571-22.2 FIG S2 Stacked bar plot of archaeal host MAGs. Bins 112.3, 35.3, and 63.4 displayed significantly greater abundance under fertilization treatment than under no fertilization (*P* < 0.05), but bin79.8 displayed the opposite trend. Under no cover, the abundance of bin 16.10 is significantly greater in fertilization than no fertilization (*P* < 0.01); under no fertilization, the vetch cover has a higher archaeal bin 16.10 than under no fertilization (*P* < 0.01). Download FIG S2, DOCX file, 2.3 MB. Copyright © 2022 Duan et al. 2022 Duan et al. https://creativecommons.org/licenses/by/4.0/ This content is distributed under the terms of the Creative Commons Attribution 4.0 International license.

10.1128/msystems.00571-22.4 TABLE S2 Evidence of link putative viruses and their bacterial hosts. Download Table S2, DOCX file, 0.1 MB. Copyright © 2022 Duan et al. 2022 Duan et al. https://creativecommons.org/licenses/by/4.0/ This content is distributed under the terms of the Creative Commons Attribution 4.0 International license.

### Linkage between viruses and bacterial host.

The result suggested that multiple viruses within the same viral genus were able to infect the same genus of putative host. For example, *Verrucomicrobiota AV55* was identified as the putative the host of vOTU_153, vOTU_81, and vOTU_252. vOTU_153, vOTU_81, and vOTU_252 were annotated within the same genus. vOTU_63 and vOTU_93 were linked to the putative host within the *Proteobacteria* Kosakonia cowanii species, and vOTU_63 and vOTU_93 were clustered together in VC_69 and belong to *Peduovirus* (or *P2virus*) in the *Myoviridae* lineage (see Table S7).

Novel viruses that share the same potential host may not necessarily do so within the same viral genus. For example, *Acidobacteriota PSRF01* in different species (bin.112.7, bin 37.3, and bin 37.7) was linked to the potential hosts vOTU_14, vOTU_181, vOTU_3, and vOTU_152. vOTU_14 could not be assigned to a taxonomy through gene-sharing networks, and vOTU_3 did not cluster with vOTU_181, vOTU_14, and vOTU_152 at the genus level (Fig. 3; see also Table S3).

A linear mixed-effects model was built to test the effect of N-fertilization and cover crops on the abundances of viruses and their host, respectively. The relative abundances of *Paenibacillus* (*Acidobacteriota*) (*P < *0.05) and g_*AV55* (*Verrucomicrobiota*) (*P < *0.001) increased in N-fertilization compared to no N-fertilization. For viruses, the relative abundances of vOTU_19, vOTU_203, vOTU_81, vOTU_115, vOTU_163, and vOTU_252 were significantly greater in N-fertilized soil. No significant effect of cover crops on the relative abundance of hosts and viruses was found (Fig. 3). Furthermore, vOTU_203, vOTU_81, vOTU_115, vOTU_163, and vOTU_252 were analyzed separately based on the different N-fertilization treatments. Similarly, the abundances between virus and host were positively correlated (Fig. 4).

### Linkage between viruses and archaeal host.

**FIG 6:**
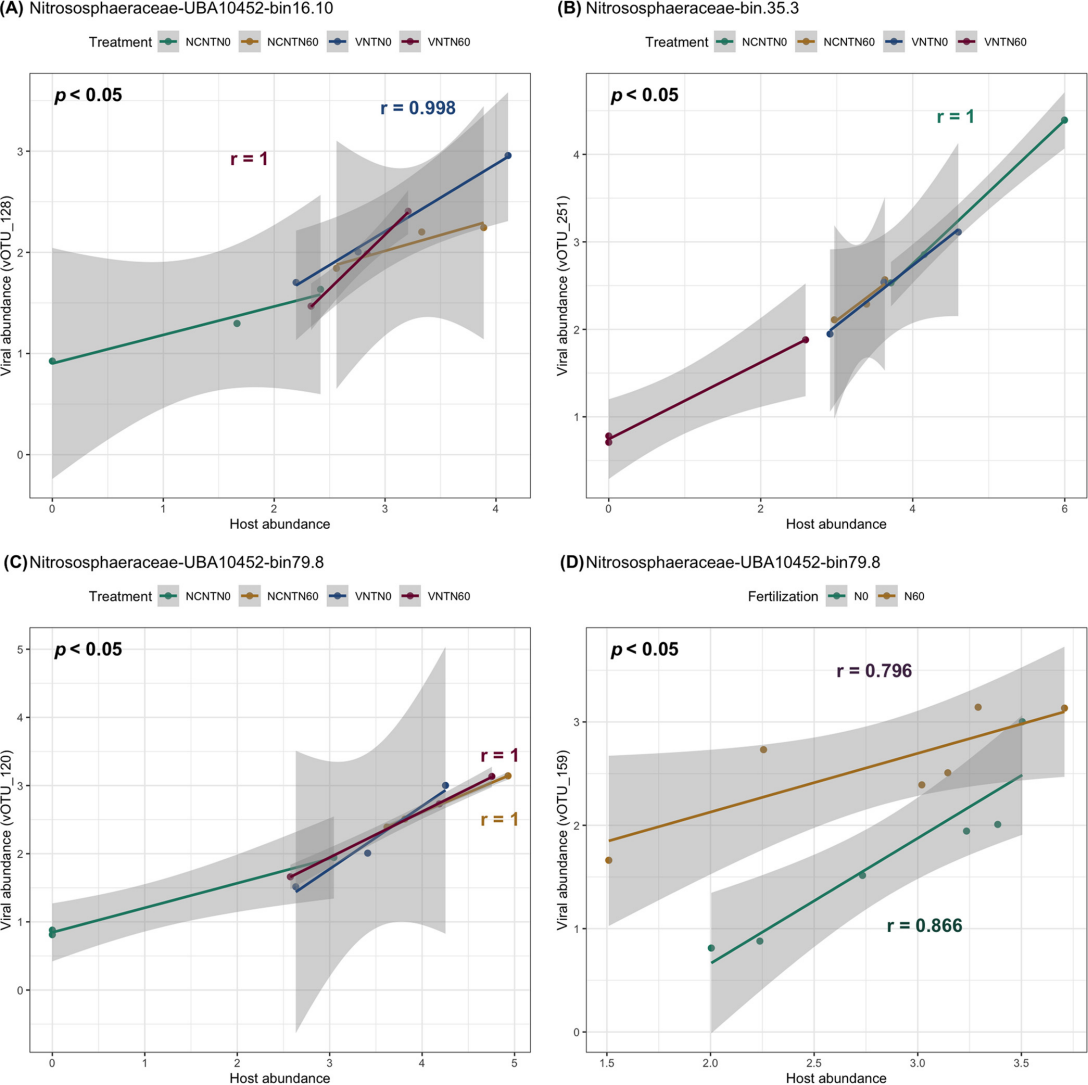
Abundance patterns of four viruses with the archaeal host *Thermoproteota* (phyla) *Nitrososphaeraceae* (family) across four different treatments. Green, yellow, blue, and red in panels A to C represent NCNTN0, NCNTN60, VNTN0, and VNTN60, respectively. Green and yellow in panel D represent no N-fertilization and N-fertilization, respectively. The viral and host abundance was calculated as the normalized mean coverage depth. Correlation coefficients (*r*) were calculated by using Pearson’s product-moment correlation. The normality of the data was checked by using the Shapiro-Wilk normality test before the correlation analysis.

All of the taxonomy of the vOTUs connected to *Nitrososphaeraceae* could not be assigned using gene-sharing network due to its novelty or the lack of virus hallmark genes. We observed that *Nitrososphaeraceae* were significantly increased under cover crops (e.g., genus *Nitrosocosmicus* bin 16.5) (*P < *0.05, no significant fertilization effect) or under no N-fertilization (e.g., genus *UBA10452*, *TA21*, and bin35.3) (*P < *0.05, no significant cover-cropping effect). The correlation between the relative abundances of vOTUs and archaea were positively correlated like bacterial phages in our study (Fig. 6; see also Table S3). Moreover, the correlation between abundances of the vOTUs (vOTU_128, vOTU_251, vOTU_120, and vOTU_169) and the potential *Nitrososphaeraceae* host significantly correlated, indicating that both cover-cropping management and N-fertilization can influence the relationship between the vOTUs and the *Nitrososphaeraceae* (Fig. 6).

## DISCUSSION

### Long-term inorganic N-fertilization influenced the viral community diversity and structure.

Agricultural soils are relatively fertile, having balanced components, including minerals, soil organic matter, air, and water, which provide more nutrient and physical support for crop growth. Agricultural activities, including fertilization and cover cropping, can increase inputs of soil organic matter, soil moisture, and microbial activities (19, 20), which may influence the mobility, survival rate, and distribution of soil viruses (51–53). In this study, inorganic N-fertilization positively influenced viral community diversity and played a role in shaping viral community structure. It is likely N-fertilization positively influenced viral population through increasing abundance of host communities (53–56). Also, a strong positive correlation between the abundances of *Verrucomicrobiota* and its vOTUs was observed, and N-fertilization influenced the abundance of genus *PSRF01* affiliated with *Blastocatellia*; the abundance of *Blastocatellia* was correlated with nitrogen availability in soils (57), which might indirectly influence the viruses related to *Blastocatellia*. The results in this study also supported the idea that variation of the viral population may directly result from changes in the host population or abundances under N-fertilization.

The survival strategy of bacteria can change (e.g., endospore formation in *Firmicutes*) due to the lack of nutrients (such as N) or their surroundings (58). Phages could respond to the bacterial signals and modify the outcome of their infection from obligately productive to temporarily reductive (59, 60). For example, phages could be packed into bacterial endospores and use them as a viral genome protection shell to resist environmental stress (59). Spore formation for septum- and coat assembly-related AMGs (*spoVS*, *whiB*, and *spoIIIE*, etc.) have been reported in viral genomes (32, 61). This mechanism is supported by previous studies reporting that lysogeny is an effective strategy to maintain the viral population survival in a detrimental soil microenvironment (10, 32, 39, 62). Accordingly, we observed more vOTUs with integrase, which might be temperate phage in unfertilized soil, than in soils that received long-term N-fertilization.

### Roles of viruses in influencing C and N cycling via putative hosts.

Different vOTUs linked to the same *Acidobacteriota*, *Proteobacteria*, *Verrucomicrobiota*, and *Firmicutes* as the host have been reported in other studies (31, 32). Multiple vOTUs are related to *Chthoniobacterales* within the phylum *Verrucomicrobiota*. N-fertilization increased the abundance of *Chthoniobacterales* species. *Chthoniobacterales* have contributed to carbon cycling by decomposing carbohydrates, such as cellulose and xylan (63). The results indicated that *Chthoniobacterales* phage could influence carbon transformations by controlling its host’s metabolism. *Nitrososphaeraceae* affiliated with the *Thaumarchaeota* phylum are ammonia oxidizers, which play important roles in nitrification (64–66). It has been observed that ammonia-oxidizing archaea can grow in a wide range of ammonia concentrations (64, 67, 68). The close positive correlation between the relative abundance of virus and its predicted host provided the evidence that the soil viruses found in long-term managed agricultural soil tend to control carbon and nitrogen cycling by infecting the functional hosts, which is consistent with results reported in other soil systems (29, 31, 32).

### Limitations of using metagenomes to discover soil viruses.

The recovery rate of vOTU from bulk soil metagenomes is somewhat inefficient but increases with increasing numbers of samples and/or sequencing depth. About 2,699 vOTUs across 82 bulk soil metagenomes were identified from peatland in northern Minnesota; all of these total soil metagenomes were sequenced from 6 to 15 Gbp on average per library on the NovaSeq platform (29). In a recent study of Stordalen Mire, 1,831 vOTUs were identified from 178 bulk soil metagenomes in northern Sweden permafrost soil with an average output of 8 Gbp per metagenomic sample (31, 32). In biochar-amended agricultural soil, 97 vOTUs were identified from across 16 samples at a depth of ~2 Gbp per library from bulk soil metagenome sequencing (69). Based on the studies described above, the vOTU yield rate ranged from (normalized per gigabase pair of metagenomes) 1.30 to 3.29 number of vOTU/Gbp. To increase the efficiency of the vOTU recovery rate or to decrease the complexity of soil samples based on the purpose of types of different research, virome, stable isotope probing, and different-size filters can be combined with metagenomes to decrease the noise of the microorganism (69), to target active viruses (48), or to concentrate cells with specific sizes in order to study rare soil biosphere (70).

Here, the high-quality sequences obtained reached up to ~10-Gbp paired reads per library, and 260 vOTUs (including 232 novel vOTUs and 28 overlapped with PIGEON database) were identified from 12 agricultural soil DNA samples. The vOTU yield rate of this study was 2.17, which is a reasonable recovery rate for soil studies. However, the use of a virus-enriched virome is a better approach for recovering more vOTU from soils compared to bulk soil metagenomes, since they can have about 3 to 30 times more viral populations per sample, which means that >90% of the viral information can be lost when using only bulk soil metagenomic sequencing compared to viromic sequencing (29, 32, 69). Viromics can provide the most viral information for even rare viral species, although viromes lack host information and can be lab intensive to prepare (30, 69). However, combining bulk soil metagenome and viromes is an ideal way to study the links between soil viruses and their hosts. A trade-off between the number of samples and the sequencing depth should be based on the aim and budget of the researcher’s study. In the present study, the taxonomy of >90% of viruses could not be assigned according to known bacterial viruses in the RefSeq database. This finding suggests that the soil environment is home to many diverse and yet-to-be-classified viral taxa. The limited information in current viral databases is one of the challenges that restricts our understanding of soil viral communities (10, 30, 32).

Lacking marker gene present a great challenge to identifying temperate or a virulent phage. A more credible way, compared to the existence of integrase, is to identify high-frequency functional genes on a viral genome; for example, temperate viruses usually contain integrase, excisionase, and DNA polymerase (*polA*) genes with leucine substitution, etc. (71–73). Relatively accurately classifying the vOTUs in our study can be further verified using novel bioinformatic tools (i.e., DeePhage) for future study (74).

### Conclusion.

The 260 vOTUs, including 232 novel viruses and 28 previously reported, were identified from 12 bulk soil metagenomes with 10-Gbp output per library. The samples represented a single soil type but had been treated for >40 years with various conservation management practices with combinations of inorganic N-fertilization and cover cropping. Multiple vOTUs at the genus level or at a higher level were potentially linked to the microbial genus level or higher within the same phyla. The microbial host populations were taxa that decompose carbohydrates (i.e., *Chthoniobacterales*) and oxidize ammonia (i.e., *Nitrososphaeraceae*). Long-term inorganic N-fertilization had a greater influence on viral alpha-diversity and community structure than did cover cropping. The findings indicated a close relationship between viruses and host microbes, suggesting that viruses could modulate abundance in hosts and may further influence the functional behavior of the host in C and N cycling. Although bulk-soil metagenomic analysis is an effective approach for detecting both viruses and their hosts, this approach may underestimate the diversity of soil viruses. Therefore, the use of viromics as a complement for studying viral ecology is suggested in future studies.

## MATERIALS AND METHODS

### Site description and sample collection.

The ongoing long-term conservation management experimental site used in this study is located at the West Tennessee Research and Education Center (WTREC; Jackson, TN), established in 1981, and the soil type was Lexington silt loam (fine-silty, mixed, 127 thermic, Ultic Hapludalf) (56). The continuous cotton production experiment was arranged in a randomized complete block with a split-split plot design. Inorganic nitrogen fertilizer (ammonium nitrate [NH_4_NO_3_]) is applied at two nitrogen levels (0 and 67 kg/ha) as the main plots and is divided into three subplots that contain two levels of cover crop treatments (no cover and hairy vetch, *Vicia villosa* Roth) and no-tillage treatment. All four treatments with three replications were sampled on 21 May 2019, shortly after the burndown of the cover crop and just before planting in the spring. The samples were coded as follows: “type of cover crop,” “no-tillage,” “N-fertilization or not,” and “serial number of replicates,” e.g., NCNTN0_1 represents no-cover, no-tilled, and no N-fertilization treatment of replicate 1, whereas VNTN60_4 is vetch-covered, no-tilled with N-fertilization of 67 N kg/ha (60 N lb/acre), replicate 4. Bulk soil metagenomic sequencing was applied to 12 samples from selected treatments.

### Soil properties.

The soil pH was measured using an electrode (Ul-trabasic; Denver Instrument, Bohemia, NY). The water content was determined gravimetrically at room temperature (~25°C). Measurement of the NO_3_^–^ and NH_4_^+^ concentration was performed using microplate-based spectrophotometric determinations (75, 76). Permanganate-oxidizable carbon extraction and measurement were conducted using a microplate reader (96-well microplate) at 550 nm as previously reported (77, 78).

### DNA extraction, library construction, and metagenomic sequencing.

Soil samples were collected from a depth of 0 to 10 cm at a distance of 10 to 15 cm from the center of the crop row using a 2.5-cm-diameter soil probe. About 10 to 15 subsamples were randomly taken within each plot. The samples were transported in a cooler with ice bags within 24 h to the lab freezer (–20°C). Preprocessing and DNA extraction were accomplished within 48 h after coming back from the field.

First, fine rocks, roots, and other debris were removed by passing each composite soil sample through a 2-mm sieve. Second, total soil DNA was extracted using a DNeasy PowerLyzer Powersoil kit (catalog no. 12855; Qiagen) from 0.25 g of soil per sample according to the manufacturer’s instructions for the kit. The extracted DNA was dissolved in sterile DNA-free PCR-grade water. A Qubit 1× dsDNA HS (high sensitivity) assay kit (Invitrogen, catalog no. Q33230) was used to evaluate the quantity of whole soil DNA on a Qubit 2.0 fluorometer (total 200-μL reaction). *A*_260_/*A*_280_ and *A*_230_/*A*_280_ values were measured to examine the DNA quality using a NanoDrop OneC Microvolume UV-Vis spectrophotometer (Thermo Scientific).

Genomic DNA libraries were constructed for sequencing on an Illumina platform using a KAPA library preparation kit (Kapa Biosystems, Woburn, MA). First, DNA was fragmented with a Covaris E210. Then, libraries were prepared using a modified version of the manufacturer’s protocol. DNA was purified between enzymatic reactions, and size selection of the library was performed with SPRI select beads (Beckman Coulter Genomics, Danvers, MA). For indexed samples, a PCR amplification step was performed with primers containing dual unique barcode sequences 8 nucleotides in length.

Libraries were assessed for concentration and fragment size using a DNA high-sensitivity assay on a LabChip GX (Perkin-Elmer, Waltham, MA). The library concentrations were also assessed by qPCR using the KAPA library quantification kit (Complete, Universal; KAPA Biosystems). The libraries were pooled and sequenced on an Illumina NovaSeq6000 S2 using 150-bp PE reads (0.5 S2-lane, 900M read pairs, 275-Gbp yield average; Illumina, San Diego, CA) at the Institute for Genome Sciences, School of Medicine, at the University of Maryland.

### Reads preprocessing and assembly.

Cutadapt (v1.18) was used to remove low-quality reads (the quality phred score cutoff was 20, the maximum trimming error rate was 0.1, and the reads were shorter than 50 bp) (79). *De novo* assembly was performed using MEGAHIT (80, 81). QUAST (v.5.0.2) was used to estimate contig statistics (82).

### vOTU identification.

The assemblies in each sample were clustered with PSI-CD-HIT implementation of BLASTn to cluster contigs with a global identity threshold of 0.95 to remove the redundant sequences (dereplication) (83–85). Nonredundant assemblies were processed by VirSorter (v.1), DeepVirFinder (86, 87), and VIBRANT (v1.2.0) (88). Category 1, 2, 4, and 5 viral contigs, as identified by Virsorter, were retained. Categories 3 and 6 were removed due to the lack of hallmark genes or the absence of enrichment in viral or non-*Caudovirales* genes (86, 89). Contigs with DeepVirFinder scores higher than 0.9 and a *P* value of *<*0.05 and phages predicted by VIBRANT (v1.2.0) were considered (88). All of the contigs were manually combined in each sample. All of contig lengths greater than 10,000 bp were clustered into vOTUs using CD-HIT with a global identity threshold of 0.95 and an alignment coverage for shorter contigs length of >85% (90). A bbmap was used to map short-read sequences in each sample to the updated PIGEON database (viral database PIGEON v1.0; Phages and Integrated Genomes Encapsidated Or Not [https://datadryad.org/]) plus the viral contigs assembled across the samples (29, 91). A SAM file with alignment information for each sample was transformed to the Bam files and then sorted and indexed by SAMtools (92). BEDtools (93) was used to parse the Bam file generated by the last step. A coverage table was generate using BamM (http://ecogenomics.github.io/BamM). The vOTU table was normalized based on the contig length and library size to make it comparable among samples (29, 31, 32). vOTUs with low coverage (<0.25×) of its length when mapped by reads were filtered and converted to zero (29, 31). Square-root transformation was applied to correct the data normality for statistical analysis.

Viral taxonomy assignment was performed by building the gene-sharing network in vContact2 (94). The 260 vOTUs generated by reading mapping mentioned above was annotated by MetaProdigal (95), and the output file with amino acid annotation was fed into vContact2 for taxonomy assignment. Nodes and edges represented viral genomes (or contigs) and significant similarities between protein cluster profiles, respectively. Similarity between sequences depends on the number of shared protein clusters (96). vOTUs were clustered with 2,617 viral genomes in the RefSeq database (v94) based upon shared protein clusters (97). Shared genus level viral clusters were selected manually for further analysis.

### Metagenome-assembled genome construction.

Identified viral contigs across 12 samples were removed from all contigs assembled by MEGAHIT mentioned above. Metabat2 (v2.12.1) (98), Maxbin2 (v2.2.6) (99, 100), and concoct (1.0.0) (101) within MetaWRAP (v1.3.2) (102) were applied on the microbial contigs assembled by megahits >2.5 kbp on each sample to recover the microbial draft genomes. CheckM (103) was used to assess the quality of the genome, and those with quality score of ≥50 were retained (104). Metagenome-assembled genomes (MAGs) were deduplicated by dRep (v3.0.0) (105), and taxonomic affiliations were classified with the GTDB-tk (v1.3.0) workflow (106).

To estimate the relative abundance of each MAG, the short-read sequences were mapped to assembled contigs before binning as described above. First, the contigs in all the samples were combined, and all of the contigs were clustered using CD-HIT with a global identity threshold of 0.95 and an alignment coverage for shorter contig length of >85%. The renamed, deduplicated, and sorted fasta file as reference contigs were indexed by using bbmap, which was similar to the approach for the viral abundance calculation. Third, all of the clean reads in every sample were mapped back to the reference contigs by using bbmap. This step was used to generate the coverage table for each sample. A final OTU table was generated using the weighted contig length in base pairs and then calculating the average of all its binned contig coverages; the MAG abundance table was normalized by the read depth for each sample as described for the vOTU table above. The square-root transformation was also applied for statistical analysis.

### Phylogenetic tree for the host genome.

Alignment of 43 conserved maker genes with largely congruent phylogenetic histories was performed, and concatenated protein sequences of single-copy genes were generated by CheckM (v1.1.3) and used to build an unrooted phylogenetic tree (103). Maximum-likelihood phylogenetic trees were built by using the unweighted pair-group method with arithmetic means (UPGMA) as the distance method and 500 bootstraps as the phylogeny test. The trees were constructed using the Le_Gascuel_2008 substitution model (107) by MEGA X (108, 109). The percentage of trees clustered at the nodes and the genetic divergence (branch length) are shown within the tree.

### Virus-host linkage based on CRISPR arrays and BLAST.

We used two methods to identify the link between viruses and their hosts. First, CRISPR repeats and spacer arrays were applied for the 12 samples by Crass v1.0.1 using the default settings individually (110). Viral contigs and spacer sequences (protospacer-spacer matches) recovered from metagenomes were compared by using BLASTn (blastn-short task, percent identity 0.95, and an E value threshold of 10e–5, mismatch ≤ 1) (111). The direct repeats were chosen based upon the spacer matches in viral genomes, and BLASTn was used against direct repeats to bacterial and archaeal genomes (blastn-short task, percentage identity 1, and E value threshold of 10e–10, mismatch ≤ 1) to link the virus with its putative host.

The second approach identified vOTU nucleotide sequences using BLAST against the MAGs in the soil samples. The vOTUs were retained if the bit score was >50, the E value was <10e–3, and the sample exhibited a ≥70% average nucleotide identity (ANI), as determined using FastANI (112). Hits that were ≥2,500 bp and covered ≤ 90% of viral contigs in microbial genomes were considered for the most confident provirus predictions, and if the hits covered >90% they were considered less confident (31, 32, 113). The Pearson’s product-moment correlation was determined for both viruses and their potential host normalized abundances. Normality tests were conducted using a Shapiro-Wilk test prior to correlation analysis (114). vOTUs were categorized as temperate or virulent based upon the presence or absence of an integrase (88).

### Statistical analysis.

A square-root transformation was performed to correct the normality of the relative abundance of viruses and microbes. A Shapiro-Wilk normality test was performed to check the normality (115). A mixed-effect model was built, the block was regarded as a random effect, and N-fertilization and cover crops were treated as fixed effect. Type II Wald chi-square tests were applied on the mixed model to test an alternative hypothesis. A Tukey’s HSD (honest significant difference) test was performed for the *post hoc* test (116).

### Data availability.

All sequencing data have been deposited in in National Center for Biotechnology Information database (Sequence Read Archive) under BioProject accession number is PRJNA820715. vOTUs were deposited in GenBank database (under accession numbers ON448394 to ON448625, see Table S8 for details). The information of bacterial and archaeal draft genomes can be obtained in the NCBI database (see Table S8 for the BioSample accession number).

10.1128/msystems.00571-22.10 TABLE S8 Accessions of viral contigs and bacterial and archaeal draft genome in the NCBI database. Download Table S8, DOCX file, 0.03 MB. Copyright © 2022 Duan et al. 2022 Duan et al. https://creativecommons.org/licenses/by/4.0/ This content is distributed under the terms of the Creative Commons Attribution 4.0 International license.

## References

[1] Wommack KE, Nasko DJ, Chopyk J, Sakowski EG. 2015. Counts and sequences, observations that continue to change our understanding of viruses in nature. J Microbiol 53:181–192. doi:10.1007/s12275-015-5068-6. 25732739

[2] Fuhrman JA. 1999. Marine viruses and their biogeochemical and ecological effects. Nature 399:541–548. doi:10.1038/21119. 10376593

[3] Clokie MR, Millard AD, Letarov AV, Heaphy S. 2011. Phages in nature. Bacteriophage 1:31–45. doi:10.4161/bact.1.1.14942. 21687533 PMC3109452

[4] Azam F, Fenchel T, Field JG, Gray J, Meyer-Reil L, Thingstad F. 1983. The ecological role of water-column microbes in the sea. Mar Ecol Prog Ser 10:257–263. doi:10.3354/meps010257.

[5] Fuhrman J. 1992. Bacterioplankton roles in cycling of organic matter: the microbial food web, p 361–383. *In* Primary productivity and biogeochemical cycles in the sea. Springer, New York, NY.

[6] Kimura M, Jia Z, Nakayama N, Asakawa S. 2008. Ecology of viruses in soils: past, present, and future perspectives. Soil Sci Plant Nutr 54:1–32. doi:10.1111/j.1747-0765.2007.00197.x.

[7] Suttle CA. 2007. Marine viruses: major players in the global ecosystem. Nat Rev Microbiol 5:801–812. doi:10.1038/nrmicro1750. 17853907

[8] Breitbart M. 2012. Marine viruses: truth or dare. Annu Rev Mar Sci 4:425–448. doi:10.1146/annurev-marine-120709-142805. 22457982

[9] Sokol NW, Slessarev E, Marschmann GL, Nicolas A, Blazewicz SJ, Brodie EL, Firestone MK, Foley MM, Hestrin R, Hungate BA, Koch BJ, Stone BW, Sullivan MB, Zablocki O, Pett-Ridge J, LLNL Soil Microbiome Consortium. 2022. Life and death in the soil microbiome: how ecological processes influence biogeochemistry. Nat Rev Microbiol 20:415–430. doi:10.1038/s41579-022-00695-z. 35228712

[10] Williamson KE, Fuhrmann JJ, Wommack KE, Radosevich M. 2017. Viruses in soil ecosystems: an unknown quantity within an unexplored territory. Annu Rev Virol 4:201–219. doi:10.1146/annurev-virology-101416-041639. 28961409

[11] Cardoso EJBN, Vasconcellos RLF, Bini D, Miyauchi MYH, Santos C, Alves PRL, Paula A, Nakatani AS, Pereira J, Nogueira MA. 2013. Soil health: looking for suitable indicators. What should be considered to assess the effects of use and management on soil health? Sci Agric (Piracicaba, Brazil) 70:274–289. doi:10.1590/S0103-90162013000400009.

[12] Van Bruggen AH, Semenov AM. 2000. In search of biological indicators for soil health and disease suppression. Appl Soil Ecol 15:13–24. doi:10.1016/S0929-1393(00)00068-8.

[13] Jackson L, Calderon F, Steenwerth K, Scow K, Rolston D. 2003. Responses of soil microbial processes and community structure to tillage events and implications for soil quality. Geoderma 114:305–317. doi:10.1016/S0016-7061(03)00046-6.

[14] Allison SD, Martiny JB. 2008. Resistance, resilience, and redundancy in microbial communities. Proc Natl Acad Sci USA 105 (Supplement 1):11512–11519. doi:10.1073/pnas.0801925105. 18695234 PMC2556421

[15] Trivedi P, Delgado-Baquerizo M, Trivedi C, Hu HW, Anderson IC, Jeffries TC, Zhou JZ, Singh BK. 2016. Microbial regulation of the soil carbon cycle: evidence from gene-enzyme relationships. ISME J 10:2593–2604. doi:10.1038/ismej.2016.65. 27168143 PMC5113853

[16] Torsvik V, Øvreås L. 2002. Microbial diversity and function in soil: from genes to ecosystems. Curr Opin Microbiol 5:240–245. doi:10.1016/S1369-5274(02)00324-7. 12057676

[17] Mbuthia LW. 2014. Long-term impacts of tillage, cover crops, and nitrogen rates on microbial community dynamics and soil quality parameters under continuous cotton production in west Tennessee. https://trace.tennessee.edu/cgi/viewcontent.cgi?article=4078&context=utk_graddiss.

[18] Bending GD, Turner MK, Jones JE. 2002. Interactions between crop residue and soil organic matter quality and the functional diversity of soil microbial communities. Biochemistry 34:1073–1082. doi:10.1016/S0038-0717(02)00040-8.

[19] Rousk J, Brookes PC, Baath E. 2009. Contrasting soil pH effects on fungal and bacterial growth suggest functional redundancy in carbon mineralization. Appl Environ Microbiol 75:1589–1596. doi:10.1128/AEM.02775-08. 19151179 PMC2655475

[20] Geisseler D, Scow KM. 2014. Long-term effects of mineral fertilizers on soil microorganisms: a review. Soil Biol Biochem 75:54–63. doi:10.1016/j.soilbio.2014.03.023.

[21] Zhou J, Jiang X, Wei D, Zhao B, Ma M, Chen S, Cao F, Shen D, Guan D, Li J. 2017. Consistent effects of nitrogen fertilization on soil bacterial communities in black soils for two crop seasons in China. Sci Rep 7:3267. doi:10.1038/s41598-017-03539-6. 28607352 PMC5468298

[22] Nannipieri P, Ascher J, Ceccherini M, Landi L, Pietramellara G, Renella G. 2003. Microbial diversity and soil functions. Eur J Soil Sci 54:655–670. doi:10.1046/j.1351-0754.2003.0556.x.

[23] Burge W, Enkiri N. 1978. Virus adsorption by five soils. Wiley Online Library, Hoboken, NJ.

[24] Zhuang J, Jin Y. 2003. Virus retention and transport as influenced by different forms of soil organic matter. J Environ Qual 32:816–823. doi:10.2134/jeq2003.8160. 12809282

[25] Powelson DK, Simpson JR, Gerba CP. 1991. Effects of organic matter on virus transport in unsaturated flow. Appl Environ Microbiol 57:2192–2196. doi:10.1128/aem.57.8.2192-2196.1991. 1768089 PMC183549

[26] Waldbauer JR, Coleman ML, Rizzo AI, Campbell KL, Lotus J, Zhang L. 2019. Nitrogen sourcing during viral infection of marine cyanobacteria. Proc Natl Acad Sci USA 116:15590–15595. doi:10.1073/pnas.1901856116. 31308237 PMC6681717

[27] Weinbauer MG, Rassoulzadegan F. 2004. Are viruses driving microbial diversification and diversity? Environ Microbiol 6:1–11. doi:10.1046/j.1462-2920.2003.00539.x. 14686936

[28] Mann NH. 2005. The third age of phage. PLoS Biol 3:e182. doi:10.1371/journal.pbio.0030182. 15884981 PMC1110918

[29] ter Horst AM, Santos-Medellín C, Sorensen JW, Zinke LA, Wilson RM, Johnston ER, Trubl GG, Pett-Ridge J, Blazewicz SJ, Hanson PJ. 2020. Minnesota peat viromes reveal terrestrial and aquatic niche partitioning for local and global viral populations. bioRxiv https://www.biorxiv.org/content/10.1101/2020.12.15.422944v1. 10.1186/s40168-021-01156-0 PMC8626947 34836550

[30] Trubl G, Hyman P, Roux S, Abedon ST. 2020. Coming-of-age characterization of soil viruses: a user’s guide to virus isolation, detection within metagenomes, and viromics. Soil Syst 4:23. doi:10.3390/soilsystems4020023.

[31] Emerson JB, Roux S, Brum JR, Bolduc B, Woodcroft BJ, Jang HB, Singleton CM, Solden LM, Naas AE, Boyd JA, Hodgkins SB, Wilson RM, Trubl G, Li C, Frolking S, Pope PB, Wrighton KC, Crill PM, Chanton JP, Saleska SR, Tyson GW, Rich VI, Sullivan MB. 2018. Host-linked soil viral ecology along a permafrost thaw gradient. Nat Microbiol 3:870–880. doi:10.1038/s41564-018-0190-y. 30013236 PMC6786970

[32] Trubl G, Jang HB, Roux S, Emerson JB, Solonenko N, Vik DR, Solden L, Ellenbogen J, Runyon AT, Bolduc B, Woodcroft BJ, Saleska SR, Tyson GW, Wrighton KC, Sullivan MB, Rich VI. 2018. Soil viruses are underexplored players in ecosystem carbon processing. mSystems 3:e00076-18. doi:10.1128/mSystems.00076-18. 30320215 PMC6172770

[33] Allen B, Willner D, Oechel WC, Lipson D. 2010. Top-down control of microbial activity and biomass in an Arctic soil ecosystem. Environ Microbiol 12:642–648. doi:10.1111/j.1462-2920.2009.02104.x. 20002136

[34] Guemes AGC, Youle M, Cantu VA, Felts B, Nulton J, Rohwer F. 2016. Viruses as winners in the game of life. Annu Rev Virol 3:197–214. doi:10.1146/annurev-virology-100114-054952. 27741409

[35] Lauber CL, Ramirez KS, Aanderud Z, Lennon J, Fierer N. 2013. Temporal variability in soil microbial communities across land-use types. ISME J 7:1641–1650. doi:10.1038/ismej.2013.50. 23552625 PMC3721119

[36] Wommack KE, Colwell RR. 2000. Virioplankton: viruses in aquatic ecosystems. Microbiol Mol Biol Rev 64:69–114. doi:10.1128/MMBR.64.1.69-114.2000. 10704475 PMC98987

[37] Adriaenssens EM, Van Zyl L, De Maayer P, Rubagotti E, Rybicki E, Tuffin M, Cowan DA. 2015. Metagenomic analysis of the viral community in Namib Desert hypoliths. Environ Microbiol 17:480–495. doi:10.1111/1462-2920.12528. 24912085

[38] Marsh P, Wellington E. 1994. Phage-host interactions in soil. FEMS Microbiol Ecol 15:99–107. doi:10.1111/j.1574-6941.1994.tb00234.x.

[39] Williamson KE, Radosevich M, Smith DW, Wommack KE. 2007. Incidence of lysogeny within temperate and extreme soil environments. Environ Microbiol 9:2563–2574. doi:10.1111/j.1462-2920.2007.01374.x. 17803780

[40] Fornelos N, Browning DF, Pavlin A, Podlesek Z, Hodnik V, Salas M, Butala M. 2018. Lytic gene expression in the temperate bacteriophage GIL01 is activated by a phage-encoded LexA homologue. Nucleic Acids Res 46:9432–9443. doi:10.1093/nar/gky646. 30053203 PMC6182141

[41] Wang Q, Guan Z, Pei K, Wang J, Liu Z, Yin P, Peng D, Zou T. 2018. Structural basis of the arbitrium peptide-AimR communication system in the phage lysis-lysogeny decision. Nat Microbiol 3:1266–1273. doi:10.1038/s41564-018-0239-y. 30224798

[42] You L, Suthers PF, Yin J. 2002. Effects of *Escherichia coli* physiology on growth of phage T7 *in vivo* and *in silico*. J Bacteriol 184:1888–1894. doi:10.1128/JB.184.7.1888-1894.2002. 11889095 PMC134924

[43] Sime-Ngando T. 2014. Environmental bacteriophages: viruses of microbes in aquatic ecosystems. Front Microbiol 5:355. doi:10.3389/fmicb.2014.00355. 25104950 PMC4109441

[44] Howard-Varona C, Hargreaves KR, Solonenko NE, Markillie LM, White RA, Brewer HM, Ansong C, Orr G, Adkins JN, Sullivan MB. 2018. Multiple mechanisms drive phage infection efficiency in nearly identical hosts. ISME J 12:1605–1618. doi:10.1038/s41396-018-0099-8. 29568113 PMC5955906

[45] Wang I-N. 2006. Lysis timing and bacteriophage fitness. Genetics 172:17–26. doi:10.1534/genetics.105.045922. 16219778 PMC1456144

[46] Gazitúa MC, Vik DR, Roux S, Gregory AC, Bolduc B, Widner B, Mulholland MR, Hallam SJ, Ulloa O, Sullivan MB. 2021. Potential virus-mediated nitrogen cycling in oxygen-depleted oceanic waters. ISME J 15:981–998. doi:10.1038/s41396-020-00825-6. 33199808 PMC8115048

[47] Alteio LV, Schulz F, Seshadri R, Varghese N, Rodriguez-Reillo W, Ryan E, Goudeau D, Eichorst SA, Malmstrom RR, Bowers RM, Katz LA, Blanchard JL, Woyke T. 2020. Complementary metagenomic approaches improve reconstruction of microbial diversity in a forest soil. mSystems 5:e00768-19. doi:10.1128/mSystems.00768-19. 32156798 PMC7065516

[48] Trubl G, Kimbrel JA, Liquet-Gonzalez J, Nuccio EE, Weber PK, Pett-Ridge J, Jansson JK, Waldrop MP, Blazewicz SJ. 2021. Active virus-host interactions at sub-freezing temperatures in Arctic peat soil. Microbiome 9:1–15. doi:10.1186/s40168-021-01154-2. 34663463 PMC8522061

[49] Paez-Espino D, Eloe-Fadrosh EA, Pavlopoulos GA, Thomas AD, Huntemann M, Mikhailova N, Rubin E, Ivanova NN, Kyrpides NC. 2016. Uncovering Earth’s virome. Nature 536:425–430. doi:10.1038/nature19094. 27533034

[50] Roux S, Hallam SJ, Woyke T, Sullivan MB. 2015. Viral dark matter and virus–host interactions resolved from publicly available microbial genomes. Elife 4:e08490. doi:10.7554/eLife.08490. 26200428 PMC4533152

[51] Duan N, Li L, Liang X, McDearis R, Fine AK, Cheng Z, Zhuang J, Radosevich M, Schaeffer SM. 2022. Composition of soil viral and bacterial communities after long-term tillage, fertilization, and cover cropping management. Appl Soil Ecol 177:104510. doi:10.1016/j.apsoil.2022.104510.

[52] Armanious A, Aeppli M, Jacak R, Refardt D, Sigstam T, Kohn T, Sander M. 2016. Viruses at solid–water interfaces: a systematic assessment of interactions driving adsorption. Environ Sci Technol 50:732–743. doi:10.1021/acs.est.5b04644. 26636722

[53] Hurst CJ, Gerba CP, Cech I. 1980. Effects of environmental variables and soil characteristics on virus survival in soil. Appl Environ Microbiol 40:1067–1079. doi:10.1128/aem.40.6.1067-1079.1980. 6257161 PMC291723

[54] Chen L, Xun W, Sun L, Zhang N, Shen Q, Zhang R. 2014. Effect of different long-term fertilization regimes on the viral community in an agricultural soil of southern China. Eur J Soil Biol 62:121–126. doi:10.1016/j.ejsobi.2014.03.006.

[55] Duan N, Li L, Liang X, McDearis R, Fine AK, Cheng Z, Zhuang J, Radosevich M, Schaeffer SM. 2022. Composition of soil viral and bacterial communities after long-term tillage, fertilization, and cover cropping management. Appl Soil Ecol 177. 10.1016/j.apsoil.2022.104510 doi:10.1016/j.apsoil.2022.104510.

[56] Duan N, Li L, Liang X, Fine A, Zhuang J, Radosevich M, Schaeffer SM. 2022. Variation in bacterial community structure under long-term fertilization, tillage, and cover cropping in continuous cotton production. Front Microbiol 13:847005. doi:10.3389/fmicb.2022.847005. 35444635 PMC9015707

[57] Ivanova AA, Zhelezova AD, Chernov TI, Dedysh SN. 2020. Linking ecology and systematics of acidobacteria: distinct habitat preferences of the Acidobacteriia and Blastocatellia in tundra soils. PLoS One 15:e0230157. doi:10.1371/journal.pone.0230157. 32182280 PMC7077872

[58] Nicholson WL, Munakata N, Horneck G, Melosh HJ, Setlow P. 2000. Resistance of Bacillus endospores to extreme terrestrial and extraterrestrial environments. Microbiol Mol Biol Rev 64:548–572. doi:10.1128/MMBR.64.3.548-572.2000. 10974126 PMC99004

[59] Gabiatti N, Yu P, Mathieu J, Lu GW, Wang X, Zhang H, Soares HM, Alvarez PJ. 2018. Bacterial endospores as phage genome carriers and protective shells. Appl Environ Microbiol 84. doi:10.1128/AEM.01186-18. PMC6121981 30006404

[60] Abedon ST. 2009. Disambiguating bacteriophage pseudolysogeny: an historical analysis of lysogeny, pseudolysogeny, and the phage carrier state, p 285–307. *In* Adams HT (ed), Contemporary trends in bacteriophage research. Nova Science Publishers, Hauppauge, NY.

[61] Van Goethem MW, Swenson TL, Trubl G, Roux S, Northen TR. 2019. Characteristics of wetting-induced bacteriophage blooms in biological soil crust. mBio 10:e02287-19. doi:10.1128/mBio.02287-19. 31848272 PMC6918073

[62] Abedon ST. 2011. Communication among phages, bacteria, and soil environments, p 37–65. *In* Biocommunication in soil microorganisms. Springer, New York, NY.

[63] Köberl M, Wagner P, Müller H, Matzer R, Unterfrauner H, Cernava T, Berg G. 2020. Unraveling the complexity of soil microbiomes in a large-scale study subjected to different agricultural management in Styria. Front Microbiol 11:1052. doi:10.3389/fmicb.2020.01052. 32523580 PMC7261914

[64] Tourna M, Stieglmeier M, Spang A, Könneke M, Schintlmeister A, Urich T, Engel M, Schloter M, Wagner M, Richter A. 2011. *Nitrososphaera viennensis*, an ammonia oxidizing archaeon from soil. Proc Natl Acad Sci USA 108:8420–8425. doi:10.1073/pnas.1013488108. 21525411 PMC3100973

[65] Spang A, Hatzenpichler R, Brochier-Armanet C, Rattei T, Tischler P, Spieck E, Streit W, Stahl DA, Wagner M, Schleper C. 2010. Distinct gene set in two different lineages of ammonia-oxidizing archaea supports the phylum *Thaumarchaeota*. Trends Microbiol 18:331–340. doi:10.1016/j.tim.2010.06.003. 20598889

[66] Han J, Shi J, Zeng L, Xu J, Wu L. 2017. Impacts of continuous excessive fertilization on soil potential nitrification activity and nitrifying microbial community dynamics in greenhouse system. J Soils Sediments 17:471–480. doi:10.1007/s11368-016-1525-z.

[67] Di HJ, Cameron KC, Shen J, Winefield CS, O’Callaghan M, Bowatte S, He J. 2010. Ammonia-oxidizing bacteria and archaea grow under contrasting soil nitrogen conditions. FEMS Microbiol Ecol 72:386–394. doi:10.1111/j.1574-6941.2010.00861.x. 20370827

[68] Shen J, Zhang L, He J. 2014. Contrasting response of nitrification capacity in three agricultural soils to N addition during short-term incubation. J Soils Sediments 14:1861–1868. doi:10.1007/s11368-014-0968-3.

[69] Santos-Medellin C, Zinke LA, Ter Horst AM, Gelardi DL, Parikh SJ, Emerson JB. 2021. Viromes outperform total metagenomes in revealing the spatiotemporal patterns of agricultural soil viral communities. ISME J 15:1956–1970. doi:10.1038/s41396-021-00897-y. 33612831 PMC8245658

[70] Nicolas AM, Jaffe AL, Nuccio EE, Taga ME, Firestone MK, Banfield JF. 2021. Soil candidate phyla radiation bacteria encode components of aerobic metabolism and co-occur with nanoarchaea in the rare biosphere of rhizosphere grassland communities. mSystems 6:e01205-20. doi:10.1128/mSystems.01205-20. 34402646 PMC8407418

[71] Emerson JB, Thomas BC, Andrade K, Allen EE, Heidelberg KB, Banfield JF. 2012. Dynamic viral populations in hypersaline systems as revealed by metagenomic assembly. Appl Environ Microbiol 78:6309–6320. doi:10.1128/AEM.01212-12. 22773627 PMC3416638

[72] Schmidt HF, Sakowski EG, Williamson SJ, Polson SW, Wommack K. 2014. Shotgun metagenomics indicates novel family A DNA polymerases predominate within marine virioplankton. ISME J 8:103–114. doi:10.1038/ismej.2013.124. 23985748 PMC3869006

[73] McNair K, Bailey BA, Edwards RA. 2012. PHACTS, a computational approach to classifying the lifestyle of phages. Bioinformatics 28:614–618. doi:10.1093/bioinformatics/bts014. 22238260 PMC3289917

[74] Wu S, Fang Z, Tan J, Li M, Wang C, Guo Q, Xu C, Jiang X, Zhu H. 2021. DeePhage: distinguishing virulent and temperate phage-derived sequences in metavirome data with a deep learning approach. Gigascience 10:giab056. doi:10.1093/gigascience/giab056. 34498685 PMC8427542

[75] Doane TA, Horwáth WR. 2003. Spectrophotometric determination of nitrate with a single reagent. Anal Lett 36:2713–2722. doi:10.1081/AL-120024647.

[76] Rhine ED, Mulvaney RL, Pratt EJ, Sims GK. 1998. Improving the Berthelot reaction for determining ammonium in soil extracts and water. Soil Sci Soc Am J 62:473–480. doi:10.2136/sssaj1998.03615995006200020026x.

[77] Culman S, Freeman M, Snapp S. 2012. Procedure for the determination of permanganate oxidizable carbon. Kellogg Biological Station, Hickory Corners, MI.

[78] Weil RR, Islam KR, Stine MA, Gruver JB, Samson-Liebig SE. 2003. Estimating active carbon for soil quality assessment: a simplified method for laboratory and field use. Am J Alternative Agric 18:3–17. doi:10.1079/AJAA2003003.

[79] Martin M. 2011. Cutadapt removes adapter sequences from high-throughput sequencing reads. EMBnet j 17:10–12. doi:10.14806/ej.17.1.200.

[80] Li D, Liu C-M, Luo R, Sadakane K, Lam T-W. 2015. MEGAHIT: an ultra-fast single-node solution for large and complex metagenomics assembly via succinct de Bruijn graph. Bioinformatics 31:1674–1676. doi:10.1093/bioinformatics/btv033. 25609793

[81] Li D, Luo R, Liu C-M, Leung C-M, Ting H-F, Sadakane K, Yamashita H, Lam T-W. 2016. MEGAHIT v1.0: a fast and scalable metagenome assembler driven by advanced methodologies and community practices. Methods 102:3–11. doi:10.1016/j.ymeth.2016.02.020. 27012178

[82] Mikheenko A, Prjibelski A, Saveliev V, Antipov D, Gurevich A. 2018. Versatile genome assembly evaluation with QUAST-LG. Bioinformatics 34:i142–i150. doi:10.1093/bioinformatics/bty266. 29949969 PMC6022658

[83] Li W, Godzik A. 2006. Cd-hit: a fast program for clustering and comparing large sets of protein or nucleotide sequences. Bioinformatics 22:1658–1659. doi:10.1093/bioinformatics/btl158. 16731699

[84] Evans JT, Denef VJ. 2020. To dereplicate or not to dereplicate? mSphere 5:e00971-19. doi:10.1128/mSphere.00971-19. 32434845 PMC7380574

[85] Li W, Jaroszewski L, Godzik A. 2002. Tolerating some redundancy significantly speeds up clustering of large protein databases. Bioinformatics 18:77–82. doi:10.1093/bioinformatics/18.1.77. 11836214

[86] Roux S, Enault F, Hurwitz BL, Sullivan MB. 2015. VirSorter: mining viral signal from microbial genomic data. PeerJ 3:e985. doi:10.7717/peerj.985. 26038737 PMC4451026

[87] Ren J, Song K, Deng C, Ahlgren N, Fuhrman J, Li Y, Xie X, Sun F. 2018. Identifying viruses from metagenomic data by deep learning. arXiv preprint arXiv:180607810. doi:10.29007/f7jx. PMC8172088 34084563

[88] Kieft K, Zhou Z, Anantharaman K. 2020. VIBRANT: automated recovery, annotation and curation of microbial viruses, and evaluation of viral community function from genomic sequences. Microbiome 8:1–23. doi:10.1186/s40168-020-00867-0. 32522236 PMC7288430

[89] Guo J, Bolduc B, Zayed AA, Varsani A, Dominguez-Huerta G, Delmont TO, Pratama AA, Gazitúa MC, Vik D, Sullivan MB, Roux S. 2021. VirSorter2: a multi-classifier, expert-guided approach to detect diverse DNA and RNA viruses. Microbiome 9:1–13. doi:10.1186/s40168-020-00990-y. 33522966 PMC7852108

[90] Roux S, Adriaenssens EM, Dutilh BE, Koonin EV, Kropinski AM, Krupovic M, Kuhn JH, Lavigne R, Brister JR, Varsani A, Amid C, Aziz RK, Bordenstein SR, Bork P, Breitbart M, Cochrane GR, Daly RA, Desnues C, Duhaime MB, Emerson JB, Enault F, Fuhrman JA, Hingamp P, et al. 2019. Minimum information about an uncultivated virus genome (MIUViG). Nat Biotechnol 37:29–37. doi:10.1038/nbt.4306. 30556814 PMC6871006

[91] Bushnell B. 2015. BBMap short-read aligner, and other bioinformatics tools. University of California, Berkeley, CA.

[92] Li H, Handsaker B, Wysoker A, Fennell T, Ruan J, Homer N, Marth G, Abecasis G, Durbin R, 1000 Genome Project Data Processing Subgroup. 2009. The sequence alignment/map format and SAMtools. Bioinformatics 25:2078–2079. doi:10.1093/bioinformatics/btp352. 19505943 PMC2723002

[93] Quinlan AR, Hall IM. 2010. BEDTools: a flexible suite of utilities for comparing genomic features. Bioinformatics 26:841–842. doi:10.1093/bioinformatics/btq033. 20110278 PMC2832824

[94] Zablocki O, Jang HB, Bolduc B, Sullivan MB. 2019. ConTACT v2: a tool to automate genome-based prokaryotic viral taxonomy. *In* Plant and Animal Genome XXVII Conference (12 to 16 January 2019). Plant and Animal Genome XXVII Conference, San Diego, CA.

[95] Hyatt D, LoCascio PF, Hauser LJ, Uberbacher EC. 2012. Gene and translation initiation site prediction in metagenomic sequences. Bioinformatics 28:2223–2230. doi:10.1093/bioinformatics/bts429. 22796954

[96] Bolduc B, Jang HB, Doulcier G, You Z-Q, Roux S, Sullivan MB. 2017. vConTACT: an iVirus tool to classify double-stranded DNA viruses that infect *Archaea* and *Bacteria*. PeerJ 5:e3243. doi:10.7717/peerj.3243. 28480138 PMC5419219

[97] O’Leary NA, Wright MW, Brister JR, Ciufo S, Haddad D, McVeigh R, Rajput B, Robbertse B, Smith-White B, Ako-Adjei D, Astashyn A, Badretdin A, Bao Y, Blinkova O, Brover V, Chetvernin V, Choi J, Cox E, Ermolaeva O, Farrell CM, Goldfarb T, Gupta T, Haft D, Hatcher E, et al. 2016. Reference sequence (RefSeq) database at NCBI: current status, taxonomic expansion, and functional annotation. Nucleic Acids Res 44:D733–D745. doi:10.1093/nar/gkv1189. 26553804 PMC4702849

[98] Kang DD, Li F, Kirton E, Thomas A, Egan R, An H, Wang Z. 2019. MetaBAT 2: an adaptive binning algorithm for robust and efficient genome reconstruction from metagenome assemblies. PeerJ 7:e7359. doi:10.7717/peerj.7359. 31388474 PMC6662567

[99] Wu Y-W, Tang Y-H, Tringe SG, Simmons BA, Singer SW. 2014. MaxBin: an automated binning method to recover individual genomes from metagenomes using an expectation-maximization algorithm. Microbiome 2:26–18. doi:10.1186/2049-2618-2-26. 25136443 PMC4129434

[100] Wu Y-W, Simmons BA, Singer SW. 2016. MaxBin 2.0: an automated binning algorithm to recover genomes from multiple metagenomic datasets. Bioinformatics 32:605–607. doi:10.1093/bioinformatics/btv638. 26515820

[101] Alneberg J, Bjarnason BS, de Bruijn I, Schirmer M, Quick J, Ijaz UZ, Loman NJ, Andersson AF, Quince C. 2013. CONCOCT: clustering contigs on coverage and composition. arXiv preprint arXiv:1312.4038. 10.1038/nmeth.3103 25218180

[102] Uritskiy GV, DiRuggiero J, Taylor J. 2018. MetaWRAP: a flexible pipeline for genome-resolved metagenomic data analysis. Microbiome 6:1–13. doi:10.1186/s40168-018-0541-1. 30219103 PMC6138922

[103] Parks DH, Imelfort M, Skennerton CT, Hugenholtz P, Tyson GW. 2015. CheckM: assessing the quality of microbial genomes recovered from isolates, single cells, and metagenomes. Genome Res 25:1043–1055. doi:10.1101/gr.186072.114. 25977477 PMC4484387

[104] Parks DH, Rinke C, Chuvochina M, Chaumeil P-A, Woodcroft BJ, Evans PN, Hugenholtz P, Tyson GW. 2017. Recovery of nearly 8,000 metagenome-assembled genomes substantially expands the tree of life. Nat Microbiol 2:1533–1542. doi:10.1038/s41564-017-0012-7. 28894102

[105] Olm MR, Brown CT, Brooks B, Banfield JF. 2017. dRep: a tool for fast and accurate genomic comparisons that enables improved genome recovery from metagenomes through de-replication. ISME J 11:2864–2868. doi:10.1038/ismej.2017.126. 28742071 PMC5702732

[106] Chaumeil P-A, Mussig AJ, Hugenholtz P, Parks DH. 2020. GTDB-Tk: a toolkit to classify genomes with the Genome Taxonomy Database. Oxford University Press, Oxford, United Kingdom. 10.1093/bioinformatics/btz848 PMC7703759 31730192

[107] Le SQ, Gascuel O. 2008. An improved general amino acid replacement matrix. Mol Biol Evol 25:1307–1320. doi:10.1093/molbev/msn067. 18367465

[108] Kumar S, Stecher G, Li M, Knyaz C, Tamura K. 2018. MEGA X: molecular evolutionary genetics analysis across computing platforms. Mol Biol Evol 35:1547–1549. doi:10.1093/molbev/msy096. 29722887 PMC5967553

[109] Stecher G, Tamura K, Kumar S. 2020. Molecular evolutionary genetics analysis (MEGA) for macOS. Mol Biol Evol 37:1237–1239. doi:10.1093/molbev/msz312. 31904846 PMC7086165

[110] Skennerton CT, Imelfort M, Tyson GW. 2013. Crass: identification and reconstruction of CRISPR from unassembled metagenomic data. Nucleic Acids Res 41:e105. doi:10.1093/nar/gkt183. 23511966 PMC3664793

[111] McGinnis S, Madden TL. 2004. BLAST: at the core of a powerful and diverse set of sequence analysis tools. Nucleic Acids Res 32:W20–W25. doi:10.1093/nar/gkh435. 15215342 PMC441573

[112] Jain C, Rodriguez-R LM, Phillippy AM, Konstantinidis KT, Aluru S. 2018. High throughput ANI analysis of 90K prokaryotic genomes reveals clear species boundaries. Nat Commun 9:1–8. doi:10.1038/s41467-018-07641-9. 30504855 PMC6269478

[113] Roux S, Brum JR, Dutilh BE, Sunagawa S, Duhaime MB, Loy A, Poulos BT, Solonenko N, Lara E, Poulain J, Pesant S, Kandels-Lewis S, Dimier C, Picheral M, Searson S, Cruaud C, Alberti A, Duarte CM, Gasol JM, Vaqué D, Bork P, Acinas SG, Wincker P, Sullivan MB, Tara Oceans Coordinators. 2016. Ecogenomics and potential biogeochemical impacts of globally abundant ocean viruses. Nature 537:689–693. doi:10.1038/nature19366. 27654921

[114] Shapiro SS, Francia R. 1972. An approximate analysis of variance test for normality. J Am Stat Assoc 67:215–216. doi:10.1080/01621459.1972.10481232.

[115] Shapiro SS, Wilk MB, Chen HJ. 1968. A comparative study of various tests for normality. J Am Stat Assoc 63:1343–1372. doi:10.1080/01621459.1968.10480932.

[116] Abdi H, Williams LJ. 2010. Tukey’s honestly significant difference (HSD) test. Encyclopedia Res Design 3:1–5.

