# Supplementary material for: Identification of Novel Viruses and Their Microbial Hosts from Soils with Long-Term Nitrogen Fertilization and Cover Cropping Management
Source: mSystems. 2022 Nov 29;7(6):e00571-22. doi: 10.1128/msystems.00571-22 (PMC9765229; doi:10.1128/msystems.00571-22)
Supplement: TABLE S1 [file msystems.00571-22-s0001.docx]

Table S1

| Viral contigs | vOTU_ID | Length (bp) | vOTU_Lysogeny | Evidence |
| --- | --- | --- | --- | --- |
| gary_all20_3439 | vOTU_1 | 47,194 |  |  |
| gary_all20_6694 | vOTU_2 | 36,236 |  |  |
| gary_all20_7195 | vOTU_3 | 34,477 | vOTU_3 | bacterial prophage |
| SPRUCE_viral_seq_1218 | vOTU_4 | 30,104 |  |  |
| gary_all20_10349 | vOTU_5 | 24,300 |  |  |
| gary_all20_11343 | vOTU_6 | 22,251 |  |  |
| gary_all20_11399 | vOTU_7 | 22,151 |  |  |
| gary_all20_11916 | vOTU_8 | 21,202 |  |  |
| virsorter_curated_4650 | vOTU_9 | 20,110 |  |  |
| gary_all20_12995 | vOTU_10 | 19,369 |  |  |
| alaska_puertorico_377 | vOTU_11 | 17,399 |  |  |
| alaska_puertorico_431 | vOTU_12 | 15,839 |  |  |
| SPRUCE_viral_seq_2687 | vOTU_13 | 15,800 |  |  |
| gary_all20_16094 | vOTU_14 | 15,536 | vOTU_14 | bacterial prophage |
| alaska_puertorico_612 | vOTU_15 | 13,099 |  |  |
| gary_all20_20258 | vOTU_16 | 11,920 |  |  |
| gary_all20_20798 | vOTU_17 | 11,566 |  |  |
| EarthsVirome_48737 | vOTU_18 | 11,295 |  |  |
| alaska_puertorico_778 | vOTU_19 | 11,220 | vOTU_19 | bacterial prophage |
| alaska_puertorico_785 | vOTU_20 | 11,180 |  |  |
| alaska_puertorico_799 | vOTU_21 | 11,053 |  |  |
| alaska_puertorico_801 | vOTU_22 | 11,038 |  |  |
| gary_all20_21729 | vOTU_23 | 10,971 |  |  |
| alaska_puertorico_880 | vOTU_24 | 10,526 |  |  |
| alaska_puertorico_938 | vOTU_25 | 10,220 |  |  |
| gary_all20_23107 | vOTU_26 | 10,195 |  |  |
| gary_all20_23251 | vOTU_27 | 10,112 |  |  |
| alaska_puertorico_961 | vOTU_28 | 10,087 |  |  |
| NIFA_virome_16612 | vOTU_29 | 270,217 | vOTU_29 | recombinase |
| NIFA_virome_61188 | vOTU_30 | 147,364 |  |  |
| NIFA_virome_61321 | vOTU_31 | 124,975 | vOTU_31 | recombinase |
| NIFA_virome_60872 | vOTU_32 | 78,569 |  |  |
| NIFA_virome_63000 | vOTU_33 | 69,899 |  |  |
| NIFA_virome_61423 | vOTU_34 | 67,825 |  |  |
| NIFA_virome_61978 | vOTU_35 | 59,679 |  |  |
| NIFA_virome_62409 | vOTU_36 | 58,781 |  |  |
| NIFA_virome_62993 | vOTU_37 | 58,314 |  |  |
| NIFA_virome_62106 | vOTU_38 | 56,634 |  |  |
| NIFA_virome_63901 | vOTU_39 | 53,867 | vOTU_39 | recombinase |
| NIFA_virome_63029 | vOTU_40 | 51,867 |  |  |
| NIFA_virome_64073 | vOTU_41 | 51,796 |  |  |
| NIFA_virome_61585 | vOTU_42 | 49,829 |  |  |
| NIFA_virome_63128 | vOTU_43 | 49,468 |  |  |
| NIFA_virome_62399 | vOTU_44 | 49,468 |  |  |
| NIFA_virome_64080 | vOTU_45 | 49,178 |  |  |
| NIFA_virome_63920 | vOTU_46 | 47,770 | vOTU_46 | recombinase |
| NIFA_virome_61295 | vOTU_47 | 46,971 |  |  |
| NIFA_virome_61730 | vOTU_48 | 45,930 | vOTU_48 | bacterial prophage |
| NIFA_virome_62276 | vOTU_49 | 45,538 | vOTU_49 | recombinase |
| NIFA_virome_63164 | vOTU_50 | 45,418 |  |  |
| NIFA_virome_51733 | vOTU_51 | 45,401 |  |  |
| NIFA_virome_61533 | vOTU_52 | 44,849 | vOTU_52 | recombinase |
| NIFA_virome_63105 | vOTU_53 | 44,161 | vOTU_53 | recombinase |
| NIFA_virome_62311 | vOTU_54 | 44,098 |  |  |
| NIFA_virome_61802 | vOTU_55 | 43,710 |  |  |
| NIFA_virome_52668 | vOTU_56 | 43,257 | vOTU_56 | Archaeal prophage |
| NIFA_virome_62297 | vOTU_57 | 43,088 |  |  |
| NIFA_virome_62967 | vOTU_58 | 42,913 | vOTU_58 | recombinase |
| NIFA_virome_60875 | vOTU_59 | 42,826 |  |  |
| NIFA_virome_61678 | vOTU_60 | 42,680 | vOTU_60 | recombinase |
| NIFA_virome_7102 | vOTU_61 | 41,431 |  |  |
| NIFA_virome_60751 | vOTU_62 | 41,319 |  |  |
| NIFA_virome_61313 | vOTU_63 | 40,037 | vOTU_63 | recombinase & bacterial prophage |
| NIFA_virome_7290 | vOTU_64 | 39,928 |  |  |
| NIFA_virome_62477 | vOTU_65 | 39,772 |  |  |
| NIFA_virome_61876 | vOTU_66 | 39,168 |  |  |
| NIFA_virome_63121 | vOTU_67 | 38,036 |  |  |
| NIFA_virome_61061 | vOTU_68 | 37,413 |  |  |
| NIFA_virome_62964 | vOTU_69 | 36,598 |  |  |
| NIFA_virome_4537 | vOTU_70 | 34,618 |  |  |
| NIFA_virome_8979 | vOTU_71 | 33,593 |  |  |
| NIFA_virome_64136 | vOTU_72 | 31,335 |  |  |
| NIFA_virome_57183 | vOTU_73 | 31,157 | vOTU_74 | recombinase |
| NIFA_virome_63967 | vOTU_74 | 29,682 |  |  |
| NIFA_virome_60794 | vOTU_75 | 28,631 |  |  |
| NIFA_virome_63382 | vOTU_76 | 27,640 |  |  |
| NIFA_virome_7105 | vOTU_77 | 27,201 |  |  |
| NIFA_virome_60702 | vOTU_78 | 26,849 |  |  |
| NIFA_virome_62374 | vOTU_79 | 26,198 |  |  |
| NIFA_virome_62188 | vOTU_80 | 26,109 | vOTU_80 | recombinase |
| NIFA_virome_30030 | vOTU_81 | 25,318 | vOTU_81 | bacterial prophage |
| NIFA_virome_61242 | vOTU_82 | 25,314 |  |  |
| NIFA_virome_64186 | vOTU_83 | 24,609 |  |  |
| NIFA_virome_62801 | vOTU_84 | 23,560 |  |  |
| NIFA_virome_63948 | vOTU_85 | 23,282 |  |  |
| NIFA_virome_63235 | vOTU_86 | 23,188 |  |  |
| NIFA_virome_62184 | vOTU_87 | 23,119 | vOTU_87 | recombinase |
| NIFA_virome_62756 | vOTU_88 | 23,117 |  |  |
| NIFA_virome_63939 | vOTU_89 | 22,704 |  |  |
| NIFA_virome_20851 | vOTU_90 | 22,302 |  |  |
| NIFA_virome_61294 | vOTU_91 | 22,164 | vOTU_91 | recombinase |
| NIFA_virome_62084 | vOTU_92 | 21,968 |  |  |
| NIFA_virome_10698 | vOTU_93 | 21,680 | vOTU_93 | recombinase & bacterial prophage |
| NIFA_virome_60804 | vOTU_94 | 21,550 |  |  |
| NIFA_virome_61618 | vOTU_95 | 20,524 |  |  |
| NIFA_virome_63221 | vOTU_96 | 19,932 |  |  |
| NIFA_virome_60699 | vOTU_97 | 19,891 |  |  |
| NIFA_virome_63102 | vOTU_98 | 19,434 |  |  |
| NIFA_virome_7731 | vOTU_99 | 19,288 |  |  |
| NIFA_virome_61872 | vOTU_100 | 19,077 |  |  |
| NIFA_virome_60823 | vOTU_101 | 19,049 |  |  |
| NIFA_virome_62249 | vOTU_102 | 18,999 |  |  |
| NIFA_virome_64179 | vOTU_103 | 18,997 | vOTU_103 | recombinase |
| NIFA_virome_64092 | vOTU_104 | 18,697 |  |  |
| NIFA_virome_57519 | vOTU_105 | 18,657 | vOTU_106 | bacterial prophage |
| NIFA_virome_16493 | vOTU_106 | 18,622 |  |  |
| NIFA_virome_27311 | vOTU_107 | 18,579 |  |  |
| NIFA_virome_61229 | vOTU_108 | 18,462 |  |  |
| NIFA_virome_62949 | vOTU_109 | 18,431 |  |  |
| NIFA_virome_61517 | vOTU_110 | 18,315 |  |  |
| NIFA_virome_62866 | vOTU_111 | 18,202 |  |  |
| NIFA_virome_61686 | vOTU_112 | 18,060 |  |  |
| NIFA_virome_61322 | vOTU_113 | 17,966 |  |  |
| NIFA_virome_60924 | vOTU_114 | 17,895 | vOTU_115 | bacterial prophage |
| NIFA_virome_23088 | vOTU_115 | 17,889 | vOTU_116 | recombinase |
| NIFA_virome_61428 | vOTU_116 | 17,869 |  |  |
| NIFA_virome_60249 | vOTU_117 | 17,549 |  |  |
| NIFA_virome_62709 | vOTU_118 | 17,533 |  |  |
| NIFA_virome_63951 | vOTU_119 | 17,521 |  |  |
| NIFA_virome_43994 | vOTU_120 | 17,373 | vOTU_120 | Archaeal prophage |
| NIFA_virome_62163 | vOTU_121 | 17,222 |  |  |
| NIFA_virome_62887 | vOTU_122 | 17,146 |  |  |
| NIFA_virome_61451 | vOTU_123 | 17,132 |  |  |
| NIFA_virome_60918 | vOTU_124 | 17,102 |  |  |
| NIFA_virome_16073 | vOTU_125 | 17,003 |  |  |
| NIFA_virome_61818 | vOTU_126 | 16,705 |  |  |
| NIFA_virome_18499 | vOTU_127 | 16,670 | vOTU_128 | Archaeal prophage |
| NIFA_virome_62612 | vOTU_128 | 16,655 | vOTU_129 | Archaeal prophage |
| NIFA_virome_1434 | vOTU_129 | 16,636 | vOTU_130 | Archaeal prophage |
| NIFA_virome_12105 | vOTU_130 | 16,622 |  |  |
| NIFA_virome_60613 | vOTU_131 | 16,620 |  |  |
| NIFA_virome_63080 | vOTU_132 | 16,587 |  |  |
| NIFA_virome_59209 | vOTU_133 | 16,573 |  |  |
| NIFA_virome_10923 | vOTU_134 | 16,565 |  |  |
| NIFA_virome_61933 | vOTU_135 | 16,496 | vOTU_135 | recombinase |
| NIFA_virome_60628 | vOTU_136 | 16,483 |  |  |
| NIFA_virome_1141 | vOTU_137 | 16,352 | vOTU_137 | Archaeal prophage |
| NIFA_virome_53182 | vOTU_138 | 16,229 | vOTU_138 | Archaeal prophage |
| NIFA_virome_63924 | vOTU_139 | 16,204 |  |  |
| NIFA_virome_9514 | vOTU_140 | 16,165 | vOTU_141 | recombinase |
| NIFA_virome_63067 | vOTU_141 | 16,128 | vOTU_142 | Archaeal prophage |
| NIFA_virome_31962 | vOTU_142 | 16,040 |  |  |
| NIFA_virome_64183 | vOTU_143 | 15,966 |  |  |
| NIFA_virome_62642 | vOTU_144 | 15,937 |  |  |
| NIFA_virome_64091 | vOTU_145 | 15,927 | vOTU_145 | recombinase |
| NIFA_virome_63095 | vOTU_146 | 15,924 |  |  |
| NIFA_virome_62116 | vOTU_147 | 15,879 |  |  |
| NIFA_virome_15867 | vOTU_148 | 15,844 | vOTU_148 | bacterial prophage |
| NIFA_virome_61249 | vOTU_149 | 15,735 |  |  |
| NIFA_virome_60749 | vOTU_150 | 15,423 |  |  |
| NIFA_virome_9523 | vOTU_151 | 15,388 |  |  |
| NIFA_virome_61456 | vOTU_152 | 15,341 | vOTU_152 | bacterial prophage |
| NIFA_virome_17346 | vOTU_153 | 14,891 | vOTU_153 | bacterial prophage |
| NIFA_virome_61094 | vOTU_154 | 14,825 |  |  |
| NIFA_virome_47580 | vOTU_155 | 14,681 |  |  |
| NIFA_virome_62056 | vOTU_156 | 14,508 |  |  |
| NIFA_virome_60886 | vOTU_157 | 14,478 |  |  |
| NIFA_virome_63188 | vOTU_158 | 14,417 |  |  |
| NIFA_virome_48106 | vOTU_159 | 14,385 | vOTU_159 | Archaeal prophage |
| NIFA_virome_63944 | vOTU_160 | 14,303 |  |  |
| NIFA_virome_357 | vOTU_161 | 14,281 | vOTU_161 | bacterial prophage |
| NIFA_virome_60831 | vOTU_162 | 14,267 |  |  |
| NIFA_virome_47644 | vOTU_163 | 14,147 | vOTU_163 | bacterial prophage |
| NIFA_virome_29885 | vOTU_164 | 14,060 |  |  |
| NIFA_virome_62575 | vOTU_165 | 14,018 | vOTU_166 | Archaeal prophage |
| NIFA_virome_63897 | vOTU_166 | 13,948 |  |  |
| NIFA_virome_63070 | vOTU_167 | 13,779 |  |  |
| NIFA_virome_64122 | vOTU_168 | 13,578 |  |  |
| NIFA_virome_62437 | vOTU_169 | 13,520 |  |  |
| NIFA_virome_61860 | vOTU_170 | 13,469 |  |  |
| NIFA_virome_8381 | vOTU_171 | 13,351 |  |  |
| NIFA_virome_61905 | vOTU_172 | 13,247 |  |  |
| NIFA_virome_60798 | vOTU_173 | 13,207 |  |  |
| NIFA_virome_6490 | vOTU_174 | 13,132 |  |  |
| NIFA_virome_61844 | vOTU_175 | 13,130 |  |  |
| NIFA_virome_61900 | vOTU_176 | 13,053 |  |  |
| NIFA_virome_62075 | vOTU_177 | 12,807 |  |  |
| NIFA_virome_63236 | vOTU_178 | 12,776 |  |  |
| NIFA_virome_62508 | vOTU_179 | 12,744 |  |  |
| NIFA_virome_61652 | vOTU_180 | 12,727 |  |  |
| NIFA_virome_64218 | vOTU_181 | 12,675 | vOTU_181 | bacterial prophage |
| NIFA_virome_63381 | vOTU_182 | 12,669 |  |  |
| NIFA_virome_64134 | vOTU_183 | 12,620 |  |  |
| NIFA_virome_63943 | vOTU_184 | 12,416 |  |  |
| NIFA_virome_62400 | vOTU_185 | 12,390 |  |  |
| NIFA_virome_63492 | vOTU_186 | 12,277 |  |  |
| NIFA_virome_60681 | vOTU_187 | 12,212 |  |  |
| NIFA_virome_62013 | vOTU_188 | 12,175 |  |  |
| NIFA_virome_48647 | vOTU_189 | 12,125 | vOTU_189 | Archaeal prophage |
| NIFA_virome_62173 | vOTU_190 | 12,090 |  |  |
| NIFA_virome_9264 | vOTU_191 | 12,034 | vOTU_191 | Archaeal prophage |
| NIFA_virome_63176 | vOTU_192 | 12,008 |  |  |
| NIFA_virome_61971 | vOTU_193 | 12,003 |  |  |
| NIFA_virome_28509 | vOTU_194 | 11,972 |  |  |
| NIFA_virome_23092 | vOTU_195 | 11,954 |  |  |
| NIFA_virome_63181 | vOTU_196 | 11,871 |  |  |
| NIFA_virome_62986 | vOTU_197 | 11,864 |  |  |
| NIFA_virome_55897 | vOTU_198 | 11,795 |  |  |
| NIFA_virome_19520 | vOTU_199 | 11,760 | vOTU_199 | Archaeal prophage |
| NIFA_virome_23606 | vOTU_200 | 11,754 |  |  |
| NIFA_virome_46485 | vOTU_201 | 11,750 |  |  |
| NIFA_virome_62659 | vOTU_202 | 11,723 |  |  |
| NIFA_virome_29952 | vOTU_203 | 11,687 | vOTU_203 | bacterial prophage |
| NIFA_virome_10658 | vOTU_204 | 11,650 |  |  |
| NIFA_virome_63001 | vOTU_205 | 11,617 |  |  |
| NIFA_virome_49231 | vOTU_206 | 11,592 |  |  |
| NIFA_virome_62103 | vOTU_207 | 11,531 | vOTU_207 | recombinase |
| NIFA_virome_34410 | vOTU_208 | 11,521 |  |  |
| NIFA_virome_61091 | vOTU_209 | 11,428 |  |  |
| NIFA_virome_36239 | vOTU_210 | 11,428 | vOTU_210 | Archaeal prophage |
| NIFA_virome_61363 | vOTU_211 | 11,413 |  |  |
| NIFA_virome_61766 | vOTU_212 | 11,391 |  |  |
| NIFA_virome_45576 | vOTU_213 | 11,373 |  |  |
| NIFA_virome_62749 | vOTU_214 | 11,262 | vOTU_214 | recombinase |
| NIFA_virome_61744 | vOTU_215 | 11,245 |  |  |
| NIFA_virome_61183 | vOTU_216 | 11,229 |  |  |
| NIFA_virome_45440 | vOTU_217 | 11,200 |  |  |
| NIFA_virome_21056 | vOTU_218 | 11,177 |  |  |
| NIFA_virome_62962 | vOTU_219 | 11,157 |  |  |
| NIFA_virome_53111 | vOTU_220 | 11,152 |  |  |
| NIFA_virome_56672 | vOTU_221 | 11,115 |  |  |
| NIFA_virome_62945 | vOTU_222 | 11,090 |  |  |
| NIFA_virome_57641 | vOTU_223 | 10,979 |  |  |
| NIFA_virome_31247 | vOTU_224 | 10,976 |  |  |
| NIFA_virome_60765 | vOTU_225 | 10,968 |  |  |
| NIFA_virome_60737 | vOTU_226 | 10,958 |  |  |
| NIFA_virome_63974 | vOTU_227 | 10,909 |  |  |
| NIFA_virome_62692 | vOTU_228 | 10,860 |  |  |
| NIFA_virome_62479 | vOTU_229 | 10,824 |  |  |
| NIFA_virome_62356 | vOTU_230 | 10,812 |  |  |
| NIFA_virome_63344 | vOTU_231 | 10,726 |  |  |
| NIFA_virome_30307 | vOTU_232 | 10,717 | vOTU_232 | Archaeal prophage |
| NIFA_virome_61704 | vOTU_233 | 10,706 |  |  |
| NIFA_virome_8907 | vOTU_234 | 10,651 | vOTU_234 | Archaeal prophage |
| NIFA_virome_58985 | vOTU_235 | 10,647 |  |  |
| NIFA_virome_62939 | vOTU_236 | 10,630 |  |  |
| NIFA_virome_64230 | vOTU_237 | 10,541 | vOTU_237 | Archaeal prophage |
| NIFA_virome_25568 | vOTU_238 | 10,530 |  |  |
| NIFA_virome_54186 | vOTU_239 | 10,524 |  |  |
| NIFA_virome_63864 | vOTU_240 | 10,523 |  |  |
| NIFA_virome_61996 | vOTU_241 | 10,518 |  |  |
| NIFA_virome_61390 | vOTU_242 | 10,447 |  |  |
| NIFA_virome_36582 | vOTU_243 | 10,431 | vOTU_243 | Archaeal prophage |
| NIFA_virome_62109 | vOTU_244 | 10,402 |  |  |
| NIFA_virome_61703 | vOTU_245 | 10,382 |  |  |
| NIFA_virome_52407 | vOTU_246 | 10,304 |  |  |
| NIFA_virome_61419 | vOTU_247 | 10,285 |  |  |
| NIFA_virome_62558 | vOTU_248 | 10,274 |  |  |
| NIFA_virome_61761 | vOTU_249 | 10,262 |  |  |
| NIFA_virome_62211 | vOTU_250 | 10,243 |  |  |
| NIFA_virome_55160 | vOTU_251 | 10,221 | vOTU_251 | Archaeal prophage |
| NIFA_virome_51951 | vOTU_252 | 10,165 | vOTU_252 | bacterial prophage |
| NIFA_virome_62169 | vOTU_253 | 10,154 |  |  |
| NIFA_virome_1612 | vOTU_254 | 10,146 |  |  |
| NIFA_virome_62365 | vOTU_255 | 10,128 |  |  |
| NIFA_virome_60810 | vOTU_256 | 10,094 |  |  |
| NIFA_virome_27825 | vOTU_257 | 10,094 |  |  |
| NIFA_virome_61316 | vOTU_258 | 10,051 |  |  |
| NIFA_virome_7733 | vOTU_259 | 10,048 |  |  |
| NIFA_virome_62649 | vOTU_260 | 10,019 |  |  |
