# Supplementary material for: Identification of Novel Viruses and Their Microbial Hosts from Soils with Long-Term Nitrogen Fertilization and Cover Cropping Management
Source: mSystems. 2022 Nov 29;7(6):e00571-22. doi: 10.1128/msystems.00571-22 (PMC9765229; doi:10.1128/msystems.00571-22)
Supplement: TABLE S7 [file msystems.00571-22-s0007.docx]

**Table S7**

| Genome | Order | Family | Genus | VC | VC Status | VC Subcluster |
| --- | --- | --- | --- | --- | --- | --- |
| Achromobacter~phage~83-24 | Caudovirales | Siphoviridae | Jwxvirus | 0_0 | Clustered | VC_0_0 |
| Achromobacter~phage~JWAlpha | Caudovirales | Podoviridae | Jwalphavirus | 7_1 | Clustered | VC_7_1 |
| Achromobacter~phage~JWX | Caudovirales | Siphoviridae | Jwxvirus | 0_0 | Clustered | VC_0_0 |
| Achromobacter~phage~phiAxp-1 | Caudovirales | Siphoviridae | Unassigned | 1_0 | Clustered | VC_1_0 |
| Achromobacter~phage~phiAxp-3 | Caudovirales | Podoviridae | Jwalphavirus | 7_1 | Clustered | VC_7_1 |
| Acidianus~bottle-shaped~virus | Unassigned | Ampullaviridae | Ampullavirus | 17_0 | Clustered | VC_17_0 |
| Acidianus~bottle-shaped~virus~2 | Unassigned | Ampullaviridae | Ampullavirus | 17_0 | Clustered | VC_17_0 |
| Acidianus~bottle-shaped~virus~3 | Unassigned | Ampullaviridae | Ampullavirus | 17_0 | Clustered | VC_17_0 |
| Acidianus~filamentous~virus~3 | Ligamenvirales | Lipothrixviridae | Betalipothrixvirus | 18_0 | Clustered | VC_18_0 |
| Acidianus~filamentous~virus~6 | Ligamenvirales | Lipothrixviridae | Betalipothrixvirus | 18_0 | Clustered | VC_18_0 |
| Acidianus~filamentous~virus~7 | Ligamenvirales | Lipothrixviridae | Betalipothrixvirus | 18_0 | Clustered | VC_18_0 |
| Acidianus~filamentous~virus~8 | Ligamenvirales | Lipothrixviridae | Betalipothrixvirus | 18_0 | Clustered | VC_18_0 |
| Acidianus~filamentous~virus~9 | Ligamenvirales | Lipothrixviridae | Betalipothrixvirus | 18_0 | Clustered | VC_18_0 |
| Acidianus~spindle-shaped~virus~1 | Unassigned | Fuselloviridae | Betafusellovirus | 21_0 | Clustered | VC_21_0 |
| Acinetobacter~phage~AB1 | Caudovirales | Myoviridae | Ap22virus | 28_0 | Clustered | VC_28_0 |
| Acinetobacter~phage~AB3 | Caudovirales | Podoviridae | Fri1virus | 30_0 | Clustered | VC_30_0 |
| Acinetobacter~phage~AP22 | Caudovirales | Myoviridae | Ap22virus | 28_0 | Clustered | VC_28_0 |
| Acinetobacter~phage~AbP2 | Caudovirales | Myoviridae | Obolenskvirus | 28_0 | Clustered | VC_28_0 |
| Acinetobacter~phage~Abp1 | Caudovirales | Podoviridae | Fri1virus | 30_0 | Clustered | VC_30_0 |
| Acinetobacter~phage~Bphi-B1251 | Caudovirales | Siphoviridae | Vieuvirus | 40_0 | Clustered | VC_40_0 |
| Acinetobacter~phage~Fri1 | Caudovirales | Podoviridae | Fri1virus | 30_0 | Clustered | VC_30_0 |
| Acinetobacter~phage~IME-AB2 | Caudovirales | Myoviridae | Ap22virus | 28_0 | Clustered | VC_28_0 |
| Acinetobacter~phage~IME200 | Caudovirales | Podoviridae | Fri1virus | 30_0 | Clustered | VC_30_0 |
| Acinetobacter~phage~IME_AB3 | Caudovirales | Siphoviridae | Unassigned | 1_0 | Clustered | VC_1_0 |
| Acinetobacter~phage~LZ35 | Caudovirales | Myoviridae | Obolenskvirus | 28_0 | Clustered | VC_28_0 |
| Acinetobacter~phage~Petty | Caudovirales | Podoviridae | Friunavirus | 30_0 | Clustered | VC_30_0 |
| Acinetobacter~phage~SH-Ab~15519 | Caudovirales | Podoviridae | Friunavirus | 30_0 | Clustered | VC_30_0 |
| Acinetobacter~phage~WCHABP1 | Caudovirales | Myoviridae | Obolenskvirus | 28_0 | Clustered | VC_28_0 |
| Acinetobacter~phage~WCHABP12 | Caudovirales | Myoviridae | Obolenskvirus | 28_0 | Clustered | VC_28_0 |
| Acinetobacter~phage~WCHABP5 | Caudovirales | Podoviridae | Friunavirus | 30_0 | Clustered | VC_30_0 |
| Acinetobacter~phage~YMC-13-01-C62 | Caudovirales | Myoviridae | Ap22virus | 28_0 | Clustered | VC_28_0 |
| Acinetobacter~phage~YMC11/11/R3177 | Caudovirales | Siphoviridae | Vieuvirus | 40_0 | Clustered | VC_40_0 |
| Acinetobacter~phage~YMC11/12/R2315 | Caudovirales | Myoviridae | Obolenskvirus | 28_0 | Clustered | VC_28_0 |
| Acinetobacter~phage~phiAB1 | Caudovirales | Podoviridae | Fri1virus | 30_0 | Clustered | VC_30_0 |
| Acinetobacter~phage~phiAB6 | Caudovirales | Podoviridae | Friunavirus | 30_0 | Clustered | VC_30_0 |
| Acinetobacter~phage~vB_AbaM_Acibel004 | Caudovirales | Myoviridae | Unassigned | 45_0 | Clustered | VC_45_0 |
| Acinetobacter~phage~vB_AbaM_phiAbaA1 | Caudovirales | Myoviridae | Unassigned | 45_0 | Clustered | VC_45_0 |
| Acinetobacter~phage~vB_AbaP_AS11 | Caudovirales | Podoviridae | Friunavirus | 30_0 | Clustered | VC_30_0 |
| Acinetobacter~phage~vB_AbaP_AS12 | Caudovirales | Podoviridae | Friunavirus | 30_0 | Clustered | VC_30_0 |
| Acinetobacter~phage~vB_AbaP_Acibel007 | Caudovirales | Podoviridae | Friunavirus | 30_0 | Clustered | VC_30_0 |
| Acinetobacter~phage~vB_AbaP_B1 | Caudovirales | Podoviridae | Friunavirus | 30_0 | Clustered | VC_30_0 |
| Acinetobacter~phage~vB_AbaP_B3 | Caudovirales | Podoviridae | Friunavirus | 30_0 | Clustered | VC_30_0 |
| Acinetobacter~phage~vB_AbaP_B5 | Caudovirales | Podoviridae | Friunavirus | 30_0 | Clustered | VC_30_0 |
| Acinetobacter~phage~vB_AbaP_D2 | Caudovirales | Podoviridae | Friunavirus | 30_0 | Clustered | VC_30_0 |
| Acinetobacter~phage~vB_AbaP_PD-6A3 | Caudovirales | Podoviridae | Fri1virus | 30_0 | Clustered | VC_30_0 |
| Acinetobacter~phage~vB_AbaP_PD-AB9 | Caudovirales | Podoviridae | Fri1virus | 30_0 | Clustered | VC_30_0 |
| Acinetobacter~phage~vB_AbaS_Loki | Caudovirales | Siphoviridae | Unassigned | 1_0 | Clustered | VC_1_0 |
| Acinetobacter~phage~vB_AbaS_TRS1 | Caudovirales | Siphoviridae | Vieuvirus | 40_0 | Clustered | VC_40_0 |
| Acinetobacter~phage~vB_ApiP_P1 | Caudovirales | Podoviridae | Friunavirus | 30_0 | Clustered | VC_30_0 |
| Acinetobacter~phage~vB_ApiP_P2 | Caudovirales | Podoviridae | Friunavirus | 30_0 | Clustered | VC_30_0 |
| Aeromonas~phage~AS-gz | Caudovirales | Myoviridae | Tulanevirus | 35_1 | Clustered | VC_35_1 |
| Aeromonas~phage~Aes012 | Caudovirales | Myoviridae | Secunda5virus | 35_1 | Clustered | VC_35_1 |
| Aeromonas~phage~Aes508 | Caudovirales | Myoviridae | Secunda5virus | 35_1 | Clustered | VC_35_1 |
| Aeromonas~phage~CC2 | Caudovirales | Myoviridae | Unassigned | 36_0 | Clustered | VC_36_0 |
| Aeromonas~phage~PX29 | Caudovirales | Myoviridae | Unassigned | 36_1 | Clustered | VC_36_1 |
| Aeromonas~phage~pAh6-C | Caudovirales | Myoviridae | Unassigned | 59_0 | Clustered | VC_59_0 |
| Aeromonas~phage~pIS4-A | Caudovirales | Siphoviridae | Pis4avirus | 61_0 | Clustered | VC_61_0 |
| Aeromonas~phage~phiAS4 | Caudovirales | Myoviridae | Secunda5virus | 35_1 | Clustered | VC_35_1 |
| Aeromonas~phage~phiAS5 | Caudovirales | Myoviridae | Unassigned | 36_1 | Clustered | VC_36_1 |
| Aeromonas~phage~phiAS7 | Caudovirales | Podoviridae | Unassigned | 68_0 | Clustered | VC_68_0 |
| Aeromonas~virus~25 | Caudovirales | Myoviridae | Secunda5virus | 35_1 | Clustered | VC_35_1 |
| Aeromonas~virus~31 | Caudovirales | Myoviridae | Secunda5virus | 35_0 | Clustered | VC_35_0 |
| Aeromonas~virus~44RR2 | Caudovirales | Myoviridae | Biquartavirus | 35_0 | Clustered | VC_35_0 |
| Aeromonas~virus~65 | Caudovirales | Myoviridae | Unassigned | 36_0 | Clustered | VC_36_0 |
| Aeromonas~virus~Aeh1 | Caudovirales | Myoviridae | Unassigned | 36_1 | Clustered | VC_36_1 |
| Aeromonas~virus~phiO18P | Caudovirales | Myoviridae | Hp1virus | 70_0 | Clustered | VC_70_0 |
| Aggregatibacter~phage~S1249 | Caudovirales | Myoviridae | Unassigned | 6_0 | Clustered | VC_6_0 |
| Agrobacterium~phage~7-7-1 | Caudovirales | Myoviridae | Unassigned | 71_0 | Clustered | VC_71_0 |
| Alces~alces~faeces~associated~microvirus~MP10~5560 | Unassigned | Microviridae | Unassigned | 73_0 | Clustered | VC_73_0 |
| Alces~alces~faeces~associated~microvirus~MP11~5517 | Unassigned | Microviridae | Unassigned | 73_0 | Clustered | VC_73_0 |
| Alces~alces~faeces~associated~microvirus~MP12~5423 | Unassigned | Microviridae | Unassigned | 73_0 | Clustered | VC_73_0 |
| Aquamicrobium~phage~P14 | Caudovirales | Podoviridae | Aqualcavirus | 67_0 | Clustered | VC_67_0 |
| Archaeal~BJ1~virus | Caudovirales | Siphoviridae | Unassigned | 82_0 | Clustered | VC_82_0 |
| Arthrobacter~phage~Abidatro | Caudovirales | Siphoviridae | Galaxyvirus | 84_0 | Clustered | VC_84_0 |
| Arthrobacter~phage~Adat | Caudovirales | Podoviridae | Jasminevirus | 89_0 | Clustered | VC_89_0 |
| Arthrobacter~phage~Amigo | Caudovirales | Siphoviridae | Amigovirus | 90_0 | Clustered | VC_90_0 |
| Arthrobacter~phage~BarretLemon | Caudovirales | Myoviridae | Marthavirus | 92_0 | Clustered | VC_92_0 |
| Arthrobacter~phage~Beans | Caudovirales | Myoviridae | Marthavirus | 92_0 | Clustered | VC_92_0 |
| Arthrobacter~phage~Bennie | Caudovirales | Siphoviridae | Korravirus | 83_0 | Clustered | VC_83_0 |
| Arthrobacter~phage~Brent | Caudovirales | Myoviridae | Marthavirus | 92_0 | Clustered | VC_92_0 |
| Arthrobacter~phage~CaptnMurica | Caudovirales | Siphoviridae | Gordonvirus | 94_1 | Clustered | VC_94_1 |
| Arthrobacter~phage~Circum | Caudovirales | Siphoviridae | Mudcatvirus | 94_0 | Clustered | VC_94_0 |
| Arthrobacter~phage~Colucci | Caudovirales | Myoviridae | Radnorvirus | 93_0 | Clustered | VC_93_0 |
| Arthrobacter~phage~DrRobert | Caudovirales | Siphoviridae | Korravirus | 83_0 | Clustered | VC_83_0 |
| Arthrobacter~phage~Galaxy | Caudovirales | Siphoviridae | Galaxyvirus | 84_0 | Clustered | VC_84_0 |
| Arthrobacter~phage~Glenn | Caudovirales | Siphoviridae | Korravirus | 83_0 | Clustered | VC_83_0 |
| Arthrobacter~phage~Gordon | Caudovirales | Siphoviridae | Gordonvirus | 94_1 | Clustered | VC_94_1 |
| Arthrobacter~phage~HunterDalle | Caudovirales | Siphoviridae | Korravirus | 83_0 | Clustered | VC_83_0 |
| Arthrobacter~phage~Jasmine | Caudovirales | Podoviridae | Jasminevirus | 89_0 | Clustered | VC_89_0 |
| Arthrobacter~phage~Jawnski | Caudovirales | Myoviridae | Marthavirus | 92_0 | Clustered | VC_92_0 |
| Arthrobacter~phage~Joann | Caudovirales | Siphoviridae | Korravirus | 83_0 | Clustered | VC_83_0 |
| Arthrobacter~phage~KellEzio | Caudovirales | Siphoviridae | Kelleziovirus | 98_0 | Clustered | VC_98_0 |
| Arthrobacter~phage~Kitkat | Caudovirales | Siphoviridae | Kelleziovirus | 98_0 | Clustered | VC_98_0 |
| Arthrobacter~phage~Korra | Caudovirales | Siphoviridae | Korravirus | 83_0 | Clustered | VC_83_0 |
| Arthrobacter~phage~Martha | Caudovirales | Myoviridae | Marthavirus | 92_0 | Clustered | VC_92_0 |
| Arthrobacter~phage~Molivia | Caudovirales | Siphoviridae | Amigovirus | 90_0 | Clustered | VC_90_0 |
| Arthrobacter~phage~Mudcat | Caudovirales | Siphoviridae | Mudcatvirus | 94_0 | Clustered | VC_94_0 |
| Arthrobacter~phage~Piccoletto | Caudovirales | Myoviridae | Marthavirus | 92_0 | Clustered | VC_92_0 |
| Arthrobacter~phage~Preamble | Caudovirales | Siphoviridae | Korravirus | 83_0 | Clustered | VC_83_0 |
| Arthrobacter~phage~PrincessTrina | Caudovirales | Myoviridae | Arv1virus | 93_0 | Clustered | VC_93_0 |
| Arthrobacter~phage~Pumancara | Caudovirales | Siphoviridae | Korravirus | 83_0 | Clustered | VC_83_0 |
| Arthrobacter~phage~Shade | Caudovirales | Myoviridae | Marthavirus | 92_0 | Clustered | VC_92_0 |
| Arthrobacter~phage~Sonny | Caudovirales | Myoviridae | Marthavirus | 92_0 | Clustered | VC_92_0 |
| Arthrobacter~phage~Wayne | Caudovirales | Siphoviridae | Korravirus | 83_0 | Clustered | VC_83_0 |
| Arthrobacter~phage~vB_ArtM-ArV1 | Caudovirales | Myoviridae | Arv1virus | 93_0 | Clustered | VC_93_0 |
| Bacillus~phage~0305phi8-36 | Caudovirales | Myoviridae | Unassigned | 108_1 | Clustered/Singleton | VC_108_1 |
| Bacillus~phage~AR9 | Caudovirales | Myoviridae | Unassigned | 109_0 | Clustered/Singleton | VC_109_0 |
| Bacillus~phage~Aurora | Caudovirales | Podoviridae | Salasvirus | 51_0 | Clustered | VC_51_0 |
| Bacillus~phage~BPS10C | Caudovirales | Herelleviridae | Wphvirus | 111_0 | Clustered | VC_111_0 |
| Bacillus~phage~BPS13 | Caudovirales | Myoviridae | Wphvirus | 111_0 | Clustered | VC_111_0 |
| Bacillus~phage~Bam35c | Unassigned | Tectiviridae | Betatectivirus | 127_0 | Clustered | VC_127_0 |
| Bacillus~phage~Belinda | Caudovirales | Herelleviridae | Wphvirus | 111_0 | Clustered | VC_111_0 |
| Bacillus~phage~BtCS33 | Caudovirales | Siphoviridae | Unassigned | 129_0 | Clustered | VC_129_0 |
| Bacillus~phage~CP-51 | Caudovirales | Myoviridae | Cp51virus | 112_1 | Clustered | VC_112_1 |
| Bacillus~phage~CampHawk | Caudovirales | Myoviridae | Spo1virus | 112_0 | Clustered | VC_112_0 |
| Bacillus~phage~Claudi | Caudovirales | Podoviridae | Salasvirus | 51_0 | Clustered | VC_51_0 |
| Bacillus~phage~DIGNKC | Caudovirales | Herelleviridae | Wphvirus | 111_0 | Clustered | VC_111_0 |
| Bacillus~phage~DirtyBetty | Caudovirales | Herelleviridae | Wphvirus | 111_0 | Clustered | VC_111_0 |
| Bacillus~phage~Eyuki | Caudovirales | Herelleviridae | Wphvirus | 111_0 | Clustered | VC_111_0 |
| Bacillus~phage~Fah | Caudovirales | Siphoviridae | Wbetavirus | 129_0 | Clustered | VC_129_0 |
| Bacillus~phage~GIL16c | Unassigned | Tectiviridae | Betatectivirus | 127_0 | Clustered | VC_127_0 |
| Bacillus~phage~Gamma | Caudovirales | Siphoviridae | Wbetavirus | 129_0 | Clustered | VC_129_0 |
| Bacillus~phage~Hakuna | Caudovirales | Myoviridae | Wphvirus | 111_0 | Clustered | VC_111_0 |
| Bacillus~phage~JL | Caudovirales | Myoviridae | Cp51virus | 112_1 | Clustered | VC_112_1 |
| Bacillus~phage~Kida | Caudovirales | Herelleviridae | Wphvirus | 111_0 | Clustered | VC_111_0 |
| Bacillus~phage~MG-B1 | Caudovirales | Podoviridae | Salasvirus | 51_0 | Clustered | VC_51_0 |
| Bacillus~phage~Megatron | Caudovirales | Myoviridae | Wphvirus | 111_0 | Clustered | VC_111_0 |
| Bacillus~phage~Mgbh1 | Caudovirales | Siphoviridae | Magadivirus | 137_0 | Clustered | VC_137_0 |
| Bacillus~phage~Nemo | Caudovirales | Herelleviridae | Wphvirus | 111_0 | Clustered | VC_111_0 |
| Bacillus~phage~Nigalana | Caudovirales | Herelleviridae | Wphvirus | 111_0 | Clustered | VC_111_0 |
| Bacillus~phage~NotTheCreek | Caudovirales | Herelleviridae | Wphvirus | 111_0 | Clustered | VC_111_0 |
| Bacillus~phage~PBC1 | Caudovirales | Siphoviridae | Unassigned | 118_0 | Clustered | VC_118_0 |
| Bacillus~phage~PM1 | Caudovirales | Siphoviridae | Unassigned | 143_0 | Clustered | VC_143_0 |
| Bacillus~phage~Page | Caudovirales | Podoviridae | Pagevirus | 148_0 | Clustered | VC_148_0 |
| Bacillus~phage~Palmer | Caudovirales | Podoviridae | Pagevirus | 148_0 | Clustered | VC_148_0 |
| Bacillus~phage~Pascal | Caudovirales | Podoviridae | Pagevirus | 148_0 | Clustered | VC_148_0 |
| Bacillus~phage~Pavlov | Caudovirales | Podoviridae | Pagevirus | 148_0 | Clustered | VC_148_0 |
| Bacillus~phage~Pony | Caudovirales | Podoviridae | Pagevirus | 148_0 | Clustered | VC_148_0 |
| Bacillus~phage~Pookie | Caudovirales | Podoviridae | Pagevirus | 148_0 | Clustered | VC_148_0 |
| Bacillus~phage~SageFayge | Caudovirales | Herelleviridae | Wphvirus | 111_0 | Clustered | VC_111_0 |
| Bacillus~phage~SalinJah | Caudovirales | Herelleviridae | Wphvirus | 111_0 | Clustered | VC_111_0 |
| Bacillus~phage~Shanette | Caudovirales | Myoviridae | Cp51virus | 112_1 | Clustered | VC_112_1 |
| Bacillus~phage~Slash | Caudovirales | Siphoviridae | Slashvirus | 152_0 | Clustered | VC_152_0 |
| Bacillus~phage~Stahl | Caudovirales | Siphoviridae | Slashvirus | 152_0 | Clustered | VC_152_0 |
| Bacillus~phage~Staley | Caudovirales | Siphoviridae | Slashvirus | 152_0 | Clustered | VC_152_0 |
| Bacillus~phage~Stills | Caudovirales | Siphoviridae | Slashvirus | 152_0 | Clustered | VC_152_0 |
| Bacillus~phage~Stitch | Caudovirales | Podoviridae | Salasvirus | 51_0 | Clustered | VC_51_0 |
| Bacillus~phage~TP21-L | Caudovirales | Siphoviridae | Tp21virus | 133_0 | Clustered | VC_133_0 |
| Bacillus~phage~W.Ph. | Caudovirales | Myoviridae | Wphvirus | 111_0 | Clustered | VC_111_0 |
| Bacillus~phage~Wip1 | Unassigned | Tectiviridae | Betatectivirus | 127_0 | Clustered | VC_127_0 |
| Bacillus~phage~Zuko | Caudovirales | Herelleviridae | Wphvirus | 111_0 | Clustered | VC_111_0 |
| Bacillus~phage~phIS3501 | Caudovirales | Siphoviridae | Unassigned | 129_0 | Clustered | VC_129_0 |
| Bacillus~phage~phiCM3 | Caudovirales | Siphoviridae | Unassigned | 129_0 | Clustered | VC_129_0 |
| Bacillus~phage~vB_BanS-Tsamsa | Caudovirales | Siphoviridae | Unassigned | 113_3 | Clustered/Singleton | VC_113_3 |
| Bacillus~virus~1 | Caudovirales | Myoviridae | Svunavirus | 136_0 | Clustered | VC_136_0 |
| Bacillus~virus~250 | Caudovirales | Siphoviridae | Cecivirus | 118_0 | Clustered | VC_118_0 |
| Bacillus~virus~AP50 | Unassigned | Tectiviridae | Betatectivirus | 127_0 | Clustered | VC_127_0 |
| Bacillus~virus~Andromeda | Caudovirales | Siphoviridae | Andromedavirus | 156_0 | Clustered | VC_156_0 |
| Bacillus~virus~BMBtp2 | Caudovirales | Siphoviridae | Tp21virus | 133_0 | Clustered | VC_133_0 |
| Bacillus~virus~Blastoid | Caudovirales | Siphoviridae | Andromedavirus | 156_0 | Clustered | VC_156_0 |
| Bacillus~virus~Curly | Caudovirales | Siphoviridae | Andromedavirus | 156_0 | Clustered | VC_156_0 |
| Bacillus~virus~Eoghan | Caudovirales | Siphoviridae | Andromedavirus | 156_0 | Clustered | VC_156_0 |
| Bacillus~virus~Finn | Caudovirales | Siphoviridae | Andromedavirus | 156_0 | Clustered | VC_156_0 |
| Bacillus~virus~Glittering | Caudovirales | Siphoviridae | Andromedavirus | 156_0 | Clustered | VC_156_0 |
| Bacillus~virus~IEBH | Caudovirales | Siphoviridae | Cecivirus | 118_0 | Clustered | VC_118_0 |
| Bacillus~virus~Riggi | Caudovirales | Siphoviridae | Andromedavirus | 156_0 | Clustered | VC_156_0 |
| Bacillus~virus~SPO1 | Caudovirales | Myoviridae | Spo1virus | 112_0 | Clustered | VC_112_0 |
| Bacillus~virus~SPbeta | Caudovirales | Siphoviridae | Spbetavirus | 113_0 | Clustered/Singleton | VC_113_0 |
| Bacillus~virus~Taylor | Caudovirales | Siphoviridae | Andromedavirus | 156_0 | Clustered | VC_156_0 |
| Bacillus~virus~Wbeta | Caudovirales | Siphoviridae | Wbetavirus | 129_0 | Clustered | VC_129_0 |
| Bacteriophage~APSE-2 | Caudovirales | Podoviridae | Unassigned | 162_0 | Clustered | VC_162_0 |
| Bacteriophage~Lily | Caudovirales | Siphoviridae | Lilyvirus | 166_0 | Clustered | VC_166_0 |
| Bacteroides~phage~B124-14 | Caudovirales | Siphoviridae | Unassigned | 141_0 | Clustered | VC_141_0 |
| Bacteroides~phage~B40-8 | Caudovirales | Siphoviridae | Unassigned | 141_0 | Clustered | VC_141_0 |
| Bdellovibrio~phage~phiMH2K | Unassigned | Microviridae | Bdellomicrovirus | 73_0 | Clustered | VC_73_0 |
| Bordetella~virus~BPP1 | Caudovirales | Podoviridae | Bpp1virus | 161_0 | Clustered | VC_161_0 |
| Brevibacillus~phage~Abouo | Caudovirales | Myoviridae | Abouovirus | 153_0 | Clustered | VC_153_0 |
| Brevibacillus~phage~Davies | Caudovirales | Myoviridae | Abouovirus | 153_0 | Clustered | VC_153_0 |
| Brevibacillus~phage~Jenst | Caudovirales | Siphoviridae | Jenstvirus | 108_0 | Clustered | VC_108_0 |
| Brevibacillus~phage~Jimmer1 | Caudovirales | Myoviridae | Jimmervirus | 153_0 | Clustered | VC_153_0 |
| Brevibacillus~phage~Jimmer2 | Caudovirales | Myoviridae | Jimmervirus | 153_0 | Clustered | VC_153_0 |
| Brevibacillus~phage~Osiris | Caudovirales | Myoviridae | Jimmervirus | 153_0 | Clustered | VC_153_0 |
| Brevibacillus~phage~Sundance | Caudovirales | Siphoviridae | Unassigned | 113_4 | Clustered/Singleton | VC_113_4 |
| Brochothrix~phage~NF5 | Caudovirales | Siphoviridae | Unassigned | 174_0 | Clustered/Singleton | VC_174_0 |
| Brucella~phage~BiPBO1 | Caudovirales | Siphoviridae | Unassigned | 177_0 | Clustered | VC_177_0 |
| Brucella~phage~Pr | Caudovirales | Podoviridae | Prtbvirus | 178_0 | Clustered | VC_178_0 |
| Brucella~phage~Tb | Caudovirales | Podoviridae | Prtbvirus | 178_0 | Clustered | VC_178_0 |
| Burkholderia~phage~Bcep176 | Caudovirales | Siphoviridae | Stanholtvirus | 182_0 | Clustered | VC_182_0 |
| Burkholderia~phage~JG068 | Caudovirales | Podoviridae | Unassigned | 67_0 | Clustered | VC_67_0 |
| Burkholderia~phage~KL1 | Caudovirales | Siphoviridae | Septima3virus | 1_0 | Clustered | VC_1_0 |
| Burkholderia~phage~KL3 | Caudovirales | Myoviridae | Peduovirus | 69_0 | Clustered | VC_69_0 |
| Burkholderia~phage~KS14 | Caudovirales | Myoviridae | Peduovirus | 69_0 | Clustered | VC_69_0 |
| Burkholderia~phage~KS5 | Caudovirales | Myoviridae | Peduovirus | 69_0 | Clustered | VC_69_0 |
| Burkholderia~phage~KS9 | Caudovirales | Siphoviridae | Stanholtvirus | 182_0 | Clustered | VC_182_0 |
| Burkholderia~phage~ST79 | Caudovirales | Myoviridae | Peduovirus | 69_0 | Clustered | VC_69_0 |
| Burkholderia~virus~Bcep1 | Caudovirales | Myoviridae | Bcep78virus | 29_0 | Clustered | VC_29_0 |
| Burkholderia~virus~Bcep22 | Caudovirales | Podoviridae | Bcep22virus | 193_0 | Clustered | VC_193_0 |
| Burkholderia~virus~Bcep43 | Caudovirales | Myoviridae | Bcep78virus | 29_0 | Clustered | VC_29_0 |
| Burkholderia~virus~Bcep781 | Caudovirales | Myoviridae | Bcep78virus | 29_0 | Clustered | VC_29_0 |
| Burkholderia~virus~BcepC6B | Caudovirales | Podoviridae | Bpp1virus | 161_0 | Clustered | VC_161_0 |
| Burkholderia~virus~BcepNY3 | Caudovirales | Myoviridae | Bcep78virus | 29_0 | Clustered | VC_29_0 |
| Burkholderia~virus~Bcepil02 | Caudovirales | Podoviridae | Bcep22virus | 193_0 | Clustered | VC_193_0 |
| Burkholderia~virus~Bcepmigl | Caudovirales | Podoviridae | Bcep22virus | 193_0 | Clustered | VC_193_0 |
| Burkholderia~virus~DC1 | Caudovirales | Podoviridae | Bcep22virus | 193_0 | Clustered | VC_193_0 |
| Burkholderia~virus~phi1026b | Caudovirales | Siphoviridae | E125virus | 182_0 | Clustered | VC_182_0 |
| Burkholderia~virus~phi52237 | Caudovirales | Myoviridae | P2virus | 69_0 | Clustered | VC_69_0 |
| Burkholderia~virus~phi6442 | Caudovirales | Siphoviridae | E125virus | 182_0 | Clustered | VC_182_0 |
| Burkholderia~virus~phiE122 | Caudovirales | Myoviridae | P2virus | 69_0 | Clustered | VC_69_0 |
| Burkholderia~virus~phiE125 | Caudovirales | Siphoviridae | E125virus | 182_0 | Clustered | VC_182_0 |
| Burkholderia~virus~phiE202 | Caudovirales | Myoviridae | P2virus | 69_0 | Clustered | VC_69_0 |
| Campylobacter~phage~CP30A | Caudovirales | Myoviridae | Cp8virus | 195_0 | Clustered | VC_195_0 |
| Campylobacter~phage~PC14 | Caudovirales | Myoviridae | Fletchervirus | 195_0 | Clustered | VC_195_0 |
| Campylobacter~phage~vB_CjeM_Los1 | Caudovirales | Myoviridae | Fletchervirus | 195_0 | Clustered | VC_195_0 |
| Campylobacter~virus~CP21 | Caudovirales | Myoviridae | Cp220virus | 196_0 | Clustered | VC_196_0 |
| Campylobacter~virus~CP220 | Caudovirales | Myoviridae | Cp220virus | 196_0 | Clustered | VC_196_0 |
| Campylobacter~virus~CP81 | Caudovirales | Myoviridae | Cp8virus | 195_0 | Clustered | VC_195_0 |
| Campylobacter~virus~CPX | Caudovirales | Myoviridae | Cp8virus | 195_0 | Clustered | VC_195_0 |
| Campylobacter~virus~CPt10 | Caudovirales | Myoviridae | Cp220virus | 196_0 | Clustered | VC_196_0 |
| Campylobacter~virus~IBB35 | Caudovirales | Myoviridae | Cp220virus | 196_0 | Clustered | VC_196_0 |
| Campylobacter~virus~NCTC12673 | Caudovirales | Myoviridae | Cp8virus | 195_0 | Clustered | VC_195_0 |
| Caulobacter~phage~CcrColossus | Caudovirales | Siphoviridae | Unassigned | 79_2 | Clustered/Singleton | VC_79_2 |
| Caulobacter~phage~Percy | Caudovirales | Podoviridae | Aqualcavirus | 67_0 | Clustered | VC_67_0 |
| Caulobacter~phage~phiCb5 | Unassigned | Leviviridae | Unassigned | 198_0 | Clustered | VC_198_0 |
| Caulobacter~virus~Karma | Caudovirales | Siphoviridae | Phicbkvirus | 79_0 | Clustered | VC_79_0 |
| Caulobacter~virus~Magneto | Caudovirales | Siphoviridae | Phicbkvirus | 79_0 | Clustered | VC_79_0 |
| Caulobacter~virus~Rogue | Caudovirales | Siphoviridae | Phicbkvirus | 79_1 | Clustered/Singleton | VC_79_1 |
| Caulobacter~virus~Swift | Caudovirales | Siphoviridae | Phicbkvirus | 79_0 | Clustered | VC_79_0 |
| Caulobacter~virus~phiCbK | Caudovirales | Siphoviridae | Phicbkvirus | 79_0 | Clustered | VC_79_0 |
| Celeribacter~phage~P12053L | Caudovirales | Podoviridae | Unassigned | 200_0 | Clustered | VC_200_0 |
| Cellulophaga~phage~phi10:1 | Caudovirales | Siphoviridae | Unassigned | 204_0 | Clustered | VC_204_0 |
| Cellulophaga~phage~phi12:1 | Caudovirales | Siphoviridae | Cba181virus | 206_0 | Clustered | VC_206_0 |
| Cellulophaga~phage~phi12:2 | Unassigned | Microviridae | Unassigned | 207_0 | Clustered | VC_207_0 |
| Cellulophaga~phage~phi12a:1 | Unassigned | Microviridae | Unassigned | 207_0 | Clustered | VC_207_0 |
| Cellulophaga~phage~phi13:2 | Caudovirales | Podoviridae | Unassigned | 203_0 | Clustered | VC_203_0 |
| Cellulophaga~phage~phi17:1 | Caudovirales | Siphoviridae | Cba181virus | 206_0 | Clustered | VC_206_0 |
| Cellulophaga~phage~phi17:2 | Caudovirales | Podoviridae | Cba41virus | 205_0 | Clustered | VC_205_0 |
| Cellulophaga~phage~phi18:1 | Caudovirales | Siphoviridae | Cba181virus | 206_0 | Clustered | VC_206_0 |
| Cellulophaga~phage~phi18:3 | Caudovirales | Podoviridae | Unassigned | 203_0 | Clustered | VC_203_0 |
| Cellulophaga~phage~phi19:1 | Caudovirales | Siphoviridae | Unassigned | 204_0 | Clustered | VC_204_0 |
| Cellulophaga~phage~phi19:3 | Caudovirales | Podoviridae | Unassigned | 203_0 | Clustered | VC_203_0 |
| Cellulophaga~phage~phi46:3 | Caudovirales | Podoviridae | Unassigned | 203_0 | Clustered | VC_203_0 |
| Cellulophaga~phage~phi4:1 | Caudovirales | Podoviridae | Cba41virus | 205_0 | Clustered | VC_205_0 |
| Chimpanzee~faeces~associated~microphage~1 | Unassigned | Microviridae | Unassigned | 75_0 | Clustered | VC_75_0 |
| Chimpanzee~faeces~associated~microphage~3 | Unassigned | Microviridae | Unassigned | 75_0 | Clustered | VC_75_0 |
| Chlamydia~phage~2 | Unassigned | Microviridae | Chlamydiamicrovirus | 73_0 | Clustered | VC_73_0 |
| Chlamydia~phage~4 | Unassigned | Microviridae | Chlamydiamicrovirus | 73_0 | Clustered | VC_73_0 |
| Chlamydia~virus~CPAR39 | Unassigned | Microviridae | Chlamydiamicrovirus | 73_0 | Clustered | VC_73_0 |
| Citrobacter~phage~CF1~ERZ-2017 | Caudovirales | Myoviridae | Moonvirus | 34_7 | Clustered | VC_34_7 |
| Citrobacter~phage~CR44b | Caudovirales | Podoviridae | Teseptimavirus | 210_0 | Clustered | VC_210_0 |
| Citrobacter~phage~CR8 | Caudovirales | Podoviridae | Teseptimavirus | 210_0 | Clustered | VC_210_0 |
| Citrobacter~phage~CVT22 | Caudovirales | Podoviridae | Unassigned | 199_0 | Clustered | VC_199_0 |
| Citrobacter~phage~IME-CF2 | Caudovirales | Myoviridae | Unassigned | 37_1 | Clustered | VC_37_1 |
| Citrobacter~phage~Margaery | Caudovirales | Myoviridae | Unassigned | 37_1 | Clustered | VC_37_1 |
| Citrobacter~phage~Merlin | Caudovirales | Myoviridae | Moonvirus | 34_7 | Clustered | VC_34_7 |
| Citrobacter~phage~Michonne | Caudovirales | Myoviridae | Mooglevirus | 16_0 | Clustered | VC_16_0 |
| Citrobacter~phage~Miller | Caudovirales | Myoviridae | Unassigned | 37_1 | Clustered | VC_37_1 |
| Citrobacter~phage~Moogle | Caudovirales | Myoviridae | Mooglevirus | 16_0 | Clustered | VC_16_0 |
| Citrobacter~phage~Moon | Caudovirales | Myoviridae | Moonvirus | 34_7 | Clustered | VC_34_7 |
| Citrobacter~phage~Mordin | Caudovirales | Myoviridae | Mooglevirus | 16_0 | Clustered | VC_16_0 |
| Citrobacter~phage~SH1 | Caudovirales | Podoviridae | Teseptimavirus | 210_0 | Clustered | VC_210_0 |
| Citrobacter~phage~SH2 | Caudovirales | Podoviridae | Teseptimavirus | 210_0 | Clustered | VC_210_0 |
| Citrobacter~phage~SH3 | Caudovirales | Podoviridae | Teseptimavirus | 210_0 | Clustered | VC_210_0 |
| Citrobacter~phage~SH4 | Caudovirales | Podoviridae | Teseptimavirus | 210_0 | Clustered | VC_210_0 |
| Citrobacter~phage~phiCFP-1 | Caudovirales | Podoviridae | Teseptimavirus | 210_0 | Clustered | VC_210_0 |
| Citrobacter~phage~vB_CfrM_CfP1 | Caudovirales | Myoviridae | Unassigned | 37_1 | Clustered | VC_37_1 |
| Citrobacter~virus~Stevie | Caudovirales | Siphoviridae | Tlsvirus | 64_0 | Clustered | VC_64_0 |
| Clavibacter~phage~CMP1 | Caudovirales | Siphoviridae | Cimpunavirus | 91_0 | Clustered | VC_91_0 |
| Clavibacter~phage~CN1A | Caudovirales | Siphoviridae | Cinunavirus | 91_0 | Clustered | VC_91_0 |
| Clostridium~phage~CDMH1 | Caudovirales | Myoviridae | Lubbockvirus | 126_2 | Clustered | VC_126_2 |
| Clostridium~phage~PhiS63 | Caudovirales | Siphoviridae | Unassigned | 134_0 | Clustered | VC_134_0 |
| Clostridium~phage~phi3626 | Caudovirales | Siphoviridae | Unassigned | 134_0 | Clustered | VC_134_0 |
| Clostridium~phage~phiCD111 | Caudovirales | Siphoviridae | Unassigned | 217_0 | Clustered | VC_217_0 |
| Clostridium~phage~phiCD146 | Caudovirales | Siphoviridae | Unassigned | 217_0 | Clustered | VC_217_0 |
| Clostridium~phage~phiCD38-2 | Caudovirales | Siphoviridae | Unassigned | 217_0 | Clustered | VC_217_0 |
| Clostridium~phage~phiCD481-1 | Caudovirales | Myoviridae | Unassigned | 218_0 | Clustered | VC_218_0 |
| Clostridium~phage~phiCD505 | Caudovirales | Myoviridae | Lubbockvirus | 126_1 | Clustered | VC_126_1 |
| Clostridium~phage~phiCD506 | Caudovirales | Myoviridae | Unassigned | 218_0 | Clustered | VC_218_0 |
| Clostridium~phage~phiCDHM11 | Caudovirales | Myoviridae | Unassigned | 218_0 | Clustered | VC_218_0 |
| Clostridium~phage~phiCDHM13 | Caudovirales | Myoviridae | Unassigned | 218_0 | Clustered | VC_218_0 |
| Clostridium~phage~phiCDHM19 | Caudovirales | Myoviridae | Lubbockvirus | 126_0 | Clustered | VC_126_0 |
| Clostridium~phage~phiCP13O | Caudovirales | Siphoviridae | Unassigned | 221_0 | Clustered | VC_221_0 |
| Clostridium~phage~phiCP26F | Caudovirales | Siphoviridae | Unassigned | 221_0 | Clustered | VC_221_0 |
| Clostridium~phage~phiCP34O | Caudovirales | Siphoviridae | Unassigned | 221_0 | Clustered | VC_221_0 |
| Clostridium~phage~phiCP39-O | Caudovirales | Siphoviridae | Unassigned | 221_0 | Clustered | VC_221_0 |
| Clostridium~phage~phiCP7R | Caudovirales | Podoviridae | Unassigned | 219_0 | Clustered | VC_219_0 |
| Clostridium~phage~phiCPV4 | Caudovirales | Podoviridae | Unassigned | 219_0 | Clustered | VC_219_0 |
| Clostridium~phage~phiCT19406A | Caudovirales | Myoviridae | Unassigned | 140_0 | Clustered | VC_140_0 |
| Clostridium~phage~phiCT19406B | Caudovirales | Siphoviridae | Unassigned | 135_0 | Clustered | VC_135_0 |
| Clostridium~phage~phiCT19406C | Unassigned | Unassigned | Unassigned | 135_0 | Clustered | VC_135_0 |
| Clostridium~phage~phiCT453B | Caudovirales | Siphoviridae | Unassigned | 135_0 | Clustered | VC_135_0 |
| Clostridium~phage~phiCTC2A | Caudovirales | Myoviridae | Unassigned | 140_0 | Clustered | VC_140_0 |
| Clostridium~phage~phiCTC2B | Caudovirales | Siphoviridae | Unassigned | 135_0 | Clustered | VC_135_0 |
| Clostridium~phage~phiCTP1 | Caudovirales | Siphoviridae | Unassigned | 220_0 | Clustered | VC_220_0 |
| Clostridium~phage~phiMMP01 | Caudovirales | Myoviridae | Lubbockvirus | 126_2 | Clustered | VC_126_2 |
| Clostridium~phage~phiMMP02 | Caudovirales | Myoviridae | Lubbockvirus | 126_1 | Clustered | VC_126_1 |
| Clostridium~phage~phiMMP03 | Caudovirales | Myoviridae | Lubbockvirus | 126_2 | Clustered | VC_126_2 |
| Clostridium~phage~phiMMP04 | Caudovirales | Myoviridae | Unassigned | 218_0 | Clustered | VC_218_0 |
| Clostridium~phage~phiSM101 | Caudovirales | Siphoviridae | Unassigned | 134_0 | Clustered | VC_134_0 |
| Clostridium~phage~phiZP2 | Caudovirales | Podoviridae | Unassigned | 219_0 | Clustered | VC_219_0 |
| Clostridium~virus~phiC2 | Caudovirales | Myoviridae | Cd119virus | 126_2 | Clustered | VC_126_2 |
| Clostridium~virus~phiCD119 | Caudovirales | Myoviridae | Cd119virus | 126_0 | Clustered | VC_126_0 |
| Clostridium~virus~phiCD27 | Caudovirales | Myoviridae | Cd119virus | 126_1 | Clustered | VC_126_1 |
| Corynebacterium~phage~C3PO | Caudovirales | Siphoviridae | Ceetrepovirus | 222_0 | Clustered | VC_222_0 |
| Corynebacterium~phage~Darwin | Caudovirales | Siphoviridae | Ceetrepovirus | 222_0 | Clustered | VC_222_0 |
| Corynebacterium~phage~Poushou | Caudovirales | Siphoviridae | Poushouvirus | 224_0 | Clustered | VC_224_0 |
| Corynebacterium~phage~Zion | Caudovirales | Siphoviridae | Ceetrepovirus | 222_0 | Clustered | VC_222_0 |
| Cronobacter~phage~CR3 | Caudovirales | Myoviridae | Cr3virus | 225_0 | Clustered | VC_225_0 |
| Cronobacter~phage~CR5 | Caudovirales | Myoviridae | Unassigned | 229_0 | Clustered | VC_229_0 |
| Cronobacter~phage~CR8 | Caudovirales | Myoviridae | Cr3virus | 225_0 | Clustered | VC_225_0 |
| Cronobacter~phage~CR9 | Caudovirales | Myoviridae | Cr3virus | 225_0 | Clustered | VC_225_0 |
| Cronobacter~phage~Dev-CD-23823 | Caudovirales | Podoviridae | Unassigned | 68_0 | Clustered | VC_68_0 |
| Cronobacter~phage~Dev2 | Caudovirales | Podoviridae | Teseptimavirus | 210_0 | Clustered | VC_210_0 |
| Cronobacter~phage~PBES~02 | Caudovirales | Myoviridae | Cr3virus | 225_0 | Clustered | VC_225_0 |
| Cronobacter~phage~S13 | Caudovirales | Myoviridae | Unassigned | 36_2 | Clustered/Singleton | VC_36_2 |
| Cronobacter~phage~vB_CsaM_GAP161 | Caudovirales | Myoviridae | Unassigned | 37_1 | Clustered | VC_37_1 |
| Cronobacter~phage~vB_CsaM_GAP31 | Caudovirales | Myoviridae | Se1virus | 226_2 | Clustered | VC_226_2 |
| Cronobacter~phage~vB_CsaM_GAP32 | Caudovirales | Myoviridae | Eneladusvirus | 72_0 | Clustered/Singleton | VC_72_0 |
| Cronobacter~phage~vB_CsaP_GAP52 | Caudovirales | Podoviridae | Unassigned | 235_0 | Clustered | VC_235_0 |
| Cronobacter~phage~vB_CskP_GAP227 | Caudovirales | Podoviridae | Unassigned | 68_0 | Clustered | VC_68_0 |
| Cronobacter~virus~Esp2949-1 | Caudovirales | Siphoviridae | Unassigned | 64_0 | Clustered | VC_64_0 |
| Cyanophage~9515-10a | Caudovirales | Podoviridae | Unassigned | 211_0 | Clustered | VC_211_0 |
| Cyanophage~KBS-P-1A | Caudovirales | Podoviridae | Unassigned | 211_0 | Clustered | VC_211_0 |
| Cyanophage~MED4-117 | Unassigned | Unassigned | Unassigned | 237_0 | Clustered | VC_237_0 |
| Cyanophage~NATL1A-7 | Caudovirales | Podoviridae | Unassigned | 211_0 | Clustered | VC_211_0 |
| Cyanophage~NATL2A-133 | Caudovirales | Podoviridae | Unassigned | 211_0 | Clustered | VC_211_0 |
| Cyanophage~P-RSM1 | Caudovirales | Myoviridae | Unassigned | 25_9 | Clustered | VC_25_9 |
| Cyanophage~P-RSM6 | Caudovirales | Myoviridae | Unassigned | 25_11 | Clustered/Singleton | VC_25_11 |
| Cyanophage~P-SSP2 | Caudovirales | Podoviridae | Unassigned | 211_0 | Clustered | VC_211_0 |
| Cyanophage~P-TIM40 | Caudovirales | Myoviridae | Unassigned | 25_22 | Clustered/Singleton | VC_25_22 |
| Cyanophage~PP | Caudovirales | Podoviridae | Unassigned | 80_0 | Clustered | VC_80_0 |
| Cyanophage~PSS2 | Caudovirales | Siphoviridae | Unassigned | 239_0 | Clustered | VC_239_0 |
| Cyanophage~S-RIM32 | Caudovirales | Myoviridae | Unassigned | 25_14 | Clustered/Singleton | VC_25_14 |
| Cyanophage~S-RIM50 | Caudovirales | Myoviridae | Unassigned | 25_4 | Clustered | VC_25_4 |
| Cyanophage~SS120-1 | Caudovirales | Podoviridae | Unassigned | 211_0 | Clustered | VC_211_0 |
| Cyanophage~Syn30 | Caudovirales | Myoviridae | Unassigned | 25_33 | Clustered/Singleton | VC_25_33 |
| Deep-sea~thermophilic~phage~D6E | Unassigned | Unassigned | Unassigned | 132_0 | Clustered | VC_132_0 |
| Delftia~phage~IME-DE1 | Caudovirales | Podoviridae | Teseptimavirus | 210_0 | Clustered | VC_210_0 |
| Delftia~phage~RG-2014 | Caudovirales | Podoviridae | Unassigned | 7_1 | Clustered | VC_7_1 |
| Dickeya~phage~RC-2014 | Caudovirales | Ackermannviridae | Limestonevirus | 26_1 | Clustered | VC_26_1 |
| Dickeya~virus~Limestone | Caudovirales | Ackermannviridae | Limestonevirus | 26_1 | Clustered | VC_26_1 |
| Ecterococcus~phage~vB_EfaS_AL3 | Caudovirales | Siphoviridae | Efquatrovirus | 242_0 | Clustered | VC_242_0 |
| Edwardsiella~phage~KF-1 | Caudovirales | Podoviridae | Kf1virus | 243_0 | Clustered | VC_243_0 |
| Edwardsiella~phage~MSW-3 | Caudovirales | Myoviridae | Msw3virus | 65_0 | Clustered | VC_65_0 |
| Edwardsiella~phage~PEi20 | Caudovirales | Myoviridae | Unassigned | 34_2 | Clustered/Singleton | VC_34_2 |
| Edwardsiella~phage~PEi21 | Caudovirales | Myoviridae | Msw3virus | 65_0 | Clustered | VC_65_0 |
| Edwardsiella~phage~eiAU | Caudovirales | Siphoviridae | Eiauvirus | 194_0 | Clustered | VC_194_0 |
| Edwardsiella~phage~eiAU-183 | Caudovirales | Siphoviridae | Eiauvirus | 194_0 | Clustered | VC_194_0 |
| Enterobacter~phage~Arya | Caudovirales | Myoviridae | Jilinvirus | 62_0 | Clustered | VC_62_0 |
| Enterobacter~phage~CC31 | Caudovirales | Myoviridae | Cc31virus | 34_8 | Clustered | VC_34_8 |
| Enterobacter~phage~E-2 | Caudovirales | Podoviridae | Teseptimavirus | 210_0 | Clustered | VC_210_0 |
| Enterobacter~phage~E-3 | Caudovirales | Podoviridae | Teseptimavirus | 210_0 | Clustered | VC_210_0 |
| Enterobacter~phage~EcP1 | Caudovirales | Podoviridae | Unassigned | 11_0 | Clustered/Singleton | VC_11_0 |
| Enterobacter~phage~Ec_L1 | Caudovirales | Siphoviridae | Eclunavirus | 64_0 | Clustered | VC_64_0 |
| Enterobacter~phage~Enc34 | Caudovirales | Siphoviridae | Chivirus | 15_0 | Clustered | VC_15_0 |
| Enterobacter~phage~PG7 | Caudovirales | Myoviridae | Cc31virus | 34_8 | Clustered | VC_34_8 |
| Enterobacter~phage~Tyrion | Caudovirales | Podoviridae | Uetakevirus | 168_0 | Clustered | VC_168_0 |
| Enterobacter~phage~phiEap-1 | Caudovirales | Podoviridae | Przondovirus | 210_0 | Clustered | VC_210_0 |
| Enterobacter~phage~phiEap-2 | Caudovirales | Siphoviridae | Cornellvirus | 50_0 | Clustered | VC_50_0 |
| Enterobacter~phage~phiEap-3 | Caudovirales | Myoviridae | Kp15virus | 37_0 | Clustered | VC_37_0 |
| Enterobacteria~phage~13a | Caudovirales | Podoviridae | Teseptimavirus | 210_0 | Clustered | VC_210_0 |
| Enterobacteria~phage~285P | Caudovirales | Podoviridae | Teseptimavirus | 210_0 | Clustered | VC_210_0 |
| Enterobacteria~phage~933W | Caudovirales | Podoviridae | Nona33virus | 192_1 | Clustered | VC_192_1 |
| Enterobacteria~phage~9g | Caudovirales | Siphoviridae | Nonagvirus | 14_0 | Clustered | VC_14_0 |
| Enterobacteria~phage~BA14 | Caudovirales | Podoviridae | Teseptimavirus | 210_0 | Clustered | VC_210_0 |
| Enterobacteria~phage~Bp7 | Caudovirales | Myoviridae | Js98virus | 34_1 | Clustered | VC_34_1 |
| Enterobacteria~phage~C-1~INW-2012 | Unassigned | Leviviridae | Levivirus | 197_0 | Clustered | VC_197_0 |
| Enterobacteria~phage~EcoDS1 | Caudovirales | Podoviridae | Teseptimavirus | 210_0 | Clustered | VC_210_0 |
| Enterobacteria~phage~G4 | Unassigned | Microviridae | G4microvirus | 246_0 | Clustered | VC_246_0 |
| Enterobacteria~phage~GEC-3S | Caudovirales | Myoviridae | Krischvirus | 38_0 | Clustered | VC_38_0 |
| Enterobacteria~phage~HK106 | Caudovirales | Siphoviridae | Hk97virus | 176_0 | Clustered | VC_176_0 |
| Enterobacteria~phage~HK140 | Caudovirales | Siphoviridae | Hendrixvirus | 176_0 | Clustered | VC_176_0 |
| Enterobacteria~phage~Hgal1 | Unassigned | Leviviridae | Levivirus | 197_0 | Clustered | VC_197_0 |
| Enterobacteria~phage~ID18~sensu~lato | Unassigned | Microviridae | G4microvirus | 246_0 | Clustered | VC_246_0 |
| Enterobacteria~phage~ID2~Moscow/ID/2001 | Unassigned | Microviridae | G4microvirus | 246_0 | Clustered | VC_246_0 |
| Enterobacteria~phage~IME08 | Caudovirales | Myoviridae | Js98virus | 34_1 | Clustered | VC_34_1 |
| Enterobacteria~phage~If1 | Unassigned | Inoviridae | Unassigned | 252_0 | Clustered | VC_252_0 |
| Enterobacteria~phage~JS10 | Caudovirales | Myoviridae | Js98virus | 34_1 | Clustered | VC_34_1 |
| Enterobacteria~phage~JenK1 | Caudovirales | Siphoviridae | Nonagvirus | 14_0 | Clustered | VC_14_0 |
| Enterobacteria~phage~JenP1 | Caudovirales | Siphoviridae | Nonagvirus | 14_0 | Clustered | VC_14_0 |
| Enterobacteria~phage~JenP2 | Caudovirales | Siphoviridae | Nonagvirus | 14_0 | Clustered | VC_14_0 |
| Enterobacteria~phage~K1F | Caudovirales | Podoviridae | Teseptimavirus | 210_0 | Clustered | VC_210_0 |
| Enterobacteria~phage~M | Unassigned | Leviviridae | Levivirus | 197_0 | Clustered | VC_197_0 |
| Enterobacteria~phage~P88 | Caudovirales | Myoviridae | Peduovirus | 69_0 | Clustered | VC_69_0 |
| Enterobacteria~phage~PR4 | Unassigned | Tectiviridae | Alphatectivirus | 253_0 | Clustered | VC_253_0 |
| Enterobacteria~phage~PRD1 | Unassigned | Tectiviridae | Alphatectivirus | 253_0 | Clustered | VC_253_0 |
| Enterobacteria~phage~Phi1 | Caudovirales | Myoviridae | Rb49virus | 38_0 | Clustered | VC_38_0 |
| Enterobacteria~phage~RB27 | Caudovirales | Myoviridae | Tequatrovirus | 34_5 | Clustered | VC_34_5 |
| Enterobacteria~phage~RB49 | Caudovirales | Myoviridae | Rb49virus | 38_0 | Clustered | VC_38_0 |
| Enterobacteria~phage~RB51 | Caudovirales | Myoviridae | Tequatrovirus | 34_5 | Clustered | VC_34_5 |
| Enterobacteria~phage~RB68 | Caudovirales | Myoviridae | Tequatrovirus | 34_5 | Clustered | VC_34_5 |
| Enterobacteria~phage~RB69 | Caudovirales | Myoviridae | Rb69virus | 34_0 | Clustered | VC_34_0 |
| Enterobacteria~phage~SP | Unassigned | Leviviridae | Allolevivirus | 33_0 | Clustered | VC_33_0 |
| Enterobacteria~phage~ST104 | Caudovirales | Podoviridae | Lederbergvirus | 163_0 | Clustered | VC_163_0 |
| Enterobacteria~phage~Sf101 | Caudovirales | Podoviridae | Lederbergvirus | 163_0 | Clustered | VC_163_0 |
| Enterobacteria~phage~SfV | Caudovirales | Myoviridae | Unassigned | 106_0 | Clustered | VC_106_0 |
| Enterobacteria~phage~St-1 | Unassigned | Microviridae | Alpha3microvirus | 246_0 | Clustered | VC_246_0 |
| Enterobacteria~phage~T3 | Caudovirales | Podoviridae | Teseptimavirus | 210_0 | Clustered | VC_210_0 |
| Enterobacteria~phage~T7 | Caudovirales | Podoviridae | T7virus | 210_0 | Clustered | VC_210_0 |
| Enterobacteria~phage~UAB_Phi20 | Caudovirales | Podoviridae | Lederbergvirus | 163_0 | Clustered | VC_163_0 |
| Enterobacteria~phage~UAB_Phi78 | Caudovirales | Podoviridae | Zindervirus | 254_0 | Clustered | VC_254_0 |
| Enterobacteria~phage~VT2-Sakai | Caudovirales | Podoviridae | Traversvirus | 192_1 | Clustered | VC_192_1 |
| Enterobacteria~phage~VT2phi_272 | Caudovirales | Podoviridae | Oslovirus | 192_1 | Clustered | VC_192_1 |
| Enterobacteria~phage~WA13~sensu~lato | Unassigned | Microviridae | Alphatrevirus | 246_0 | Clustered | VC_246_0 |
| Enterobacteria~phage~alpha3 | Unassigned | Microviridae | Alpha3microvirus | 246_0 | Clustered | VC_246_0 |
| Enterobacteria~phage~fd | Unassigned | Inoviridae | Inovirus | 252_0 | Clustered | VC_252_0 |
| Enterobacteria~phage~fiAA91-ss | Caudovirales | Myoviridae | Peduovirus | 69_0 | Clustered | VC_69_0 |
| Enterobacteria~phage~mEp235 | Caudovirales | Siphoviridae | Hendrixvirus | 176_0 | Clustered | VC_176_0 |
| Enterobacteria~phage~phi92 | Caudovirales | Myoviridae | Unassigned | 49_0 | Clustered | VC_49_0 |
| Enterobacteria~phage~phiEcoM-GJ1 | Caudovirales | Myoviridae | Unassigned | 59_0 | Clustered | VC_59_0 |
| Enterobacteria~phage~phiP27 | Caudovirales | Myoviridae | Unassigned | 106_0 | Clustered | VC_106_0 |
| Enterobacteria~phage~vB_EcoM_VR5 | Caudovirales | Myoviridae | Js98virus | 34_1 | Clustered | VC_34_1 |
| Enterobacteria~phage~vB_EcoP_ACG-C91 | Caudovirales | Podoviridae | Zindervirus | 254_0 | Clustered | VC_254_0 |
| Enterobacteria~phage~vB_EcoS_NBD2 | Caudovirales | Siphoviridae | Eclunavirus | 64_0 | Clustered | VC_64_0 |
| Enterobacteria~phage~vB_EcoS_Rogue1 | Caudovirales | Siphoviridae | Rogue1virus | 64_0 | Clustered | VC_64_0 |
| Enterobacteria~phage~vB_KleM-RaK2 | Caudovirales | Myoviridae | Alcyoneusvirus | 72_5 | Clustered/Singleton | VC_72_5 |
| Enterobacteriaphage~UAB_Phi87 | Caudovirales | Myoviridae | Felixo1virus | 16_0 | Clustered | VC_16_0 |
| Enterococcus~phage~AUEF3 | Caudovirales | Siphoviridae | Efquatrovirus | 242_0 | Clustered | VC_242_0 |
| Enterococcus~phage~BC611 | Caudovirales | Siphoviridae | Sap6virus | 138_0 | Clustered | VC_138_0 |
| Enterococcus~phage~Ec-ZZ2 | Caudovirales | Siphoviridae | Efquatrovirus | 242_0 | Clustered | VC_242_0 |
| Enterococcus~phage~EfaCPT1 | Caudovirales | Siphoviridae | Efquatrovirus | 242_0 | Clustered | VC_242_0 |
| Enterococcus~phage~IME-EF4 | Caudovirales | Siphoviridae | Efquatrovirus | 242_0 | Clustered | VC_242_0 |
| Enterococcus~phage~IMEEF1 | Caudovirales | Siphoviridae | Sap6virus | 138_0 | Clustered | VC_138_0 |
| Enterococcus~phage~IME_EF3 | Caudovirales | Siphoviridae | Efquatrovirus | 242_0 | Clustered | VC_242_0 |
| Enterococcus~phage~LY0322 | Caudovirales | Siphoviridae | Efquatrovirus | 242_0 | Clustered | VC_242_0 |
| Enterococcus~phage~PMBT2 | Caudovirales | Siphoviridae | Efquatrovirus | 242_0 | Clustered | VC_242_0 |
| Enterococcus~phage~SANTOR1 | Caudovirales | Siphoviridae | Efquatrovirus | 242_0 | Clustered | VC_242_0 |
| Enterococcus~phage~SAP6 | Caudovirales | Siphoviridae | Sap6virus | 138_0 | Clustered | VC_138_0 |
| Enterococcus~phage~VD13 | Caudovirales | Siphoviridae | Sap6virus | 138_0 | Clustered | VC_138_0 |
| Enterococcus~phage~phiFL1A | Caudovirales | Siphoviridae | Phifelvirus | 171_0 | Clustered | VC_171_0 |
| Enterococcus~phage~phiFL2A | Caudovirales | Siphoviridae | Phifelvirus | 171_0 | Clustered | VC_171_0 |
| Enterococcus~phage~phiFL3A | Caudovirales | Siphoviridae | Phifelvirus | 171_0 | Clustered | VC_171_0 |
| Enterococcus~phage~phiSHEF2 | Caudovirales | Siphoviridae | Efquatrovirus | 242_0 | Clustered | VC_242_0 |
| Enterococcus~phage~phiSHEF4 | Caudovirales | Siphoviridae | Efquatrovirus | 242_0 | Clustered | VC_242_0 |
| Enterococcus~phage~phiSHEF5 | Caudovirales | Siphoviridae | Efquatrovirus | 242_0 | Clustered | VC_242_0 |
| Enterococcus~phage~vB_EfaP_IME195 | Caudovirales | Podoviridae | Unassigned | 257_0 | Clustered | VC_257_0 |
| Enterococcus~phage~vB_EfaS_AL2 | Caudovirales | Siphoviridae | Efquatrovirus | 242_0 | Clustered | VC_242_0 |
| Enterococcus~phage~vB_EfaS_IME196 | Caudovirales | Siphoviridae | Efquatrovirus | 242_0 | Clustered | VC_242_0 |
| Enterococcus~phage~vB_EfaS_IME198 | Caudovirales | Siphoviridae | Saphexavirus | 138_0 | Clustered | VC_138_0 |
| Enterococcus~phage~vB_Efae230P-4 | Caudovirales | Podoviridae | Unassigned | 257_0 | Clustered | VC_257_0 |
| Erwinia~amylovora~phage~Era103 | Caudovirales | Podoviridae | Sp6virus | 254_0 | Clustered | VC_254_0 |
| Erwinia~phage~ENT90 | Caudovirales | Myoviridae | Peduovirus | 69_0 | Clustered | VC_69_0 |
| Erwinia~phage~Ea35-70 | Caudovirales | Myoviridae | Agrican357virus | 228_0 | Clustered | VC_228_0 |
| Erwinia~phage~Ea9-2 | Caudovirales | Podoviridae | Ea92virus | 7_0 | Clustered | VC_7_0 |
| Erwinia~phage~FE44 | Caudovirales | Podoviridae | Teseptimavirus | 210_0 | Clustered | VC_210_0 |
| Erwinia~phage~phiEa100 | Caudovirales | Podoviridae | Zindervirus | 254_0 | Clustered | VC_254_0 |
| Erwinia~phage~phiEa104 | Caudovirales | Myoviridae | Ea214virus | 214_0 | Clustered | VC_214_0 |
| Erwinia~phage~phiEa21-4 | Caudovirales | Myoviridae | Ea214virus | 214_0 | Clustered | VC_214_0 |
| Erwinia~phage~phiEa2809 | Caudovirales | Ackermannviridae | Unassigned | 26_0 | Clustered | VC_26_0 |
| Erwinia~phage~phiEaH2 | Caudovirales | Myoviridae | Eah2virus | 229_1 | Clustered | VC_229_1 |
| Erwinia~phage~phiEt88 | Caudovirales | Myoviridae | Unassigned | 216_0 | Clustered | VC_216_0 |
| Erwinia~phage~vB_Eam-MM7 | Caudovirales | Myoviridae | Ea214virus | 214_0 | Clustered | VC_214_0 |
| Erwinia~phage~vB_EamM-Y2 | Caudovirales | Myoviridae | Unassigned | 59_0 | Clustered | VC_59_0 |
| Erwinia~phage~vB_EamM_Asesino | Caudovirales | Myoviridae | Eah2virus | 229_1 | Clustered | VC_229_1 |
| Erwinia~phage~vB_EamM_Caitlin | Caudovirales | Myoviridae | Machinavirus | 229_3 | Clustered | VC_229_3 |
| Erwinia~phage~vB_EamM_ChrisDB | Caudovirales | Myoviridae | Machinavirus | 229_3 | Clustered | VC_229_3 |
| Erwinia~phage~vB_EamM_Deimos-Minion | Caudovirales | Myoviridae | Agrican357virus | 228_0 | Clustered | VC_228_0 |
| Erwinia~phage~vB_EamM_Desertfox | Caudovirales | Myoviridae | Agricanvirus | 228_0 | Clustered | VC_228_0 |
| Erwinia~phage~vB_EamM_EarlPhillipIV | Caudovirales | Myoviridae | Unassigned | 229_0 | Clustered | VC_229_0 |
| Erwinia~phage~vB_EamM_Huxley | Caudovirales | Myoviridae | Machinavirus | 229_2 | Clustered | VC_229_2 |
| Erwinia~phage~vB_EamM_Kwan | Caudovirales | Myoviridae | Unassigned | 229_4 | Clustered/Singleton | VC_229_4 |
| Erwinia~phage~vB_EamM_Machina | Caudovirales | Myoviridae | Machinavirus | 229_2 | Clustered | VC_229_2 |
| Erwinia~phage~vB_EamM_Phobos | Caudovirales | Myoviridae | Unassigned | 229_0 | Clustered | VC_229_0 |
| Erwinia~phage~vB_EamM_RAY | Caudovirales | Myoviridae | Agrican357virus | 228_0 | Clustered | VC_228_0 |
| Erwinia~phage~vB_EamM_RisingSun | Caudovirales | Myoviridae | Risingsunvirus | 231_1 | Clustered | VC_231_1 |
| Erwinia~phage~vB_EamM_Simmy50 | Caudovirales | Myoviridae | Agrican357virus | 228_0 | Clustered | VC_228_0 |
| Erwinia~phage~vB_EamM_Special~G | Caudovirales | Myoviridae | Agrican357virus | 228_0 | Clustered | VC_228_0 |
| Erwinia~phage~vB_EamP-L1 | Caudovirales | Podoviridae | Przondovirus | 210_0 | Clustered | VC_210_0 |
| Erwinia~phage~vB_EamP-S6 | Caudovirales | Podoviridae | Unassigned | 7_1 | Clustered | VC_7_1 |
| Erwinia~phage~vB_EamP_Frozen | Caudovirales | Podoviridae | Ea92virus | 7_0 | Clustered | VC_7_0 |
| Escherichia~Stx1~converting~phage | Caudovirales | Podoviridae | Traversvirus | 192_0 | Clustered | VC_192_0 |
| Escherichia~coli~O157~typing~phage~1 | Caudovirales | Myoviridae | Felixo1virus | 16_0 | Clustered | VC_16_0 |
| Escherichia~coli~O157~typing~phage~3 | Caudovirales | Myoviridae | Mosigvirus | 34_0 | Clustered | VC_34_0 |
| Escherichia~coli~O157~typing~phage~6 | Caudovirales | Myoviridae | Mosigvirus | 34_0 | Clustered | VC_34_0 |
| Escherichia~phage~121Q | Caudovirales | Myoviridae | Asteriusvirus | 72_4 | Clustered | VC_72_4 |
| Escherichia~phage~172-1 | Caudovirales | Podoviridae | Phieco32virus | 212_0 | Clustered | VC_212_0 |
| Escherichia~phage~4MG | Caudovirales | Myoviridae | Se1virus | 226_2 | Clustered | VC_226_2 |
| Escherichia~phage~64795_ec1 | Caudovirales | Podoviridae | Teseptimavirus | 210_0 | Clustered | VC_210_0 |
| Escherichia~phage~ADB-2 | Caudovirales | Siphoviridae | T1virus | 64_0 | Clustered | VC_64_0 |
| Escherichia~phage~APCEc01 | Caudovirales | Myoviridae | Mosigvirus | 34_0 | Clustered | VC_34_0 |
| Escherichia~phage~APCEc02 | Caudovirales | Myoviridae | Vequintavirus | 226_0 | Clustered | VC_226_0 |
| Escherichia~phage~AR1 | Caudovirales | Myoviridae | T4virus | 34_5 | Clustered | VC_34_5 |
| Escherichia~phage~Av-05 | Caudovirales | Myoviridae | Avunavirus | 226_4 | Clustered/Singleton | VC_226_4 |
| Escherichia~phage~Bp4 | Caudovirales | Podoviridae | G7cvirus | 7_1 | Clustered | VC_7_1 |
| Escherichia~phage~C119 | Caudovirales | Siphoviridae | Rogue1virus | 64_0 | Clustered | VC_64_0 |
| Escherichia~phage~CAjan | Caudovirales | Siphoviridae | Seuratvirus | 14_1 | Clustered | VC_14_1 |
| Escherichia~phage~CICC~80001 | Caudovirales | Podoviridae | Teseptimavirus | 210_0 | Clustered | VC_210_0 |
| Escherichia~phage~D108 | Caudovirales | Myoviridae | Muvirus | 188_0 | Clustered | VC_188_0 |
| Escherichia~phage~EB49 | Caudovirales | Siphoviridae | Rogue1virus | 64_0 | Clustered | VC_64_0 |
| Escherichia~phage~EC1-UPM | Caudovirales | Podoviridae | G7cvirus | 7_1 | Clustered | VC_7_1 |
| Escherichia~phage~EC6 | Caudovirales | Myoviridae | Felixo1virus | 16_0 | Clustered | VC_16_0 |
| Escherichia~phage~ECBP1 | Caudovirales | Podoviridae | G7cvirus | 7_1 | Clustered | VC_7_1 |
| Escherichia~phage~ECBP2 | Caudovirales | Podoviridae | Phieco32virus | 212_0 | Clustered | VC_212_0 |
| Escherichia~phage~ECBP5 | Caudovirales | Podoviridae | Zindervirus | 254_0 | Clustered | VC_254_0 |
| Escherichia~phage~ECD7 | Caudovirales | Myoviridae | Krischvirus | 38_0 | Clustered | VC_38_0 |
| Escherichia~phage~ECML-134 | Caudovirales | Myoviridae | T4virus | 34_5 | Clustered | VC_34_5 |
| Escherichia~phage~ECML-4 | Caudovirales | Ackermannviridae | Vi1virus | 26_1 | Clustered | VC_26_1 |
| Escherichia~phage~EK99P-1 | Caudovirales | Siphoviridae | Hk578virus | 233_0 | Clustered | VC_233_0 |
| Escherichia~phage~Envy | Caudovirales | Siphoviridae | Dhillonvirus | 233_0 | Clustered | VC_233_0 |
| Escherichia~phage~FFH2 | Caudovirales | Myoviridae | V5virus | 226_0 | Clustered | VC_226_0 |
| Escherichia~phage~FV3 | Caudovirales | Myoviridae | V5virus | 226_0 | Clustered | VC_226_0 |
| Escherichia~phage~Gluttony | Caudovirales | Siphoviridae | Dhillonvirus | 233_0 | Clustered | VC_233_0 |
| Escherichia~phage~HK446 | Caudovirales | Siphoviridae | Hk97virus | 176_0 | Clustered | VC_176_0 |
| Escherichia~phage~HK542 | Caudovirales | Siphoviridae | Hk97virus | 176_0 | Clustered | VC_176_0 |
| Escherichia~phage~HK544 | Caudovirales | Siphoviridae | Hk97virus | 176_0 | Clustered | VC_176_0 |
| Escherichia~phage~HK578 | Caudovirales | Siphoviridae | Hk578virus | 233_0 | Clustered | VC_233_0 |
| Escherichia~phage~HK633 | Caudovirales | Siphoviridae | Hk97virus | 176_0 | Clustered | VC_176_0 |
| Escherichia~phage~HK75 | Caudovirales | Siphoviridae | Hk97virus | 176_0 | Clustered | VC_176_0 |
| Escherichia~phage~HY01 | Caudovirales | Myoviridae | T4virus | 34_5 | Clustered | VC_34_5 |
| Escherichia~phage~HY02 | Caudovirales | Myoviridae | Felixo1virus | 16_0 | Clustered | VC_16_0 |
| Escherichia~phage~HY03 | Caudovirales | Myoviridae | Tequatrovirus | 34_5 | Clustered | VC_34_5 |
| Escherichia~phage~IME11 | Caudovirales | Podoviridae | G7cvirus | 7_1 | Clustered | VC_7_1 |
| Escherichia~phage~JES2013 | Caudovirales | Myoviridae | V5virus | 226_0 | Clustered | VC_226_0 |
| Escherichia~phage~JH2 | Caudovirales | Myoviridae | Felixo1virus | 16_0 | Clustered | VC_16_0 |
| Escherichia~phage~JMPW1 | Caudovirales | Siphoviridae | T1virus | 64_0 | Clustered | VC_64_0 |
| Escherichia~phage~JMPW2 | Caudovirales | Siphoviridae | T1virus | 64_0 | Clustered | VC_64_0 |
| Escherichia~phage~JS98 | Caudovirales | Myoviridae | Js98virus | 34_1 | Clustered | VC_34_1 |
| Escherichia~phage~JSE | Caudovirales | Myoviridae | Rb49virus | 38_0 | Clustered | VC_38_0 |
| Escherichia~phage~K1-dep(1) | Caudovirales | Siphoviridae | K1gvirus | 50_0 | Clustered | VC_50_0 |
| Escherichia~phage~K1-dep(4) | Caudovirales | Siphoviridae | K1gvirus | 50_0 | Clustered | VC_50_0 |
| Escherichia~phage~K1-ind(2) | Caudovirales | Siphoviridae | K1gvirus | 50_0 | Clustered | VC_50_0 |
| Escherichia~phage~K1ind1 | Caudovirales | Siphoviridae | K1gvirus | 50_0 | Clustered | VC_50_0 |
| Escherichia~phage~K30 | Caudovirales | Podoviridae | Kp32virus | 210_0 | Clustered | VC_210_0 |
| Escherichia~phage~KBNP1711 | Caudovirales | Podoviridae | Phieco32virus | 212_0 | Clustered | VC_212_0 |
| Escherichia~phage~LM33_P1 | Caudovirales | Podoviridae | Teseptimavirus | 210_0 | Clustered | VC_210_0 |
| Escherichia~phage~Lw1 | Caudovirales | Myoviridae | Unassigned | 37_1 | Clustered | VC_37_1 |
| Escherichia~phage~MX01 | Caudovirales | Myoviridae | Dhakavirus | 34_1 | Clustered | VC_34_1 |
| Escherichia~phage~Min27 | Caudovirales | Podoviridae | Nona33virus | 192_1 | Clustered | VC_192_1 |
| Escherichia~phage~Murica | Caudovirales | Myoviridae | Vequintavirus | 226_0 | Clustered | VC_226_0 |
| Escherichia~phage~NJ01 | Caudovirales | Podoviridae | Phieco32virus | 212_0 | Clustered | VC_212_0 |
| Escherichia~phage~P13374 | Caudovirales | Podoviridae | Oslovirus | 192_1 | Clustered | VC_192_1 |
| Escherichia~phage~P483 | Caudovirales | Podoviridae | Teseptimavirus | 210_0 | Clustered | VC_210_0 |
| Escherichia~phage~P694 | Caudovirales | Podoviridae | Teseptimavirus | 210_0 | Clustered | VC_210_0 |
| Escherichia~phage~PA2 | Caudovirales | Podoviridae | Tl2011virus | 192_1 | Clustered | VC_192_1 |
| Escherichia~phage~PA28 | Caudovirales | Podoviridae | Nona33virus | 192_1 | Clustered | VC_192_1 |
| Escherichia~phage~PBECO~4 | Caudovirales | Myoviridae | Asteriusvirus | 72_4 | Clustered | VC_72_4 |
| Escherichia~phage~PE3-1 | Caudovirales | Podoviridae | Teseptimavirus | 210_0 | Clustered | VC_210_0 |
| Escherichia~phage~PhaxI | Caudovirales | Ackermannviridae | Cba120virus | 26_1 | Clustered | VC_26_1 |
| Escherichia~phage~QL01 | Caudovirales | Myoviridae | Js98virus | 34_1 | Clustered | VC_34_1 |
| Escherichia~phage~RB3 | Caudovirales | Myoviridae | T4virus | 34_5 | Clustered | VC_34_5 |
| Escherichia~phage~RCS47 | Caudovirales | Myoviridae | Punavirus | 260_0 | Clustered | VC_260_0 |
| Escherichia~phage~SRT8 | Caudovirales | Siphoviridae | Sertoctavirus | 64_0 | Clustered | VC_64_0 |
| Escherichia~phage~ST0 | Caudovirales | Myoviridae | Mosigvirus | 34_0 | Clustered | VC_34_0 |
| Escherichia~phage~SUSP1 | Caudovirales | Myoviridae | Suspvirus | 16_0 | Clustered | VC_16_0 |
| Escherichia~phage~SUSP2 | Caudovirales | Myoviridae | Suspvirus | 16_0 | Clustered | VC_16_0 |
| Escherichia~phage~Seurat | Caudovirales | Siphoviridae | Seuratvirus | 14_1 | Clustered | VC_14_1 |
| Escherichia~phage~Stx2~II | Caudovirales | Podoviridae | Nona33virus | 192_0 | Clustered | VC_192_0 |
| Escherichia~phage~TL-2011b | Caudovirales | Podoviridae | Uetakevirus | 168_0 | Clustered | VC_168_0 |
| Escherichia~phage~TL-2011c | Caudovirales | Podoviridae | Tl2011virus | 192_1 | Clustered | VC_192_1 |
| Escherichia~phage~UFV-AREG1 | Caudovirales | Myoviridae | Tequatrovirus | 34_5 | Clustered | VC_34_5 |
| Escherichia~phage~V18 | Caudovirales | Myoviridae | Vequintavirus | 226_0 | Clustered | VC_226_0 |
| Escherichia~phage~V5 | Caudovirales | Myoviridae | V5virus | 226_0 | Clustered | VC_226_0 |
| Escherichia~phage~VB_EcoS-Golestan | Caudovirales | Siphoviridae | Kagunavirus | 50_0 | Clustered | VC_50_0 |
| Escherichia~phage~WG01 | Caudovirales | Myoviridae | Dhakavirus | 34_1 | Clustered | VC_34_1 |
| Escherichia~phage~YD-2008.s | Caudovirales | Siphoviridae | Hk578virus | 233_0 | Clustered | VC_233_0 |
| Escherichia~phage~e4/1c | Caudovirales | Siphoviridae | Rogue1virus | 64_0 | Clustered | VC_64_0 |
| Escherichia~phage~ime09 | Caudovirales | Myoviridae | T4virus | 34_5 | Clustered | VC_34_5 |
| Escherichia~phage~mEp234 | Caudovirales | Siphoviridae | Hk97virus | 176_0 | Clustered | VC_176_0 |
| Escherichia~phage~mEpX1 | Caudovirales | Siphoviridae | Hk97virus | 176_0 | Clustered | VC_176_0 |
| Escherichia~phage~mEpX2 | Caudovirales | Siphoviridae | Hk97virus | 176_0 | Clustered | VC_176_0 |
| Escherichia~phage~phAPEC8 | Caudovirales | Myoviridae | Unassigned | 49_0 | Clustered | VC_49_0 |
| Escherichia~phage~phi191 | Caudovirales | Podoviridae | Tl2011virus | 192_1 | Clustered | VC_192_1 |
| Escherichia~phage~phiK | Unassigned | Microviridae | Alpha3microvirus | 246_0 | Clustered | VC_246_0 |
| Escherichia~phage~phiKT | Caudovirales | Podoviridae | Unassigned | 67_0 | Clustered | VC_67_0 |
| Escherichia~phage~phiV10 | Caudovirales | Podoviridae | Epsilon15virus | 168_0 | Clustered | VC_168_0 |
| Escherichia~phage~pro147 | Caudovirales | Myoviridae | Peduovirus | 69_0 | Clustered | VC_69_0 |
| Escherichia~phage~pro483 | Caudovirales | Myoviridae | Peduovirus | 69_0 | Clustered | VC_69_0 |
| Escherichia~phage~slur01 | Caudovirales | Siphoviridae | Seuratvirus | 14_1 | Clustered | VC_14_1 |
| Escherichia~phage~slur02 | Caudovirales | Myoviridae | Tequatrovirus | 34_5 | Clustered | VC_34_5 |
| Escherichia~phage~slur03 | Caudovirales | Myoviridae | Tequatrovirus | 34_5 | Clustered | VC_34_5 |
| Escherichia~phage~slur04 | Caudovirales | Myoviridae | Tequatrovirus | 34_5 | Clustered | VC_34_5 |
| Escherichia~phage~slur05 | Caudovirales | Siphoviridae | Dhillonvirus | 233_0 | Clustered | VC_233_0 |
| Escherichia~phage~slur07 | Caudovirales | Myoviridae | Tequatrovirus | 34_5 | Clustered | VC_34_5 |
| Escherichia~phage~slur09 | Caudovirales | Siphoviridae | T5virus | 261_1 | Clustered | VC_261_1 |
| Escherichia~phage~slur14 | Caudovirales | Myoviridae | Tequatrovirus | 34_5 | Clustered | VC_34_5 |
| Escherichia~phage~slur16 | Caudovirales | Myoviridae | Vequintavirus | 226_0 | Clustered | VC_226_0 |
| Escherichia~phage~vB_EcoM-UFV13 | Caudovirales | Myoviridae | Tequatrovirus | 34_5 | Clustered | VC_34_5 |
| Escherichia~phage~vB_EcoM-VpaE1 | Caudovirales | Myoviridae | Felixo1virus | 16_0 | Clustered | VC_16_0 |
| Escherichia~phage~vB_EcoM-ep3 | Caudovirales | Myoviridae | Cvm10virus | 62_0 | Clustered | VC_62_0 |
| Escherichia~phage~vB_EcoM_112 | Caudovirales | Myoviridae | T4virus | 34_5 | Clustered | VC_34_5 |
| Escherichia~phage~vB_EcoM_ACG-C40 | Caudovirales | Myoviridae | T4virus | 34_5 | Clustered | VC_34_5 |
| Escherichia~phage~vB_EcoM_AYO145A | Caudovirales | Myoviridae | Felixo1virus | 16_0 | Clustered | VC_16_0 |
| Escherichia~phage~vB_EcoM_Alf5 | Caudovirales | Myoviridae | Felixounavirus | 16_0 | Clustered | VC_16_0 |
| Escherichia~phage~vB_EcoM_ECO1230-10 | Caudovirales | Myoviridae | Cvm10virus | 62_0 | Clustered | VC_62_0 |
| Escherichia~phage~vB_EcoM_ECOO78 | Caudovirales | Myoviridae | Jilinvirus | 62_0 | Clustered | VC_62_0 |
| Escherichia~phage~vB_EcoM_JS09 | Caudovirales | Myoviridae | Rb69virus | 34_0 | Clustered | VC_34_0 |
| Escherichia~phage~vB_EcoM_PhAPEC2 | Caudovirales | Myoviridae | Mosigvirus | 34_0 | Clustered | VC_34_0 |
| Escherichia~phage~vB_EcoM_VR20 | Caudovirales | Myoviridae | Sp18virus | 34_12 | Clustered | VC_34_12 |
| Escherichia~phage~vB_EcoM_VR25 | Caudovirales | Myoviridae | Sp18virus | 34_12 | Clustered | VC_34_12 |
| Escherichia~phage~vB_EcoM_VR26 | Caudovirales | Myoviridae | Sp18virus | 34_12 | Clustered | VC_34_12 |
| Escherichia~phage~vB_EcoM_VR7 | Caudovirales | Myoviridae | Sp18virus | 34_12 | Clustered | VC_34_12 |
| Escherichia~phage~vB_EcoP_24B | Caudovirales | Podoviridae | Nona33virus | 192_1 | Clustered | VC_192_1 |
| Escherichia~phage~vB_EcoP_G7C | Caudovirales | Podoviridae | G7cvirus | 7_1 | Clustered | VC_7_1 |
| Escherichia~phage~vB_EcoP_GA2A | Caudovirales | Podoviridae | Teseptimavirus | 210_0 | Clustered | VC_210_0 |
| Escherichia~phage~vB_EcoP_PhAPEC5 | Caudovirales | Podoviridae | G7cvirus | 7_1 | Clustered | VC_7_1 |
| Escherichia~phage~vB_EcoP_PhAPEC7 | Caudovirales | Podoviridae | G7cvirus | 7_1 | Clustered | VC_7_1 |
| Escherichia~phage~vB_EcoP_SU10 | Caudovirales | Podoviridae | Phieco32virus | 212_0 | Clustered | VC_212_0 |
| Escherichia~phage~vB_EcoS_AHP42 | Caudovirales | Siphoviridae | Rogue1virus | 64_0 | Clustered | VC_64_0 |
| Escherichia~phage~vB_EcoS_AHS24 | Caudovirales | Siphoviridae | Rogue1virus | 64_0 | Clustered | VC_64_0 |
| Escherichia~phage~vB_EcoS_AKS96 | Caudovirales | Siphoviridae | Rogue1virus | 64_0 | Clustered | VC_64_0 |
| Escherichia~phage~vB_EcoS_FFH1 | Caudovirales | Siphoviridae | T5virus | 261_1 | Clustered | VC_261_1 |
| Escherichia~phage~vB_Eco_ACG-M12 | Caudovirales | Siphoviridae | Rtpvirus | 64_0 | Clustered | VC_64_0 |
| Escherichia~phage~wV7 | Caudovirales | Myoviridae | Tequatrovirus | 34_5 | Clustered | VC_34_5 |
| Escherichia~phage~wV8 | Caudovirales | Myoviridae | Felixo1virus | 16_0 | Clustered | VC_16_0 |
| Escherichia~virus~186 | Caudovirales | Myoviridae | P2virus | 69_0 | Clustered | VC_69_0 |
| Escherichia~virus~AKFV33 | Caudovirales | Siphoviridae | T5virus | 261_1 | Clustered | VC_261_1 |
| Escherichia~virus~CBA120 | Caudovirales | Ackermannviridae | Cba120virus | 26_1 | Clustered | VC_26_1 |
| Escherichia~virus~DT57C | Caudovirales | Siphoviridae | T5virus | 261_1 | Clustered | VC_261_1 |
| Escherichia~virus~EPS7 | Caudovirales | Siphoviridae | T5virus | 261_1 | Clustered | VC_261_1 |
| Escherichia~virus~FI | Unassigned | Leviviridae | Allolevivirus | 33_0 | Clustered | VC_33_0 |
| Escherichia~virus~HK022 | Caudovirales | Siphoviridae | Hk97virus | 176_0 | Clustered | VC_176_0 |
| Escherichia~virus~HK97 | Caudovirales | Siphoviridae | Hk97virus | 176_0 | Clustered | VC_176_0 |
| Escherichia~virus~I22 | Unassigned | Inoviridae | Lineavirus | 251_0 | Clustered/Singleton | VC_251_0 |
| Escherichia~virus~JL1 | Caudovirales | Siphoviridae | Hk578virus | 233_0 | Clustered | VC_233_0 |
| Escherichia~virus~K1-5 | Caudovirales | Podoviridae | Sp6virus | 254_0 | Clustered | VC_254_0 |
| Escherichia~virus~K1E | Caudovirales | Podoviridae | Sp6virus | 254_0 | Clustered | VC_254_0 |
| Escherichia~virus~KP26 | Caudovirales | Siphoviridae | Rogue1virus | 64_0 | Clustered | VC_64_0 |
| Escherichia~virus~M13 | Unassigned | Inoviridae | Inovirus | 252_0 | Clustered | VC_252_0 |
| Escherichia~virus~Mu | Caudovirales | Myoviridae | Muvirus | 188_0 | Clustered | VC_188_0 |
| Escherichia~virus~N4 | Caudovirales | Podoviridae | N4virus | 7_1 | Clustered | VC_7_1 |
| Escherichia~virus~P1 | Caudovirales | Myoviridae | P1virus | 260_0 | Clustered | VC_260_0 |
| Escherichia~virus~P2 | Caudovirales | Myoviridae | P2virus | 69_0 | Clustered | VC_69_0 |
| Escherichia~virus~Qbeta | Unassigned | Leviviridae | Allolevivirus | 33_0 | Clustered | VC_33_0 |
| Escherichia~virus~RB14 | Caudovirales | Myoviridae | T4virus | 34_5 | Clustered | VC_34_5 |
| Escherichia~virus~RB16 | Caudovirales | Myoviridae | Unassigned | 37_1 | Clustered | VC_37_1 |
| Escherichia~virus~RB32 | Caudovirales | Myoviridae | Tequatrovirus | 34_5 | Clustered | VC_34_5 |
| Escherichia~virus~RB43 | Caudovirales | Myoviridae | Unassigned | 37_1 | Clustered | VC_37_1 |
| Escherichia~virus~Rtp | Caudovirales | Siphoviridae | Rtpvirus | 64_0 | Clustered | VC_64_0 |
| Escherichia~virus~SSL2009a | Caudovirales | Siphoviridae | Hk578virus | 233_0 | Clustered | VC_233_0 |
| Escherichia~virus~T1 | Caudovirales | Siphoviridae | T1virus | 64_0 | Clustered | VC_64_0 |
| Escherichia~virus~T4 | Caudovirales | Myoviridae | T4virus | 34_5 | Clustered | VC_34_5 |
| Escherichia~virus~T5 | Caudovirales | Siphoviridae | T5virus | 261_1 | Clustered | VC_261_1 |
| Escherichia~virus~TLS | Caudovirales | Siphoviridae | Tlsvirus | 64_0 | Clustered | VC_64_0 |
| Escherichia~virus~Wphi | Caudovirales | Myoviridae | P2virus | 69_0 | Clustered | VC_69_0 |
| Escherichia~virus~phiEco32 | Caudovirales | Podoviridae | Phieco32virus | 212_0 | Clustered | VC_212_0 |
| Escherichia~virus~phiX174 | Unassigned | Microviridae | Phix174microvirus | 246_0 | Clustered | VC_246_0 |
| Flavobacterium~phage~1H | Caudovirales | Siphoviridae | Unahavirus | 263_0 | Clustered | VC_263_0 |
| Flavobacterium~phage~23T | Caudovirales | Siphoviridae | Unahavirus | 263_0 | Clustered | VC_263_0 |
| Flavobacterium~phage~2A | Caudovirales | Siphoviridae | Unahavirus | 263_0 | Clustered | VC_263_0 |
| Flavobacterium~phage~6H | Caudovirales | Siphoviridae | Unahavirus | 263_0 | Clustered | VC_263_0 |
| Flavobacterium~phage~FCL-2 | Caudovirales | Myoviridae | Ficleduovirus | 209_0 | Clustered | VC_209_0 |
| Flavobacterium~phage~FCV-1 | Caudovirales | Myoviridae | Ficleduovirus | 209_0 | Clustered | VC_209_0 |
| Flavobacterium~phage~FpV4 | Caudovirales | Podoviridae | Fipvunavirus | 208_0 | Clustered | VC_208_0 |
| Flavobacterium~phage~Fpv1 | Caudovirales | Podoviridae | Fipvunavirus | 208_0 | Clustered | VC_208_0 |
| Flavobacterium~phage~Fpv10 | Unassigned | Unassigned | Unassigned | 167_0 | Clustered | VC_167_0 |
| Flavobacterium~phage~Fpv11 | Unassigned | Unassigned | Unassigned | 167_0 | Clustered | VC_167_0 |
| Flavobacterium~phage~Fpv2 | Caudovirales | Podoviridae | Fipvunavirus | 208_0 | Clustered | VC_208_0 |
| Flavobacterium~phage~Fpv20 | Caudovirales | Podoviridae | Fipvunavirus | 208_0 | Clustered | VC_208_0 |
| Flavobacterium~phage~Fpv3 | Caudovirales | Podoviridae | Fipvunavirus | 208_0 | Clustered | VC_208_0 |
| Flavobacterium~phage~Fpv5 | Unassigned | Unassigned | Unassigned | 167_0 | Clustered | VC_167_0 |
| Flavobacterium~phage~Fpv6 | Unassigned | Unassigned | Unassigned | 167_0 | Clustered | VC_167_0 |
| Flavobacterium~phage~Fpv7 | Unassigned | Unassigned | Unassigned | 167_0 | Clustered | VC_167_0 |
| Flavobacterium~phage~Fpv8 | Unassigned | Unassigned | Unassigned | 167_0 | Clustered | VC_167_0 |
| Geobacillus~phage~GBK2 | Caudovirales | Siphoviridae | Unassigned | 143_0 | Clustered | VC_143_0 |
| Geobacillus~phage~GBSV1 | Caudovirales | Myoviridae | Svunavirus | 136_0 | Clustered | VC_136_0 |
| Geobacillus~phage~TP-84 | Caudovirales | Siphoviridae | Tp84virus | 143_0 | Clustered | VC_143_0 |
| Geobacillus~virus~E2 | Caudovirales | Siphoviridae | Unassigned | 132_0 | Clustered | VC_132_0 |
| Geobacillus~virus~E3 | Caudovirales | Siphoviridae | Unassigned | 113_2 | Clustered/Singleton | VC_113_2 |
| Gokushovirinae~Bog1183_53 | Unassigned | Microviridae | Unassigned | 73_0 | Clustered | VC_73_0 |
| Gokushovirinae~Bog5712_52 | Unassigned | Microviridae | Unassigned | 73_0 | Clustered | VC_73_0 |
| Gokushovirinae~Bog8989_22 | Unassigned | Microviridae | Unassigned | 73_0 | Clustered | VC_73_0 |
| Gokushovirinae~Fen672_31 | Unassigned | Microviridae | Unassigned | 73_0 | Clustered | VC_73_0 |
| Gokushovirinae~Fen7875_21 | Unassigned | Microviridae | Unassigned | 73_0 | Clustered | VC_73_0 |
| Gordonia~phage~Attis | Caudovirales | Siphoviridae | Attisvirus | 264_0 | Clustered | VC_264_0 |
| Gordonia~phage~Bachita | Caudovirales | Siphoviridae | Smoothievirus | 268_0 | Clustered | VC_268_0 |
| Gordonia~phage~BaxterFox | Caudovirales | Siphoviridae | Baxtervirus | 264_1 | Clustered | VC_264_1 |
| Gordonia~phage~Blueberry | Caudovirales | Siphoviridae | Unassigned | 265_0 | Clustered | VC_265_0 |
| Gordonia~phage~Bowser | Caudovirales | Siphoviridae | Bowservirus | 103_0 | Clustered | VC_103_0 |
| Gordonia~phage~Brandonk123 | Caudovirales | Siphoviridae | Vividuovirus | 271_0 | Clustered | VC_271_0 |
| Gordonia~phage~BritBrat | Caudovirales | Siphoviridae | Britbratvirus | 100_0 | Clustered/Singleton | VC_100_0 |
| Gordonia~phage~CaptainKirk2 | Caudovirales | Siphoviridae | Unassigned | 265_0 | Clustered | VC_265_0 |
| Gordonia~phage~CarolAnn | Caudovirales | Siphoviridae | Unassigned | 265_0 | Clustered | VC_265_0 |
| Gordonia~phage~ClubL | Caudovirales | Siphoviridae | Smoothievirus | 268_0 | Clustered | VC_268_0 |
| Gordonia~phage~Cozz | Caudovirales | Siphoviridae | Emalynvirus | 275_0 | Clustered | VC_275_0 |
| Gordonia~phage~Cucurbita | Caudovirales | Siphoviridae | Smoothievirus | 268_0 | Clustered | VC_268_0 |
| Gordonia~phage~Demosthenes | Caudovirales | Siphoviridae | Demosthenesvirus | 278_0 | Clustered | VC_278_0 |
| Gordonia~phage~Emalyn | Caudovirales | Siphoviridae | Emalynvirus | 275_0 | Clustered | VC_275_0 |
| Gordonia~phage~GAL1 | Caudovirales | Siphoviridae | Galunavirus | 100_1 | Clustered/Singleton | VC_100_1 |
| Gordonia~phage~GMA1 | Caudovirales | Siphoviridae | Unassigned | 266_0 | Clustered | VC_266_0 |
| Gordonia~phage~GMA3 | Caudovirales | Siphoviridae | Gamtrevirus | 277_0 | Clustered | VC_277_0 |
| Gordonia~phage~GMA4 | Caudovirales | Siphoviridae | Unassigned | 224_0 | Clustered | VC_224_0 |
| Gordonia~phage~GMA5 | Caudovirales | Siphoviridae | Unassigned | 97_0 | Clustered | VC_97_0 |
| Gordonia~phage~GMA7 | Caudovirales | Siphoviridae | Getseptimavirus | 278_2 | Clustered | VC_278_2 |
| Gordonia~phage~GRU1 | Caudovirales | Siphoviridae | Unassigned | 223_0 | Clustered | VC_223_0 |
| Gordonia~phage~GRU3 | Caudovirales | Siphoviridae | Unassigned | 97_0 | Clustered | VC_97_0 |
| Gordonia~phage~GTE2 | Caudovirales | Siphoviridae | Emalynvirus | 275_0 | Clustered | VC_275_0 |
| Gordonia~phage~GTE5 | Caudovirales | Siphoviridae | Unassigned | 223_0 | Clustered | VC_223_0 |
| Gordonia~phage~GTE6 | Caudovirales | Siphoviridae | Unassigned | 271_0 | Clustered | VC_271_0 |
| Gordonia~phage~GTE7 | Caudovirales | Siphoviridae | Getseptimavirus | 278_2 | Clustered | VC_278_2 |
| Gordonia~phage~GTE8 | Caudovirales | Siphoviridae | Unassigned | 223_0 | Clustered | VC_223_0 |
| Gordonia~phage~Gmala1 | Caudovirales | Siphoviridae | Gordtnkvirus | 277_0 | Clustered | VC_277_0 |
| Gordonia~phage~GordDuk1 | Caudovirales | Siphoviridae | Gordtnkvirus | 277_0 | Clustered | VC_277_0 |
| Gordonia~phage~GordTnk2 | Caudovirales | Siphoviridae | Gordtnkvirus | 277_0 | Clustered | VC_277_0 |
| Gordonia~phage~Gsput1 | Caudovirales | Siphoviridae | Gesputvirus | 86_0 | Clustered | VC_86_0 |
| Gordonia~phage~Guacamole | Caudovirales | Siphoviridae | Unassigned | 265_0 | Clustered | VC_265_0 |
| Gordonia~phage~Gustav | Caudovirales | Siphoviridae | Gustavvirus | 85_0 | Clustered | VC_85_0 |
| Gordonia~phage~Hedwig | Caudovirales | Siphoviridae | Hedwigvirus | 103_0 | Clustered | VC_103_0 |
| Gordonia~phage~Hotorobo | Caudovirales | Siphoviridae | Woesvirus | 278_2 | Clustered | VC_278_2 |
| Gordonia~phage~Jumbo | Caudovirales | Siphoviridae | Gorjumvirus | 277_0 | Clustered | VC_277_0 |
| Gordonia~phage~Katyusha | Caudovirales | Siphoviridae | Demosthenesvirus | 278_0 | Clustered | VC_278_0 |
| Gordonia~phage~Kita | Caudovirales | Siphoviridae | Nymphadoravirus | 264_1 | Clustered | VC_264_1 |
| Gordonia~phage~Kvothe | Caudovirales | Siphoviridae | Demosthenesvirus | 278_0 | Clustered | VC_278_0 |
| Gordonia~phage~Lennon | Caudovirales | Siphoviridae | Vividuovirus | 271_0 | Clustered | VC_271_0 |
| Gordonia~phage~Lucky10 | Caudovirales | Siphoviridae | Unassigned | 100_2 | Clustered/Singleton | VC_100_2 |
| Gordonia~phage~Mahdia | Caudovirales | Siphoviridae | Gustavvirus | 85_0 | Clustered | VC_85_0 |
| Gordonia~phage~McGonagall | Caudovirales | Siphoviridae | Unassigned | 97_0 | Clustered | VC_97_0 |
| Gordonia~phage~Monty | Caudovirales | Siphoviridae | Woesvirus | 278_2 | Clustered | VC_278_2 |
| Gordonia~phage~Nymphadora | Caudovirales | Siphoviridae | Nymphadoravirus | 264_1 | Clustered | VC_264_1 |
| Gordonia~phage~Obliviate | Caudovirales | Siphoviridae | Unassigned | 265_0 | Clustered | VC_265_0 |
| Gordonia~phage~OneUp | Caudovirales | Siphoviridae | Smoothievirus | 268_1 | Clustered/Singleton | VC_268_1 |
| Gordonia~phage~Phinally | Caudovirales | Siphoviridae | Unassigned | 271_0 | Clustered | VC_271_0 |
| Gordonia~phage~Schwabeltier | Caudovirales | Siphoviridae | Bowservirus | 103_0 | Clustered | VC_103_0 |
| Gordonia~phage~Smoothie | Caudovirales | Siphoviridae | Smoothievirus | 268_0 | Clustered | VC_268_0 |
| Gordonia~phage~SoilAssassin | Caudovirales | Siphoviridae | Attisvirus | 264_0 | Clustered | VC_264_0 |
| Gordonia~phage~Splinter | Caudovirales | Siphoviridae | Vendettavirus | 86_0 | Clustered | VC_86_0 |
| Gordonia~phage~Troje | Caudovirales | Siphoviridae | Emalynvirus | 275_0 | Clustered | VC_275_0 |
| Gordonia~phage~Twister6 | Caudovirales | Siphoviridae | Wizardvirus | 99_0 | Clustered | VC_99_0 |
| Gordonia~phage~UmaThurman | Caudovirales | Siphoviridae | Unassigned | 265_0 | Clustered | VC_265_0 |
| Gordonia~phage~Utz | Caudovirales | Siphoviridae | Unassigned | 265_0 | Clustered | VC_265_0 |
| Gordonia~phage~Vendetta | Caudovirales | Siphoviridae | Vendettavirus | 86_0 | Clustered | VC_86_0 |
| Gordonia~phage~Vivi2 | Caudovirales | Siphoviridae | Vividuovirus | 271_0 | Clustered | VC_271_0 |
| Gordonia~phage~Wizard | Caudovirales | Siphoviridae | Wizardvirus | 99_0 | Clustered | VC_99_0 |
| Gordonia~phage~Woes | Caudovirales | Siphoviridae | Woesvirus | 278_2 | Clustered | VC_278_2 |
| Gordonia~phage~Yeezy | Caudovirales | Siphoviridae | Baxtervirus | 264_1 | Clustered | VC_264_1 |
| Gordonia~phage~Zirinka | Caudovirales | Siphoviridae | Nymphadoravirus | 264_1 | Clustered | VC_264_1 |
| Guinea~pig~Chlamydia~phage | Unassigned | Microviridae | Chlamydiamicrovirus | 73_0 | Clustered | VC_73_0 |
| Haemophilus~phage~Aaphi23 | Caudovirales | Myoviridae | Unassigned | 6_0 | Clustered | VC_6_0 |
| Haemophilus~virus~HP1 | Caudovirales | Myoviridae | Hp1virus | 70_0 | Clustered | VC_70_0 |
| Haemophilus~virus~HP2 | Caudovirales | Myoviridae | Hp1virus | 70_0 | Clustered | VC_70_0 |
| Haloarcula~californiae~icosahedral~virus~1 | Unassigned | Sphaerolipoviridae | Alphasphaerolipovirus | 288_0 | Clustered | VC_288_0 |
| Haloarcula~hispanica~icosahedral~virus~2 | Unassigned | Sphaerolipoviridae | Alphasphaerolipovirus | 288_0 | Clustered | VC_288_0 |
| Haloarcula~hispanica~pleomorphic~virus~1 | Unassigned | Pleolipoviridae | Alphapleolipovirus | 289_0 | Clustered | VC_289_0 |
| Haloarcula~hispanica~pleomorphic~virus~2 | Unassigned | Pleolipoviridae | Alphapleolipovirus | 289_0 | Clustered | VC_289_0 |
| Haloarcula~hispanica~virus~PH1 | Unassigned | Sphaerolipoviridae | Alphasphaerolipovirus | 288_0 | Clustered | VC_288_0 |
| Haloarcula~hispanica~virus~SH1 | Unassigned | Sphaerolipoviridae | Alphasphaerolipovirus | 288_0 | Clustered | VC_288_0 |
| Halomonas~virus~HAP1 | Caudovirales | Myoviridae | Hapunavirus | 247_0 | Clustered | VC_247_0 |
| Halorubrum~phage~CGphi46 | Unassigned | Unassigned | Unassigned | 82_0 | Clustered | VC_82_0 |
| Halorubrum~phage~HF2 | Unassigned | Unassigned | Unassigned | 292_0 | Clustered | VC_292_0 |
| Halorubrum~pleomorphic~virus~1 | Unassigned | Pleolipoviridae | Alphapleolipovirus | 290_0 | Clustered | VC_290_0 |
| Halorubrum~pleomorphic~virus~2 | Unassigned | Pleolipoviridae | Alphapleolipovirus | 290_0 | Clustered | VC_290_0 |
| Halorubrum~pleomorphic~virus~6 | Unassigned | Pleolipoviridae | Alphapleolipovirus | 290_0 | Clustered | VC_290_0 |
| Halovirus~HCTV-1 | Unassigned | Unassigned | Unassigned | 291_0 | Clustered | VC_291_0 |
| Halovirus~HCTV-2 | Unassigned | Unassigned | Unassigned | 294_0 | Clustered | VC_294_0 |
| Halovirus~HCTV-5 | Unassigned | Unassigned | Unassigned | 291_0 | Clustered | VC_291_0 |
| Halovirus~HF1 | Unassigned | Unassigned | Unassigned | 292_0 | Clustered | VC_292_0 |
| Halovirus~HHTV-2 | Unassigned | Unassigned | Unassigned | 294_0 | Clustered | VC_294_0 |
| Halovirus~HRTV-5 | Unassigned | Unassigned | Unassigned | 292_0 | Clustered | VC_292_0 |
| Halovirus~HRTV-8 | Unassigned | Unassigned | Unassigned | 292_0 | Clustered | VC_292_0 |
| Halovirus~HVTV-1 | Unassigned | Unassigned | Unassigned | 291_0 | Clustered | VC_291_0 |
| Hamiltonella~virus~APSE1 | Caudovirales | Podoviridae | Unassigned | 162_0 | Clustered | VC_162_0 |
| Helicobacter~phage~1961P | Caudovirales | Podoviridae | Una961virus | 295_0 | Clustered | VC_295_0 |
| Helicobacter~phage~KHP30 | Caudovirales | Podoviridae | Una961virus | 295_0 | Clustered | VC_295_0 |
| Helicobacter~phage~KHP40 | Caudovirales | Podoviridae | Una961virus | 295_0 | Clustered | VC_295_0 |
| Helicobacter~phage~phiHP33 | Caudovirales | Podoviridae | Schmidvirus | 295_0 | Clustered | VC_295_0 |
| Idiomarinaceae~phage~Phi1M2-2 | Caudovirales | Siphoviridae | Unassigned | 60_0 | Clustered | VC_60_0 |
| Iodobacteriophage~phiPLPE | Caudovirales | Myoviridae | Unassigned | 65_0 | Clustered | VC_65_0 |
| Klebsiella~phage~1513 | Caudovirales | Siphoviridae | Kp36virus | 64_0 | Clustered | VC_64_0 |
| Klebsiella~phage~JD001 | Caudovirales | Myoviridae | Jedunavirus | 65_0 | Clustered | VC_65_0 |
| Klebsiella~phage~JD18 | Caudovirales | Myoviridae | Jd18virus | 34_9 | Clustered | VC_34_9 |
| Klebsiella~phage~K11 | Caudovirales | Podoviridae | Kp32virus | 210_0 | Clustered | VC_210_0 |
| Klebsiella~phage~K5 | Caudovirales | Podoviridae | Kp32virus | 210_0 | Clustered | VC_210_0 |
| Klebsiella~phage~KLPN1 | Caudovirales | Siphoviridae | Kp36virus | 64_0 | Clustered | VC_64_0 |
| Klebsiella~phage~KP15 | Caudovirales | Myoviridae | Kp15virus | 37_0 | Clustered | VC_37_0 |
| Klebsiella~phage~KP27 | Caudovirales | Myoviridae | Kp15virus | 37_0 | Clustered | VC_37_0 |
| Klebsiella~phage~KP32 | Caudovirales | Podoviridae | Kp32virus | 210_0 | Clustered | VC_210_0 |
| Klebsiella~phage~KP36 | Caudovirales | Siphoviridae | Kp36virus | 64_0 | Clustered | VC_64_0 |
| Klebsiella~phage~Matisse | Caudovirales | Myoviridae | Kp15virus | 37_0 | Clustered | VC_37_0 |
| Klebsiella~phage~Miro | Caudovirales | Myoviridae | Kp15virus | 37_0 | Clustered | VC_37_0 |
| Klebsiella~phage~PKO111 | Caudovirales | Myoviridae | Jd18virus | 34_10 | Clustered/Singleton | VC_34_10 |
| Klebsiella~phage~PKP126 | Caudovirales | Siphoviridae | Kp36virus | 64_0 | Clustered | VC_64_0 |
| Klebsiella~phage~PMBT1 | Caudovirales | Myoviridae | Slopekvirus | 37_0 | Clustered | VC_37_0 |
| Klebsiella~phage~Sugarland | Caudovirales | Siphoviridae | Sugarlandvirus | 261_0 | Clustered | VC_261_0 |
| Klebsiella~phage~Sushi | Caudovirales | Siphoviridae | Kp36virus | 64_0 | Clustered | VC_64_0 |
| Klebsiella~phage~phiKO2 | Caudovirales | Siphoviridae | Unassigned | 185_0 | Clustered | VC_185_0 |
| Klebsiella~phage~vB_Kp1 | Caudovirales | Podoviridae | Kp32virus | 210_0 | Clustered | VC_210_0 |
| Klebsiella~phage~vB_KpnM_KB57 | Caudovirales | Myoviridae | Unassigned | 226_3 | Clustered/Singleton | VC_226_3 |
| Klebsiella~phage~vB_KpnM_KpV477 | Caudovirales | Myoviridae | Jiaodavirus | 34_9 | Clustered | VC_34_9 |
| Klebsiella~phage~vB_KpnM_KpV52 | Caudovirales | Myoviridae | Jedunavirus | 65_0 | Clustered | VC_65_0 |
| Klebsiella~phage~vB_KpnM_KpV79 | Caudovirales | Myoviridae | Jedunavirus | 65_0 | Clustered | VC_65_0 |
| Klebsiella~phage~vB_KpnP_KpV289 | Caudovirales | Podoviridae | Kp32virus | 210_0 | Clustered | VC_210_0 |
| Klebsiella~phage~vB_Kpn_IME260 | Caudovirales | Siphoviridae | Sugarlandvirus | 261_0 | Clustered | VC_261_0 |
| Klebsiella~virus~0507KN21 | Caudovirales | Ackermannviridae | Unassigned | 26_1 | Clustered | VC_26_1 |
| Kluyvera~phage~Kvp1 | Caudovirales | Podoviridae | T7virus | 210_0 | Clustered | VC_210_0 |
| Lactobacillus~phage~A2 | Caudovirales | Siphoviridae | Unassigned | 149_0 | Clustered | VC_149_0 |
| Lactobacillus~phage~J-1 | Caudovirales | Siphoviridae | Unassigned | 149_0 | Clustered | VC_149_0 |
| Lactobacillus~phage~JCL1032 | Caudovirales | Siphoviridae | Unassigned | 119_0 | Clustered | VC_119_0 |
| Lactobacillus~phage~KC5a | Caudovirales | Myoviridae | Unassigned | 170_0 | Clustered | VC_170_0 |
| Lactobacillus~phage~LF1 | Caudovirales | Siphoviridae | Unassigned | 255_0 | Clustered | VC_255_0 |
| Lactobacillus~phage~LL-H | Caudovirales | Siphoviridae | Unassigned | 119_0 | Clustered | VC_119_0 |
| Lactobacillus~phage~Lc-Nu | Caudovirales | Siphoviridae | Unassigned | 149_0 | Clustered | VC_149_0 |
| Lactobacillus~phage~Ld17 | Caudovirales | Siphoviridae | C5virus | 259_0 | Clustered | VC_259_0 |
| Lactobacillus~phage~Ld25A | Caudovirales | Siphoviridae | C5virus | 259_0 | Clustered | VC_259_0 |
| Lactobacillus~phage~Ld3 | Caudovirales | Siphoviridae | C5virus | 259_0 | Clustered | VC_259_0 |
| Lactobacillus~phage~Ldl1 | Caudovirales | Siphoviridae | Unassigned | 119_0 | Clustered | VC_119_0 |
| Lactobacillus~phage~Lrm1 | Caudovirales | Siphoviridae | Unassigned | 149_0 | Clustered | VC_149_0 |
| Lactobacillus~phage~PL-1 | Caudovirales | Siphoviridae | Unassigned | 149_0 | Clustered | VC_149_0 |
| Lactobacillus~phage~PLE2 | Caudovirales | Siphoviridae | Unassigned | 131_0 | Clustered | VC_131_0 |
| Lactobacillus~phage~iA2 | Caudovirales | Siphoviridae | Unassigned | 131_0 | Clustered | VC_131_0 |
| Lactobacillus~phage~phi~jlb1 | Caudovirales | Myoviridae | Unassigned | 170_0 | Clustered | VC_170_0 |
| Lactobacillus~phage~phiAQ113 | Caudovirales | Myoviridae | Unassigned | 170_0 | Clustered | VC_170_0 |
| Lactobacillus~phage~phiAT3 | Caudovirales | Siphoviridae | Unassigned | 149_0 | Clustered | VC_149_0 |
| Lactobacillus~phage~phiJB | Caudovirales | Siphoviridae | Unassigned | 119_0 | Clustered | VC_119_0 |
| Lactobacillus~phage~phiLdb | Caudovirales | Siphoviridae | C5virus | 259_0 | Clustered | VC_259_0 |
| Lactobacillus~phage~phiPYB5 | Caudovirales | Siphoviridae | Unassigned | 255_0 | Clustered | VC_255_0 |
| Lactobacillus~prophage~Lj771 | Caudovirales | Myoviridae | Unassigned | 170_0 | Clustered | VC_170_0 |
| Lactobacillus~virus~ATCC8014 | Caudovirales | Siphoviridae | Phijl1virus | 175_0 | Clustered | VC_175_0 |
| Lactobacillus~virus~LLKu | Caudovirales | Siphoviridae | C5virus | 259_0 | Clustered | VC_259_0 |
| Lactobacillus~virus~c5 | Caudovirales | Siphoviridae | C5virus | 259_0 | Clustered | VC_259_0 |
| Lactobacillus~virus~phiJL1 | Caudovirales | Siphoviridae | Phijl1virus | 175_0 | Clustered | VC_175_0 |
| Lactococcus~phage~1358 | Caudovirales | Siphoviridae | Unassigned | 142_0 | Clustered | VC_142_0 |
| Lactococcus~phage~340 | Caudovirales | Siphoviridae | Skunavirus | 299_0 | Clustered | VC_299_0 |
| Lactococcus~phage~949 | Caudovirales | Siphoviridae | Unassigned | 154_0 | Clustered | VC_154_0 |
| Lactococcus~phage~BK5-T | Caudovirales | Siphoviridae | Unassigned | 158_0 | Clustered | VC_158_0 |
| Lactococcus~phage~BM13 | Caudovirales | Siphoviridae | Unassigned | 158_0 | Clustered | VC_158_0 |
| Lactococcus~phage~D4410 | Caudovirales | Siphoviridae | Ceduovirus | 300_0 | Clustered | VC_300_0 |
| Lactococcus~phage~D4412 | Caudovirales | Siphoviridae | Ceduovirus | 300_0 | Clustered | VC_300_0 |
| Lactococcus~phage~M5938 | Caudovirales | Siphoviridae | Ceduovirus | 300_0 | Clustered | VC_300_0 |
| Lactococcus~phage~M6162 | Caudovirales | Siphoviridae | Ceduovirus | 300_0 | Clustered | VC_300_0 |
| Lactococcus~phage~M6165 | Caudovirales | Siphoviridae | Ceduovirus | 300_0 | Clustered | VC_300_0 |
| Lactococcus~phage~P078 | Caudovirales | Siphoviridae | Unassigned | 95_0 | Clustered | VC_95_0 |
| Lactococcus~phage~P092 | Caudovirales | Siphoviridae | Unassigned | 95_0 | Clustered | VC_95_0 |
| Lactococcus~phage~P118 | Caudovirales | Siphoviridae | Unassigned | 95_0 | Clustered | VC_95_0 |
| Lactococcus~phage~P162 | Caudovirales | Siphoviridae | Unassigned | 95_0 | Clustered | VC_95_0 |
| Lactococcus~phage~P335~sensu~lato | Caudovirales | Siphoviridae | Unassigned | 158_0 | Clustered | VC_158_0 |
| Lactococcus~phage~P680 | Caudovirales | Siphoviridae | Skunavirus | 299_0 | Clustered | VC_299_0 |
| Lactococcus~phage~WRP3 | Caudovirales | Siphoviridae | Unassigned | 154_0 | Clustered | VC_154_0 |
| Lactococcus~phage~bIL285 | Caudovirales | Siphoviridae | Unassigned | 158_0 | Clustered | VC_158_0 |
| Lactococcus~phage~bIL286 | Caudovirales | Siphoviridae | Unassigned | 158_0 | Clustered | VC_158_0 |
| Lactococcus~phage~bIL309 | Caudovirales | Siphoviridae | Unassigned | 158_0 | Clustered | VC_158_0 |
| Lactococcus~phage~bIL310 | Caudovirales | Siphoviridae | Unassigned | 301_0 | Clustered | VC_301_0 |
| Lactococcus~phage~bIL312 | Caudovirales | Siphoviridae | Unassigned | 301_0 | Clustered | VC_301_0 |
| Lactococcus~phage~jm2 | Caudovirales | Siphoviridae | Skunavirus | 299_0 | Clustered | VC_299_0 |
| Lactococcus~phage~jm3 | Caudovirales | Siphoviridae | Skunavirus | 299_0 | Clustered | VC_299_0 |
| Lactococcus~phage~phi7 | Caudovirales | Siphoviridae | Skunavirus | 299_0 | Clustered | VC_299_0 |
| Lactococcus~phage~phiL47 | Caudovirales | Siphoviridae | Unassigned | 154_0 | Clustered | VC_154_0 |
| Lactococcus~virus~712 | Caudovirales | Siphoviridae | Sk1virus | 299_0 | Clustered | VC_299_0 |
| Lactococcus~virus~ASCC191 | Caudovirales | Siphoviridae | Sk1virus | 299_0 | Clustered | VC_299_0 |
| Lactococcus~virus~Bibb29 | Caudovirales | Siphoviridae | Sk1virus | 299_0 | Clustered | VC_299_0 |
| Lactococcus~virus~CB13 | Caudovirales | Siphoviridae | Sk1virus | 299_0 | Clustered | VC_299_0 |
| Lactococcus~virus~CB14 | Caudovirales | Siphoviridae | Sk1virus | 299_0 | Clustered | VC_299_0 |
| Lactococcus~virus~CB19 | Caudovirales | Siphoviridae | Sk1virus | 299_0 | Clustered | VC_299_0 |
| Lactococcus~virus~CB20 | Caudovirales | Siphoviridae | Sk1virus | 299_0 | Clustered | VC_299_0 |
| Lactococcus~virus~P008 | Caudovirales | Siphoviridae | Sk1virus | 299_0 | Clustered | VC_299_0 |
| Lactococcus~virus~P2 | Caudovirales | Siphoviridae | Sk1virus | 299_0 | Clustered | VC_299_0 |
| Lactococcus~virus~Sl4 | Caudovirales | Siphoviridae | Sk1virus | 299_0 | Clustered | VC_299_0 |
| Lactococcus~virus~bIL170 | Caudovirales | Siphoviridae | Sk1virus | 299_0 | Clustered | VC_299_0 |
| Lactococcus~virus~bIL67 | Caudovirales | Siphoviridae | C2virus | 300_0 | Clustered | VC_300_0 |
| Lactococcus~virus~c2 | Caudovirales | Siphoviridae | C2virus | 300_0 | Clustered | VC_300_0 |
| Lactococcus~virus~jj50 | Caudovirales | Siphoviridae | Sk1virus | 299_0 | Clustered | VC_299_0 |
| Lactococcus~virus~sk1 | Caudovirales | Siphoviridae | Sk1virus | 299_0 | Clustered | VC_299_0 |
| Lelliottia~phage~phD2B | Caudovirales | Podoviridae | Zindervirus | 254_0 | Clustered | VC_254_0 |
| Leuconostoc~phage~1-A4 | Caudovirales | Siphoviridae | Una4virus | 303_0 | Clustered | VC_303_0 |
| Leuconostoc~phage~Lmd1 | Caudovirales | Siphoviridae | Lmd1virus | 302_0 | Clustered | VC_302_0 |
| Leuconostoc~phage~Ln-8 | Caudovirales | Siphoviridae | Una4virus | 303_0 | Clustered | VC_303_0 |
| Leuconostoc~phage~Ln-9 | Caudovirales | Siphoviridae | Una4virus | 303_0 | Clustered | VC_303_0 |
| Leuconostoc~phage~P793 | Caudovirales | Siphoviridae | Lmd1virus | 302_0 | Clustered | VC_302_0 |
| Leuconostoc~phage~phiLN03 | Caudovirales | Siphoviridae | Lmd1virus | 302_0 | Clustered | VC_302_0 |
| Leuconostoc~phage~phiLN04 | Caudovirales | Siphoviridae | Lmd1virus | 302_0 | Clustered | VC_302_0 |
| Leuconostoc~phage~phiLN12 | Caudovirales | Siphoviridae | Lmd1virus | 302_0 | Clustered | VC_302_0 |
| Leuconostoc~phage~phiLN25 | Caudovirales | Siphoviridae | Una4virus | 303_0 | Clustered | VC_303_0 |
| Leuconostoc~phage~phiLN34 | Caudovirales | Siphoviridae | Una4virus | 303_0 | Clustered | VC_303_0 |
| Leuconostoc~phage~phiLN6B | Caudovirales | Siphoviridae | Lmd1virus | 302_0 | Clustered | VC_302_0 |
| Leuconostoc~phage~phiLNTR2 | Caudovirales | Siphoviridae | Unaquatrovirus | 303_0 | Clustered | VC_303_0 |
| Leuconostoc~phage~phiLNTR3 | Caudovirales | Siphoviridae | Una4virus | 303_0 | Clustered | VC_303_0 |
| Liberibacter~phage~SC1 | Caudovirales | Podoviridae | Unassigned | 169_0 | Clustered | VC_169_0 |
| Liberibacter~phage~SC2 | Caudovirales | Podoviridae | Unassigned | 169_0 | Clustered | VC_169_0 |
| Listeria~phage~A118 | Caudovirales | Siphoviridae | Unassigned | 121_0 | Clustered | VC_121_0 |
| Listeria~phage~A500 | Caudovirales | Siphoviridae | Unassigned | 121_0 | Clustered | VC_121_0 |
| Listeria~phage~B025 | Caudovirales | Siphoviridae | Psavirus | 155_0 | Clustered | VC_155_0 |
| Listeria~phage~LMSP-25 | Caudovirales | Herelleviridae | Pecentumvirus | 115_0 | Clustered | VC_115_0 |
| Listeria~phage~LMTA-148 | Caudovirales | Herelleviridae | Pecentumvirus | 115_0 | Clustered | VC_115_0 |
| Listeria~phage~LMTA-34 | Caudovirales | Herelleviridae | Pecentumvirus | 115_0 | Clustered | VC_115_0 |
| Listeria~phage~LP-026 | Caudovirales | Siphoviridae | P70virus | 139_0 | Clustered | VC_139_0 |
| Listeria~phage~LP-030-2 | Caudovirales | Siphoviridae | Psavirus | 155_0 | Clustered | VC_155_0 |
| Listeria~phage~LP-030-3 | Caudovirales | Siphoviridae | Unassigned | 121_0 | Clustered | VC_121_0 |
| Listeria~phage~LP-037 | Caudovirales | Siphoviridae | P70virus | 139_0 | Clustered | VC_139_0 |
| Listeria~phage~LP-048 | Caudovirales | Herelleviridae | Pecentumvirus | 115_0 | Clustered | VC_115_0 |
| Listeria~phage~LP-064 | Caudovirales | Herelleviridae | Pecentumvirus | 115_0 | Clustered | VC_115_0 |
| Listeria~phage~LP-083-2 | Caudovirales | Herelleviridae | Pecentumvirus | 115_0 | Clustered | VC_115_0 |
| Listeria~phage~LP-101 | Caudovirales | Siphoviridae | Psavirus | 155_0 | Clustered | VC_155_0 |
| Listeria~phage~LP-110 | Caudovirales | Siphoviridae | P70virus | 139_0 | Clustered | VC_139_0 |
| Listeria~phage~LP-114 | Caudovirales | Siphoviridae | P70virus | 139_0 | Clustered | VC_139_0 |
| Listeria~phage~LP-125 | Caudovirales | Myoviridae | P100virus | 115_0 | Clustered | VC_115_0 |
| Listeria~phage~List-36 | Caudovirales | Herelleviridae | Pecentumvirus | 115_0 | Clustered | VC_115_0 |
| Listeria~phage~P35 | Caudovirales | Siphoviridae | Unassigned | 142_0 | Clustered | VC_142_0 |
| Listeria~phage~P40 | Caudovirales | Siphoviridae | Unassigned | 142_0 | Clustered | VC_142_0 |
| Listeria~phage~P70 | Caudovirales | Siphoviridae | P70virus | 139_0 | Clustered | VC_139_0 |
| Listeria~phage~PSA | Caudovirales | Siphoviridae | Psavirus | 155_0 | Clustered | VC_155_0 |
| Listeria~phage~vB_LmoM_AG20 | Caudovirales | Herelleviridae | Pecentumvirus | 115_0 | Clustered | VC_115_0 |
| Listeria~phage~vB_LmoS_188 | Caudovirales | Siphoviridae | Unassigned | 121_0 | Clustered | VC_121_0 |
| Listeria~phage~vB_LmoS_293 | Caudovirales | Siphoviridae | Unassigned | 121_0 | Clustered | VC_121_0 |
| Listeria~virus~A511 | Caudovirales | Myoviridae | P100virus | 115_0 | Clustered | VC_115_0 |
| Listeria~virus~P100 | Caudovirales | Myoviridae | P100virus | 115_0 | Clustered | VC_115_0 |
| Lynx~canadensis~associated~microvirus~CLP~9413 | Unassigned | Microviridae | Unassigned | 75_0 | Clustered | VC_75_0 |
| Mannheimia~phage~vB_MhM_587AP1 | Caudovirales | Myoviridae | Peduovirus | 69_0 | Clustered | VC_69_0 |
| Mannheimia~phage~vB_MhS_1152AP2 | Caudovirales | Siphoviridae | Unassigned | 66_0 | Clustered | VC_66_0 |
| Mannheimia~phage~vB_MhS_535AP2 | Caudovirales | Siphoviridae | Unassigned | 66_0 | Clustered | VC_66_0 |
| Mannheimia~phage~vB_MhS_587AP2 | Caudovirales | Siphoviridae | Unassigned | 66_0 | Clustered | VC_66_0 |
| Mannheimia~virus~PHL101 | Caudovirales | Myoviridae | P2virus | 69_0 | Clustered | VC_69_0 |
| Marine~gokushovirus | Unassigned | Microviridae | Unassigned | 73_0 | Clustered | VC_73_0 |
| Methanobacterium~phage~psiM2 | Caudovirales | Siphoviridae | Unassigned | 304_0 | Clustered | VC_304_0 |
| Methanothermobacter~phage~psiM100 | Caudovirales | Siphoviridae | Unassigned | 304_0 | Clustered | VC_304_0 |
| Microbacterium~phage~Eleri | Caudovirales | Siphoviridae | Elerivirus | 305_0 | Clustered | VC_305_0 |
| Microbacterium~phage~Golden | Caudovirales | Siphoviridae | Kojivirus | 305_0 | Clustered | VC_305_0 |
| Microbacterium~phage~Hamlet | Caudovirales | Siphoviridae | Ilzatvirus | 305_0 | Clustered | VC_305_0 |
| Microbacterium~phage~Ilzat | Caudovirales | Siphoviridae | Ilzatvirus | 305_0 | Clustered | VC_305_0 |
| Microbacterium~phage~Koji | Caudovirales | Siphoviridae | Kojivirus | 305_0 | Clustered | VC_305_0 |
| Microbacterium~phage~Pikmin | Caudovirales | Siphoviridae | Pikminvirus | 305_0 | Clustered | VC_305_0 |
| Microbacterium~phage~vB_MoxS-ISF9 | Caudovirales | Siphoviridae | Unassigned | 91_0 | Clustered | VC_91_0 |
| Microcystis~phage~MaMV-DC | Caudovirales | Myoviridae | Unassigned | 306_0 | Clustered | VC_306_0 |
| Microcystis~virus~Ma-LMM01 | Caudovirales | Myoviridae | Unassigned | 306_0 | Clustered | VC_306_0 |
| Microviridae~Bog5275_51 | Unassigned | Microviridae | Unassigned | 76_0 | Clustered | VC_76_0 |
| Microviridae~Bog9017_22 | Unassigned | Microviridae | Unassigned | 76_0 | Clustered | VC_76_0 |
| Microviridae~Fen2266_11 | Unassigned | Microviridae | Unassigned | 76_0 | Clustered | VC_76_0 |
| Microviridae~Fen418_41 | Unassigned | Microviridae | Unassigned | 76_0 | Clustered | VC_76_0 |
| Microviridae~Fen685_11 | Unassigned | Microviridae | Unassigned | 76_0 | Clustered | VC_76_0 |
| Microviridae~Fen7786_21 | Unassigned | Microviridae | Unassigned | 76_0 | Clustered | VC_76_0 |
| Microviridae~Fen7895_21 | Unassigned | Microviridae | Unassigned | 76_0 | Clustered | VC_76_0 |
| Morganella~phage~MmP1 | Caudovirales | Podoviridae | Teseptimavirus | 210_0 | Clustered | VC_210_0 |
| Morganella~phage~vB_MmoP_MP2 | Caudovirales | Podoviridae | Teseptimavirus | 210_0 | Clustered | VC_210_0 |
| Mycobacterium~phage~20ES | Caudovirales | Siphoviridae | Fromanvirus | 283_2 | Clustered | VC_283_2 |
| Mycobacterium~phage~40AC | Caudovirales | Siphoviridae | Fromanvirus | 283_2 | Clustered | VC_283_2 |
| Mycobacterium~phage~Abrogate | Caudovirales | Siphoviridae | Fromanvirus | 307_0 | Clustered | VC_307_0 |
| Mycobacterium~phage~Adzzy | Caudovirales | Siphoviridae | Fromanvirus | 283_2 | Clustered | VC_283_2 |
| Mycobacterium~phage~Aeneas | Caudovirales | Siphoviridae | Fromanvirus | 307_0 | Clustered | VC_307_0 |
| Mycobacterium~phage~Alice | Caudovirales | Myoviridae | Bxz1virus | 308_0 | Clustered | VC_308_0 |
| Mycobacterium~phage~Alsfro | Caudovirales | Siphoviridae | Fromanvirus | 307_0 | Clustered | VC_307_0 |
| Mycobacterium~phage~Alvin | Caudovirales | Siphoviridae | Fromanvirus | 307_0 | Clustered | VC_307_0 |
| Mycobacterium~phage~Angel | Caudovirales | Siphoviridae | Liefievirus | 101_0 | Clustered | VC_101_0 |
| Mycobacterium~phage~AnnaL29 | Caudovirales | Siphoviridae | Fromanvirus | 283_2 | Clustered | VC_283_2 |
| Mycobacterium~phage~Anubis | Caudovirales | Siphoviridae | Fromanvirus | 284_0 | Clustered | VC_284_0 |
| Mycobacterium~phage~Apizium | Caudovirales | Siphoviridae | Pg1virus | 58_0 | Clustered | VC_58_0 |
| Mycobacterium~phage~ArcherNM | Caudovirales | Siphoviridae | Fromanvirus | 283_2 | Clustered | VC_283_2 |
| Mycobacterium~phage~ArcherS7 | Caudovirales | Myoviridae | Bxz1virus | 308_0 | Clustered | VC_308_0 |
| Mycobacterium~phage~Archie | Caudovirales | Siphoviridae | Bronvirus | 309_0 | Clustered | VC_309_0 |
| Mycobacterium~phage~Ariel | Caudovirales | Siphoviridae | Omegavirus | 272_0 | Clustered | VC_272_0 |
| Mycobacterium~phage~Artemis2UCLA | Caudovirales | Siphoviridae | Fromanvirus | 283_1 | Clustered | VC_283_1 |
| Mycobacterium~phage~Astraea | Caudovirales | Myoviridae | Bxz1virus | 308_0 | Clustered | VC_308_0 |
| Mycobacterium~phage~BTCU-1 | Caudovirales | Siphoviridae | Fromanvirus | 284_0 | Clustered | VC_284_0 |
| Mycobacterium~phage~Bactobuster | Caudovirales | Siphoviridae | Fromanvirus | 283_2 | Clustered | VC_283_2 |
| Mycobacterium~phage~Badfish | Caudovirales | Siphoviridae | Pg1virus | 58_0 | Clustered | VC_58_0 |
| Mycobacterium~phage~Barnyard | Caudovirales | Siphoviridae | Barnyardvirus | 280_0 | Clustered | VC_280_0 |
| Mycobacterium~phage~BarrelRoll | Caudovirales | Siphoviridae | Timquatrovirus | 102_2 | Clustered | VC_102_2 |
| Mycobacterium~phage~Barriga | Caudovirales | Siphoviridae | Fromanvirus | 307_0 | Clustered | VC_307_0 |
| Mycobacterium~phage~BellusTerra | Caudovirales | Siphoviridae | Fromanvirus | 284_1 | Clustered | VC_284_1 |
| Mycobacterium~phage~Bernal13 | Caudovirales | Siphoviridae | Bernal13virus | 269_0 | Clustered/Singleton | VC_269_0 |
| Mycobacterium~phage~BigNuz | Caudovirales | Siphoviridae | Bignuzvirus | 267_0 | Clustered | VC_267_0 |
| Mycobacterium~phage~Bipolar | Caudovirales | Siphoviridae | Cheoctovirus | 274_0 | Clustered | VC_274_0 |
| Mycobacterium~phage~Blue7 | Caudovirales | Siphoviridae | Fromanvirus | 283_1 | Clustered | VC_283_1 |
| Mycobacterium~phage~Bobi | Caudovirales | Siphoviridae | Cheoctovirus | 274_1 | Clustered | VC_274_1 |
| Mycobacterium~phage~Bongo | Caudovirales | Siphoviridae | Bongovirus | 310_0 | Clustered | VC_310_0 |
| Mycobacterium~phage~Breeniome | Caudovirales | Myoviridae | Bxz1virus | 308_0 | Clustered | VC_308_0 |
| Mycobacterium~phage~Brocalys | Caudovirales | Siphoviridae | Cheoctovirus | 274_0 | Clustered | VC_274_0 |
| Mycobacterium~phage~Bruin | Caudovirales | Siphoviridae | Kostyavirus | 311_0 | Clustered | VC_311_0 |
| Mycobacterium~phage~Brusacoram | Caudovirales | Siphoviridae | Fishburnevirus | 267_0 | Clustered | VC_267_0 |
| Mycobacterium~phage~Butters | Caudovirales | Siphoviridae | Buttersvirus | 87_0 | Clustered | VC_87_0 |
| Mycobacterium~phage~BuzzLyseyear | Caudovirales | Siphoviridae | Cheoctovirus | 274_2 | Clustered | VC_274_2 |
| Mycobacterium~phage~Bxz1 | Caudovirales | Myoviridae | Bxz1virus | 308_0 | Clustered | VC_308_0 |
| Mycobacterium~phage~CASbig | Caudovirales | Siphoviridae | Fromanvirus | 307_0 | Clustered | VC_307_0 |
| Mycobacterium~phage~CRB1 | Caudovirales | Siphoviridae | Fromanvirus | 283_2 | Clustered | VC_283_2 |
| Mycobacterium~phage~Cabrinians | Caudovirales | Siphoviridae | Cheoctovirus | 274_0 | Clustered | VC_274_0 |
| Mycobacterium~phage~Cali | Caudovirales | Myoviridae | Bxz1virus | 308_0 | Clustered | VC_308_0 |
| Mycobacterium~phage~Cambiare | Caudovirales | Siphoviridae | Liefievirus | 101_0 | Clustered | VC_101_0 |
| Mycobacterium~phage~CaptainTrips | Caudovirales | Siphoviridae | Cheoctovirus | 274_0 | Clustered | VC_274_0 |
| Mycobacterium~phage~Carcharodon | Caudovirales | Siphoviridae | Charlievirus | 87_0 | Clustered | VC_87_0 |
| Mycobacterium~phage~Catalina | Caudovirales | Siphoviridae | Fromanvirus | 283_0 | Clustered | VC_283_0 |
| Mycobacterium~phage~Catdawg | Caudovirales | Siphoviridae | Corndogvirus | 270_0 | Clustered | VC_270_0 |
| Mycobacterium~phage~Catera | Caudovirales | Myoviridae | Bxz1virus | 308_0 | Clustered | VC_308_0 |
| Mycobacterium~phage~Cerasum | Caudovirales | Siphoviridae | Cheoctovirus | 274_2 | Clustered | VC_274_2 |
| Mycobacterium~phage~Charlie | Caudovirales | Siphoviridae | Charlievirus | 87_0 | Clustered | VC_87_0 |
| Mycobacterium~phage~Cheetobro | Caudovirales | Siphoviridae | Timquatrovirus | 102_0 | Clustered | VC_102_0 |
| Mycobacterium~phage~Chy4 | Caudovirales | Siphoviridae | Fromanvirus | 283_2 | Clustered | VC_283_2 |
| Mycobacterium~phage~Chy5 | Caudovirales | Siphoviridae | Fromanvirus | 283_2 | Clustered | VC_283_2 |
| Mycobacterium~phage~CloudWang3 | Caudovirales | Siphoviridae | Fromanvirus | 283_1 | Clustered | VC_283_1 |
| Mycobacterium~phage~Colbert | Caudovirales | Siphoviridae | Pg1virus | 58_0 | Clustered | VC_58_0 |
| Mycobacterium~phage~Contagion | Caudovirales | Siphoviridae | Kostyavirus | 311_0 | Clustered | VC_311_0 |
| Mycobacterium~phage~Crossroads | Caudovirales | Siphoviridae | Bronvirus | 309_0 | Clustered | VC_309_0 |
| Mycobacterium~phage~DD5 | Caudovirales | Siphoviridae | Fromanvirus | 307_0 | Clustered | VC_307_0 |
| Mycobacterium~phage~Daenerys | Caudovirales | Siphoviridae | Cheoctovirus | 274_0 | Clustered | VC_274_0 |
| Mycobacterium~phage~Damien | Caudovirales | Siphoviridae | Barnyardvirus | 280_0 | Clustered | VC_280_0 |
| Mycobacterium~phage~Dandelion | Caudovirales | Myoviridae | Bxz1virus | 308_0 | Clustered | VC_308_0 |
| Mycobacterium~phage~Dante | Caudovirales | Siphoviridae | Cheoctovirus | 274_0 | Clustered | VC_274_0 |
| Mycobacterium~phage~Donovan | Caudovirales | Siphoviridae | Fishburnevirus | 267_0 | Clustered | VC_267_0 |
| Mycobacterium~phage~DrDrey | Caudovirales | Siphoviridae | Kostyavirus | 311_0 | Clustered | VC_311_0 |
| Mycobacterium~phage~Dreamboat | Caudovirales | Siphoviridae | Fromanvirus | 307_0 | Clustered | VC_307_0 |
| Mycobacterium~phage~Dumbo | Caudovirales | Siphoviridae | Kostyavirus | 311_0 | Clustered | VC_311_0 |
| Mycobacterium~phage~Dusk | Caudovirales | Siphoviridae | Kostyavirus | 311_0 | Clustered | VC_311_0 |
| Mycobacterium~phage~Dylan | Caudovirales | Siphoviridae | Corndogvirus | 270_0 | Clustered | VC_270_0 |
| Mycobacterium~phage~ET08 | Caudovirales | Myoviridae | Bxz1virus | 308_0 | Clustered | VC_308_0 |
| Mycobacterium~phage~EagleEye | Caudovirales | Siphoviridae | Fromanvirus | 283_2 | Clustered | VC_283_2 |
| Mycobacterium~phage~Echild | Caudovirales | Siphoviridae | Fromanvirus | 283_2 | Clustered | VC_283_2 |
| Mycobacterium~phage~Edtherson | Caudovirales | Siphoviridae | Fromanvirus | 307_0 | Clustered | VC_307_0 |
| Mycobacterium~phage~Enkosi | Caudovirales | Siphoviridae | Timquatrovirus | 102_2 | Clustered | VC_102_2 |
| Mycobacterium~phage~Equemioh13 | Caudovirales | Siphoviridae | Fromanvirus | 283_2 | Clustered | VC_283_2 |
| Mycobacterium~phage~Eremos | Caudovirales | Siphoviridae | Pg1virus | 58_0 | Clustered | VC_58_0 |
| Mycobacterium~phage~Estave1 | Caudovirales | Siphoviridae | Cheoctovirus | 274_3 | Clustered/Singleton | VC_274_3 |
| Mycobacterium~phage~EvilGenius | Caudovirales | Siphoviridae | Fromanvirus | 283_2 | Clustered | VC_283_2 |
| Mycobacterium~phage~First | Caudovirales | Siphoviridae | Fromanvirus | 283_2 | Clustered | VC_283_2 |
| Mycobacterium~phage~Fishburne | Caudovirales | Siphoviridae | Fishburnevirus | 267_0 | Clustered | VC_267_0 |
| Mycobacterium~phage~FlagStaff | Caudovirales | Siphoviridae | Liefievirus | 101_0 | Clustered | VC_101_0 |
| Mycobacterium~phage~Florinda | Caudovirales | Siphoviridae | Cheoctovirus | 274_0 | Clustered | VC_274_0 |
| Mycobacterium~phage~Gardann | Caudovirales | Siphoviridae | Bronvirus | 309_0 | Clustered | VC_309_0 |
| Mycobacterium~phage~Gengar | Caudovirales | Siphoviridae | Timquatrovirus | 102_1 | Clustered | VC_102_1 |
| Mycobacterium~phage~Gizmo | Caudovirales | Myoviridae | Bxz1virus | 308_0 | Clustered | VC_308_0 |
| Mycobacterium~phage~Goku | Caudovirales | Siphoviridae | Kostyavirus | 311_0 | Clustered | VC_311_0 |
| Mycobacterium~phage~Gompeii16 | Caudovirales | Siphoviridae | Fromanvirus | 307_0 | Clustered | VC_307_0 |
| Mycobacterium~phage~Graduation | Caudovirales | Siphoviridae | Fromanvirus | 307_0 | Clustered | VC_307_0 |
| Mycobacterium~phage~Gumball | Caudovirales | Siphoviridae | Pbi1virus | 279_0 | Clustered | VC_279_0 |
| Mycobacterium~phage~HINdeR | Caudovirales | Siphoviridae | Fromanvirus | 284_1 | Clustered | VC_284_1 |
| Mycobacterium~phage~Hades | Caudovirales | Siphoviridae | Cheoctovirus | 274_0 | Clustered | VC_274_0 |
| Mycobacterium~phage~Hamulus | Caudovirales | Siphoviridae | Cheoctovirus | 274_0 | Clustered | VC_274_0 |
| Mycobacterium~phage~HanShotFirst | Caudovirales | Siphoviridae | Fromanvirus | 307_0 | Clustered | VC_307_0 |
| Mycobacterium~phage~Hawkeye | Caudovirales | Siphoviridae | Hawkeyevirus | 279_0 | Clustered | VC_279_0 |
| Mycobacterium~phage~HufflyPuff | Caudovirales | Siphoviridae | Kostyavirus | 311_0 | Clustered | VC_311_0 |
| Mycobacterium~phage~HyRo | Caudovirales | Myoviridae | Bxz1virus | 308_0 | Clustered | VC_308_0 |
| Mycobacterium~phage~Inventum | Caudovirales | Siphoviridae | Cheoctovirus | 274_0 | Clustered | VC_274_0 |
| Mycobacterium~phage~Iracema64 | Caudovirales | Siphoviridae | Fromanvirus | 284_1 | Clustered | VC_284_1 |
| Mycobacterium~phage~Jabbawokkie | Caudovirales | Siphoviridae | Cheoctovirus | 273_0 | Clustered | VC_273_0 |
| Mycobacterium~phage~JacAttac | Caudovirales | Siphoviridae | Pg1virus | 58_0 | Clustered | VC_58_0 |
| Mycobacterium~phage~Jebeks | Caudovirales | Siphoviridae | Fishburnevirus | 267_0 | Clustered | VC_267_0 |
| Mycobacterium~phage~Job42 | Caudovirales | Siphoviridae | Cheoctovirus | 274_2 | Clustered | VC_274_2 |
| Mycobacterium~phage~Jobu08 | Caudovirales | Siphoviridae | Fromanvirus | 284_0 | Clustered | VC_284_0 |
| Mycobacterium~phage~Jolie2 | Caudovirales | Siphoviridae | Liefievirus | 101_0 | Clustered | VC_101_0 |
| Mycobacterium~phage~Kampy | Caudovirales | Siphoviridae | Fromanvirus | 284_1 | Clustered | VC_284_1 |
| Mycobacterium~phage~Keshu | Caudovirales | Siphoviridae | Timquatrovirus | 102_2 | Clustered | VC_102_2 |
| Mycobacterium~phage~Kikipoo | Caudovirales | Siphoviridae | Pg1virus | 58_0 | Clustered | VC_58_0 |
| Mycobacterium~phage~Kimberlium | Caudovirales | Siphoviridae | Cheoctovirus | 274_0 | Clustered | VC_274_0 |
| Mycobacterium~phage~Konstantine | Caudovirales | Siphoviridae | Barnyardvirus | 280_0 | Clustered | VC_280_0 |
| Mycobacterium~phage~Kratio | Caudovirales | Siphoviridae | Timquatrovirus | 102_1 | Clustered | VC_102_1 |
| Mycobacterium~phage~LadyBird | Caudovirales | Siphoviridae | Fromanvirus | 283_2 | Clustered | VC_283_2 |
| Mycobacterium~phage~Lamina13 | Caudovirales | Siphoviridae | Fromanvirus | 307_0 | Clustered | VC_307_0 |
| Mycobacterium~phage~Larenn | Caudovirales | Siphoviridae | Fromanvirus | 283_2 | Clustered | VC_283_2 |
| Mycobacterium~phage~Leo | Caudovirales | Siphoviridae | Liefievirus | 101_0 | Clustered | VC_101_0 |
| Mycobacterium~phage~Lilac | Caudovirales | Siphoviridae | Kostyavirus | 311_0 | Clustered | VC_311_0 |
| Mycobacterium~phage~LinStu | Caudovirales | Myoviridae | Bxz1virus | 308_0 | Clustered | VC_308_0 |
| Mycobacterium~phage~Llama | Caudovirales | Siphoviridae | Cheoctovirus | 274_0 | Clustered | VC_274_0 |
| Mycobacterium~phage~Lolly9 | Caudovirales | Siphoviridae | Bronvirus | 309_0 | Clustered | VC_309_0 |
| Mycobacterium~phage~Loser | Caudovirales | Siphoviridae | Fromanvirus | 283_2 | Clustered | VC_283_2 |
| Mycobacterium~phage~Luchador | Caudovirales | Siphoviridae | Fromanvirus | 283_2 | Clustered | VC_283_2 |
| Mycobacterium~phage~Lukilu | Caudovirales | Myoviridae | Bixzunavirus | 308_0 | Clustered | VC_308_0 |
| Mycobacterium~phage~MOOREtheMARYer | Caudovirales | Siphoviridae | Liefievirus | 101_0 | Clustered | VC_101_0 |
| Mycobacterium~phage~Makemake | Caudovirales | Siphoviridae | Fromanvirus | 307_0 | Clustered | VC_307_0 |
| Mycobacterium~phage~Malithi | Caudovirales | Siphoviridae | Fishburnevirus | 267_0 | Clustered | VC_267_0 |
| Mycobacterium~phage~Manad | Caudovirales | Siphoviridae | Pg1virus | 58_0 | Clustered | VC_58_0 |
| Mycobacterium~phage~MarQuardt | Caudovirales | Siphoviridae | Fromanvirus | 284_0 | Clustered | VC_284_0 |
| Mycobacterium~phage~Marvin | Caudovirales | Siphoviridae | Marvinvirus | 312_0 | Clustered | VC_312_0 |
| Mycobacterium~phage~MiaZeal | Caudovirales | Siphoviridae | Omegavirus | 272_0 | Clustered | VC_272_0 |
| Mycobacterium~phage~MichelleMyBell | Caudovirales | Siphoviridae | Buttersvirus | 87_0 | Clustered | VC_87_0 |
| Mycobacterium~phage~Milly | Caudovirales | Siphoviridae | Timquatrovirus | 102_2 | Clustered | VC_102_2 |
| Mycobacterium~phage~Mindy | Caudovirales | Siphoviridae | Kostyavirus | 311_0 | Clustered | VC_311_0 |
| Mycobacterium~phage~Minerva | Caudovirales | Siphoviridae | Omegavirus | 272_1 | Clustered | VC_272_1 |
| Mycobacterium~phage~MoMoMixon | Caudovirales | Myoviridae | Bxz1virus | 308_0 | Clustered | VC_308_0 |
| Mycobacterium~phage~MosMoris | Caudovirales | Siphoviridae | Marvinvirus | 312_0 | Clustered | VC_312_0 |
| Mycobacterium~phage~Muddy | Caudovirales | Siphoviridae | Ff47virus | 276_0 | Clustered | VC_276_0 |
| Mycobacterium~phage~Mufasa | Caudovirales | Siphoviridae | Timquatrovirus | 102_2 | Clustered | VC_102_2 |
| Mycobacterium~phage~Mulciber | Caudovirales | Siphoviridae | Fromanvirus | 283_2 | Clustered | VC_283_2 |
| Mycobacterium~phage~Murphy | Caudovirales | Siphoviridae | Kostyavirus | 311_0 | Clustered | VC_311_0 |
| Mycobacterium~phage~Murucutumbu | Caudovirales | Siphoviridae | Timquatrovirus | 102_2 | Clustered | VC_102_2 |
| Mycobacterium~phage~Nala | Caudovirales | Siphoviridae | Kostyavirus | 311_0 | Clustered | VC_311_0 |
| Mycobacterium~phage~Nappy | Caudovirales | Myoviridae | Bxz1virus | 308_0 | Clustered | VC_308_0 |
| Mycobacterium~phage~NelitzaMV | Caudovirales | Siphoviridae | Kostyavirus | 311_0 | Clustered | VC_311_0 |
| Mycobacterium~phage~Nerujay | Caudovirales | Siphoviridae | Fromanvirus | 307_0 | Clustered | VC_307_0 |
| Mycobacterium~phage~Newman | Caudovirales | Siphoviridae | Pg1virus | 58_0 | Clustered | VC_58_0 |
| Mycobacterium~phage~Nhonho | Caudovirales | Siphoviridae | Fromanvirus | 307_0 | Clustered | VC_307_0 |
| Mycobacterium~phage~Nyxis | Caudovirales | Siphoviridae | Fromanvirus | 284_1 | Clustered | VC_284_1 |
| Mycobacterium~phage~OSmaximus | Caudovirales | Siphoviridae | Pg1virus | 58_0 | Clustered | VC_58_0 |
| Mycobacterium~phage~Oaker | Caudovirales | Siphoviridae | Barnyardvirus | 280_0 | Clustered | VC_280_0 |
| Mycobacterium~phage~Obama12 | Caudovirales | Siphoviridae | L5virus | 284_1 | Clustered | VC_284_1 |
| Mycobacterium~phage~OkiRoe | Caudovirales | Siphoviridae | Timquatrovirus | 102_1 | Clustered | VC_102_1 |
| Mycobacterium~phage~Oline | Caudovirales | Siphoviridae | Pg1virus | 58_0 | Clustered | VC_58_0 |
| Mycobacterium~phage~Omnicron | Caudovirales | Siphoviridae | Timquatrovirus | 102_1 | Clustered | VC_102_1 |
| Mycobacterium~phage~Orion | Caudovirales | Siphoviridae | Pg1virus | 58_0 | Clustered | VC_58_0 |
| Mycobacterium~phage~Ovechkin | Caudovirales | Siphoviridae | Cheoctovirus | 274_0 | Clustered | VC_274_0 |
| Mycobacterium~phage~PBI1 | Caudovirales | Siphoviridae | Pbi1virus | 279_0 | Clustered | VC_279_0 |
| Mycobacterium~phage~PG1 | Caudovirales | Siphoviridae | Pg1virus | 58_0 | Clustered | VC_58_0 |
| Mycobacterium~phage~PLot | Caudovirales | Siphoviridae | Plotvirus | 279_0 | Clustered | VC_279_0 |
| Mycobacterium~phage~Panchino | Caudovirales | Siphoviridae | Redivirus | 87_0 | Clustered | VC_87_0 |
| Mycobacterium~phage~Papez | Caudovirales | Siphoviridae | Fromanvirus | 307_0 | Clustered | VC_307_0 |
| Mycobacterium~phage~Papyrus | Caudovirales | Siphoviridae | Send513virus | 281_0 | Clustered | VC_281_0 |
| Mycobacterium~phage~Pari | Caudovirales | Siphoviridae | Fromanvirus | 307_0 | Clustered | VC_307_0 |
| Mycobacterium~phage~Patience | Caudovirales | Siphoviridae | Patiencevirus | 280_0 | Clustered | VC_280_0 |
| Mycobacterium~phage~PattyP | Caudovirales | Siphoviridae | Fromanvirus | 307_0 | Clustered | VC_307_0 |
| Mycobacterium~phage~PegLeg | Caudovirales | Siphoviridae | Bongovirus | 310_0 | Clustered | VC_310_0 |
| Mycobacterium~phage~Pepe | Caudovirales | Siphoviridae | Fromanvirus | 307_0 | Clustered | VC_307_0 |
| Mycobacterium~phage~Phantastic | Caudovirales | Siphoviridae | Fromanvirus | 284_0 | Clustered | VC_284_0 |
| Mycobacterium~phage~PhatBacter | Caudovirales | Siphoviridae | Kostyavirus | 311_0 | Clustered | VC_311_0 |
| Mycobacterium~phage~Phatniss | Caudovirales | Siphoviridae | Cheoctovirus | 274_0 | Clustered | VC_274_0 |
| Mycobacterium~phage~Phaux | Caudovirales | Siphoviridae | Kostyavirus | 311_0 | Clustered | VC_311_0 |
| Mycobacterium~phage~Phayonce | Caudovirales | Siphoviridae | Phayoncevirus | 267_0 | Clustered | VC_267_0 |
| Mycobacterium~phage~Phipps | Caudovirales | Siphoviridae | Pg1virus | 58_0 | Clustered | VC_58_0 |
| Mycobacterium~phage~Phlei | Caudovirales | Siphoviridae | Fromanvirus | 283_2 | Clustered | VC_283_2 |
| Mycobacterium~phage~Phrann | Caudovirales | Siphoviridae | Redivirus | 87_0 | Clustered | VC_87_0 |
| Mycobacterium~phage~PhrostyMug | Caudovirales | Siphoviridae | Fromanvirus | 307_0 | Clustered | VC_307_0 |
| Mycobacterium~phage~Phrux | Caudovirales | Siphoviridae | Kostyavirus | 311_0 | Clustered | VC_311_0 |
| Mycobacterium~phage~Pinto | Caudovirales | Siphoviridae | Fromanvirus | 307_0 | Clustered | VC_307_0 |
| Mycobacterium~phage~Pioneer | Caudovirales | Siphoviridae | Fromanvirus | 283_0 | Clustered | VC_283_0 |
| Mycobacterium~phage~Pipsqueaks | Caudovirales | Siphoviridae | Charlievirus | 87_0 | Clustered | VC_87_0 |
| Mycobacterium~phage~Piro94 | Caudovirales | Siphoviridae | Fromanvirus | 283_2 | Clustered | VC_283_2 |
| Mycobacterium~phage~Pleione | Caudovirales | Myoviridae | Bxz1virus | 308_0 | Clustered | VC_308_0 |
| Mycobacterium~phage~PopTart | Caudovirales | Siphoviridae | Cheoctovirus | 274_0 | Clustered | VC_274_0 |
| Mycobacterium~phage~Pops | Caudovirales | Siphoviridae | Pg1virus | 58_0 | Clustered | VC_58_0 |
| Mycobacterium~phage~Predator | Caudovirales | Siphoviridae | Barnyardvirus | 280_0 | Clustered | VC_280_0 |
| Mycobacterium~phage~Quico | Caudovirales | Siphoviridae | Cheoctovirus | 274_0 | Clustered | VC_274_0 |
| Mycobacterium~phage~Quink | Caudovirales | Siphoviridae | Kostyavirus | 311_0 | Clustered | VC_311_0 |
| Mycobacterium~phage~RedRock | Caudovirales | Siphoviridae | L5virus | 283_2 | Clustered | VC_283_2 |
| Mycobacterium~phage~Redi | Caudovirales | Siphoviridae | Redivirus | 87_0 | Clustered | VC_87_0 |
| Mycobacterium~phage~Redno2 | Caudovirales | Siphoviridae | Omegavirus | 272_2 | Clustered/Singleton | VC_272_2 |
| Mycobacterium~phage~Rey | Caudovirales | Siphoviridae | Reyvirus | 310_0 | Clustered | VC_310_0 |
| Mycobacterium~phage~RhynO | Caudovirales | Siphoviridae | Fromanvirus | 284_1 | Clustered | VC_284_1 |
| Mycobacterium~phage~RidgeCB | Caudovirales | Siphoviridae | L5virus | 307_0 | Clustered | VC_307_0 |
| Mycobacterium~phage~Rizal | Caudovirales | Myoviridae | Bxz1virus | 308_0 | Clustered | VC_308_0 |
| Mycobacterium~phage~Rockstar | Caudovirales | Siphoviridae | L5virus | 284_0 | Clustered | VC_284_0 |
| Mycobacterium~phage~Rufus | Caudovirales | Siphoviridae | Fromanvirus | 307_0 | Clustered | VC_307_0 |
| Mycobacterium~phage~SWU1 | Caudovirales | Siphoviridae | L5virus | 283_2 | Clustered | VC_283_2 |
| Mycobacterium~phage~Saal | Caudovirales | Siphoviridae | Cheoctovirus | 274_0 | Clustered | VC_274_0 |
| Mycobacterium~phage~SarFire | Caudovirales | Siphoviridae | Fromanvirus | 307_0 | Clustered | VC_307_0 |
| Mycobacterium~phage~ScottMcG | Caudovirales | Myoviridae | Bxz1virus | 308_0 | Clustered | VC_308_0 |
| Mycobacterium~phage~Seabiscuit | Caudovirales | Siphoviridae | Fromanvirus | 307_0 | Clustered | VC_307_0 |
| Mycobacterium~phage~Seagreen | Caudovirales | Siphoviridae | Cheoctovirus | 274_0 | Clustered | VC_274_0 |
| Mycobacterium~phage~Sebata | Caudovirales | Myoviridae | Bxz1virus | 308_0 | Clustered | VC_308_0 |
| Mycobacterium~phage~Send513 | Caudovirales | Siphoviridae | Send513virus | 281_0 | Clustered | VC_281_0 |
| Mycobacterium~phage~Serenity | Caudovirales | Siphoviridae | Fromanvirus | 283_2 | Clustered | VC_283_2 |
| Mycobacterium~phage~Severus | Caudovirales | Siphoviridae | Fromanvirus | 284_1 | Clustered | VC_284_1 |
| Mycobacterium~phage~Shauna1 | Caudovirales | Siphoviridae | Che8virus | 274_0 | Clustered | VC_274_0 |
| Mycobacterium~phage~ShedlockHolmes | Caudovirales | Siphoviridae | Timquatrovirus | 102_2 | Clustered | VC_102_2 |
| Mycobacterium~phage~Sheen | Caudovirales | Siphoviridae | Fromanvirus | 284_1 | Clustered | VC_284_1 |
| Mycobacterium~phage~ShiLan | Caudovirales | Siphoviridae | Che8virus | 274_0 | Clustered | VC_274_0 |
| Mycobacterium~phage~ShiVal | Caudovirales | Siphoviridae | Pg1virus | 58_0 | Clustered | VC_58_0 |
| Mycobacterium~phage~Shipwreck | Caudovirales | Siphoviridae | Fishburnevirus | 267_0 | Clustered | VC_267_0 |
| Mycobacterium~phage~SiSi | Caudovirales | Siphoviridae | Cheoctovirus | 274_0 | Clustered | VC_274_0 |
| Mycobacterium~phage~SirDuracell | Caudovirales | Siphoviridae | Cjw1virus | 311_0 | Clustered | VC_311_0 |
| Mycobacterium~phage~SkiPole | Caudovirales | Siphoviridae | L5virus | 307_0 | Clustered | VC_307_0 |
| Mycobacterium~phage~SkinnyPete | Caudovirales | Siphoviridae | Redivirus | 87_0 | Clustered | VC_87_0 |
| Mycobacterium~phage~Sneeze | Caudovirales | Siphoviridae | Liefievirus | 101_0 | Clustered | VC_101_0 |
| Mycobacterium~phage~Snenia | Caudovirales | Siphoviridae | Bronvirus | 309_0 | Clustered | VC_309_0 |
| Mycobacterium~phage~Solon | Caudovirales | Siphoviridae | L5virus | 307_0 | Clustered | VC_307_0 |
| Mycobacterium~phage~Soto | Caudovirales | Siphoviridae | Pg1virus | 58_0 | Clustered | VC_58_0 |
| Mycobacterium~phage~Sparkdehlily | Caudovirales | Siphoviridae | Cheoctovirus | 274_0 | Clustered | VC_274_0 |
| Mycobacterium~phage~Sparky | Caudovirales | Siphoviridae | Unassigned | 269_1 | Clustered/Singleton | VC_269_1 |
| Mycobacterium~phage~Spartacus | Caudovirales | Siphoviridae | Che8virus | 274_0 | Clustered | VC_274_0 |
| Mycobacterium~phage~Spud | Caudovirales | Myoviridae | Bxz1virus | 308_0 | Clustered | VC_308_0 |
| Mycobacterium~phage~Squirty | Caudovirales | Siphoviridae | Cheoctovirus | 274_4 | Clustered/Singleton | VC_274_4 |
| Mycobacterium~phage~Suffolk | Caudovirales | Siphoviridae | Pg1virus | 58_0 | Clustered | VC_58_0 |
| Mycobacterium~phage~SweetiePie | Caudovirales | Siphoviridae | Fromanvirus | 283_2 | Clustered | VC_283_2 |
| Mycobacterium~phage~Swish | Caudovirales | Siphoviridae | Pg1virus | 58_0 | Clustered | VC_58_0 |
| Mycobacterium~phage~Switzer | Caudovirales | Siphoviridae | L5virus | 307_0 | Clustered | VC_307_0 |
| Mycobacterium~phage~TM4 | Caudovirales | Siphoviridae | Tm4virus | 102_2 | Clustered | VC_102_2 |
| Mycobacterium~phage~Taj | Caudovirales | Siphoviridae | Che8virus | 274_0 | Clustered | VC_274_0 |
| Mycobacterium~phage~Tasp14 | Caudovirales | Siphoviridae | Fromanvirus | 307_0 | Clustered | VC_307_0 |
| Mycobacterium~phage~TheloniousMonk | Caudovirales | Siphoviridae | Fromanvirus | 307_0 | Clustered | VC_307_0 |
| Mycobacterium~phage~Thibault | Caudovirales | Siphoviridae | Omegavirus | 272_3 | Clustered/Singleton | VC_272_3 |
| Mycobacterium~phage~Tiffany | Caudovirales | Siphoviridae | Fromanvirus | 284_0 | Clustered | VC_284_0 |
| Mycobacterium~phage~Timshel | Caudovirales | Siphoviridae | L5virus | 284_1 | Clustered | VC_284_1 |
| Mycobacterium~phage~Tonenili | Caudovirales | Myoviridae | Bixzunavirus | 308_0 | Clustered | VC_308_0 |
| Mycobacterium~phage~Toto | Caudovirales | Siphoviridae | Cjw1virus | 311_0 | Clustered | VC_311_0 |
| Mycobacterium~phage~Trike | Caudovirales | Siphoviridae | Fromanvirus | 284_1 | Clustered | VC_284_1 |
| Mycobacterium~phage~Trixie | Caudovirales | Siphoviridae | L5virus | 283_2 | Clustered | VC_283_2 |
| Mycobacterium~phage~Troll4 | Caudovirales | Siphoviridae | Pbi1virus | 279_0 | Clustered | VC_279_0 |
| Mycobacterium~phage~Trouble | Caudovirales | Siphoviridae | Fromanvirus | 307_0 | Clustered | VC_307_0 |
| Mycobacterium~phage~Turbido | Caudovirales | Siphoviridae | L5virus | 283_2 | Clustered | VC_283_2 |
| Mycobacterium~phage~Turj99 | Caudovirales | Siphoviridae | Fromanvirus | 307_0 | Clustered | VC_307_0 |
| Mycobacterium~phage~Tweety | Caudovirales | Siphoviridae | Che8virus | 274_0 | Clustered | VC_274_0 |
| Mycobacterium~phage~Twister | Caudovirales | Siphoviridae | L5virus | 284_1 | Clustered | VC_284_1 |
| Mycobacterium~phage~U2 | Caudovirales | Siphoviridae | L5virus | 307_0 | Clustered | VC_307_0 |
| Mycobacterium~phage~UncleHowie | Caudovirales | Siphoviridae | Pg1virus | 58_0 | Clustered | VC_58_0 |
| Mycobacterium~phage~Validus | Caudovirales | Siphoviridae | Timquatrovirus | 102_2 | Clustered | VC_102_2 |
| Mycobacterium~phage~Velveteen | Caudovirales | Siphoviridae | Cheoctovirus | 274_2 | Clustered | VC_274_2 |
| Mycobacterium~phage~Violet | Caudovirales | Siphoviridae | L5virus | 307_0 | Clustered | VC_307_0 |
| Mycobacterium~phage~Vista | Caudovirales | Siphoviridae | Pg1virus | 58_0 | Clustered | VC_58_0 |
| Mycobacterium~phage~VohminGhazi | Caudovirales | Siphoviridae | Fromanvirus | 283_1 | Clustered | VC_283_1 |
| Mycobacterium~phage~Vortex | Caudovirales | Siphoviridae | Pegunavirus | 58_0 | Clustered | VC_58_0 |
| Mycobacterium~phage~WIVsmall | Caudovirales | Siphoviridae | Cheoctovirus | 274_1 | Clustered | VC_274_1 |
| Mycobacterium~phage~Wanda | Caudovirales | Siphoviridae | Omegavirus | 272_1 | Clustered | VC_272_1 |
| Mycobacterium~phage~Wee | Caudovirales | Siphoviridae | Che8virus | 274_0 | Clustered | VC_274_0 |
| Mycobacterium~phage~Wheeler | Caudovirales | Siphoviridae | Fromanvirus | 307_0 | Clustered | VC_307_0 |
| Mycobacterium~phage~Whirlwind | Caudovirales | Siphoviridae | Bronvirus | 309_0 | Clustered | VC_309_0 |
| Mycobacterium~phage~Wile | Caudovirales | Siphoviridae | Fromanvirus | 284_1 | Clustered | VC_284_1 |
| Mycobacterium~phage~XFactor | Caudovirales | Siphoviridae | Cheoctovirus | 274_0 | Clustered | VC_274_0 |
| Mycobacterium~phage~Xeno | Caudovirales | Siphoviridae | Charlievirus | 87_0 | Clustered | VC_87_0 |
| Mycobacterium~phage~Zaka | Caudovirales | Siphoviridae | Fromanvirus | 283_1 | Clustered | VC_283_1 |
| Mycobacterium~phage~ZoeJ | Caudovirales | Siphoviridae | Timquatrovirus | 102_2 | Clustered | VC_102_2 |
| Mycobacterium~phage~vB_MapS_FF47 | Caudovirales | Siphoviridae | Ff47virus | 276_0 | Clustered | VC_276_0 |
| Mycobacterium~virus~244 | Caudovirales | Siphoviridae | Cjw1virus | 311_0 | Clustered | VC_311_0 |
| Mycobacterium~virus~Alma | Caudovirales | Siphoviridae | L5virus | 283_0 | Clustered | VC_283_0 |
| Mycobacterium~virus~Anaya | Caudovirales | Siphoviridae | Tm4virus | 102_2 | Clustered | VC_102_2 |
| Mycobacterium~virus~Angelica | Caudovirales | Siphoviridae | Tm4virus | 102_2 | Clustered | VC_102_2 |
| Mycobacterium~virus~Ardmore | Caudovirales | Siphoviridae | Che8virus | 274_0 | Clustered | VC_274_0 |
| Mycobacterium~virus~Avani | Caudovirales | Siphoviridae | Che8virus | 273_0 | Clustered | VC_273_0 |
| Mycobacterium~virus~Bask21 | Caudovirales | Siphoviridae | Cjw1virus | 311_0 | Clustered | VC_311_0 |
| Mycobacterium~virus~Bethlehem | Caudovirales | Siphoviridae | L5virus | 307_0 | Clustered | VC_307_0 |
| Mycobacterium~virus~Billknuckles | Caudovirales | Siphoviridae | L5virus | 307_0 | Clustered | VC_307_0 |
| Mycobacterium~virus~Boomer | Caudovirales | Siphoviridae | Che8virus | 274_0 | Clustered | VC_274_0 |
| Mycobacterium~virus~Bron | Caudovirales | Siphoviridae | Bronvirus | 309_0 | Clustered | VC_309_0 |
| Mycobacterium~virus~Bruns | Caudovirales | Siphoviridae | L5virus | 307_0 | Clustered | VC_307_0 |
| Mycobacterium~virus~Bxb1 | Caudovirales | Siphoviridae | L5virus | 307_0 | Clustered | VC_307_0 |
| Mycobacterium~virus~Bxz2 | Caudovirales | Siphoviridae | L5virus | 284_0 | Clustered | VC_284_0 |
| Mycobacterium~virus~CJW1 | Caudovirales | Siphoviridae | Cjw1virus | 311_0 | Clustered | VC_311_0 |
| Mycobacterium~virus~Che12 | Caudovirales | Siphoviridae | L5virus | 283_2 | Clustered | VC_283_2 |
| Mycobacterium~virus~Che8 | Caudovirales | Siphoviridae | Che8virus | 274_0 | Clustered | VC_274_0 |
| Mycobacterium~virus~Che9d | Caudovirales | Siphoviridae | Che8virus | 273_0 | Clustered | VC_273_0 |
| Mycobacterium~virus~Corndog | Caudovirales | Siphoviridae | Corndogvirus | 270_0 | Clustered | VC_270_0 |
| Mycobacterium~virus~Courthouse | Caudovirales | Siphoviridae | Omegavirus | 272_0 | Clustered | VC_272_0 |
| Mycobacterium~virus~Crimd | Caudovirales | Siphoviridae | Tm4virus | 102_2 | Clustered | VC_102_2 |
| Mycobacterium~virus~D29 | Caudovirales | Siphoviridae | L5virus | 283_2 | Clustered | VC_283_2 |
| Mycobacterium~virus~Deadp | Caudovirales | Siphoviridae | Che8virus | 274_0 | Clustered | VC_274_0 |
| Mycobacterium~virus~Doom | Caudovirales | Siphoviridae | L5virus | 307_0 | Clustered | VC_307_0 |
| Mycobacterium~virus~Dorothy | Caudovirales | Siphoviridae | Che8virus | 274_0 | Clustered | VC_274_0 |
| Mycobacterium~virus~Drago | Caudovirales | Siphoviridae | Che8virus | 274_0 | Clustered | VC_274_0 |
| Mycobacterium~virus~Euphoria | Caudovirales | Siphoviridae | L5virus | 307_0 | Clustered | VC_307_0 |
| Mycobacterium~virus~Eureka | Caudovirales | Siphoviridae | Cjw1virus | 311_0 | Clustered | VC_311_0 |
| Mycobacterium~virus~Faith1 | Caudovirales | Siphoviridae | Bronvirus | 309_0 | Clustered | VC_309_0 |
| Mycobacterium~virus~Fionnbarth | Caudovirales | Siphoviridae | Tm4virus | 102_0 | Clustered | VC_102_0 |
| Mycobacterium~virus~Firecracker | Caudovirales | Siphoviridae | Corndogvirus | 270_0 | Clustered | VC_270_0 |
| Mycobacterium~virus~Fruitloop | Caudovirales | Siphoviridae | Che8virus | 274_0 | Clustered | VC_274_0 |
| Mycobacterium~virus~Gumbie | Caudovirales | Siphoviridae | Che8virus | 274_0 | Clustered | VC_274_0 |
| Mycobacterium~virus~Halo | Caudovirales | Siphoviridae | Liefievirus | 101_0 | Clustered | VC_101_0 |
| Mycobacterium~virus~Jasper | Caudovirales | Siphoviridae | L5virus | 307_0 | Clustered | VC_307_0 |
| Mycobacterium~virus~KBG | Caudovirales | Siphoviridae | L5virus | 307_0 | Clustered | VC_307_0 |
| Mycobacterium~virus~Kostya | Caudovirales | Siphoviridae | Cjw1virus | 311_0 | Clustered | VC_311_0 |
| Mycobacterium~virus~Kugel | Caudovirales | Siphoviridae | L5virus | 307_0 | Clustered | VC_307_0 |
| Mycobacterium~virus~L5 | Caudovirales | Siphoviridae | L5virus | 283_2 | Clustered | VC_283_2 |
| Mycobacterium~virus~Larva | Caudovirales | Siphoviridae | Tm4virus | 102_1 | Clustered | VC_102_1 |
| Mycobacterium~virus~Liefie | Caudovirales | Siphoviridae | Liefievirus | 101_0 | Clustered | VC_101_0 |
| Mycobacterium~virus~Llij | Caudovirales | Siphoviridae | Che8virus | 274_0 | Clustered | VC_274_0 |
| Mycobacterium~virus~Marcell | Caudovirales | Siphoviridae | L5virus | 307_0 | Clustered | VC_307_0 |
| Mycobacterium~virus~Mutaforma13 | Caudovirales | Siphoviridae | Che8virus | 274_0 | Clustered | VC_274_0 |
| Mycobacterium~virus~Nepal | Caudovirales | Siphoviridae | L5virus | 307_0 | Clustered | VC_307_0 |
| Mycobacterium~virus~Omega | Caudovirales | Siphoviridae | Omegavirus | 272_4 | Clustered/Singleton | VC_272_4 |
| Mycobacterium~virus~Optimus | Caudovirales | Siphoviridae | Omegavirus | 272_1 | Clustered | VC_272_1 |
| Mycobacterium~virus~PMC | Caudovirales | Siphoviridae | Che8virus | 274_0 | Clustered | VC_274_0 |
| Mycobacterium~virus~Pacc40 | Caudovirales | Siphoviridae | Che8virus | 274_0 | Clustered | VC_274_0 |
| Mycobacterium~virus~Peaches | Caudovirales | Siphoviridae | L5virus | 284_1 | Clustered | VC_284_1 |
| Mycobacterium~virus~Perseus | Caudovirales | Siphoviridae | L5virus | 307_0 | Clustered | VC_307_0 |
| Mycobacterium~virus~Porky | Caudovirales | Siphoviridae | Cjw1virus | 311_0 | Clustered | VC_311_0 |
| Mycobacterium~virus~Pukovnik | Caudovirales | Siphoviridae | L5virus | 283_2 | Clustered | VC_283_2 |
| Mycobacterium~virus~Pumpkin | Caudovirales | Siphoviridae | Cjw1virus | 311_0 | Clustered | VC_311_0 |
| Mycobacterium~virus~Ramsey | Caudovirales | Siphoviridae | Che8virus | 274_2 | Clustered | VC_274_2 |
| Mycobacterium~virus~Rumpelstiltskin | Caudovirales | Siphoviridae | Bronvirus | 309_0 | Clustered | VC_309_0 |
| Mycobacterium~virus~SG4 | Caudovirales | Siphoviridae | Che8virus | 274_0 | Clustered | VC_274_0 |
| Mycobacterium~virus~Wonder | Caudovirales | Siphoviridae | L5virus | 284_0 | Clustered | VC_284_0 |
| Mycobacterium~virus~Yoshi | Caudovirales | Siphoviridae | Che8virus | 273_0 | Clustered | VC_273_0 |
| Mycobacterium~virus~lockley | Caudovirales | Siphoviridae | L5virus | 307_0 | Clustered | VC_307_0 |
| Mycoplasma~phage~MAV1 | Unassigned | Unassigned | Unassigned | 313_0 | Clustered | VC_313_0 |
| Mycoplasma~phage~phiMFV1 | Unassigned | Unassigned | Unassigned | 313_0 | Clustered | VC_313_0 |
| NIFA_virome_10698 | Unassigned | Unassigned | Unassigned | 69_0 | Clustered | VC_69_0 |
| NIFA_virome_10923 | Unassigned | Unassigned | Unassigned | 314_0 | Clustered | VC_314_0 |
| NIFA_virome_12105 | Unassigned | Unassigned | Unassigned | 315_0 | Clustered | VC_315_0 |
| NIFA_virome_16073 | Unassigned | Unassigned | Unassigned | 316_0 | Clustered | VC_316_0 |
| NIFA_virome_1612 | Unassigned | Unassigned | Unassigned | 314_0 | Clustered | VC_314_0 |
| NIFA_virome_17346 | Unassigned | Unassigned | Unassigned | 317_0 | Clustered | VC_317_0 |
| NIFA_virome_30030 | Unassigned | Unassigned | Unassigned | 317_0 | Clustered | VC_317_0 |
| NIFA_virome_357 | Unassigned | Unassigned | Unassigned | 318_0 | Clustered | VC_318_0 |
| NIFA_virome_47580 | Unassigned | Unassigned | Unassigned | 317_0 | Clustered | VC_317_0 |
| NIFA_virome_49231 | Unassigned | Unassigned | Unassigned | 320_0 | Clustered | VC_320_0 |
| NIFA_virome_51733 | Unassigned | Unassigned | Unassigned | 321_0 | Clustered | VC_321_0 |
| NIFA_virome_51951 | Unassigned | Unassigned | Unassigned | 317_0 | Clustered | VC_317_0 |
| NIFA_virome_52668 | Unassigned | Unassigned | Unassigned | 319_0 | Clustered | VC_319_0 |
| NIFA_virome_55160 | Unassigned | Unassigned | Unassigned | 315_0 | Clustered | VC_315_0 |
| NIFA_virome_57519 | Unassigned | Unassigned | Unassigned | 322_0 | Clustered | VC_322_0 |
| NIFA_virome_60613 | Unassigned | Unassigned | Unassigned | 323_0 | Clustered | VC_323_0 |
| NIFA_virome_60823 | Unassigned | Unassigned | Unassigned | 324_0 | Clustered | VC_324_0 |
| NIFA_virome_60831 | Unassigned | Unassigned | Unassigned | 325_0 | Clustered | VC_325_0 |
| NIFA_virome_60872 | Unassigned | Unassigned | Unassigned | 326_0 | Clustered | VC_326_0 |
| NIFA_virome_61188 | Unassigned | Unassigned | Unassigned | 225_1 | Clustered/Singleton | VC_225_1 |
| NIFA_virome_61249 | Unassigned | Unassigned | Unassigned | 108_0 | Clustered | VC_108_0 |
| NIFA_virome_61313 | Unassigned | Unassigned | Unassigned | 69_0 | Clustered | VC_69_0 |
| NIFA_virome_61321 | Unassigned | Unassigned | Unassigned | 326_0 | Clustered | VC_326_0 |
| NIFA_virome_61322 | Unassigned | Unassigned | Unassigned | 327_0 | Clustered | VC_327_0 |
| NIFA_virome_61451 | Unassigned | Unassigned | Unassigned | 316_0 | Clustered | VC_316_0 |
| NIFA_virome_61533 | Unassigned | Unassigned | Unassigned | 328_0 | Clustered | VC_328_0 |
| NIFA_virome_61686 | Unassigned | Unassigned | Unassigned | 327_0 | Clustered | VC_327_0 |
| NIFA_virome_61802 | Unassigned | Unassigned | Unassigned | 236_0 | Clustered | VC_236_0 |
| NIFA_virome_61844 | Unassigned | Unassigned | Unassigned | 329_0 | Clustered | VC_329_0 |
| NIFA_virome_61872 | Unassigned | Unassigned | Unassigned | 161_0 | Clustered | VC_161_0 |
| NIFA_virome_61978 | Unassigned | Unassigned | Unassigned | 330_0 | Clustered | VC_330_0 |
| NIFA_virome_62084 | Unassigned | Unassigned | Unassigned | 321_0 | Clustered | VC_321_0 |
| NIFA_virome_62106 | Unassigned | Unassigned | Unassigned | 331_0 | Clustered | VC_331_0 |
| NIFA_virome_62169 | Unassigned | Unassigned | Unassigned | 116_0 | Clustered | VC_116_0 |
| NIFA_virome_62211 | Unassigned | Unassigned | Unassigned | 329_0 | Clustered | VC_329_0 |
| NIFA_virome_62399 | Unassigned | Unassigned | Unassigned | 236_0 | Clustered | VC_236_0 |
| NIFA_virome_62409 | Unassigned | Unassigned | Unassigned | 330_0 | Clustered | VC_330_0 |
| NIFA_virome_62479 | Unassigned | Unassigned | Unassigned | 248_0 | Clustered | VC_248_0 |
| NIFA_virome_62939 | Unassigned | Unassigned | Unassigned | 325_0 | Clustered | VC_325_0 |
| NIFA_virome_62964 | Unassigned | Unassigned | Unassigned | 164_0 | Clustered | VC_164_0 |
| NIFA_virome_62967 | Unassigned | Unassigned | Unassigned | 334_0 | Clustered | VC_334_0 |
| NIFA_virome_62993 | Unassigned | Unassigned | Unassigned | 335_0 | Clustered | VC_335_0 |
| NIFA_virome_63029 | Unassigned | Unassigned | Unassigned | 335_0 | Clustered | VC_335_0 |
| NIFA_virome_63105 | Unassigned | Unassigned | Unassigned | 336_0 | Clustered | VC_336_0 |
| NIFA_virome_63121 | Unassigned | Unassigned | Unassigned | 161_0 | Clustered | VC_161_0 |
| NIFA_virome_63181 | Unassigned | Unassigned | Unassigned | 116_0 | Clustered | VC_116_0 |
| NIFA_virome_63901 | Unassigned | Unassigned | Unassigned | 331_0 | Clustered | VC_331_0 |
| NIFA_virome_63920 | Unassigned | Unassigned | Unassigned | 334_0 | Clustered | VC_334_0 |
| NIFA_virome_63943 | Unassigned | Unassigned | Unassigned | 323_0 | Clustered | VC_323_0 |
| NIFA_virome_63944 | Unassigned | Unassigned | Unassigned | 322_0 | Clustered | VC_322_0 |
| NIFA_virome_64073 | Unassigned | Unassigned | Unassigned | 55_0 | Clustered | VC_55_0 |
| NIFA_virome_64080 | Unassigned | Unassigned | Unassigned | 328_0 | Clustered | VC_328_0 |
| NIFA_virome_64091 | Unassigned | Unassigned | Unassigned | 337_0 | Clustered | VC_337_0 |
| NIFA_virome_64136 | Unassigned | Unassigned | Unassigned | 324_0 | Clustered | VC_324_0 |
| NIFA_virome_64179 | Unassigned | Unassigned | Unassigned | 336_0 | Clustered | VC_336_0 |
| NIFA_virome_8907 | Unassigned | Unassigned | Unassigned | 320_0 | Clustered | VC_320_0 |
| NIFA_virome_9264 | Unassigned | Unassigned | Unassigned | 319_0 | Clustered | VC_319_0 |
| Nonlabens~phage~P12024L | Caudovirales | Siphoviridae | P12024virus | 338_0 | Clustered | VC_338_0 |
| Nonlabens~phage~P12024S | Caudovirales | Siphoviridae | P12024virus | 338_0 | Clustered | VC_338_0 |
| Oenococcus~phage~phi9805 | Unassigned | Unassigned | Unassigned | 150_0 | Clustered | VC_150_0 |
| Oenococcus~phage~phiS11 | Unassigned | Unassigned | Unassigned | 150_0 | Clustered | VC_150_0 |
| Oenococcus~phage~phiS13 | Unassigned | Unassigned | Unassigned | 150_0 | Clustered | VC_150_0 |
| Paenibacillus~phage~Diva | Caudovirales | Siphoviridae | Sitaravirus | 122_0 | Clustered | VC_122_0 |
| Paenibacillus~phage~Fern | Caudovirales | Siphoviridae | Sitaravirus | 122_0 | Clustered | VC_122_0 |
| Paenibacillus~phage~HB10c2 | Caudovirales | Siphoviridae | Sitaravirus | 122_0 | Clustered | VC_122_0 |
| Paenibacillus~phage~Rani | Caudovirales | Siphoviridae | Sitaravirus | 122_0 | Clustered | VC_122_0 |
| Paenibacillus~phage~Shelly | Caudovirales | Siphoviridae | Sitaravirus | 122_0 | Clustered | VC_122_0 |
| Paenibacillus~phage~Sitara | Caudovirales | Siphoviridae | Sitaravirus | 122_0 | Clustered | VC_122_0 |
| Paenibacillus~phage~Tripp | Caudovirales | Siphoviridae | Trippvirus | 137_0 | Clustered | VC_137_0 |
| Paenibacillus~phage~Willow | Caudovirales | Siphoviridae | Sitaravirus | 122_0 | Clustered | VC_122_0 |
| Paenibacillus~phage~Xenia | Caudovirales | Siphoviridae | Sitaravirus | 122_0 | Clustered | VC_122_0 |
| Paenibacillus~phage~phiIBB_Pl23 | Caudovirales | Siphoviridae | Sitaravirus | 122_0 | Clustered | VC_122_0 |
| Pantoea~phage~LIMElight | Caudovirales | Podoviridae | Phikmvvirus | 31_0 | Clustered | VC_31_0 |
| Pantoea~phage~vB_PagS_Vid5 | Caudovirales | Siphoviridae | Vidquintavirus | 14_1 | Clustered | VC_14_1 |
| Paracoccus~phage~vB_PmaS-R3 | Caudovirales | Siphoviridae | Unassigned | 1_0 | Clustered | VC_1_0 |
| Pasteurella~virus~F108 | Caudovirales | Myoviridae | Hp1virus | 70_0 | Clustered | VC_70_0 |
| Pectobacterium~bacteriophage~PM2 | Caudovirales | Myoviridae | Unassigned | 34_3 | Clustered/Singleton | VC_34_3 |
| Pectobacterium~phage~CBB | Caudovirales | Myoviridae | Eneladusvirus | 72_1 | Clustered/Singleton | VC_72_1 |
| Pectobacterium~phage~My1 | Caudovirales | Siphoviridae | Myunavirus | 261_2 | Clustered | VC_261_2 |
| Pectobacterium~phage~PM1 | Caudovirales | Myoviridae | Unassigned | 59_0 | Clustered | VC_59_0 |
| Pectobacterium~phage~PP1 | Caudovirales | Podoviridae | Zindervirus | 254_0 | Clustered | VC_254_0 |
| Pectobacterium~phage~PP16 | Caudovirales | Podoviridae | Phimunavirus | 31_0 | Clustered | VC_31_0 |
| Pectobacterium~phage~PP90 | Caudovirales | Podoviridae | Phimunavirus | 31_0 | Clustered | VC_31_0 |
| Pectobacterium~phage~Peat1 | Caudovirales | Podoviridae | Phimunavirus | 31_0 | Clustered | VC_31_0 |
| Pectobacterium~phage~PhiM1 | Caudovirales | Podoviridae | Phimunavirus | 31_0 | Clustered | VC_31_0 |
| Pectobacterium~phage~phiTE | Caudovirales | Myoviridae | Cr3virus | 225_2 | Clustered/Singleton | VC_225_2 |
| Pectobacterium~phage~vB_PatP_CB5 | Caudovirales | Podoviridae | Phimunavirus | 31_0 | Clustered | VC_31_0 |
| Pediococcus~virus~cIP1 | Caudovirales | Siphoviridae | Phijl1virus | 175_0 | Clustered | VC_175_0 |
| Phormidium~phage~MIS-PhV1A | Unassigned | Unassigned | Unassigned | 339_0 | Clustered | VC_339_0 |
| Phormidium~phage~MIS-PhV1B | Unassigned | Unassigned | Unassigned | 339_0 | Clustered | VC_339_0 |
| Phormidium~virus~WMP3 | Caudovirales | Podoviridae | Unassigned | 80_0 | Clustered | VC_80_0 |
| Polaribacter~phage~P12002L | Caudovirales | Siphoviridae | P12002virus | 333_0 | Clustered | VC_333_0 |
| Polaribacter~phage~P12002S | Caudovirales | Siphoviridae | P12002virus | 333_0 | Clustered | VC_333_0 |
| Prochlorococcus~phage~MED4-184 | Caudovirales | Myoviridae | Unassigned | 237_0 | Clustered | VC_237_0 |
| Prochlorococcus~phage~MED4-213 | Caudovirales | Myoviridae | Unassigned | 25_0 | Clustered | VC_25_0 |
| Prochlorococcus~phage~P-GSP1 | Caudovirales | Podoviridae | Unassigned | 211_0 | Clustered | VC_211_0 |
| Prochlorococcus~phage~P-HM1 | Caudovirales | Myoviridae | Unassigned | 25_0 | Clustered | VC_25_0 |
| Prochlorococcus~phage~P-HM2 | Caudovirales | Myoviridae | Unassigned | 25_0 | Clustered | VC_25_0 |
| Prochlorococcus~phage~P-RSM4 | Caudovirales | Myoviridae | Unassigned | 25_10 | Clustered/Singleton | VC_25_10 |
| Prochlorococcus~phage~P-SSM2 | Caudovirales | Myoviridae | Unassigned | 25_24 | Clustered/Singleton | VC_25_24 |
| Prochlorococcus~phage~P-SSM3 | Caudovirales | Myoviridae | Unassigned | 25_8 | Clustered | VC_25_8 |
| Prochlorococcus~phage~P-SSM4 | Caudovirales | Myoviridae | Unassigned | 25_8 | Clustered | VC_25_8 |
| Prochlorococcus~phage~P-SSM7 | Caudovirales | Myoviridae | Unassigned | 25_12 | Clustered/Singleton | VC_25_12 |
| Prochlorococcus~phage~P-SSP10 | Caudovirales | Podoviridae | Unassigned | 211_0 | Clustered | VC_211_0 |
| Prochlorococcus~phage~P-SSP3 | Caudovirales | Podoviridae | Unassigned | 211_0 | Clustered | VC_211_0 |
| Prochlorococcus~phage~P-TIM68 | Caudovirales | Myoviridae | Unassigned | 25_21 | Clustered/Singleton | VC_25_21 |
| Prochlorococcus~phage~Syn1 | Caudovirales | Myoviridae | Unassigned | 25_15 | Clustered/Singleton | VC_25_15 |
| Prochlorococcus~phage~Syn33 | Caudovirales | Myoviridae | Unassigned | 25_2 | Clustered | VC_25_2 |
| Prochlorococcus~virus~PSSP7 | Caudovirales | Podoviridae | Unassigned | 211_0 | Clustered | VC_211_0 |
| Propionibacterium~phage~ATCC29399B_C | Caudovirales | Siphoviridae | Pa6virus | 340_0 | Clustered | VC_340_0 |
| Propionibacterium~phage~ATCC29399B_T | Caudovirales | Siphoviridae | Pa6virus | 340_0 | Clustered | VC_340_0 |
| Propionibacterium~phage~Anatole | Caudovirales | Siphoviridae | Anatolevirus | 88_0 | Clustered | VC_88_0 |
| Propionibacterium~phage~Attacne | Caudovirales | Siphoviridae | Pa6virus | 340_0 | Clustered | VC_340_0 |
| Propionibacterium~phage~B22 | Caudovirales | Siphoviridae | Doucettevirus | 88_0 | Clustered | VC_88_0 |
| Propionibacterium~phage~B3 | Caudovirales | Siphoviridae | Anatolevirus | 88_0 | Clustered | VC_88_0 |
| Propionibacterium~phage~BruceLethal | Caudovirales | Siphoviridae | Pahexavirus | 340_0 | Clustered | VC_340_0 |
| Propionibacterium~phage~Doucette | Caudovirales | Siphoviridae | Doucettevirus | 88_0 | Clustered | VC_88_0 |
| Propionibacterium~phage~E6 | Caudovirales | Siphoviridae | Doucettevirus | 88_0 | Clustered | VC_88_0 |
| Propionibacterium~phage~Enoki | Caudovirales | Siphoviridae | Pahexavirus | 340_0 | Clustered | VC_340_0 |
| Propionibacterium~phage~G4 | Caudovirales | Siphoviridae | Doucettevirus | 88_0 | Clustered | VC_88_0 |
| Propionibacterium~phage~Keiki | Caudovirales | Siphoviridae | Pa6virus | 340_0 | Clustered | VC_340_0 |
| Propionibacterium~phage~Kubed | Caudovirales | Siphoviridae | Pa6virus | 340_0 | Clustered | VC_340_0 |
| Propionibacterium~phage~Lauchelly | Caudovirales | Siphoviridae | Pa6virus | 340_0 | Clustered | VC_340_0 |
| Propionibacterium~phage~Moyashi | Caudovirales | Siphoviridae | Pahexavirus | 340_0 | Clustered | VC_340_0 |
| Propionibacterium~phage~MrAK | Caudovirales | Siphoviridae | Pa6virus | 340_0 | Clustered | VC_340_0 |
| Propionibacterium~phage~Ouroboros | Caudovirales | Siphoviridae | Pa6virus | 340_0 | Clustered | VC_340_0 |
| Propionibacterium~phage~P1.1 | Caudovirales | Siphoviridae | Pa6virus | 340_0 | Clustered | VC_340_0 |
| Propionibacterium~phage~P100D | Caudovirales | Siphoviridae | Pa6virus | 340_0 | Clustered | VC_340_0 |
| Propionibacterium~phage~P100_1 | Caudovirales | Siphoviridae | Pa6virus | 340_0 | Clustered | VC_340_0 |
| Propionibacterium~phage~P100_A | Caudovirales | Siphoviridae | Pa6virus | 340_0 | Clustered | VC_340_0 |
| Propionibacterium~phage~P101A | Caudovirales | Siphoviridae | Pa6virus | 340_0 | Clustered | VC_340_0 |
| Propionibacterium~phage~P104A | Caudovirales | Siphoviridae | Pa6virus | 340_0 | Clustered | VC_340_0 |
| Propionibacterium~phage~P105 | Caudovirales | Siphoviridae | Pa6virus | 340_0 | Clustered | VC_340_0 |
| Propionibacterium~phage~P14.4 | Caudovirales | Siphoviridae | Pa6virus | 340_0 | Clustered | VC_340_0 |
| Propionibacterium~phage~P9.1 | Caudovirales | Siphoviridae | Pa6virus | 340_0 | Clustered | VC_340_0 |
| Propionibacterium~phage~PA1-14 | Caudovirales | Siphoviridae | Pahexavirus | 340_0 | Clustered | VC_340_0 |
| Propionibacterium~phage~PA6 | Caudovirales | Siphoviridae | Pa6virus | 340_0 | Clustered | VC_340_0 |
| Propionibacterium~phage~PAC1 | Caudovirales | Siphoviridae | Pahexavirus | 340_0 | Clustered | VC_340_0 |
| Propionibacterium~phage~PAD20 | Caudovirales | Siphoviridae | Pa6virus | 340_0 | Clustered | VC_340_0 |
| Propionibacterium~phage~PAS50 | Caudovirales | Siphoviridae | Pa6virus | 340_0 | Clustered | VC_340_0 |
| Propionibacterium~phage~PHL009M11 | Caudovirales | Siphoviridae | Pa6virus | 340_0 | Clustered | VC_340_0 |
| Propionibacterium~phage~PHL010M04 | Caudovirales | Siphoviridae | Pahexavirus | 340_0 | Clustered | VC_340_0 |
| Propionibacterium~phage~PHL025M00 | Caudovirales | Siphoviridae | Pa6virus | 340_0 | Clustered | VC_340_0 |
| Propionibacterium~phage~PHL030N00 | Caudovirales | Siphoviridae | Pahexavirus | 340_0 | Clustered | VC_340_0 |
| Propionibacterium~phage~PHL037M02 | Caudovirales | Siphoviridae | Pa6virus | 340_0 | Clustered | VC_340_0 |
| Propionibacterium~phage~PHL041M10 | Caudovirales | Siphoviridae | Pa6virus | 340_0 | Clustered | VC_340_0 |
| Propionibacterium~phage~PHL055N00 | Caudovirales | Siphoviridae | Pahexavirus | 340_0 | Clustered | VC_340_0 |
| Propionibacterium~phage~PHL060L00 | Caudovirales | Siphoviridae | Pa6virus | 340_0 | Clustered | VC_340_0 |
| Propionibacterium~phage~PHL067M01 | Caudovirales | Siphoviridae | Pa6virus | 340_0 | Clustered | VC_340_0 |
| Propionibacterium~phage~PHL067M10 | Caudovirales | Siphoviridae | Pahexavirus | 340_0 | Clustered | VC_340_0 |
| Propionibacterium~phage~PHL070N00 | Caudovirales | Siphoviridae | Pa6virus | 340_0 | Clustered | VC_340_0 |
| Propionibacterium~phage~PHL071N05 | Caudovirales | Siphoviridae | Pa6virus | 340_0 | Clustered | VC_340_0 |
| Propionibacterium~phage~PHL082M00 | Caudovirales | Siphoviridae | Pahexavirus | 340_0 | Clustered | VC_340_0 |
| Propionibacterium~phage~PHL082M03 | Caudovirales | Siphoviridae | Pa6virus | 340_0 | Clustered | VC_340_0 |
| Propionibacterium~phage~PHL085N00 | Caudovirales | Siphoviridae | Pahexavirus | 340_0 | Clustered | VC_340_0 |
| Propionibacterium~phage~PHL092M00 | Caudovirales | Siphoviridae | Pa6virus | 340_0 | Clustered | VC_340_0 |
| Propionibacterium~phage~PHL095N00 | Caudovirales | Siphoviridae | Pa6virus | 340_0 | Clustered | VC_340_0 |
| Propionibacterium~phage~PHL111M01 | Caudovirales | Siphoviridae | Pa6virus | 340_0 | Clustered | VC_340_0 |
| Propionibacterium~phage~PHL112N00 | Caudovirales | Siphoviridae | Pa6virus | 340_0 | Clustered | VC_340_0 |
| Propionibacterium~phage~PHL113M01 | Caudovirales | Siphoviridae | Pa6virus | 340_0 | Clustered | VC_340_0 |
| Propionibacterium~phage~PHL114L00 | Caudovirales | Siphoviridae | Pa6virus | 340_0 | Clustered | VC_340_0 |
| Propionibacterium~phage~PHL116M00 | Caudovirales | Siphoviridae | Pa6virus | 340_0 | Clustered | VC_340_0 |
| Propionibacterium~phage~PHL117M00 | Caudovirales | Siphoviridae | Pa6virus | 340_0 | Clustered | VC_340_0 |
| Propionibacterium~phage~PHL117M01 | Caudovirales | Siphoviridae | Pa6virus | 340_0 | Clustered | VC_340_0 |
| Propionibacterium~phage~PHL132N00 | Caudovirales | Siphoviridae | Pa6virus | 340_0 | Clustered | VC_340_0 |
| Propionibacterium~phage~PHL141N00 | Caudovirales | Siphoviridae | Pa6virus | 340_0 | Clustered | VC_340_0 |
| Propionibacterium~phage~PHL150M00 | Caudovirales | Siphoviridae | Pahexavirus | 340_0 | Clustered | VC_340_0 |
| Propionibacterium~phage~PHL151M00 | Caudovirales | Siphoviridae | Pa6virus | 340_0 | Clustered | VC_340_0 |
| Propionibacterium~phage~PHL151N00 | Caudovirales | Siphoviridae | Pa6virus | 340_0 | Clustered | VC_340_0 |
| Propionibacterium~phage~PHL152M00 | Caudovirales | Siphoviridae | Pa6virus | 340_0 | Clustered | VC_340_0 |
| Propionibacterium~phage~PHL163M00 | Caudovirales | Siphoviridae | Pa6virus | 340_0 | Clustered | VC_340_0 |
| Propionibacterium~phage~PHL171M01 | Caudovirales | Siphoviridae | Pa6virus | 340_0 | Clustered | VC_340_0 |
| Propionibacterium~phage~PHL179M00 | Caudovirales | Siphoviridae | Pa6virus | 340_0 | Clustered | VC_340_0 |
| Propionibacterium~phage~PHL194M00 | Caudovirales | Siphoviridae | Pa6virus | 340_0 | Clustered | VC_340_0 |
| Propionibacterium~phage~PHL199M00 | Caudovirales | Siphoviridae | Pa6virus | 340_0 | Clustered | VC_340_0 |
| Propionibacterium~phage~PHL301M00 | Caudovirales | Siphoviridae | Pa6virus | 340_0 | Clustered | VC_340_0 |
| Propionibacterium~phage~PHL308M00 | Caudovirales | Siphoviridae | Pa6virus | 340_0 | Clustered | VC_340_0 |
| Propionibacterium~phage~Pacnes~2012-15 | Caudovirales | Siphoviridae | Pa6virus | 340_0 | Clustered | VC_340_0 |
| Propionibacterium~phage~Pirate | Caudovirales | Siphoviridae | Pa6virus | 340_0 | Clustered | VC_340_0 |
| Propionibacterium~phage~Procrass1 | Caudovirales | Siphoviridae | Pa6virus | 340_0 | Clustered | VC_340_0 |
| Propionibacterium~phage~QueenBey | Caudovirales | Siphoviridae | Pahexavirus | 340_0 | Clustered | VC_340_0 |
| Propionibacterium~phage~SKKY | Caudovirales | Siphoviridae | Pa6virus | 340_0 | Clustered | VC_340_0 |
| Propionibacterium~phage~Solid | Caudovirales | Siphoviridae | Pa6virus | 340_0 | Clustered | VC_340_0 |
| Propionibacterium~phage~Stormborn | Caudovirales | Siphoviridae | Pa6virus | 340_0 | Clustered | VC_340_0 |
| Propionibacterium~phage~Wizzo | Caudovirales | Siphoviridae | Pa6virus | 340_0 | Clustered | VC_340_0 |
| Proteus~phage~PM~75 | Caudovirales | Podoviridae | Unassigned | 31_0 | Clustered | VC_31_0 |
| Proteus~phage~PM~85 | Caudovirales | Podoviridae | Zindervirus | 254_0 | Clustered | VC_254_0 |
| Proteus~phage~PM~93 | Caudovirales | Podoviridae | Zindervirus | 254_0 | Clustered | VC_254_0 |
| Proteus~phage~PM135 | Caudovirales | Siphoviridae | Novosibvirus | 261_2 | Clustered | VC_261_2 |
| Proteus~phage~PM16 | Caudovirales | Podoviridae | Unassigned | 31_0 | Clustered | VC_31_0 |
| Proteus~phage~VB_PmiS-Isfahan | Caudovirales | Siphoviridae | Nonanavirus | 245_0 | Clustered | VC_245_0 |
| Proteus~phage~pPM_01 | Caudovirales | Siphoviridae | Chivirus | 15_0 | Clustered | VC_15_0 |
| Proteus~phage~vB_PmiP_Pm5460 | Caudovirales | Podoviridae | Zindervirus | 254_0 | Clustered | VC_254_0 |
| Providencia~phage~Redjac | Caudovirales | Siphoviridae | Chivirus | 15_0 | Clustered | VC_15_0 |
| Providencia~phage~vB_PreS_PR1 | Caudovirales | Siphoviridae | Priunavirus | 261_2 | Clustered | VC_261_2 |
| Pseudoalteromonas~phage~BS5 | Caudovirales | Siphoviridae | Unassigned | 44_0 | Clustered | VC_44_0 |
| Pseudoalteromonas~phage~Cr39582 | Unassigned | Corticoviridae | Corticovirus | 342_0 | Clustered | VC_342_0 |
| Pseudoalteromonas~phage~H103 | Caudovirales | Siphoviridae | Unassigned | 60_0 | Clustered | VC_60_0 |
| Pseudoalteromonas~phage~H105/1 | Caudovirales | Siphoviridae | Unassigned | 44_0 | Clustered | VC_44_0 |
| Pseudoalteromonas~phage~PH1 | Caudovirales | Podoviridae | Kafunavirus | 243_0 | Clustered | VC_243_0 |
| Pseudoalteromonas~virus~PM2 | Unassigned | Corticoviridae | Corticovirus | 342_0 | Clustered | VC_342_0 |
| Pseudomonad~phage~gh-1 | Caudovirales | Podoviridae | T7virus | 210_0 | Clustered | VC_210_0 |
| Pseudomonas~phage~14-1 | Caudovirales | Myoviridae | Pbunavirus | 186_0 | Clustered | VC_186_0 |
| Pseudomonas~phage~201phi2-1 | Caudovirales | Myoviridae | Phikzvirus | 230_1 | Clustered/Singleton | VC_230_1 |
| Pseudomonas~phage~73 | Caudovirales | Siphoviridae | Septima3virus | 1_0 | Clustered | VC_1_0 |
| Pseudomonas~phage~AF | Caudovirales | Podoviridae | Unassigned | 161_0 | Clustered | VC_161_0 |
| Pseudomonas~phage~Andromeda | Caudovirales | Podoviridae | Bifseptvirus | 67_0 | Clustered | VC_67_0 |
| Pseudomonas~phage~B3 | Caudovirales | Siphoviridae | Beetrevirus | 189_0 | Clustered | VC_189_0 |
| Pseudomonas~phage~Bf7 | Caudovirales | Podoviridae | Bifseptvirus | 67_0 | Clustered | VC_67_0 |
| Pseudomonas~phage~Bjorn | Caudovirales | Podoviridae | Bjornvirus | 213_0 | Clustered | VC_213_0 |
| Pseudomonas~phage~C11 | Caudovirales | Myoviridae | Pakpunavirus | 46_0 | Clustered | VC_46_0 |
| Pseudomonas~phage~DL54 | Caudovirales | Podoviridae | Bruynoghevirus | 213_0 | Clustered | VC_213_0 |
| Pseudomonas~phage~DL60 | Caudovirales | Myoviridae | Pbunavirus | 186_0 | Clustered | VC_186_0 |
| Pseudomonas~phage~DL62 | Caudovirales | Podoviridae | Phikmvvirus | 43_0 | Clustered | VC_43_0 |
| Pseudomonas~phage~DL64 | Caudovirales | Podoviridae | Litunavirus | 13_0 | Clustered | VC_13_0 |
| Pseudomonas~phage~DL68 | Caudovirales | Myoviridae | Pbunavirus | 186_0 | Clustered | VC_186_0 |
| Pseudomonas~phage~EL | Caudovirales | Myoviridae | Elvirus | 231_1 | Clustered | VC_231_1 |
| Pseudomonas~phage~F10 | Caudovirales | Siphoviridae | Unassigned | 249_0 | Clustered | VC_249_0 |
| Pseudomonas~phage~F8 | Caudovirales | Myoviridae | Pbunavirus | 186_0 | Clustered | VC_186_0 |
| Pseudomonas~phage~H70 | Caudovirales | Siphoviridae | Casadabanvirus | 190_0 | Clustered | VC_190_0 |
| Pseudomonas~phage~JBD18 | Caudovirales | Siphoviridae | Beetrevirus | 189_0 | Clustered | VC_189_0 |
| Pseudomonas~phage~JBD24 | Caudovirales | Siphoviridae | Casadabanvirus | 190_0 | Clustered | VC_190_0 |
| Pseudomonas~phage~JBD25 | Caudovirales | Siphoviridae | Unassigned | 189_0 | Clustered | VC_189_0 |
| Pseudomonas~phage~JBD30 | Caudovirales | Siphoviridae | Casadabanvirus | 190_0 | Clustered | VC_190_0 |
| Pseudomonas~phage~JBD44 | Caudovirales | Siphoviridae | Unassigned | 41_1 | Clustered/Singleton | VC_41_1 |
| Pseudomonas~phage~JBD5 | Caudovirales | Siphoviridae | Casadabanvirus | 190_0 | Clustered | VC_190_0 |
| Pseudomonas~phage~JBD67 | Caudovirales | Siphoviridae | Beetrevirus | 189_0 | Clustered | VC_189_0 |
| Pseudomonas~phage~JBD69 | Caudovirales | Siphoviridae | Casadabanvirus | 190_0 | Clustered | VC_190_0 |
| Pseudomonas~phage~JBD88a | Caudovirales | Siphoviridae | Casadabanvirus | 190_0 | Clustered | VC_190_0 |
| Pseudomonas~phage~JBD93 | Caudovirales | Siphoviridae | Casadabanvirus | 190_0 | Clustered | VC_190_0 |
| Pseudomonas~phage~JD024 | Caudovirales | Siphoviridae | Casadabanvirus | 190_0 | Clustered | VC_190_0 |
| Pseudomonas~phage~JG004 | Caudovirales | Myoviridae | Pakpunavirus | 46_0 | Clustered | VC_46_0 |
| Pseudomonas~phage~JG024 | Caudovirales | Myoviridae | Pbunavirus | 186_0 | Clustered | VC_186_0 |
| Pseudomonas~phage~K5 | Caudovirales | Myoviridae | Pakpunavirus | 46_0 | Clustered | VC_46_0 |
| Pseudomonas~phage~K8 | Caudovirales | Myoviridae | Pakpunavirus | 46_0 | Clustered | VC_46_0 |
| Pseudomonas~phage~KPP12 | Caudovirales | Myoviridae | Pbunavirus | 186_0 | Clustered | VC_186_0 |
| Pseudomonas~phage~KPP21 | Caudovirales | Podoviridae | Luz7virus | 8_0 | Clustered | VC_8_0 |
| Pseudomonas~phage~KPP25 | Caudovirales | Podoviridae | Kpp25virus | 344_0 | Clustered | VC_344_0 |
| Pseudomonas~phage~LBL3 | Caudovirales | Myoviridae | Pbunavirus | 186_0 | Clustered | VC_186_0 |
| Pseudomonas~phage~LIT1 | Caudovirales | Podoviridae | Lit1virus | 13_0 | Clustered | VC_13_0 |
| Pseudomonas~phage~LKD16 | Caudovirales | Podoviridae | Phikmvvirus | 43_0 | Clustered | VC_43_0 |
| Pseudomonas~phage~LKO4 | Caudovirales | Siphoviridae | Yuavirus | 2_0 | Clustered | VC_2_0 |
| Pseudomonas~phage~LMA2 | Caudovirales | Myoviridae | Pbunavirus | 186_0 | Clustered | VC_186_0 |
| Pseudomonas~phage~LUZ19 | Caudovirales | Podoviridae | Phikmvvirus | 43_0 | Clustered | VC_43_0 |
| Pseudomonas~phage~LUZ7 | Caudovirales | Podoviridae | Luz7virus | 8_0 | Clustered | VC_8_0 |
| Pseudomonas~phage~Lu11 | Caudovirales | Myoviridae | Unassigned | 345_0 | Clustered | VC_345_0 |
| Pseudomonas~phage~MD8 | Caudovirales | Siphoviridae | Unassigned | 249_0 | Clustered | VC_249_0 |
| Pseudomonas~phage~MP1412 | Caudovirales | Siphoviridae | Yuavirus | 2_0 | Clustered | VC_2_0 |
| Pseudomonas~phage~MP42 | Caudovirales | Siphoviridae | Casadabanvirus | 190_0 | Clustered | VC_190_0 |
| Pseudomonas~phage~MP48 | Caudovirales | Siphoviridae | Casadabanvirus | 190_0 | Clustered | VC_190_0 |
| Pseudomonas~phage~MPK6 | Caudovirales | Podoviridae | Phikmvvirus | 43_0 | Clustered | VC_43_0 |
| Pseudomonas~phage~MPK7 | Caudovirales | Podoviridae | Phikmvvirus | 43_0 | Clustered | VC_43_0 |
| Pseudomonas~phage~NH-4 | Caudovirales | Myoviridae | Pbunavirus | 186_0 | Clustered | VC_186_0 |
| Pseudomonas~phage~NP1 | Caudovirales | Siphoviridae | Np1virus | 3_0 | Clustered | VC_3_0 |
| Pseudomonas~phage~NV1 | Caudovirales | Podoviridae | Vicosavirus | 213_0 | Clustered | VC_213_0 |
| Pseudomonas~phage~Noxifer | Caudovirales | Myoviridae | Noxifervirus | 230_3 | Clustered/Singleton | VC_230_3 |
| Pseudomonas~phage~O4 | Unassigned | Unassigned | Unassigned | 199_0 | Clustered | VC_199_0 |
| Pseudomonas~phage~OBP | Caudovirales | Myoviridae | Unassigned | 231_2 | Clustered/Singleton | VC_231_2 |
| Pseudomonas~phage~PA10 | Caudovirales | Myoviridae | Pakpunavirus | 46_0 | Clustered | VC_46_0 |
| Pseudomonas~phage~PA11 | Caudovirales | Myoviridae | Unassigned | 199_0 | Clustered | VC_199_0 |
| Pseudomonas~phage~PA26 | Caudovirales | Podoviridae | Lit1virus | 13_0 | Clustered | VC_13_0 |
| Pseudomonas~phage~PA5 | Caudovirales | Myoviridae | Pbunavirus | 186_0 | Clustered | VC_186_0 |
| Pseudomonas~phage~PA7 | Caudovirales | Myoviridae | Phikzvirus | 230_0 | Clustered | VC_230_0 |
| Pseudomonas~phage~PAE1 | Caudovirales | Siphoviridae | Yuavirus | 2_0 | Clustered | VC_2_0 |
| Pseudomonas~phage~PAJU2 | Caudovirales | Siphoviridae | Unassigned | 41_3 | Clustered/Singleton | VC_41_3 |
| Pseudomonas~phage~PAK_P1 | Caudovirales | Myoviridae | Pakpunavirus | 46_0 | Clustered | VC_46_0 |
| Pseudomonas~phage~PAK_P2 | Caudovirales | Myoviridae | Pakpunavirus | 46_0 | Clustered | VC_46_0 |
| Pseudomonas~phage~PAK_P4 | Caudovirales | Myoviridae | Pakpunavirus | 46_0 | Clustered | VC_46_0 |
| Pseudomonas~phage~PB1 | Caudovirales | Myoviridae | Pbunavirus | 186_0 | Clustered | VC_186_0 |
| Pseudomonas~phage~PEV2 | Caudovirales | Podoviridae | Lit1virus | 13_0 | Clustered | VC_13_0 |
| Pseudomonas~phage~PP7 | Unassigned | Leviviridae | Unassigned | 198_0 | Clustered | VC_198_0 |
| Pseudomonas~phage~PPPL-1 | Caudovirales | Podoviridae | Teseptimavirus | 210_0 | Clustered | VC_210_0 |
| Pseudomonas~phage~PPpW-3 | Caudovirales | Myoviridae | Jilinvirus | 62_0 | Clustered | VC_62_0 |
| Pseudomonas~phage~PPpW-4 | Caudovirales | Podoviridae | Teseptimavirus | 210_0 | Clustered | VC_210_0 |
| Pseudomonas~phage~PRR1 | Unassigned | Leviviridae | Levivirus | 197_0 | Clustered | VC_197_0 |
| Pseudomonas~phage~PT2 | Caudovirales | Podoviridae | Phikmvvirus | 43_0 | Clustered | VC_43_0 |
| Pseudomonas~phage~Pa2 | Caudovirales | Podoviridae | Litunavirus | 13_0 | Clustered | VC_13_0 |
| Pseudomonas~phage~PaBG | Caudovirales | Myoviridae | Unassigned | 345_0 | Clustered | VC_345_0 |
| Pseudomonas~phage~PaMx25 | Caudovirales | Siphoviridae | Np1virus | 3_0 | Clustered | VC_3_0 |
| Pseudomonas~phage~PaMx42 | Caudovirales | Siphoviridae | Septimatrevirus | 1_0 | Clustered | VC_1_0 |
| Pseudomonas~phage~PaP1 | Caudovirales | Myoviridae | Pakpunavirus | 46_0 | Clustered | VC_46_0 |
| Pseudomonas~phage~PaP2 | Caudovirales | Podoviridae | Unassigned | 332_0 | Clustered | VC_332_0 |
| Pseudomonas~phage~PaoP5 | Caudovirales | Myoviridae | Pakpunavirus | 46_0 | Clustered | VC_46_0 |
| Pseudomonas~phage~Pf-10 | Caudovirales | Podoviridae | Teseptimavirus | 210_0 | Clustered | VC_210_0 |
| Pseudomonas~phage~Phi-S1 | Caudovirales | Podoviridae | Teseptimavirus | 210_0 | Clustered | VC_210_0 |
| Pseudomonas~phage~PhiCHU | Caudovirales | Podoviridae | Luz24virus | 213_0 | Clustered | VC_213_0 |
| Pseudomonas~phage~PhiPA3 | Caudovirales | Myoviridae | Phikzvirus | 230_2 | Clustered/Singleton | VC_230_2 |
| Pseudomonas~phage~PollyC | Caudovirales | Podoviridae | Pollyceevirus | 67_0 | Clustered | VC_67_0 |
| Pseudomonas~phage~SL2 | Caudovirales | Myoviridae | Phikzvirus | 230_0 | Clustered | VC_230_0 |
| Pseudomonas~phage~SN | Caudovirales | Myoviridae | Pbunavirus | 186_0 | Clustered | VC_186_0 |
| Pseudomonas~phage~TL | Caudovirales | Podoviridae | Luz24virus | 213_0 | Clustered | VC_213_0 |
| Pseudomonas~phage~UFV-P2 | Caudovirales | Podoviridae | Vicosavirus | 213_0 | Clustered | VC_213_0 |
| Pseudomonas~phage~VSW-3 | Caudovirales | Podoviridae | Napahaivirus | 67_0 | Clustered | VC_67_0 |
| Pseudomonas~phage~YH30 | Caudovirales | Podoviridae | Litunavirus | 13_0 | Clustered | VC_13_0 |
| Pseudomonas~phage~YH6 | Caudovirales | Podoviridae | Litunavirus | 13_0 | Clustered | VC_13_0 |
| Pseudomonas~phage~YMC11/02/R656 | Caudovirales | Siphoviridae | Unassigned | 41_4 | Clustered/Singleton | VC_41_4 |
| Pseudomonas~phage~YMC11/06/C171_PPU_BP | Caudovirales | Podoviridae | Unassigned | 31_0 | Clustered | VC_31_0 |
| Pseudomonas~phage~YMC11/07/P54_PAE_BP | Caudovirales | Siphoviridae | Unassigned | 41_0 | Clustered | VC_41_0 |
| Pseudomonas~phage~Zigelbrucke | Caudovirales | Myoviridae | Pakpunavirus | 46_0 | Clustered | VC_46_0 |
| Pseudomonas~phage~nickie | Caudovirales | Siphoviridae | Nickievirus | 250_0 | Clustered | VC_250_0 |
| Pseudomonas~phage~phi12 | Unassigned | Cystoviridae | Cystovirus | 347_0 | Clustered | VC_347_0 |
| Pseudomonas~phage~phi13 | Unassigned | Cystoviridae | Cystovirus | 346_0 | Clustered | VC_346_0 |
| Pseudomonas~phage~phi15 | Caudovirales | Podoviridae | Teseptimavirus | 210_0 | Clustered | VC_210_0 |
| Pseudomonas~phage~phi2 | Caudovirales | Siphoviridae | Unassigned | 249_0 | Clustered | VC_249_0 |
| Pseudomonas~phage~phi2954 | Unassigned | Cystoviridae | Cystovirus | 347_0 | Clustered | VC_347_0 |
| Pseudomonas~phage~phi297 | Caudovirales | Siphoviridae | Unassigned | 41_0 | Clustered | VC_41_0 |
| Pseudomonas~phage~phi3 | Caudovirales | Myoviridae | Hpunavirus | 70_0 | Clustered | VC_70_0 |
| Pseudomonas~phage~phiIBB-PAA2 | Caudovirales | Podoviridae | Luz24virus | 213_0 | Clustered | VC_213_0 |
| Pseudomonas~phage~phiIBB-PF7A | Caudovirales | Podoviridae | Teseptimavirus | 210_0 | Clustered | VC_210_0 |
| Pseudomonas~phage~phiKMV | Caudovirales | Podoviridae | Phikmvvirus | 43_0 | Clustered | VC_43_0 |
| Pseudomonas~phage~phiKTN6 | Caudovirales | Myoviridae | Pbunavirus | 186_0 | Clustered | VC_186_0 |
| Pseudomonas~phage~phiMK | Caudovirales | Myoviridae | Pakpunavirus | 46_0 | Clustered | VC_46_0 |
| Pseudomonas~phage~phiNN | Unassigned | Cystoviridae | Cystovirus | 348_0 | Clustered | VC_348_0 |
| Pseudomonas~phage~phiPMW | Caudovirales | Myoviridae | Plaisancevirus | 234_0 | Clustered/Singleton | VC_234_0 |
| Pseudomonas~phage~phiPSA1 | Caudovirales | Siphoviridae | Unassigned | 248_0 | Clustered | VC_248_0 |
| Pseudomonas~phage~phiPSA2 | Caudovirales | Podoviridae | Teseptimavirus | 210_0 | Clustered | VC_210_0 |
| Pseudomonas~phage~phiPto-bp6g | Caudovirales | Siphoviridae | Unassigned | 234_1 | Clustered/Singleton | VC_234_1 |
| Pseudomonas~phage~phiR18 | Caudovirales | Podoviridae | Kpp25virus | 344_0 | Clustered | VC_344_0 |
| Pseudomonas~phage~phiYY | Unassigned | Cystoviridae | Cystovirus | 346_0 | Clustered | VC_346_0 |
| Pseudomonas~phage~phikF77 | Caudovirales | Podoviridae | Phikmvvirus | 43_0 | Clustered | VC_43_0 |
| Pseudomonas~phage~tf | Caudovirales | Podoviridae | Krylovvirus | 213_0 | Clustered | VC_213_0 |
| Pseudomonas~phage~vB_Pae-Kakheti25 | Caudovirales | Siphoviridae | Septima3virus | 1_0 | Clustered | VC_1_0 |
| Pseudomonas~phage~vB_Pae-TbilisiM32 | Caudovirales | Podoviridae | Phikmvvirus | 43_0 | Clustered | VC_43_0 |
| Pseudomonas~phage~vB_PaeM_C2-10_Ab02 | Caudovirales | Myoviridae | Pakpunavirus | 46_0 | Clustered | VC_46_0 |
| Pseudomonas~phage~vB_PaeM_C2-10_Ab1 | Caudovirales | Myoviridae | Pakpunavirus | 46_0 | Clustered | VC_46_0 |
| Pseudomonas~phage~vB_PaeM_CEB_DP1 | Caudovirales | Myoviridae | Pbunavirus | 186_0 | Clustered | VC_186_0 |
| Pseudomonas~phage~vB_PaeM_E215 | Caudovirales | Myoviridae | Pbunavirus | 186_0 | Clustered | VC_186_0 |
| Pseudomonas~phage~vB_PaeM_E217 | Caudovirales | Myoviridae | Pbunavirus | 186_0 | Clustered | VC_186_0 |
| Pseudomonas~phage~vB_PaeM_MAG1 | Caudovirales | Myoviridae | Pakpunavirus | 46_0 | Clustered | VC_46_0 |
| Pseudomonas~phage~vB_PaeM_PAO1_Ab27 | Caudovirales | Myoviridae | Pbunavirus | 186_0 | Clustered | VC_186_0 |
| Pseudomonas~phage~vB_PaeP_C2-10_Ab09 | Caudovirales | Podoviridae | Lit1virus | 13_0 | Clustered | VC_13_0 |
| Pseudomonas~phage~vB_PaeP_C2-10_Ab22 | Caudovirales | Podoviridae | Luz24virus | 213_0 | Clustered | VC_213_0 |
| Pseudomonas~phage~vB_PaeP_MAG4 | Caudovirales | Podoviridae | Litunavirus | 13_0 | Clustered | VC_13_0 |
| Pseudomonas~phage~vB_PaeP_PAO1_Ab05 | Caudovirales | Podoviridae | Phikmvvirus | 43_0 | Clustered | VC_43_0 |
| Pseudomonas~phage~vB_PaeP_PPA-ABTNL | Caudovirales | Podoviridae | Phikmvvirus | 43_0 | Clustered | VC_43_0 |
| Pseudomonas~phage~vB_PaeP_Tr60_Ab31 | Unassigned | Unassigned | Unassigned | 161_0 | Clustered | VC_161_0 |
| Pseudomonas~phage~vB_PaeP_p2-10_Or1 | Caudovirales | Podoviridae | Bruynoghevirus | 213_0 | Clustered | VC_213_0 |
| Pseudomonas~phage~vB_PaeS_PAO1_Ab30 | Caudovirales | Siphoviridae | Casadabanvirus | 190_0 | Clustered | VC_190_0 |
| Pseudomonas~phage~vB_PaeS_PM105 | Caudovirales | Siphoviridae | Beetrevirus | 189_0 | Clustered | VC_189_0 |
| Pseudomonas~phage~vB_PaeS_SCH_Ab26 | Caudovirales | Siphoviridae | Septima3virus | 1_0 | Clustered | VC_1_0 |
| Pseudomonas~phage~vB_Pae_PS44 | Caudovirales | Myoviridae | Pbunavirus | 186_0 | Clustered | VC_186_0 |
| Pseudomonas~phage~vB_PsyM_KIL1 | Caudovirales | Myoviridae | Flaumdravirus | 48_0 | Clustered | VC_48_0 |
| Pseudomonas~phage~vB_PsyM_KIL4 | Caudovirales | Myoviridae | Flaumdravirus | 48_0 | Clustered | VC_48_0 |
| Pseudomonas~virus~119X | Caudovirales | Podoviridae | Unassigned | 332_0 | Clustered | VC_332_0 |
| Pseudomonas~virus~D3 | Caudovirales | Siphoviridae | D3virus | 41_2 | Clustered | VC_41_2 |
| Pseudomonas~virus~D3112 | Caudovirales | Siphoviridae | D3112virus | 190_0 | Clustered | VC_190_0 |
| Pseudomonas~virus~DMS3 | Caudovirales | Siphoviridae | D3112virus | 190_0 | Clustered | VC_190_0 |
| Pseudomonas~virus~FHA0480 | Caudovirales | Siphoviridae | D3112virus | 190_0 | Clustered | VC_190_0 |
| Pseudomonas~virus~LPB1 | Caudovirales | Siphoviridae | D3112virus | 190_0 | Clustered | VC_190_0 |
| Pseudomonas~virus~LUZ24 | Caudovirales | Podoviridae | Luz24virus | 213_0 | Clustered | VC_213_0 |
| Pseudomonas~virus~M6 | Caudovirales | Siphoviridae | Yuavirus | 2_0 | Clustered | VC_2_0 |
| Pseudomonas~virus~MP22 | Caudovirales | Siphoviridae | D3112virus | 190_0 | Clustered | VC_190_0 |
| Pseudomonas~virus~MP29 | Caudovirales | Siphoviridae | D3112virus | 190_0 | Clustered | VC_190_0 |
| Pseudomonas~virus~MP38 | Caudovirales | Siphoviridae | D3112virus | 190_0 | Clustered | VC_190_0 |
| Pseudomonas~virus~PA1KOR | Caudovirales | Siphoviridae | D3112virus | 190_0 | Clustered | VC_190_0 |
| Pseudomonas~virus~PMG1 | Caudovirales | Siphoviridae | D3virus | 41_2 | Clustered | VC_41_2 |
| Pseudomonas~virus~PaP3 | Caudovirales | Podoviridae | Luz24virus | 213_0 | Clustered | VC_213_0 |
| Pseudomonas~virus~Yua | Caudovirales | Siphoviridae | Yuavirus | 2_0 | Clustered | VC_2_0 |
| Pseudomonas~virus~phi6 | Unassigned | Cystoviridae | Cystovirus | 348_0 | Clustered | VC_348_0 |
| Pseudomonas~virus~phiCTX | Caudovirales | Myoviridae | P2virus | 69_0 | Clustered | VC_69_0 |
| Pseudomonas~virus~phiKZ | Caudovirales | Myoviridae | Phikzvirus | 230_0 | Clustered | VC_230_0 |
| Puniceispirillum~phage~HMO-2011 | Caudovirales | Podoviridae | Unassigned | 201_0 | Clustered | VC_201_0 |
| Pyrobaculum~spherical~virus | Unassigned | Globuloviridae | Globulovirus | 349_0 | Clustered | VC_349_0 |
| Ralstonia~phage~PE226 | Unassigned | Inoviridae | Unassigned | 351_0 | Clustered | VC_351_0 |
| Ralstonia~phage~RS603 | Unassigned | Inoviridae | Habenivirus | 350_0 | Clustered | VC_350_0 |
| Ralstonia~phage~RSB1 | Caudovirales | Podoviridae | Unassigned | 67_0 | Clustered | VC_67_0 |
| Ralstonia~phage~RSB2 | Caudovirales | Podoviridae | Teseptimavirus | 210_0 | Clustered | VC_210_0 |
| Ralstonia~phage~RSJ2 | Caudovirales | Podoviridae | Unassigned | 67_0 | Clustered | VC_67_0 |
| Ralstonia~phage~RSJ5 | Caudovirales | Podoviridae | Unassigned | 67_0 | Clustered | VC_67_0 |
| Ralstonia~phage~RSK1 | Caudovirales | Podoviridae | Unassigned | 164_0 | Clustered | VC_164_0 |
| Ralstonia~phage~RSM1 | Unassigned | Inoviridae | Habenivirus | 350_0 | Clustered | VC_350_0 |
| Ralstonia~phage~RSM3 | Unassigned | Inoviridae | Habenivirus | 350_0 | Clustered | VC_350_0 |
| Ralstonia~phage~RSS0 | Unassigned | Inoviridae | Unassigned | 353_0 | Clustered | VC_353_0 |
| Ralstonia~phage~RSS1 | Unassigned | Inoviridae | Unassigned | 353_0 | Clustered | VC_353_0 |
| Ralstonia~phage~RSS20 | Unassigned | Inoviridae | Unassigned | 353_0 | Clustered | VC_353_0 |
| Ralstonia~phage~RSY1 | Caudovirales | Myoviridae | Peduovirus | 69_0 | Clustered | VC_69_0 |
| Ralstonia~phage~p12J | Unassigned | Inoviridae | Unassigned | 351_0 | Clustered | VC_351_0 |
| Ralstonia~virus~RSA1 | Caudovirales | Myoviridae | P2virus | 69_0 | Clustered | VC_69_0 |
| Rhizobium~phage~16-3 | Caudovirales | Siphoviridae | Unassigned | 177_0 | Clustered | VC_177_0 |
| Rhizobium~phage~RHEph04 | Caudovirales | Myoviridae | Rheph4virus | 42_0 | Clustered | VC_42_0 |
| Rhizobium~phage~RHEph06 | Caudovirales | Myoviridae | Kleczkowskavirus | 42_0 | Clustered | VC_42_0 |
| Rhizobium~phage~RHEph10 | Caudovirales | Siphoviridae | Nickievirus | 250_1 | Clustered/Singleton | VC_250_1 |
| Rhizobium~phage~RR1-A | Caudovirales | Myoviridae | Unassigned | 104_0 | Clustered | VC_104_0 |
| Rhizobium~phage~vB_RglS_P106B | Caudovirales | Siphoviridae | Rigallicvirus | 71_0 | Clustered | VC_71_0 |
| Rhizobium~phage~vB_RleM_P10VF | Caudovirales | Myoviridae | Unassigned | 27_0 | Clustered | VC_27_0 |
| Rhizobium~phage~vB_RleM_PPF1 | Caudovirales | Myoviridae | Unassigned | 104_0 | Clustered | VC_104_0 |
| Rhizobium~phage~vB_RleS_L338C | Caudovirales | Siphoviridae | Unassigned | 343_0 | Clustered | VC_343_0 |
| Rhodobacter~phage~RC1 | Caudovirales | Siphoviridae | Unassigned | 191_0 | Clustered | VC_191_0 |
| Rhodobacter~phage~RcCronus | Caudovirales | Siphoviridae | Cronusvirus | 241_0 | Clustered | VC_241_0 |
| Rhodobacter~phage~RcRhea | Caudovirales | Siphoviridae | Cronusvirus | 241_0 | Clustered | VC_241_0 |
| Rhodobacter~phage~RcSpartan | Caudovirales | Siphoviridae | Titanvirus | 1_0 | Clustered | VC_1_0 |
| Rhodobacter~phage~RcTitan | Caudovirales | Siphoviridae | Titanvirus | 1_0 | Clustered | VC_1_0 |
| Rhodococcus~phage~CosmicSans | Caudovirales | Siphoviridae | Rerduovirus | 286_0 | Clustered | VC_286_0 |
| Rhodococcus~phage~REQ3 | Caudovirales | Siphoviridae | Unassigned | 266_0 | Clustered | VC_266_0 |
| Rhodococcus~phage~RER2 | Caudovirales | Siphoviridae | Rer2virus | 286_0 | Clustered | VC_286_0 |
| Rhodococcus~phage~RRH1 | Caudovirales | Siphoviridae | Unassigned | 97_0 | Clustered | VC_97_0 |
| Rhodococcus~phage~Trina | Caudovirales | Siphoviridae | Trinavirus | 287_0 | Clustered/Singleton | VC_287_0 |
| Rhodococcus~phage~Weasels2 | Caudovirales | Siphoviridae | Weaselvirus | 287_1 | Clustered/Singleton | VC_287_1 |
| Rhodococcus~virus~RGL3 | Caudovirales | Siphoviridae | Unassigned | 286_0 | Clustered | VC_286_0 |
| Rhodoferax~phage~P26218 | Caudovirales | Podoviridae | Unassigned | 165_0 | Clustered/Singleton | VC_165_0 |
| Rhodovulum~phage~RS1 | Caudovirales | Siphoviridae | Unassigned | 191_0 | Clustered | VC_191_0 |
| Roseobacter~phage~RDJL~Phi~1 | Caudovirales | Siphoviridae | Rdjlvirus | 5_0 | Clustered | VC_5_0 |
| Roseobacter~phage~RDJL~Phi~2 | Caudovirales | Siphoviridae | Rdjlvirus | 5_0 | Clustered | VC_5_0 |
| Roseobacter~virus~SIO1 | Caudovirales | Podoviridae | Unassigned | 200_0 | Clustered | VC_200_0 |
| SPRUCE_viral_seq_1218 | Unassigned | Unassigned | Unassigned | 318_0 | Clustered | VC_318_0 |
| SPRUCE_viral_seq_2687 | Unassigned | Unassigned | Unassigned | 318_0 | Clustered | VC_318_0 |
| Salinivibrio~phage~CW02 | Caudovirales | Podoviridae | Unassigned | 199_0 | Clustered | VC_199_0 |
| Salmonella~phage~100268_sal2 | Caudovirales | Siphoviridae | T5virus | 261_1 | Clustered | VC_261_1 |
| Salmonella~phage~103203_sal5 | Caudovirales | Podoviridae | Lederbergvirus | 163_0 | Clustered | VC_163_0 |
| Salmonella~phage~118970_sal1 | Caudovirales | Siphoviridae | Chivirus | 15_0 | Clustered | VC_15_0 |
| Salmonella~phage~118970_sal2 | Caudovirales | Siphoviridae | T5virus | 261_1 | Clustered | VC_261_1 |
| Salmonella~phage~118970_sal3 | Caudovirales | Myoviridae | Unassigned | 106_0 | Clustered | VC_106_0 |
| Salmonella~phage~118970_sal4 | Caudovirales | Podoviridae | Lederbergvirus | 163_0 | Clustered | VC_163_0 |
| Salmonella~phage~36 | Caudovirales | Siphoviridae | Tlsvirus | 64_0 | Clustered | VC_64_0 |
| Salmonella~phage~37 | Caudovirales | Siphoviridae | Chivirus | 15_0 | Clustered | VC_15_0 |
| Salmonella~phage~38 | Caudovirales | Ackermannviridae | Cba120virus | 26_1 | Clustered | VC_26_1 |
| Salmonella~phage~64795_sal3 | Caudovirales | Siphoviridae | Roufvirus | 61_0 | Clustered | VC_61_0 |
| Salmonella~phage~7-11 | Caudovirales | Podoviridae | Unassigned | 235_0 | Clustered | VC_235_0 |
| Salmonella~phage~9NA | Caudovirales | Podoviridae | Nonanavirus | 245_0 | Clustered | VC_245_0 |
| Salmonella~phage~BP12A | Caudovirales | Podoviridae | Teseptimavirus | 210_0 | Clustered | VC_210_0 |
| Salmonella~phage~BP12B | Caudovirales | Podoviridae | Zindervirus | 254_0 | Clustered | VC_254_0 |
| Salmonella~phage~BP12C | Caudovirales | Siphoviridae | Chivirus | 15_0 | Clustered | VC_15_0 |
| Salmonella~phage~BPS11Q3 | Caudovirales | Siphoviridae | Jerseyvirus | 50_0 | Clustered | VC_50_0 |
| Salmonella~phage~BPS15Q2 | Caudovirales | Myoviridae | Felixounavirus | 16_0 | Clustered | VC_16_0 |
| Salmonella~phage~BPS17L1 | Caudovirales | Myoviridae | Felixounavirus | 16_0 | Clustered | VC_16_0 |
| Salmonella~phage~BPS17W1 | Caudovirales | Myoviridae | Felixounavirus | 16_0 | Clustered | VC_16_0 |
| Salmonella~phage~Det7 | Caudovirales | Ackermannviridae | Cba120virus | 26_1 | Clustered | VC_26_1 |
| Salmonella~phage~Ent1 | Caudovirales | Siphoviridae | Jerseyvirus | 50_0 | Clustered | VC_50_0 |
| Salmonella~phage~FSL~SP-004 | Caudovirales | Myoviridae | Peduovirus | 69_0 | Clustered | VC_69_0 |
| Salmonella~phage~FSL~SP-031 | Caudovirales | Siphoviridae | Sp31virus | 50_0 | Clustered | VC_50_0 |
| Salmonella~phage~FSL~SP-101 | Caudovirales | Siphoviridae | Jerseyvirus | 50_0 | Clustered | VC_50_0 |
| Salmonella~phage~FelixO1 | Caudovirales | Myoviridae | Felixo1virus | 16_0 | Clustered | VC_16_0 |
| Salmonella~phage~GG32 | Caudovirales | Ackermannviridae | Cba120virus | 26_1 | Clustered | VC_26_1 |
| Salmonella~phage~IME207 | Caudovirales | Siphoviridae | Roufvirus | 61_0 | Clustered | VC_61_0 |
| Salmonella~phage~Jersey | Caudovirales | Siphoviridae | Jerseyvirus | 50_0 | Clustered | VC_50_0 |
| Salmonella~phage~LSPA1 | Caudovirales | Siphoviridae | Jerseyvirus | 50_0 | Clustered | VC_50_0 |
| Salmonella~phage~MA12 | Caudovirales | Siphoviridae | Jerseyvirus | 50_0 | Clustered | VC_50_0 |
| Salmonella~phage~Marshall | Caudovirales | Ackermannviridae | Vi1virus | 26_1 | Clustered | VC_26_1 |
| Salmonella~phage~Maynard | Caudovirales | Ackermannviridae | Vi1virus | 26_1 | Clustered | VC_26_1 |
| Salmonella~phage~Melville | Caudovirales | Myoviridae | Gelderlandvirus | 34_6 | Clustered | VC_34_6 |
| Salmonella~phage~Mushroom | Caudovirales | Myoviridae | Felixo1virus | 16_0 | Clustered | VC_16_0 |
| Salmonella~phage~NR01 | Caudovirales | Siphoviridae | Tequintavirus | 261_1 | Clustered | VC_261_1 |
| Salmonella~phage~PVP-SE1 | Caudovirales | Myoviridae | Se1virus | 226_1 | Clustered | VC_226_1 |
| Salmonella~phage~RE-2010 | Caudovirales | Myoviridae | Peduovirus | 69_0 | Clustered | VC_69_0 |
| Salmonella~phage~SE1~(in:Nonagvirus) | Caudovirales | Siphoviridae | Nonagvirus | 14_0 | Clustered | VC_14_0 |
| Salmonella~phage~SE1~(in:P22virus) | Caudovirales | Podoviridae | Lederbergvirus | 163_0 | Clustered | VC_163_0 |
| Salmonella~phage~SE2 | Caudovirales | Siphoviridae | Jerseyvirus | 50_0 | Clustered | VC_50_0 |
| Salmonella~phage~SEN1 | Caudovirales | Myoviridae | Peduovirus | 69_0 | Clustered | VC_69_0 |
| Salmonella~phage~SEN22 | Caudovirales | Podoviridae | Lederbergvirus | 163_0 | Clustered | VC_163_0 |
| Salmonella~phage~SEN4 | Caudovirales | Myoviridae | Peduovirus | 69_0 | Clustered | VC_69_0 |
| Salmonella~phage~SEN5 | Caudovirales | Myoviridae | Peduovirus | 69_0 | Clustered | VC_69_0 |
| Salmonella~phage~SETP13 | Caudovirales | Siphoviridae | Jerseyvirus | 50_0 | Clustered | VC_50_0 |
| Salmonella~phage~SETP3 | Caudovirales | Siphoviridae | Jerseyvirus | 50_0 | Clustered | VC_50_0 |
| Salmonella~phage~SETP7 | Caudovirales | Siphoviridae | Jerseyvirus | 50_0 | Clustered | VC_50_0 |
| Salmonella~phage~SFP10 | Caudovirales | Ackermannviridae | Cba120virus | 26_1 | Clustered | VC_26_1 |
| Salmonella~phage~SJ46 | Caudovirales | Myoviridae | Punavirus | 260_0 | Clustered | VC_260_0 |
| Salmonella~phage~SKML-39 | Caudovirales | Ackermannviridae | Ag3virus | 26_1 | Clustered | VC_26_1 |
| Salmonella~phage~SP069 | Caudovirales | Podoviridae | Nonanavirus | 245_0 | Clustered | VC_245_0 |
| Salmonella~phage~SPN1S | Caudovirales | Podoviridae | Epsilon15virus | 168_0 | Clustered | VC_168_0 |
| Salmonella~phage~SPN3US | Caudovirales | Myoviridae | Spn3virus | 229_1 | Clustered | VC_229_1 |
| Salmonella~phage~SPN9CC | Caudovirales | Podoviridae | Lederbergvirus | 163_0 | Clustered | VC_163_0 |
| Salmonella~phage~SS3e | Caudovirales | Siphoviridae | Jerseyvirus | 50_0 | Clustered | VC_50_0 |
| Salmonella~phage~SSE121 | Caudovirales | Myoviridae | Se1virus | 226_1 | Clustered | VC_226_1 |
| Salmonella~phage~SSU5 | Caudovirales | Siphoviridae | Nickievirus | 250_0 | Clustered | VC_250_0 |
| Salmonella~phage~ST160 | Caudovirales | Podoviridae | Lederbergvirus | 163_0 | Clustered | VC_163_0 |
| Salmonella~phage~ST64B | Caudovirales | Myoviridae | Unassigned | 106_0 | Clustered | VC_106_0 |
| Salmonella~phage~STML-13-1 | Caudovirales | Ackermannviridae | Vi1virus | 26_1 | Clustered | VC_26_1 |
| Salmonella~phage~STML-198 | Caudovirales | Myoviridae | S16virus | 34_6 | Clustered | VC_34_6 |
| Salmonella~phage~STP4-a | Caudovirales | Myoviridae | Gelderlandvirus | 34_6 | Clustered | VC_34_6 |
| Salmonella~phage~Sh19 | Caudovirales | Ackermannviridae | Cba120virus | 26_1 | Clustered | VC_26_1 |
| Salmonella~phage~Shivani | Caudovirales | Siphoviridae | T5virus | 261_1 | Clustered | VC_261_1 |
| Salmonella~phage~Si3 | Caudovirales | Myoviridae | Felixounavirus | 16_0 | Clustered | VC_16_0 |
| Salmonella~phage~Vi~II-E1 | Caudovirales | Siphoviridae | Roufvirus | 61_0 | Clustered | VC_61_0 |
| Salmonella~phage~Vi06 | Caudovirales | Podoviridae | Teseptimavirus | 210_0 | Clustered | VC_210_0 |
| Salmonella~phage~epsilon15 | Caudovirales | Podoviridae | Epsilon15virus | 168_0 | Clustered | VC_168_0 |
| Salmonella~phage~epsilon34 | Caudovirales | Podoviridae | Lederbergvirus | 163_0 | Clustered | VC_163_0 |
| Salmonella~phage~f18SE | Caudovirales | Siphoviridae | Jerseyvirus | 50_0 | Clustered | VC_50_0 |
| Salmonella~phage~g341c | Caudovirales | Podoviridae | Lederbergvirus | 163_0 | Clustered | VC_163_0 |
| Salmonella~phage~phSE-2 | Caudovirales | Siphoviridae | Tlsvirus | 64_0 | Clustered | VC_64_0 |
| Salmonella~phage~phiSG-JL2 | Caudovirales | Podoviridae | Teseptimavirus | 210_0 | Clustered | VC_210_0 |
| Salmonella~phage~vB_SPuM_SP116 | Caudovirales | Myoviridae | Felixounavirus | 16_0 | Clustered | VC_16_0 |
| Salmonella~phage~vB_SalM_PM10 | Caudovirales | Ackermannviridae | Cba120virus | 26_1 | Clustered | VC_26_1 |
| Salmonella~phage~vB_SalM_SJ2 | Caudovirales | Ackermannviridae | Vi1virus | 26_1 | Clustered | VC_26_1 |
| Salmonella~phage~vB_SalM_SJ3 | Caudovirales | Ackermannviridae | Cba120virus | 26_1 | Clustered | VC_26_1 |
| Salmonella~phage~vB_SemP_Emek | Caudovirales | Podoviridae | Lederbergvirus | 163_0 | Clustered | VC_163_0 |
| Salmonella~phage~vB_SenMS16 | Caudovirales | Myoviridae | S16virus | 34_6 | Clustered | VC_34_6 |
| Salmonella~phage~vB_SenS-Ent2 | Caudovirales | Siphoviridae | Jerseyvirus | 50_0 | Clustered | VC_50_0 |
| Salmonella~phage~vB_SenS-Ent3 | Caudovirales | Siphoviridae | Jerseyvirus | 50_0 | Clustered | VC_50_0 |
| Salmonella~phage~vB_SenS_AG11 | Caudovirales | Siphoviridae | Jerseyvirus | 50_0 | Clustered | VC_50_0 |
| Salmonella~phage~vB_SnwM_CGG4-1 | Caudovirales | Myoviridae | Gelderlandvirus | 34_6 | Clustered | VC_34_6 |
| Salmonella~phage~wksl3 | Caudovirales | Siphoviridae | Jerseyvirus | 50_0 | Clustered | VC_50_0 |
| Salmonella~virus~Chi | Caudovirales | Siphoviridae | Chivirus | 15_0 | Clustered | VC_15_0 |
| Salmonella~virus~FSLSP030 | Caudovirales | Siphoviridae | Chivirus | 15_0 | Clustered | VC_15_0 |
| Salmonella~virus~FSLSP088 | Caudovirales | Siphoviridae | Chivirus | 15_0 | Clustered | VC_15_0 |
| Salmonella~virus~Fels2 | Caudovirales | Myoviridae | P2virus | 69_0 | Clustered | VC_69_0 |
| Salmonella~virus~HK620 | Caudovirales | Podoviridae | P22virus | 163_0 | Clustered | VC_163_0 |
| Salmonella~virus~P22 | Caudovirales | Podoviridae | P22virus | 163_0 | Clustered | VC_163_0 |
| Salmonella~virus~PsP3 | Caudovirales | Myoviridae | P2virus | 69_0 | Clustered | VC_69_0 |
| Salmonella~virus~SP126 | Caudovirales | Siphoviridae | Tlsvirus | 64_0 | Clustered | VC_64_0 |
| Salmonella~virus~SP6 | Caudovirales | Podoviridae | Sp6virus | 254_0 | Clustered | VC_254_0 |
| Salmonella~virus~SPC35 | Caudovirales | Siphoviridae | T5virus | 261_1 | Clustered | VC_261_1 |
| Salmonella~virus~SPN19 | Caudovirales | Siphoviridae | Chivirus | 15_0 | Clustered | VC_15_0 |
| Salmonella~virus~ST64T | Caudovirales | Podoviridae | P22virus | 163_0 | Clustered | VC_163_0 |
| Salmonella~virus~Stitch | Caudovirales | Siphoviridae | T5virus | 261_1 | Clustered | VC_261_1 |
| Salmonella~virus~ViI | Caudovirales | Ackermannviridae | Vi1virus | 26_1 | Clustered | VC_26_1 |
| Salmonella~virus~iEPS5 | Caudovirales | Siphoviridae | Chivirus | 15_0 | Clustered | VC_15_0 |
| Serratia~phage~BF | Caudovirales | Myoviridae | Eneladusvirus | 72_2 | Clustered/Singleton | VC_72_2 |
| Serratia~phage~CHI14 | Caudovirales | Myoviridae | Winklervirus | 34_4 | Clustered/Singleton | VC_34_4 |
| Serratia~phage~phiMAM1 | Caudovirales | Ackermannviridae | Unassigned | 26_0 | Clustered | VC_26_0 |
| Serratia~phage~vB_Sru_IME250 | Caudovirales | Ackermannviridae | Unassigned | 26_1 | Clustered | VC_26_1 |
| Shewanella~phage~Spp001 | Caudovirales | Myoviridae | Unassigned | 59_0 | Clustered | VC_59_0 |
| Shewanella~sp.~phage~1/4 | Caudovirales | Myoviridae | Unassigned | 215_0 | Clustered | VC_215_0 |
| Shewanella~sp.~phage~1/40 | Caudovirales | Myoviridae | Unassigned | 215_0 | Clustered | VC_215_0 |
| Shewanella~sp.~phage~1/44 | Caudovirales | Siphoviridae | Unassigned | 244_0 | Clustered | VC_244_0 |
| Shigella~phage~75/02~Stx | Caudovirales | Podoviridae | Pocjvirus | 192_1 | Clustered | VC_192_1 |
| Shigella~phage~Ag3 | Caudovirales | Ackermannviridae | Ag3virus | 26_1 | Clustered | VC_26_1 |
| Shigella~phage~EP23 | Caudovirales | Siphoviridae | Hk578virus | 233_0 | Clustered | VC_233_0 |
| Shigella~phage~POCJ13 | Caudovirales | Podoviridae | Pocjvirus | 192_1 | Clustered | VC_192_1 |
| Shigella~phage~SHBML-50-1 | Caudovirales | Myoviridae | Tequatrovirus | 34_5 | Clustered | VC_34_5 |
| Shigella~phage~SHFML-11 | Caudovirales | Myoviridae | Tequatrovirus | 34_5 | Clustered | VC_34_5 |
| Shigella~phage~SHFML-26 | Caudovirales | Myoviridae | Tequatrovirus | 34_5 | Clustered | VC_34_5 |
| Shigella~phage~SHSML-45 | Caudovirales | Siphoviridae | Tequintavirus | 261_1 | Clustered | VC_261_1 |
| Shigella~phage~SHSML-52-1 | Caudovirales | Myoviridae | Mosigvirus | 34_0 | Clustered | VC_34_0 |
| Shigella~phage~SP18 | Caudovirales | Myoviridae | Sp18virus | 34_12 | Clustered | VC_34_12 |
| Shigella~phage~Sf13 | Caudovirales | Myoviridae | Mooglevirus | 16_0 | Clustered | VC_16_0 |
| Shigella~phage~Sf14 | Caudovirales | Myoviridae | Mooglevirus | 16_0 | Clustered | VC_16_0 |
| Shigella~phage~Sf17 | Caudovirales | Myoviridae | Mooglevirus | 16_0 | Clustered | VC_16_0 |
| Shigella~phage~Sf21 | Caudovirales | Myoviridae | Tequatrovirus | 34_5 | Clustered | VC_34_5 |
| Shigella~phage~Sf22 | Caudovirales | Myoviridae | Tequatrovirus | 34_5 | Clustered | VC_34_5 |
| Shigella~phage~Sf24 | Caudovirales | Myoviridae | Tequatrovirus | 34_5 | Clustered | VC_34_5 |
| Shigella~phage~SfII | Caudovirales | Myoviridae | Unassigned | 106_0 | Clustered | VC_106_0 |
| Shigella~phage~SfIV | Caudovirales | Myoviridae | Unassigned | 106_0 | Clustered | VC_106_0 |
| Shigella~phage~SfMu | Caudovirales | Myoviridae | Muvirus | 188_0 | Clustered | VC_188_0 |
| Shigella~phage~Shf125875 | Caudovirales | Myoviridae | Rb69virus | 34_0 | Clustered | VC_34_0 |
| Shigella~phage~Shfl2 | Caudovirales | Myoviridae | T4virus | 34_5 | Clustered | VC_34_5 |
| Shigella~phage~Ss-VASD | Caudovirales | Podoviridae | Tl2011virus | 192_1 | Clustered | VC_192_1 |
| Shigella~phage~pSb-1 | Caudovirales | Podoviridae | G7cvirus | 7_1 | Clustered | VC_7_1 |
| Shigella~phage~pSf-1 | Caudovirales | Siphoviridae | Hanrivervirus | 64_0 | Clustered | VC_64_0 |
| Shigella~phage~pSf-2 | Caudovirales | Siphoviridae | T1virus | 64_0 | Clustered | VC_64_0 |
| Shigella~phage~pSs-1 | Caudovirales | Myoviridae | T4virus | 34_5 | Clustered | VC_34_5 |
| Shigella~phage~vB_SsoS-ISF002 | Caudovirales | Siphoviridae | Tunavirus | 64_0 | Clustered | VC_64_0 |
| Shigella~virus~Sf6 | Caudovirales | Podoviridae | P22virus | 163_0 | Clustered | VC_163_0 |
| Shigella~virus~Shfl1 | Caudovirales | Siphoviridae | T1virus | 64_0 | Clustered | VC_64_0 |
| Sinorhizobium~phage~phiLM21 | Caudovirales | Siphoviridae | Unassigned | 248_0 | Clustered | VC_248_0 |
| Sinorhizobium~phage~phiM12 | Caudovirales | Myoviridae | M12virus | 24_0 | Clustered | VC_24_0 |
| Sinorhizobium~phage~phiM7 | Caudovirales | Myoviridae | M12virus | 24_0 | Clustered | VC_24_0 |
| Sinorhizobium~phage~phiM9 | Caudovirales | Myoviridae | Unassigned | 27_0 | Clustered | VC_27_0 |
| Sinorhizobium~phage~phiN3 | Caudovirales | Myoviridae | M12virus | 24_0 | Clustered | VC_24_0 |
| Sodalis~phage~SO1 | Caudovirales | Siphoviridae | Hk578virus | 233_0 | Clustered | VC_233_0 |
| Spiroplasma~phage~1-C74 | Unassigned | Inoviridae | Vespertiliovirus | 356_0 | Clustered | VC_356_0 |
| Spiroplasma~phage~1-R8A2B | Unassigned | Inoviridae | Vespertiliovirus | 356_0 | Clustered | VC_356_0 |
| Spiroplasma~virus~SkV1CR23x | Unassigned | Inoviridae | Vespertiliovirus | 356_0 | Clustered | VC_356_0 |
| Staphylococcus~phage~23MRA | Caudovirales | Siphoviridae | Biseptimavirus | 144_0 | Clustered | VC_144_0 |
| Staphylococcus~phage~3MRA | Caudovirales | Siphoviridae | Phietavirus | 147_0 | Clustered | VC_147_0 |
| Staphylococcus~phage~66 | Caudovirales | Podoviridae | Rosenblumvirus | 258_0 | Clustered | VC_258_0 |
| Staphylococcus~phage~6ec | Caudovirales | Siphoviridae | Sextaecvirus | 160_0 | Clustered | VC_160_0 |
| Staphylococcus~phage~812 | Caudovirales | Herelleviridae | Kayvirus | 128_0 | Clustered | VC_128_0 |
| Staphylococcus~phage~B166 | Caudovirales | Siphoviridae | Phietavirus | 147_0 | Clustered | VC_147_0 |
| Staphylococcus~phage~B236 | Caudovirales | Siphoviridae | Phietavirus | 147_0 | Clustered | VC_147_0 |
| Staphylococcus~phage~BP39 | Caudovirales | Podoviridae | Rosenblumvirus | 258_0 | Clustered | VC_258_0 |
| Staphylococcus~phage~DW2 | Caudovirales | Siphoviridae | Phietavirus | 147_0 | Clustered | VC_147_0 |
| Staphylococcus~phage~GH15 | Caudovirales | Myoviridae | Kayvirus | 128_0 | Clustered | VC_128_0 |
| Staphylococcus~phage~GRCS | Caudovirales | Podoviridae | Rosenblumvirus | 258_0 | Clustered | VC_258_0 |
| Staphylococcus~phage~JD007 | Caudovirales | Myoviridae | Kayvirus | 128_0 | Clustered | VC_128_0 |
| Staphylococcus~phage~JS01 | Caudovirales | Siphoviridae | Biseptimavirus | 144_0 | Clustered | VC_144_0 |
| Staphylococcus~phage~MCE-2014 | Caudovirales | Myoviridae | Kayvirus | 128_0 | Clustered | VC_128_0 |
| Staphylococcus~phage~P108 | Caudovirales | Myoviridae | Kayvirus | 128_0 | Clustered | VC_128_0 |
| Staphylococcus~phage~P68 | Caudovirales | Podoviridae | Rosenblumvirus | 258_0 | Clustered | VC_258_0 |
| Staphylococcus~phage~P954 | Caudovirales | Siphoviridae | Biseptimavirus | 144_0 | Clustered | VC_144_0 |
| Staphylococcus~phage~PVL | Caudovirales | Siphoviridae | Biseptimavirus | 144_0 | Clustered | VC_144_0 |
| Staphylococcus~phage~ROSA | Caudovirales | Siphoviridae | Phietavirus | 147_0 | Clustered | VC_147_0 |
| Staphylococcus~phage~S24-1 | Caudovirales | Podoviridae | Rosenblumvirus | 258_0 | Clustered | VC_258_0 |
| Staphylococcus~phage~S25-3 | Caudovirales | Myoviridae | Kayvirus | 128_0 | Clustered | VC_128_0 |
| Staphylococcus~phage~S25-4 | Caudovirales | Myoviridae | Kayvirus | 128_0 | Clustered | VC_128_0 |
| Staphylococcus~phage~SA1 | Caudovirales | Myoviridae | Unassigned | 16_0 | Clustered | VC_16_0 |
| Staphylococcus~phage~SA12 | Caudovirales | Siphoviridae | Phietavirus | 147_0 | Clustered | VC_147_0 |
| Staphylococcus~phage~SA13 | Caudovirales | Siphoviridae | Phietavirus | 147_0 | Clustered | VC_147_0 |
| Staphylococcus~phage~SA97 | Caudovirales | Siphoviridae | Phietavirus | 147_0 | Clustered | VC_147_0 |
| Staphylococcus~phage~SAP-2 | Caudovirales | Podoviridae | Rosenblumvirus | 258_0 | Clustered | VC_258_0 |
| Staphylococcus~phage~SLPW | Caudovirales | Podoviridae | Rosenblumvirus | 258_0 | Clustered | VC_258_0 |
| Staphylococcus~phage~SMSAP5 | Caudovirales | Siphoviridae | Triavirus | 145_0 | Clustered | VC_145_0 |
| Staphylococcus~phage~SPbeta-like | Caudovirales | Siphoviridae | Spbetavirus | 113_1 | Clustered/Singleton | VC_113_1 |
| Staphylococcus~phage~Sb1 | Caudovirales | Herelleviridae | Kayvirus | 128_0 | Clustered | VC_128_0 |
| Staphylococcus~phage~Stau2 | Caudovirales | Myoviridae | Silviavirus | 128_2 | Clustered | VC_128_2 |
| Staphylococcus~phage~StauST398-1 | Caudovirales | Siphoviridae | Phietavirus | 147_0 | Clustered | VC_147_0 |
| Staphylococcus~phage~StauST398-3 | Caudovirales | Siphoviridae | Phietavirus | 147_0 | Clustered | VC_147_0 |
| Staphylococcus~phage~StauST398-4 | Caudovirales | Siphoviridae | Biseptimavirus | 144_0 | Clustered | VC_144_0 |
| Staphylococcus~phage~StauST398-5 | Caudovirales | Siphoviridae | Phietavirus | 147_0 | Clustered | VC_147_0 |
| Staphylococcus~phage~Team1 | Caudovirales | Myoviridae | Kayvirus | 128_0 | Clustered | VC_128_0 |
| Staphylococcus~phage~phi5967PVL | Caudovirales | Siphoviridae | Biseptimavirus | 144_0 | Clustered | VC_144_0 |
| Staphylococcus~phage~phi7401PVL | Caudovirales | Siphoviridae | Triavirus | 145_0 | Clustered | VC_145_0 |
| Staphylococcus~phage~phiBU01 | Caudovirales | Siphoviridae | Biseptimavirus | 144_0 | Clustered | VC_144_0 |
| Staphylococcus~phage~phiIBB-SEP1 | Caudovirales | Myoviridae | Sep1virus | 128_1 | Clustered | VC_128_1 |
| Staphylococcus~phage~phiIPLA-C1C | Caudovirales | Myoviridae | Sep1virus | 128_1 | Clustered | VC_128_1 |
| Staphylococcus~phage~phiIPLA-RODI | Caudovirales | Myoviridae | Kayvirus | 128_0 | Clustered | VC_128_0 |
| Staphylococcus~phage~phiJB | Caudovirales | Siphoviridae | Phietavirus | 147_0 | Clustered | VC_147_0 |
| Staphylococcus~phage~phiN315 | Caudovirales | Siphoviridae | Unassigned | 144_0 | Clustered | VC_144_0 |
| Staphylococcus~phage~phiNM3 | Caudovirales | Siphoviridae | Biseptimavirus | 144_0 | Clustered | VC_144_0 |
| Staphylococcus~phage~phiPVL-CN125 | Caudovirales | Siphoviridae | Biseptimavirus | 144_0 | Clustered | VC_144_0 |
| Staphylococcus~phage~phiSA12 | Caudovirales | Myoviridae | Kayvirus | 128_0 | Clustered | VC_128_0 |
| Staphylococcus~phage~phiSa119 | Caudovirales | Siphoviridae | Biseptimavirus | 144_0 | Clustered | VC_144_0 |
| Staphylococcus~phage~tp310-1 | Caudovirales | Siphoviridae | Biseptimavirus | 144_0 | Clustered | VC_144_0 |
| Staphylococcus~phage~tp310-3 | Caudovirales | Siphoviridae | Biseptimavirus | 144_0 | Clustered | VC_144_0 |
| Staphylococcus~phage~vB_SauM_Remus | Caudovirales | Myoviridae | Silviavirus | 128_2 | Clustered | VC_128_2 |
| Staphylococcus~phage~vB_SauM_Romulus | Caudovirales | Myoviridae | Silviavirus | 128_2 | Clustered | VC_128_2 |
| Staphylococcus~phage~vB_SepS_SEP9 | Caudovirales | Siphoviridae | Sextaecvirus | 160_0 | Clustered | VC_160_0 |
| Staphylococcus~prophage~phiPV83 | Caudovirales | Siphoviridae | Biseptimavirus | 144_0 | Clustered | VC_144_0 |
| Staphylococcus~virus~108PVL | Caudovirales | Siphoviridae | Biseptimavirus | 144_0 | Clustered | VC_144_0 |
| Staphylococcus~virus~11 | Caudovirales | Siphoviridae | Phietavirus | 147_0 | Clustered | VC_147_0 |
| Staphylococcus~virus~13 | Caudovirales | Siphoviridae | Biseptimavirus | 144_0 | Clustered | VC_144_0 |
| Staphylococcus~virus~187 | Caudovirales | Siphoviridae | Phietavirus | 147_0 | Clustered | VC_147_0 |
| Staphylococcus~virus~29 | Caudovirales | Siphoviridae | Phietavirus | 147_0 | Clustered | VC_147_0 |
| Staphylococcus~virus~44AHJD | Caudovirales | Podoviridae | P68virus | 258_0 | Clustered | VC_258_0 |
| Staphylococcus~virus~52a | Caudovirales | Siphoviridae | Phietavirus | 147_0 | Clustered | VC_147_0 |
| Staphylococcus~virus~53 | Caudovirales | Siphoviridae | Phietavirus | 147_0 | Clustered | VC_147_0 |
| Staphylococcus~virus~55 | Caudovirales | Siphoviridae | Phietavirus | 147_0 | Clustered | VC_147_0 |
| Staphylococcus~virus~69 | Caudovirales | Siphoviridae | Phietavirus | 147_0 | Clustered | VC_147_0 |
| Staphylococcus~virus~71 | Caudovirales | Siphoviridae | Phietavirus | 147_0 | Clustered | VC_147_0 |
| Staphylococcus~virus~80 | Caudovirales | Siphoviridae | Phietavirus | 147_0 | Clustered | VC_147_0 |
| Staphylococcus~virus~80alpha | Caudovirales | Siphoviridae | Phietavirus | 147_0 | Clustered | VC_147_0 |
| Staphylococcus~virus~85 | Caudovirales | Siphoviridae | Phietavirus | 147_0 | Clustered | VC_147_0 |
| Staphylococcus~virus~88 | Caudovirales | Siphoviridae | Phietavirus | 147_0 | Clustered | VC_147_0 |
| Staphylococcus~virus~92 | Caudovirales | Siphoviridae | Phietavirus | 147_0 | Clustered | VC_147_0 |
| Staphylococcus~virus~96 | Caudovirales | Siphoviridae | Phietavirus | 147_0 | Clustered | VC_147_0 |
| Staphylococcus~virus~G1 | Caudovirales | Myoviridae | Kayvirus | 128_0 | Clustered | VC_128_0 |
| Staphylococcus~virus~IPLA88 | Caudovirales | Siphoviridae | Phietavirus | 147_0 | Clustered | VC_147_0 |
| Staphylococcus~virus~K | Caudovirales | Myoviridae | Kayvirus | 128_0 | Clustered | VC_128_0 |
| Staphylococcus~virus~SA11 | Caudovirales | Myoviridae | Silviavirus | 128_2 | Clustered | VC_128_2 |
| Staphylococcus~virus~SAP26 | Caudovirales | Siphoviridae | Phietavirus | 147_0 | Clustered | VC_147_0 |
| Staphylococcus~virus~Twort | Caudovirales | Myoviridae | Twortvirus | 128_3 | Clustered/Singleton | VC_128_3 |
| Staphylococcus~virus~X2 | Caudovirales | Siphoviridae | Phietavirus | 147_0 | Clustered | VC_147_0 |
| Staphylococcus~virus~phiETA | Caudovirales | Siphoviridae | Phietavirus | 147_0 | Clustered | VC_147_0 |
| Staphylococcus~virus~phiETA2 | Caudovirales | Siphoviridae | Phietavirus | 147_0 | Clustered | VC_147_0 |
| Staphylococcus~virus~phiETA3 | Caudovirales | Siphoviridae | Phietavirus | 147_0 | Clustered | VC_147_0 |
| Staphylococcus~virus~phiMR11 | Caudovirales | Siphoviridae | Phietavirus | 147_0 | Clustered | VC_147_0 |
| Staphylococcus~virus~phiMR25 | Caudovirales | Siphoviridae | Phietavirus | 147_0 | Clustered | VC_147_0 |
| Staphylococcus~virus~phiNM1 | Caudovirales | Siphoviridae | Phietavirus | 147_0 | Clustered | VC_147_0 |
| Staphylococcus~virus~phiNM2 | Caudovirales | Siphoviridae | Phietavirus | 147_0 | Clustered | VC_147_0 |
| Staphylococcus~virus~phiNM4 | Caudovirales | Siphoviridae | Phietavirus | 147_0 | Clustered | VC_147_0 |
| Stenotrophomonas~phage~IME13 | Caudovirales | Myoviridae | Secunda5virus | 35_2 | Clustered/Singleton | VC_35_2 |
| Stenotrophomonas~phage~IME15 | Caudovirales | Podoviridae | Teseptimavirus | 210_0 | Clustered | VC_210_0 |
| Stenotrophomonas~phage~PSH1 | Unassigned | Inoviridae | Unassigned | 352_0 | Clustered | VC_352_0 |
| Stenotrophomonas~phage~S1 | Caudovirales | Siphoviridae | Unassigned | 244_0 | Clustered | VC_244_0 |
| Stenotrophomonas~phage~SMA7 | Unassigned | Inoviridae | Unassigned | 352_0 | Clustered | VC_352_0 |
| Stenotrophomonas~phage~SMA9 | Unassigned | Inoviridae | Unassigned | 352_0 | Clustered | VC_352_0 |
| Stenotrophomonas~phage~Smp131 | Caudovirales | Myoviridae | Peduovirus | 69_0 | Clustered | VC_69_0 |
| Stenotrophomonas~phage~phiSHP2 | Unassigned | Inoviridae | Unassigned | 352_0 | Clustered | VC_352_0 |
| Stenotrophomonas~phage~vB_SmaS-DLP_2 | Caudovirales | Siphoviridae | Septimatrevirus | 1_0 | Clustered | VC_1_0 |
| Stenotrophomonas~phage~vB_SmaS_DLP_5 | Caudovirales | Siphoviridae | Delepquintavirus | 343_0 | Clustered | VC_343_0 |
| Streptococcus~phage~20617 | Caudovirales | Siphoviridae | Brussowvirus | 124_0 | Clustered | VC_124_0 |
| Streptococcus~phage~315.1 | Caudovirales | Siphoviridae | Unassigned | 157_0 | Clustered | VC_157_0 |
| Streptococcus~phage~315.3 | Caudovirales | Siphoviridae | Unassigned | 256_0 | Clustered | VC_256_0 |
| Streptococcus~phage~315.6 | Caudovirales | Siphoviridae | Unassigned | 146_0 | Clustered | VC_146_0 |
| Streptococcus~phage~A25 | Caudovirales | Siphoviridae | Unassigned | 125_0 | Clustered | VC_125_0 |
| Streptococcus~phage~APCM01 | Caudovirales | Siphoviridae | Unassigned | 298_0 | Clustered | VC_298_0 |
| Streptococcus~phage~CP-7 | Caudovirales | Podoviridae | Cp1virus | 53_0 | Clustered | VC_53_0 |
| Streptococcus~phage~Cp-1 | Caudovirales | Podoviridae | Cp1virus | 53_0 | Clustered | VC_53_0 |
| Streptococcus~phage~DCC1738 | Caudovirales | Siphoviridae | Unassigned | 172_0 | Clustered | VC_172_0 |
| Streptococcus~phage~Dp-1 | Caudovirales | Siphoviridae | Unassigned | 220_0 | Clustered | VC_220_0 |
| Streptococcus~phage~IC1 | Caudovirales | Siphoviridae | Unassigned | 172_0 | Clustered | VC_172_0 |
| Streptococcus~phage~K13 | Caudovirales | Siphoviridae | Unassigned | 172_0 | Clustered | VC_172_0 |
| Streptococcus~phage~M102 | Caudovirales | Siphoviridae | Unassigned | 298_0 | Clustered | VC_298_0 |
| Streptococcus~phage~M102AD | Caudovirales | Siphoviridae | Unassigned | 298_0 | Clustered | VC_298_0 |
| Streptococcus~phage~MM1 | Caudovirales | Siphoviridae | Unassigned | 125_0 | Clustered | VC_125_0 |
| Streptococcus~phage~P9 | Caudovirales | Siphoviridae | Unassigned | 146_0 | Clustered | VC_146_0 |
| Streptococcus~phage~PH10 | Caudovirales | Siphoviridae | Unassigned | 159_0 | Clustered | VC_159_0 |
| Streptococcus~phage~SM1 | Caudovirales | Siphoviridae | Unassigned | 256_0 | Clustered | VC_256_0 |
| Streptococcus~phage~SPQS1 | Caudovirales | Siphoviridae | Sap6virus | 138_0 | Clustered | VC_138_0 |
| Streptococcus~phage~SpSL1 | Caudovirales | Siphoviridae | Unassigned | 159_0 | Clustered | VC_159_0 |
| Streptococcus~phage~Str-PAP-1 | Caudovirales | Siphoviridae | Unassigned | 256_0 | Clustered | VC_256_0 |
| Streptococcus~phage~TP-778L | Caudovirales | Siphoviridae | Brussowvirus | 124_0 | Clustered | VC_124_0 |
| Streptococcus~phage~TP-J34 | Caudovirales | Siphoviridae | Brussowvirus | 124_0 | Clustered | VC_124_0 |
| Streptococcus~phage~phi3396 | Caudovirales | Siphoviridae | Unassigned | 157_0 | Clustered | VC_157_0 |
| Streptococcus~phage~phiARI0004 | Caudovirales | Siphoviridae | Unassigned | 172_0 | Clustered | VC_172_0 |
| Streptococcus~phage~phiARI0031 | Caudovirales | Siphoviridae | Unassigned | 172_0 | Clustered | VC_172_0 |
| Streptococcus~phage~phiARI0131-1 | Caudovirales | Siphoviridae | Unassigned | 172_0 | Clustered | VC_172_0 |
| Streptococcus~phage~phiARI0131-2 | Caudovirales | Siphoviridae | Unassigned | 159_0 | Clustered | VC_159_0 |
| Streptococcus~phage~phiARI0460-1 | Caudovirales | Siphoviridae | Unassigned | 172_0 | Clustered | VC_172_0 |
| Streptococcus~phage~phiARI0462 | Caudovirales | Siphoviridae | Unassigned | 172_0 | Clustered | VC_172_0 |
| Streptococcus~phage~phiARI0468-1 | Caudovirales | Siphoviridae | Unassigned | 172_0 | Clustered | VC_172_0 |
| Streptococcus~phage~phiARI0468-2 | Caudovirales | Siphoviridae | Unassigned | 172_0 | Clustered | VC_172_0 |
| Streptococcus~phage~phiARI0468-4 | Caudovirales | Siphoviridae | Unassigned | 125_0 | Clustered | VC_125_0 |
| Streptococcus~phage~phiARI0746 | Caudovirales | Siphoviridae | Unassigned | 172_0 | Clustered | VC_172_0 |
| Streptococcus~phage~phiARI0923 | Caudovirales | Siphoviridae | Unassigned | 159_0 | Clustered | VC_159_0 |
| Streptococcus~phage~phiBHN167 | Caudovirales | Siphoviridae | Unassigned | 125_0 | Clustered | VC_125_0 |
| Streptococcus~phage~phiNJ2 | Caudovirales | Siphoviridae | Unassigned | 125_0 | Clustered | VC_125_0 |
| Streptococcus~virus~2972 | Caudovirales | Siphoviridae | Sfi11virus | 124_0 | Clustered | VC_124_0 |
| Streptococcus~virus~858 | Caudovirales | Siphoviridae | Sfi11virus | 124_0 | Clustered | VC_124_0 |
| Streptococcus~virus~ALQ132 | Caudovirales | Siphoviridae | Sfi11virus | 124_0 | Clustered | VC_124_0 |
| Streptococcus~virus~O1205 | Caudovirales | Siphoviridae | Sfi11virus | 124_0 | Clustered | VC_124_0 |
| Streptococcus~virus~Sfi11 | Caudovirales | Siphoviridae | Sfi11virus | 124_0 | Clustered | VC_124_0 |
| Streptomyces~phage~Aaronocolus | Caudovirales | Siphoviridae | Likavirus | 357_0 | Clustered | VC_357_0 |
| Streptomyces~phage~Amela | Caudovirales | Siphoviridae | Camvirus | 357_0 | Clustered | VC_357_0 |
| Streptomyces~phage~BillNye | Caudovirales | Siphoviridae | Unassigned | 354_1 | Clustered/Singleton | VC_354_1 |
| Streptomyces~phage~Bing | Caudovirales | Siphoviridae | Bingvirus | 94_2 | Clustered | VC_94_2 |
| Streptomyces~phage~Caliburn | Caudovirales | Siphoviridae | Likavirus | 357_0 | Clustered | VC_357_0 |
| Streptomyces~phage~Danzina | Caudovirales | Siphoviridae | Likavirus | 357_0 | Clustered | VC_357_0 |
| Streptomyces~phage~DrGrey | Caudovirales | Siphoviridae | Rimavirus | 94_2 | Clustered | VC_94_2 |
| Streptomyces~phage~Hydra | Caudovirales | Siphoviridae | Likavirus | 357_0 | Clustered | VC_357_0 |
| Streptomyces~phage~Izzy | Caudovirales | Siphoviridae | Likavirus | 357_0 | Clustered | VC_357_0 |
| Streptomyces~phage~Jay2Jay | Caudovirales | Siphoviridae | Samistivirus | 354_0 | Clustered | VC_354_0 |
| Streptomyces~phage~Lannister | Caudovirales | Siphoviridae | Likavirus | 357_0 | Clustered | VC_357_0 |
| Streptomyces~phage~Lika | Caudovirales | Siphoviridae | Likavirus | 357_0 | Clustered | VC_357_0 |
| Streptomyces~phage~Mildred21 | Caudovirales | Siphoviridae | Samistivirus | 354_0 | Clustered | VC_354_0 |
| Streptomyces~phage~Nanodon | Caudovirales | Siphoviridae | Likavirus | 357_0 | Clustered | VC_357_0 |
| Streptomyces~phage~NootNoot | Caudovirales | Siphoviridae | Samistivirus | 354_0 | Clustered | VC_354_0 |
| Streptomyces~phage~Paradiddles | Caudovirales | Siphoviridae | Samistivirus | 354_0 | Clustered | VC_354_0 |
| Streptomyces~phage~Peebs | Caudovirales | Siphoviridae | Samistivirus | 354_0 | Clustered | VC_354_0 |
| Streptomyces~phage~R4 | Caudovirales | Siphoviridae | R4virus | 357_0 | Clustered | VC_357_0 |
| Streptomyces~phage~Rima | Caudovirales | Siphoviridae | Rimavirus | 94_2 | Clustered | VC_94_2 |
| Streptomyces~phage~SF1 | Caudovirales | Siphoviridae | Unassigned | 359_0 | Clustered | VC_359_0 |
| Streptomyces~phage~SF3 | Caudovirales | Siphoviridae | Unassigned | 359_0 | Clustered | VC_359_0 |
| Streptomyces~phage~SV1 | Caudovirales | Siphoviridae | Unassigned | 266_0 | Clustered | VC_266_0 |
| Streptomyces~phage~Samisti12 | Caudovirales | Siphoviridae | Samistivirus | 354_0 | Clustered | VC_354_0 |
| Streptomyces~phage~Scap1 | Caudovirales | Siphoviridae | Scapunavirus | 94_2 | Clustered | VC_94_2 |
| Streptomyces~phage~Sujidade | Caudovirales | Siphoviridae | Likavirus | 357_0 | Clustered | VC_357_0 |
| Streptomyces~phage~TP1604 | Caudovirales | Siphoviridae | Ydn12virus | 360_0 | Clustered | VC_360_0 |
| Streptomyces~phage~VWB | Caudovirales | Siphoviridae | Unassigned | 359_0 | Clustered | VC_359_0 |
| Streptomyces~phage~YDN12 | Caudovirales | Siphoviridae | Ydn12virus | 360_0 | Clustered | VC_360_0 |
| Streptomyces~phage~Zemlya | Caudovirales | Siphoviridae | Likavirus | 357_0 | Clustered | VC_357_0 |
| Streptomyces~phage~phiCAM | Caudovirales | Siphoviridae | Camvirus | 357_0 | Clustered | VC_357_0 |
| Streptomyces~phage~phiELB20 | Caudovirales | Siphoviridae | R4virus | 357_0 | Clustered | VC_357_0 |
| Streptomyces~phage~phiHau3 | Caudovirales | Siphoviridae | Unassigned | 357_0 | Clustered | VC_357_0 |
| Streptomyces~phage~phiSAJS1 | Caudovirales | Siphoviridae | Woodruffvirus | 360_0 | Clustered | VC_360_0 |
| Streptomyces~virus~TG1 | Caudovirales | Siphoviridae | Phic31virus | 358_0 | Clustered | VC_358_0 |
| Streptomyces~virus~phiBT1 | Caudovirales | Siphoviridae | Phic31virus | 358_0 | Clustered | VC_358_0 |
| Streptomyces~virus~phiC31 | Caudovirales | Siphoviridae | Phic31virus | 358_0 | Clustered | VC_358_0 |
| Stx2-converting~phage~86 | Caudovirales | Podoviridae | Traversvirus | 192_1 | Clustered | VC_192_1 |
| Stygiolobus~rod-shaped~virus | Ligamenvirales | Rudiviridae | Rudivirus | 19_0 | Clustered | VC_19_0 |
| Sulfitobacter~phage~NYA-2014a | Unassigned | Unassigned | Unassigned | 181_0 | Clustered | VC_181_0 |
| Sulfitobacter~phage~pCB2047-A | Unassigned | Unassigned | Unassigned | 181_0 | Clustered | VC_181_0 |
| Sulfitobacter~phage~pCB2047-C | Unassigned | Unassigned | Unassigned | 181_0 | Clustered | VC_181_0 |
| Sulfolobales~Virus~YNP2 | Unassigned | Unassigned | Unassigned | 23_0 | Clustered | VC_23_0 |
| Sulfolobales~virus~YNP1 | Unassigned | Unassigned | Unassigned | 23_0 | Clustered | VC_23_0 |
| Sulfolobus~islandicus~filamentous~virus | Ligamenvirales | Lipothrixviridae | Betalipothrixvirus | 18_0 | Clustered | VC_18_0 |
| Sulfolobus~islandicus~rod-shaped~virus~1 | Ligamenvirales | Rudiviridae | Rudivirus | 19_0 | Clustered | VC_19_0 |
| Sulfolobus~islandicus~rod-shaped~virus~10 | Ligamenvirales | Rudiviridae | Rudivirus | 19_0 | Clustered | VC_19_0 |
| Sulfolobus~islandicus~rod-shaped~virus~11 | Ligamenvirales | Rudiviridae | Rudivirus | 19_0 | Clustered | VC_19_0 |
| Sulfolobus~islandicus~rod-shaped~virus~2 | Ligamenvirales | Rudiviridae | Rudivirus | 19_0 | Clustered | VC_19_0 |
| Sulfolobus~islandicus~rod-shaped~virus~4 | Ligamenvirales | Rudiviridae | Rudivirus | 19_0 | Clustered | VC_19_0 |
| Sulfolobus~islandicus~rod-shaped~virus~5 | Ligamenvirales | Rudiviridae | Rudivirus | 19_0 | Clustered | VC_19_0 |
| Sulfolobus~islandicus~rod-shaped~virus~6 | Ligamenvirales | Rudiviridae | Rudivirus | 19_0 | Clustered | VC_19_0 |
| Sulfolobus~islandicus~rod-shaped~virus~7 | Ligamenvirales | Rudiviridae | Rudivirus | 19_0 | Clustered | VC_19_0 |
| Sulfolobus~islandicus~rod-shaped~virus~8 | Ligamenvirales | Rudiviridae | Rudivirus | 19_0 | Clustered | VC_19_0 |
| Sulfolobus~islandicus~rod-shaped~virus~9 | Ligamenvirales | Rudiviridae | Rudivirus | 19_0 | Clustered | VC_19_0 |
| Sulfolobus~islandicus~rudivirus~3 | Ligamenvirales | Rudiviridae | Rudivirus | 19_0 | Clustered | VC_19_0 |
| Sulfolobus~monocaudavirus~SMV1 | Unassigned | Bicaudaviridae | Unassigned | 20_0 | Clustered | VC_20_0 |
| Sulfolobus~monocaudavirus~SMV2 | Unassigned | Bicaudaviridae | Unassigned | 20_0 | Clustered | VC_20_0 |
| Sulfolobus~monocaudavirus~SMV3 | Unassigned | Bicaudaviridae | Unassigned | 20_0 | Clustered | VC_20_0 |
| Sulfolobus~monocaudavirus~SMV4 | Unassigned | Bicaudaviridae | Unassigned | 20_0 | Clustered | VC_20_0 |
| Sulfolobus~spindle-shaped~virus~1 | Unassigned | Fuselloviridae | Alphafusellovirus | 21_0 | Clustered | VC_21_0 |
| Sulfolobus~spindle-shaped~virus~2 | Unassigned | Fuselloviridae | Alphafusellovirus | 21_0 | Clustered | VC_21_0 |
| Sulfolobus~spindle-shaped~virus~4 | Unassigned | Fuselloviridae | Alphafusellovirus | 21_0 | Clustered | VC_21_0 |
| Sulfolobus~spindle-shaped~virus~5 | Unassigned | Fuselloviridae | Alphafusellovirus | 21_0 | Clustered | VC_21_0 |
| Sulfolobus~spindle-shaped~virus~6 | Unassigned | Fuselloviridae | Betafusellovirus | 21_0 | Clustered | VC_21_0 |
| Sulfolobus~spindle-shaped~virus~7 | Unassigned | Fuselloviridae | Alphafusellovirus | 21_0 | Clustered | VC_21_0 |
| Sulfolobus~turreted~icosahedral~virus~1 | Unassigned | Turriviridae | Alphaturrivirus | 361_0 | Clustered | VC_361_0 |
| Sulfolobus~turreted~icosahedral~virus~2 | Unassigned | Turriviridae | Alphaturrivirus | 361_0 | Clustered | VC_361_0 |
| Sulfolobus~virus~Kamchatka~1 | Unassigned | Fuselloviridae | Alphafusellovirus | 21_0 | Clustered | VC_21_0 |
| Sulfolobus~virus~Ragged~Hills | Unassigned | Fuselloviridae | Alphafusellovirus | 21_0 | Clustered | VC_21_0 |
| Sulfolobus~virus~STSV1 | Unassigned | Bicaudaviridae | Unassigned | 22_0 | Clustered | VC_22_0 |
| Sulfolobus~virus~STSV2 | Unassigned | Bicaudaviridae | Unassigned | 22_0 | Clustered | VC_22_0 |
| Synechococcus~phage~ACG-2014b | Caudovirales | Myoviridae | Unassigned | 25_3 | Clustered | VC_25_3 |
| Synechococcus~phage~ACG-2014c | Caudovirales | Myoviridae | Unassigned | 25_3 | Clustered | VC_25_3 |
| Synechococcus~phage~ACG-2014d | Caudovirales | Myoviridae | Unassigned | 25_31 | Clustered/Singleton | VC_25_31 |
| Synechococcus~phage~ACG-2014e | Caudovirales | Myoviridae | Unassigned | 25_19 | Clustered | VC_25_19 |
| Synechococcus~phage~ACG-2014f | Caudovirales | Myoviridae | Unassigned | 25_25 | Clustered/Singleton | VC_25_25 |
| Synechococcus~phage~ACG-2014g | Caudovirales | Myoviridae | Unassigned | 25_3 | Clustered | VC_25_3 |
| Synechococcus~phage~ACG-2014h | Caudovirales | Myoviridae | Unassigned | 25_20 | Clustered/Singleton | VC_25_20 |
| Synechococcus~phage~ACG-2014i | Caudovirales | Myoviridae | Unassigned | 25_19 | Clustered | VC_25_19 |
| Synechococcus~phage~ACG-2014j | Caudovirales | Myoviridae | Unassigned | 25_19 | Clustered | VC_25_19 |
| Synechococcus~phage~S-CAM1 | Caudovirales | Myoviridae | Unassigned | 25_13 | Clustered | VC_25_13 |
| Synechococcus~phage~S-CAM22 | Caudovirales | Myoviridae | Unassigned | 25_5 | Clustered | VC_25_5 |
| Synechococcus~phage~S-CAM3 | Caudovirales | Myoviridae | Unassigned | 25_13 | Clustered | VC_25_13 |
| Synechococcus~phage~S-CAM4 | Caudovirales | Myoviridae | Unassigned | 25_19 | Clustered | VC_25_19 |
| Synechococcus~phage~S-CAM7 | Caudovirales | Myoviridae | Unassigned | 25_26 | Clustered/Singleton | VC_25_26 |
| Synechococcus~phage~S-CAM8 | Caudovirales | Myoviridae | Unassigned | 25_3 | Clustered | VC_25_3 |
| Synechococcus~phage~S-CAM9 | Caudovirales | Myoviridae | Unassigned | 25_27 | Clustered/Singleton | VC_25_27 |
| Synechococcus~phage~S-CBP1 | Caudovirales | Podoviridae | Unassigned | 211_0 | Clustered | VC_211_0 |
| Synechococcus~phage~S-CBP2 | Caudovirales | Podoviridae | Unassigned | 211_0 | Clustered | VC_211_0 |
| Synechococcus~phage~S-CBP3 | Caudovirales | Podoviridae | Unassigned | 211_0 | Clustered | VC_211_0 |
| Synechococcus~phage~S-CBP4 | Caudovirales | Podoviridae | Unassigned | 211_0 | Clustered | VC_211_0 |
| Synechococcus~phage~S-CBP42 | Caudovirales | Podoviridae | Unassigned | 211_0 | Clustered | VC_211_0 |
| Synechococcus~phage~S-CBS1 | Caudovirales | Siphoviridae | Unassigned | 238_0 | Clustered | VC_238_0 |
| Synechococcus~phage~S-CBS2 | Caudovirales | Siphoviridae | Unassigned | 239_0 | Clustered | VC_239_0 |
| Synechococcus~phage~S-CBS3 | Caudovirales | Siphoviridae | Unassigned | 238_0 | Clustered | VC_238_0 |
| Synechococcus~phage~S-IOM18 | Caudovirales | Myoviridae | Unassigned | 25_5 | Clustered | VC_25_5 |
| Synechococcus~phage~S-MbCM100 | Caudovirales | Myoviridae | Unassigned | 25_3 | Clustered | VC_25_3 |
| Synechococcus~phage~S-PM2 | Caudovirales | Myoviridae | Unassigned | 25_16 | Clustered/Singleton | VC_25_16 |
| Synechococcus~phage~S-RIM2~R1_1999 | Caudovirales | Myoviridae | Unassigned | 25_2 | Clustered | VC_25_2 |
| Synechococcus~phage~S-RIM8~A.HR1 | Caudovirales | Myoviridae | Unassigned | 25_4 | Clustered | VC_25_4 |
| Synechococcus~phage~S-RIP1 | Caudovirales | Podoviridae | Unassigned | 211_0 | Clustered | VC_211_0 |
| Synechococcus~phage~S-RIP2 | Caudovirales | Podoviridae | Unassigned | 211_0 | Clustered | VC_211_0 |
| Synechococcus~phage~S-RSM4 | Caudovirales | Myoviridae | Unassigned | 25_17 | Clustered/Singleton | VC_25_17 |
| Synechococcus~phage~S-SKS1 | Caudovirales | Siphoviridae | Unassigned | 25_29 | Clustered/Singleton | VC_25_29 |
| Synechococcus~phage~S-SM1 | Caudovirales | Myoviridae | Unassigned | 25_34 | Clustered/Singleton | VC_25_34 |
| Synechococcus~phage~S-SM2 | Caudovirales | Myoviridae | Unassigned | 25_28 | Clustered/Singleton | VC_25_28 |
| Synechococcus~phage~S-SSM4 | Caudovirales | Myoviridae | Unassigned | 25_32 | Clustered/Singleton | VC_25_32 |
| Synechococcus~phage~S-SSM5 | Caudovirales | Myoviridae | Unassigned | 25_35 | Clustered/Singleton | VC_25_35 |
| Synechococcus~phage~S-SSM7 | Caudovirales | Myoviridae | Unassigned | 25_23 | Clustered/Singleton | VC_25_23 |
| Synechococcus~phage~S-ShM2 | Caudovirales | Myoviridae | Unassigned | 25_7 | Clustered/Singleton | VC_25_7 |
| Synechococcus~phage~S-WAM1 | Caudovirales | Myoviridae | Unassigned | 25_30 | Clustered/Singleton | VC_25_30 |
| Synechococcus~phage~S-WAM2 | Caudovirales | Myoviridae | Unassigned | 25_18 | Clustered/Singleton | VC_25_18 |
| Synechococcus~phage~Syn19 | Caudovirales | Myoviridae | Unassigned | 25_1 | Clustered/Singleton | VC_25_1 |
| Synechococcus~phage~metaG-MbCM1 | Caudovirales | Myoviridae | Unassigned | 25_9 | Clustered | VC_25_9 |
| Synechococcus~phage~syn9 | Caudovirales | Myoviridae | Unassigned | 25_6 | Clustered/Singleton | VC_25_6 |
| Synechococcus~virus~P60 | Caudovirales | Podoviridae | Unassigned | 211_0 | Clustered | VC_211_0 |
| Synechococcus~virus~Syn5 | Caudovirales | Podoviridae | Unassigned | 211_0 | Clustered | VC_211_0 |
| Thermoanaerobacterium~phage~THSA-485A | Caudovirales | Siphoviridae | Unassigned | 337_0 | Clustered | VC_337_0 |
| Thermoproteus~tenax~spherical~virus~1 | Unassigned | Globuloviridae | Globulovirus | 349_0 | Clustered | VC_349_0 |
| Thermus~phage~TMA | Caudovirales | Myoviridae | Unassigned | 362_0 | Clustered | VC_362_0 |
| Thermus~phage~phiYS40 | Caudovirales | Myoviridae | Unassigned | 362_0 | Clustered | VC_362_0 |
| Thermus~virus~IN93 | Unassigned | Sphaerolipoviridae | Gammasphaerolipovirus | 363_0 | Clustered | VC_363_0 |
| Thermus~virus~P23-45 | Caudovirales | Siphoviridae | P23virus | 364_0 | Clustered | VC_364_0 |
| Thermus~virus~P23-77 | Unassigned | Sphaerolipoviridae | Gammasphaerolipovirus | 363_0 | Clustered | VC_363_0 |
| Thermus~virus~P74-26 | Caudovirales | Siphoviridae | P23virus | 364_0 | Clustered | VC_364_0 |
| Tsukamurella~phage~TIN2 | Caudovirales | Siphoviridae | Tin2virus | 278_1 | Clustered | VC_278_1 |
| Tsukamurella~phage~TIN3 | Caudovirales | Siphoviridae | Tin2virus | 278_1 | Clustered | VC_278_1 |
| Tsukamurella~phage~TIN4 | Caudovirales | Siphoviridae | Tin2virus | 278_1 | Clustered | VC_278_1 |
| Uncultured~phage~WW-nAnB~strain~2 | Unassigned | Inoviridae | Unassigned | 365_0 | Clustered | VC_365_0 |
| Uncultured~phage~WW-nAnB~strain~3 | Unassigned | Inoviridae | Unassigned | 365_0 | Clustered | VC_365_0 |
| Vibrio~phage~11895-B1 | Caudovirales | Myoviridae | Unassigned | 227_3 | Clustered/Singleton | VC_227_3 |
| Vibrio~phage~Aphrodite1 | Caudovirales | Myoviridae | Aphroditevirus | 231_0 | Clustered | VC_231_0 |
| Vibrio~phage~CHOED | Caudovirales | Podoviridae | Unassigned | 201_0 | Clustered | VC_201_0 |
| Vibrio~phage~CP-T1 | Caudovirales | Myoviridae | Unassigned | 65_0 | Clustered | VC_65_0 |
| Vibrio~phage~Ceto | Caudovirales | Siphoviridae | Cetovirus | 262_1 | Clustered | VC_262_1 |
| Vibrio~phage~ICP1 | Caudovirales | Myoviridae | Unassigned | 227_4 | Clustered/Singleton | VC_227_4 |
| Vibrio~phage~ICP2 | Caudovirales | Podoviridae | Unassigned | 202_0 | Clustered | VC_202_0 |
| Vibrio~phage~ICP2_2013_A_Haiti | Caudovirales | Podoviridae | Unassigned | 202_0 | Clustered | VC_202_0 |
| Vibrio~phage~ICP3 | Caudovirales | Podoviridae | Teseptimavirus | 210_0 | Clustered | VC_210_0 |
| Vibrio~phage~J2 | Caudovirales | Podoviridae | Enhodamvirus | 366_0 | Clustered | VC_366_0 |
| Vibrio~phage~JSF10 | Caudovirales | Siphoviridae | Jesfedecavirus | 262_0 | Clustered | VC_262_0 |
| Vibrio~phage~N4 | Caudovirales | Podoviridae | Teseptimavirus | 210_0 | Clustered | VC_210_0 |
| Vibrio~phage~PWH3a-P1 | Unassigned | Unassigned | Unassigned | 227_1 | Clustered/Singleton | VC_227_1 |
| Vibrio~phage~QH | Caudovirales | Podoviridae | Enhodamvirus | 366_0 | Clustered | VC_366_0 |
| Vibrio~phage~SHOU24 | Caudovirales | Siphoviridae | Unassigned | 248_0 | Clustered | VC_248_0 |
| Vibrio~phage~SIO-2 | Caudovirales | Siphoviridae | Unassigned | 343_0 | Clustered | VC_343_0 |
| Vibrio~phage~SSP002 | Caudovirales | Siphoviridae | Ssp2virus | 369_0 | Clustered | VC_369_0 |
| Vibrio~phage~Thalassa | Caudovirales | Siphoviridae | Cetovirus | 262_1 | Clustered | VC_262_1 |
| Vibrio~phage~VEJphi | Unassigned | Inoviridae | Fibrovirus | 370_0 | Clustered | VC_370_0 |
| Vibrio~phage~VFJ | Unassigned | Inoviridae | Saetivirus | 371_0 | Clustered | VC_371_0 |
| Vibrio~phage~VGJphi | Unassigned | Inoviridae | Fibrovirus | 370_0 | Clustered | VC_370_0 |
| Vibrio~phage~VHML | Caudovirales | Myoviridae | Vhmlvirus | 105_0 | Clustered | VC_105_0 |
| Vibrio~phage~VP2 | Caudovirales | Podoviridae | Vp5virus | 366_0 | Clustered | VC_366_0 |
| Vibrio~phage~VP4B | Caudovirales | Myoviridae | Tidunavirus | 231_0 | Clustered | VC_231_0 |
| Vibrio~phage~VP5 | Caudovirales | Podoviridae | Vp5virus | 366_0 | Clustered | VC_366_0 |
| Vibrio~phage~VP585 | Caudovirales | Myoviridae | Vhmlvirus | 105_0 | Clustered | VC_105_0 |
| Vibrio~phage~VP93 | Caudovirales | Podoviridae | Unassigned | 31_0 | Clustered | VC_31_0 |
| Vibrio~phage~VPMS1 | Caudovirales | Podoviridae | Kafunavirus | 243_0 | Clustered | VC_243_0 |
| Vibrio~phage~VPUSM~8 | Caudovirales | Myoviridae | Hpunavirus | 70_0 | Clustered | VC_70_0 |
| Vibrio~phage~VSK | Unassigned | Inoviridae | Fibrovirus | 370_0 | Clustered | VC_370_0 |
| Vibrio~phage~VfO3K6 | Unassigned | Inoviridae | Unassigned | 368_0 | Clustered | VC_368_0 |
| Vibrio~phage~VfO4K68 | Unassigned | Inoviridae | Unassigned | 368_0 | Clustered | VC_368_0 |
| Vibrio~phage~X29 | Caudovirales | Myoviridae | Unassigned | 166_0 | Clustered | VC_166_0 |
| Vibrio~phage~eugene~12A10 | Unassigned | Unassigned | Unassigned | 227_0 | Clustered | VC_227_0 |
| Vibrio~phage~fs2 | Unassigned | Inoviridae | Saetivirus | 371_0 | Clustered | VC_371_0 |
| Vibrio~phage~helene~12B3 | Unassigned | Unassigned | Unassigned | 227_0 | Clustered | VC_227_0 |
| Vibrio~phage~pTD1 | Caudovirales | Myoviridae | Tidunavirus | 231_0 | Clustered | VC_231_0 |
| Vibrio~phage~pVp-1 | Caudovirales | Siphoviridae | Cetovirus | 262_1 | Clustered | VC_262_1 |
| Vibrio~phage~pYD21-A | Caudovirales | Siphoviridae | Unassigned | 60_0 | Clustered | VC_60_0 |
| Vibrio~phage~pYD38-A | Caudovirales | Siphoviridae | Roufvirus | 61_0 | Clustered | VC_61_0 |
| Vibrio~phage~phi~3 | Caudovirales | Siphoviridae | Jesfedecavirus | 262_0 | Clustered | VC_262_0 |
| Vibrio~phage~phi-A318 | Caudovirales | Podoviridae | Zindervirus | 254_0 | Clustered | VC_254_0 |
| Vibrio~phage~phiVC8 | Caudovirales | Podoviridae | Vp5virus | 366_0 | Clustered | VC_366_0 |
| Vibrio~phage~qdvp001 | Unassigned | Unassigned | Unassigned | 227_2 | Clustered/Singleton | VC_227_2 |
| Vibrio~phage~vB_VchM-138 | Caudovirales | Myoviridae | Unassigned | 65_0 | Clustered | VC_65_0 |
| Vibrio~phage~vB_VpaM_MAR | Caudovirales | Myoviridae | Vhmlvirus | 105_0 | Clustered | VC_105_0 |
| Vibrio~phage~vB_VpaS_MAR10 | Caudovirales | Siphoviridae | Ssp2virus | 369_0 | Clustered | VC_369_0 |
| Vibrio~virus~K139 | Caudovirales | Myoviridae | Hp1virus | 70_0 | Clustered | VC_70_0 |
| Vibrio~virus~VP882 | Caudovirales | Myoviridae | Hapunavirus | 247_0 | Clustered | VC_247_0 |
| Vibrio~virus~fs1 | Unassigned | Inoviridae | Fibrovirus | 370_0 | Clustered | VC_370_0 |
| Vibriophage~VP4 | Caudovirales | Podoviridae | Teseptimavirus | 210_0 | Clustered | VC_210_0 |
| Xanthomonas~citri~phage~CP2 | Caudovirales | Podoviridae | Unassigned | 161_0 | Clustered | VC_161_0 |
| Xanthomonas~phage~f20-Xaj | Caudovirales | Podoviridae | Pradovirus | 67_0 | Clustered | VC_67_0 |
| Xanthomonas~phage~f30-Xaj | Caudovirales | Podoviridae | Pradovirus | 67_0 | Clustered | VC_67_0 |
| Xanthomonas~phage~vB_XveM_DIBBI | Caudovirales | Myoviridae | Unassigned | 216_0 | Clustered | VC_216_0 |
| Xanthomonas~virus~CP1 | Caudovirales | Siphoviridae | Xp10virus | 81_0 | Clustered | VC_81_0 |
| Xanthomonas~virus~OP1 | Caudovirales | Siphoviridae | Xp10virus | 81_0 | Clustered | VC_81_0 |
| Xanthomonas~virus~Xop411 | Caudovirales | Siphoviridae | Xp10virus | 81_0 | Clustered | VC_81_0 |
| Xanthomonas~virus~Xp10 | Caudovirales | Siphoviridae | Xp10virus | 81_0 | Clustered | VC_81_0 |
| Xanthomonas~virus~phil7 | Caudovirales | Siphoviridae | Xp10virus | 81_0 | Clustered | VC_81_0 |
| Xylella~phage~Paz | Caudovirales | Podoviridae | Pradovirus | 67_0 | Clustered | VC_67_0 |
| Xylella~phage~Prado | Caudovirales | Podoviridae | Pradovirus | 67_0 | Clustered | VC_67_0 |
| Yersinia~phage~Berlin | Caudovirales | Podoviridae | Teseptimavirus | 210_0 | Clustered | VC_210_0 |
| Yersinia~phage~PST | Caudovirales | Myoviridae | T4virus | 34_5 | Clustered | VC_34_5 |
| Yersinia~phage~PY54 | Caudovirales | Siphoviridae | Unassigned | 185_0 | Clustered | VC_185_0 |
| Yersinia~phage~Yep-phi | Caudovirales | Podoviridae | Teseptimavirus | 210_0 | Clustered | VC_210_0 |
| Yersinia~phage~Yepe2 | Caudovirales | Podoviridae | Teseptimavirus | 210_0 | Clustered | VC_210_0 |
| Yersinia~phage~fHe-Yen9-04 | Caudovirales | Myoviridae | Eneladusvirus | 72_3 | Clustered/Singleton | VC_72_3 |
| Yersinia~phage~phi80-18 | Caudovirales | Podoviridae | Unassigned | 68_0 | Clustered | VC_68_0 |
| Yersinia~phage~phiA1122 | Caudovirales | Podoviridae | Teseptimavirus | 210_0 | Clustered | VC_210_0 |
| Yersinia~phage~phiD1 | Caudovirales | Myoviridae | T4virus | 34_5 | Clustered | VC_34_5 |
| Yersinia~phage~phiR1-37 | Caudovirales | Myoviridae | Unassigned | 109_1 | Clustered/Singleton | VC_109_1 |
| Yersinia~phage~phiR1-RT | Caudovirales | Myoviridae | Tg1virus | 34_11 | Clustered | VC_34_11 |
| Yersinia~phage~phiR201 | Caudovirales | Siphoviridae | Tequintavirus | 261_1 | Clustered | VC_261_1 |
| Yersinia~phage~phiYeO3-12 | Caudovirales | Podoviridae | Teseptimavirus | 210_0 | Clustered | VC_210_0 |
| Yersinia~phage~vB_YenM_TG1 | Caudovirales | Myoviridae | Tg1virus | 34_11 | Clustered | VC_34_11 |
| Yersinia~phage~vB_YenP_AP10 | Caudovirales | Podoviridae | Teseptimavirus | 210_0 | Clustered | VC_210_0 |
| Yersinia~phage~vB_YenP_AP5 | Caudovirales | Podoviridae | Teseptimavirus | 210_0 | Clustered | VC_210_0 |
| Yersinia~phage~vB_YenP_ISAO8 | Caudovirales | Podoviridae | Unassigned | 68_0 | Clustered | VC_68_0 |
| Yersinia~virus~L413C | Caudovirales | Myoviridae | P2virus | 69_0 | Clustered | VC_69_0 |
| alaska_puertorico_377 | Unassigned | Unassigned | Unassigned | 355_0 | Clustered | VC_355_0 |
| alaska_puertorico_431 | Unassigned | Unassigned | Unassigned | 372_0 | Clustered | VC_372_0 |
| alaska_puertorico_612 | Unassigned | Unassigned | Unassigned | 355_0 | Clustered | VC_355_0 |
| alaska_puertorico_799 | Unassigned | Unassigned | Unassigned | 355_0 | Clustered | VC_355_0 |
| alaska_puertorico_801 | Unassigned | Unassigned | Unassigned | 372_0 | Clustered | VC_372_0 |
| alaska_puertorico_880 | Unassigned | Unassigned | Unassigned | 355_0 | Clustered | VC_355_0 |
| gary_all20_10349 | Unassigned | Unassigned | Unassigned | 116_0 | Clustered | VC_116_0 |
| gary_all20_11343 | Unassigned | Unassigned | Unassigned | 117_0 | Clustered | VC_117_0 |
| gary_all20_11399 | Unassigned | Unassigned | Unassigned | 117_0 | Clustered | VC_117_0 |
| gary_all20_12995 | Unassigned | Unassigned | Unassigned | 117_0 | Clustered | VC_117_0 |
| gary_all20_20798 | Unassigned | Unassigned | Unassigned | 117_0 | Clustered | VC_117_0 |
| gary_all20_21729 | Unassigned | Unassigned | Unassigned | 116_0 | Clustered | VC_116_0 |
| gary_all20_23107 | Unassigned | Unassigned | Unassigned | 373_0 | Clustered | VC_373_0 |
| gary_all20_23251 | Unassigned | Unassigned | Unassigned | 55_0 | Clustered | VC_55_0 |
| gary_all20_3439 | Unassigned | Unassigned | Unassigned | 117_0 | Clustered | VC_117_0 |
| gary_all20_6694 | Unassigned | Unassigned | Unassigned | 373_0 | Clustered | VC_373_0 |
| gary_all20_7195 | Unassigned | Unassigned | Unassigned | 117_0 | Clustered | VC_117_0 |
| uncultured~phage~WW-nAnB | Unassigned | Inoviridae | Unassigned | 365_0 | Clustered | VC_365_0 |
| virsorter_curated_4650 | Unassigned | Unassigned | Unassigned | 118_0 | Clustered | VC_118_0 |
| Acholeplasma~virus~L2 | Unassigned | Plasmaviridae | Plasmavirus |  | Singleton |  |
| Acholeplasma~virus~MV-L51 | Unassigned | Inoviridae | Plectrovirus |  | Singleton |  |
| Achromobacter~phage~JWF | Caudovirales | Siphoviridae | Unassigned |  | Singleton |  |
| Achromobacter~phage~phiAxp-2 | Caudovirales | Siphoviridae | Unassigned |  | Outlier |  |
| Acidianus~filamentous~virus~1 | Ligamenvirales | Lipothrixviridae | Gammalipothrixvirus |  | Outlier |  |
| Acidianus~filamentous~virus~2 | Ligamenvirales | Lipothrixviridae | Deltalipothrixvirus |  | Outlier |  |
| Acidianus~rod-shaped~virus~1 | Ligamenvirales | Rudiviridae | Rudivirus |  | Outlier |  |
| Acidianus~rod-shaped~virus~2 | Ligamenvirales | Rudiviridae | Rudivirus |  | Outlier |  |
| Acidianus~tailed~spindle~virus | Unassigned | Bicaudaviridae | Unassigned |  | Outlier |  |
| Acidianus~two-tailed~virus | Unassigned | Bicaudaviridae | Bicaudavirus |  | Outlier |  |
| Acidovorax~phage~ACP17 | Caudovirales | Myoviridae | Busanvirus |  | Outlier |  |
| Acinetobacter~phage~AP205 | Unassigned | Leviviridae | Unassigned |  | Outlier |  |
| Acinetobacter~phage~Ac42 | Caudovirales | Myoviridae | Unassigned |  | Outlier |  |
| Acinetobacter~phage~Acj61 | Caudovirales | Myoviridae | Unassigned |  | Outlier |  |
| Acinetobacter~phage~Acj9 | Caudovirales | Myoviridae | Unassigned |  | Outlier |  |
| Acinetobacter~phage~Presley | Caudovirales | Podoviridae | Unassigned |  | Outlier |  |
| Acinetobacter~phage~YMC13/03/R2096 | Caudovirales | Myoviridae | Unassigned |  | Outlier |  |
| Acinetobacter~phage~ZZ1 | Caudovirales | Myoviridae | Unassigned |  | Outlier |  |
| Acinetobacter~phage~phiAC-1 | Caudovirales | Myoviridae | Unassigned |  | Outlier |  |
| Acinetobacter~phage~vB_AbaM_ME3 | Caudovirales | Myoviridae | Metrivirus |  | Singleton |  |
| Acinetobacter~virus~133 | Caudovirales | Myoviridae | Unassigned |  | Outlier |  |
| Actinomyces~virus~Av1 | Caudovirales | Podoviridae | Unassigned |  | Outlier |  |
| Actinoplanes~phage~phiAsp2 | Caudovirales | Siphoviridae | Unassigned |  | Outlier |  |
| Aeromonas~phage~vB_AsaM-56 | Caudovirales | Myoviridae | Popoffvirus |  | Outlier |  |
| Aeropyrum~globular~virus~1 | Unassigned | Unassigned | Unassigned |  | Singleton |  |
| Aeropyrum~pernix~ovoid~virus~1 | Unassigned | Guttaviridae | Betaguttavirus |  | Singleton |  |
| Aeropyrum~pernix~spindle-shaped~virus~1 | Unassigned | Unassigned | Unassigned |  | Singleton |  |
| Agrobacterium~phage~Atu_ph07 | Caudovirales | Myoviridae | Polybotosvirus |  | Outlier |  |
| Alces~alces~faeces~associated~microvirus~MP15~5067 | Unassigned | Microviridae | Unassigned |  | Outlier |  |
| Alces~alces~faeces~associated~microvirus~MP18~4940 | Unassigned | Microviridae | Unassigned |  | Outlier |  |
| Alces~alces~faeces~associated~microvirus~MP21~4718 | Unassigned | Microviridae | Unassigned |  | Outlier |  |
| Alces~alces~faeces~associated~microvirus~MP3~6497 | Unassigned | Microviridae | Unassigned |  | Outlier |  |
| Alphaproteobacteria~virus~phiJl001 | Caudovirales | Siphoviridae | Yuavirus |  | Outlier |  |
| Alteromonas~phage~vB_AmaP_AD45-P1 | Caudovirales | Podoviridae | Unassigned |  | Outlier |  |
| Anabaena~phage~A-4L | Caudovirales | Podoviridae | Unassigned |  | Outlier |  |
| Arthrobacter~phage~Decurro | Caudovirales | Siphoviridae | Decurrovirus |  | Outlier |  |
| Arthrobacter~phage~Laroye | Caudovirales | Siphoviridae | Laroyevirus |  | Outlier |  |
| Arthrobacter~phage~Tank | Caudovirales | Siphoviridae | Tankvirus |  | Outlier |  |
| Arthrobacter~phage~vB_ArS-ArV2 | Caudovirales | Siphoviridae | Unassigned |  | Outlier |  |
| Aurantimonas~phage~AmM-1 | Caudovirales | Podoviridae | Unassigned |  | Outlier |  |
| Azospirillum~phage~Cd | Caudovirales | Siphoviridae | Unassigned |  | Outlier |  |
| Bacillus~phage~AvesoBmore | Caudovirales | Myoviridae | B4virus |  | Overlap (VC_111/VC_112) |  |
| Bacillus~phage~B4 | Caudovirales | Myoviridae | B4virus |  | Overlap (VC_111/VC_112) |  |
| Bacillus~phage~BCD7 | Caudovirales | Myoviridae | Unassigned |  | Outlier |  |
| Bacillus~phage~BCJA1c | Caudovirales | Siphoviridae | Unassigned |  | Outlier |  |
| Bacillus~phage~BCP78 | Caudovirales | Myoviridae | Tsarbombavirus |  | Overlap (VC_111/VC_112) |  |
| Bacillus~phage~BCP8-2 | Caudovirales | Myoviridae | Bc431virus |  | Overlap (VC_111/VC_112) |  |
| Bacillus~phage~BalMu-1 | Unassigned | Unassigned | Unassigned |  | Outlier |  |
| Bacillus~phage~Bastille | Caudovirales | Myoviridae | Bastillevirus |  | Overlap (VC_111/VC_112) |  |
| Bacillus~phage~Bcp1 | Caudovirales | Myoviridae | Bc431virus |  | Overlap (VC_111/VC_112) |  |
| Bacillus~phage~BigBertha | Caudovirales | Myoviridae | B4virus |  | Overlap (VC_111/VC_112) |  |
| Bacillus~phage~Bobb | Caudovirales | Myoviridae | Agatevirus |  | Overlap (VC_111/VC_112) |  |
| Bacillus~phage~Bp8p-C | Caudovirales | Myoviridae | Agatevirus |  | Overlap (VC_111/VC_112) |  |
| Bacillus~phage~CAM003 | Caudovirales | Myoviridae | Bastillevirus |  | Overlap (VC_111/VC_112) |  |
| Bacillus~phage~Deep~Blue | Caudovirales | Myoviridae | Bc431virus |  | Overlap (VC_111/VC_112) |  |
| Bacillus~phage~Eldridge | Caudovirales | Herelleviridae | Unassigned |  | Overlap (VC_111/VC_112) |  |
| Bacillus~phage~Evoli | Caudovirales | Herelleviridae | Bastillevirus |  | Overlap (VC_111/VC_112) |  |
| Bacillus~phage~Grass | Caudovirales | Myoviridae | Nit1virus |  | Overlap (VC_111/VC_112) |  |
| Bacillus~phage~Hoody~T | Caudovirales | Herelleviridae | Bastillevirus |  | Overlap (VC_111/VC_112) |  |
| Bacillus~phage~JBP901 | Caudovirales | Myoviridae | Bc431virus |  | Overlap (VC_111/VC_112) |  |
| Bacillus~phage~Mater | Caudovirales | Herelleviridae | Unassigned |  | Overlap (VC_111/VC_112) |  |
| Bacillus~phage~Moonbeam | Caudovirales | Herelleviridae | Unassigned |  | Overlap (VC_111/VC_112) |  |
| Bacillus~phage~PfEFR-5 | Caudovirales | Siphoviridae | Unassigned |  | Outlier |  |
| Bacillus~phage~Phrodo | Caudovirales | Herelleviridae | Bequatrovirus |  | Overlap (VC_111/VC_112) |  |
| Bacillus~phage~Riley | Caudovirales | Myoviridae | B4virus |  | Overlap (VC_111/VC_112) |  |
| Bacillus~phage~SIOphi | Caudovirales | Herelleviridae | Unassigned |  | Overlap (VC_111/VC_112) |  |
| Bacillus~phage~SP-10 | Caudovirales | Herelleviridae | Unassigned |  | Outlier |  |
| Bacillus~phage~SP-15 | Caudovirales | Myoviridae | Thornevirus |  | Overlap (VC_108/VC_109) |  |
| Bacillus~phage~SPG24 | Caudovirales | Myoviridae | Nit1virus |  | Outlier |  |
| Bacillus~phage~SPP1 | Caudovirales | Siphoviridae | Unassigned |  | Outlier |  |
| Bacillus~phage~Shbh1 | Caudovirales | Myoviridae | Shalavirus |  | Overlap (VC_111/VC_112) |  |
| Bacillus~phage~Spock | Caudovirales | Myoviridae | B4virus |  | Overlap (VC_111/VC_112) |  |
| Bacillus~phage~Troll | Caudovirales | Myoviridae | B4virus |  | Overlap (VC_111/VC_112) |  |
| Bacillus~phage~TsarBomba | Caudovirales | Myoviridae | Tsarbombavirus |  | Overlap (VC_111/VC_112) |  |
| Bacillus~phage~VMY22 | Caudovirales | Podoviridae | Salasvirus |  | Overlap (VC_52/VC_53) |  |
| Bacillus~phage~Waukesha92 | Caudovirales | Siphoviridae | Unassigned |  | Overlap (VC_130/VC_131) |  |
| Bacillus~phage~phBC6A51 | Caudovirales | Siphoviridae | Unassigned |  | Outlier |  |
| Bacillus~phage~phBC6A52 | Caudovirales | Siphoviridae | Unassigned |  | Outlier |  |
| Bacillus~phage~phi105 | Caudovirales | Siphoviridae | Unassigned |  | Outlier |  |
| Bacillus~phage~phi4B1 | Caudovirales | Siphoviridae | Unassigned |  | Outlier |  |
| Bacillus~phage~phi4J1 | Caudovirales | Siphoviridae | Unassigned |  | Overlap (VC_130/VC_131) |  |
| Bacillus~phage~phiAGATE | Caudovirales | Myoviridae | Agatevirus |  | Overlap (VC_111/VC_112) |  |
| Bacillus~phage~phiNIT1 | Caudovirales | Myoviridae | Nit1virus |  | Overlap (VC_111/VC_112) |  |
| Bacillus~phage~vB_BceM_Bc431v3 | Caudovirales | Myoviridae | Bc431virus |  | Overlap (VC_111/VC_112) |  |
| Bacillus~phage~vB_BhaS-171 | Caudovirales | Siphoviridae | Unassigned |  | Outlier |  |
| Bacillus~phage~vB_BtS_BMBtp3 | Caudovirales | Siphoviridae | Unassigned |  | Overlap (VC_130/VC_131) |  |
| Bacillus~virus~B103 | Caudovirales | Podoviridae | Phi29virus |  | Overlap (VC_52/VC_53) |  |
| Bacillus~virus~BM15 | Caudovirales | Myoviridae | Bc431virus |  | Overlap (VC_111/VC_112) |  |
| Bacillus~virus~G | Caudovirales | Myoviridae | Unassigned |  | Overlap (VC_108/VC_109) |  |
| Bacillus~virus~GA1 | Caudovirales | Podoviridae | Phi29virus |  | Overlap (VC_52/VC_53) |  |
| Bacillus~virus~phi29 | Caudovirales | Podoviridae | Phi29virus |  | Overlap (VC_52/VC_53) |  |
| Bdellovibrio~phage~phi1402 | Caudovirales | Myoviridae | Unassigned |  | Singleton |  |
| Bdellovibrio~phage~phi1422 | Caudovirales | Myoviridae | Unassigned |  | Outlier |  |
| Brochothrix~phage~A9 | Caudovirales | Herelleviridae | Unassigned |  | Outlier |  |
| Brochothrix~phage~BL3 | Caudovirales | Siphoviridae | Unassigned |  | Outlier |  |
| Burkholderia~phage~AH2 | Caudovirales | Siphoviridae | Ahduovirus |  | Outlier |  |
| Burkholderia~phage~BcepB1A | Caudovirales | Myoviridae | Unassigned |  | Outlier |  |
| Burkholderia~phage~BcepGomr | Caudovirales | Siphoviridae | Unassigned |  | Outlier |  |
| Burkholderia~phage~BcepNazgul | Caudovirales | Myoviridae | Nazgulvirus |  | Outlier |  |
| Burkholderia~phage~KS10 | Caudovirales | Myoviridae | Unassigned |  | Outlier |  |
| Burkholderia~virus~BcepF1 | Caudovirales | Myoviridae | Pbunavirus |  | Outlier |  |
| Burkholderia~virus~BcepMu | Caudovirales | Myoviridae | Bcepmuvirus |  | Overlap (VC_188/VC_190) |  |
| Burkholderia~virus~phiE255 | Caudovirales | Myoviridae | Bcepmuvirus |  | Overlap (VC_188/VC_190) |  |
| Caulobacter~phage~Cr30 | Caudovirales | Myoviridae | Unassigned |  | Outlier |  |
| Cellulophaga~phage~phi14:2 | Unassigned | Unassigned | Unassigned |  | Outlier |  |
| Cellulophaga~phage~phi38:1 | Caudovirales | Podoviridae | Unassigned |  | Outlier |  |
| Cellulophaga~phage~phi39:1 | Caudovirales | Siphoviridae | Unassigned |  | Singleton |  |
| Cellulophaga~phage~phi46:1 | Caudovirales | Siphoviridae | Unassigned |  | Singleton |  |
| Cellulophaga~phage~phi48:2 | Unassigned | Unassigned | Unassigned |  | Singleton |  |
| Cellulophaga~phage~phiSM | Caudovirales | Myoviridae | Unassigned |  | Outlier |  |
| Cellulophaga~phage~phiST | Caudovirales | Siphoviridae | Cbastvirus |  | Singleton |  |
| Chimpanzee~faeces~associated~microphage~2 | Unassigned | Microviridae | Unassigned |  | Outlier |  |
| Chlamydia~virus~Chp1 | Unassigned | Microviridae | Chlamydiamicrovirus |  | Singleton |  |
| Clostridium~phage~c-st | Caudovirales | Myoviridae | Unassigned |  | Outlier |  |
| Clostridium~phage~phi24R | Caudovirales | Podoviridae | Unassigned |  | Outlier |  |
| Clostridium~phage~phi8074-B1 | Caudovirales | Siphoviridae | Unassigned |  | Outlier |  |
| Clostridium~phage~phiCD211 | Caudovirales | Siphoviridae | Unassigned |  | Outlier |  |
| Clostridium~phage~phiCD6356 | Caudovirales | Siphoviridae | Unassigned |  | Outlier |  |
| Clostridium~phage~phiCT453A | Caudovirales | Myoviridae | Unassigned |  | Outlier |  |
| Clostridium~phage~phiCT9441A | Caudovirales | Myoviridae | Unassigned |  | Outlier |  |
| Clostridium~phage~vB_CpeS-CP51 | Caudovirales | Siphoviridae | Unassigned |  | Outlier |  |
| Colwellia~phage~9A | Caudovirales | Siphoviridae | Unassigned |  | Singleton |  |
| Corynebacterium~phage~BFK20 | Caudovirales | Siphoviridae | Sasvirus |  | Outlier |  |
| Corynebacterium~phage~P1201 | Caudovirales | Siphoviridae | Chunghsingvirus |  | Outlier |  |
| Croceibacter~phage~P2559S | Caudovirales | Siphoviridae | Unassigned |  | Outlier |  |
| Croceibacter~phage~P2559Y | Caudovirales | Siphoviridae | Unassigned |  | Outlier |  |
| Cronobacter~phage~ENT39118 | Caudovirales | Siphoviridae | Unassigned |  | Outlier |  |
| Cronobacter~phage~ENT47670 | Caudovirales | Myoviridae | Unassigned |  | Outlier |  |
| Cronobacter~phage~phiES15 | Caudovirales | Siphoviridae | Unassigned |  | Outlier |  |
| Cyanophage~KBS-S-2A | Unassigned | Unassigned | Unassigned |  | Outlier |  |
| Cyanophage~S-TIM5 | Caudovirales | Myoviridae | Unassigned |  | Outlier |  |
| Deftia~phage~phiW-14 | Caudovirales | Myoviridae | Unassigned |  | Outlier |  |
| Dinoroseobacter~phage~DFL12phi1 | Caudovirales | Podoviridae | Dfl12virus |  | Overlap (VC_8/VC_11) |  |
| Dinoroseobacter~phage~vB_DshS-R5C | Caudovirales | Siphoviridae | Nanhaivirus |  | Outlier |  |
| EarthsVirome_48737 | Unassigned | Unassigned | Unassigned |  | Outlier |  |
| Edwardsiella~phage~GF-2 | Caudovirales | Myoviridae | Gofduovirus |  | Outlier |  |
| Eel~River~basin~pequenovirus | Unassigned | Microviridae | Unassigned |  | Outlier |  |
| Enterobacteria~phage~BP-4795 | Caudovirales | Siphoviridae | Unassigned |  | Outlier |  |
| Enterobacteria~phage~DE3 | Caudovirales | Siphoviridae | Lambdavirus |  | Overlap (VC_185/VC_233) |  |
| Enterobacteria~phage~ES18 | Caudovirales | Siphoviridae | Unassigned |  | Outlier |  |
| Enterobacteria~phage~HK225 | Caudovirales | Siphoviridae | Unassigned |  | Overlap (VC_184/VC_185/VC_233) |  |
| Enterobacteria~phage~IME10 | Caudovirales | Podoviridae | Lederbergvirus |  | Outlier |  |
| Enterobacteria~phage~Ike | Unassigned | Inoviridae | Lineavirus |  | Overlap (VC_252/VC_253) |  |
| Enterobacteria~phage~J8-65 | Caudovirales | Podoviridae | Phikmvvirus |  | Outlier |  |
| Enterobacteria~phage~MS2 | Unassigned | Leviviridae | Levivirus |  | Overlap (VC_198/VC_199) |  |
| Enterobacteria~phage~P4 | Caudovirales | Unassigned | Unassigned |  | Singleton |  |
| Enterobacteria~phage~SfI | Caudovirales | Myoviridae | Unassigned |  | Outlier |  |
| Enterobacteria~phage~YYZ-2008 | Caudovirales | Siphoviridae | Unassigned |  | Outlier |  |
| Enterobacteria~phage~cdtI | Caudovirales | Siphoviridae | Unassigned |  | Overlap (VC_64/VC_184/VC_185/VC_233) |  |
| Enterobacteria~phage~mEp043~c-1 | Caudovirales | Siphoviridae | Unassigned |  | Overlap (VC_177/VC_185) |  |
| Enterobacteria~phage~mEp237 | Caudovirales | Siphoviridae | Unassigned |  | Overlap (VC_184/VC_185/VC_233) |  |
| Enterobacteria~phage~mEp460 | Caudovirales | Siphoviridae | Unassigned |  | Overlap (VC_64/VC_184/VC_185/VC_233) |  |
| Enterobacteria~phage~phi80 | Caudovirales | Siphoviridae | Unassigned |  | Overlap (VC_184/VC_185/VC_233) |  |
| Enterobacterial~phage~mEp213 | Caudovirales | Siphoviridae | Unassigned |  | Overlap (VC_177/VC_185) |  |
| Enterobacterial~phage~mEp390 | Caudovirales | Siphoviridae | Unassigned |  | Outlier |  |
| Enterococcus~phage~ECP3 | Caudovirales | Herelleviridae | Kochikohdavirus |  | Overlap (VC_115/VC_116) |  |
| Enterococcus~phage~EF62phi | Caudovirales | Podoviridae | Unassigned |  | Singleton |  |
| Enterococcus~phage~EFAP-1 | Caudovirales | Siphoviridae | Efquatrovirus |  | Outlier |  |
| Enterococcus~phage~EFC-1 | Caudovirales | Siphoviridae | Unassigned |  | Overlap (VC_121/VC_175) |  |
| Enterococcus~phage~EFDG1 | Caudovirales | Herelleviridae | Unassigned |  | Overlap (VC_115/VC_116) |  |
| Enterococcus~phage~EFLK1 | Caudovirales | Herelleviridae | Kochikohdavirus |  | Overlap (VC_115/VC_116) |  |
| Enterococcus~phage~EFRM31 | Caudovirales | Siphoviridae | Efquatrovirus |  | Outlier |  |
| Enterococcus~phage~IME-EFm1 | Caudovirales | Siphoviridae | Efquatrovirus |  | Outlier |  |
| Enterococcus~phage~IME-EFm5 | Caudovirales | Siphoviridae | Efquatrovirus |  | Outlier |  |
| Enterococcus~phage~phiEF24C | Caudovirales | Herelleviridae | Kochikohdavirus |  | Overlap (VC_115/VC_116) |  |
| Enterococcus~phage~phiEf11 | Caudovirales | Siphoviridae | Unassigned |  | Overlap (VC_121/VC_175) |  |
| Enterococcus~phage~phiFL4A | Caudovirales | Siphoviridae | Unassigned |  | Outlier |  |
| Enterococcus~phage~vB_EfaS_IME197 | Caudovirales | Siphoviridae | Unassigned |  | Overlap (VC_121/VC_175) |  |
| Erwinia~phage~PEp14 | Caudovirales | Podoviridae | Unassigned |  | Outlier |  |
| Erwinia~phage~PhiEaH1 | Caudovirales | Myoviridae | Iapetusvirus |  | Outlier |  |
| Erysipelothrix~phage~SE-1 | Caudovirales | Siphoviridae | Unassigned |  | Outlier |  |
| Escherichia~phage~ECML-117 | Caudovirales | Myoviridae | Unassigned |  | Outlier |  |
| Escherichia~phage~HK629 | Caudovirales | Siphoviridae | Lambdavirus |  | Overlap (VC_185/VC_233) |  |
| Escherichia~phage~HK630 | Caudovirales | Siphoviridae | Lambdavirus |  | Overlap (VC_185/VC_233) |  |
| Escherichia~phage~HK639 | Caudovirales | Siphoviridae | Unassigned |  | Outlier |  |
| Escherichia~phage~HX01 | Caudovirales | Myoviridae | Rb69virus |  | Outlier |  |
| Escherichia~phage~Pollock | Caudovirales | Podoviridae | Sp58virus |  | Overlap (VC_8/VC_12) |  |
| Escherichia~virus~BZ13 | Unassigned | Leviviridae | Levivirus |  | Overlap (VC_198/VC_199) |  |
| Escherichia~virus~Lambda | Caudovirales | Siphoviridae | Lambdavirus |  | Overlap (VC_185/VC_233) |  |
| Escherichia~virus~N15 | Caudovirales | Siphoviridae | N15virus |  | Overlap (VC_184/VC_185/VC_233) |  |
| Flavobacterium~phage~11b | Caudovirales | Siphoviridae | Unassigned |  | Outlier |  |
| Gluconobacter~phage~GC1 | Unassigned | Tectiviridae | Gammatectivirus |  | Outlier |  |
| Gokushovirinae~GAIR4 | Unassigned | Microviridae | Unassigned |  | Singleton |  |
| Gokushovirinae~GNX3R | Unassigned | Microviridae | Unassigned |  | Singleton |  |
| Gordonia~phage~Bantam | Caudovirales | Siphoviridae | Bantamvirus |  | Outlier |  |
| Gordonia~phage~BetterKatz | Caudovirales | Siphoviridae | Betterkatzvirus |  | Outlier |  |
| Gordonia~phage~Eyre | Caudovirales | Siphoviridae | Eyrevirus |  | Outlier |  |
| Gordonia~phage~GMA6 | Caudovirales | Siphoviridae | Bendigovirus |  | Singleton |  |
| Gordonia~phage~Ghobes | Caudovirales | Siphoviridae | Ghobesvirus |  | Outlier |  |
| Gordonia~phage~JSwag | Caudovirales | Siphoviridae | Soupsvirus |  | Overlap (VC_283/VC_284) |  |
| Gordonia~phage~KatherineG | Caudovirales | Siphoviridae | Soupsvirus |  | Overlap (VC_283/VC_284) |  |
| Gordonia~phage~Nyceirae | Caudovirales | Siphoviridae | Nyceiraevirus |  | Outlier |  |
| Gordonia~phage~Orchid | Caudovirales | Siphoviridae | Orchidvirus |  | Outlier |  |
| Gordonia~phage~Remus | Caudovirales | Siphoviridae | Soupsvirus |  | Overlap (VC_283/VC_284) |  |
| Gordonia~phage~Rosalind | Caudovirales | Siphoviridae | Soupsvirus |  | Overlap (VC_283/VC_284) |  |
| Gordonia~phage~Soups | Caudovirales | Siphoviridae | Soupsvirus |  | Overlap (VC_283/VC_284) |  |
| Gordonia~phage~Sour | Caudovirales | Siphoviridae | Sourvirus |  | Overlap (VC_57/VC_59) |  |
| Gordonia~phage~Strosahl | Caudovirales | Siphoviridae | Soupsvirus |  | Overlap (VC_283/VC_284) |  |
| Gordonia~phage~Terapin | Caudovirales | Siphoviridae | Unassigned |  | Outlier |  |
| Gordonia~phage~Waits | Caudovirales | Siphoviridae | Soupsvirus |  | Overlap (VC_283/VC_284) |  |
| Gordonia~phage~Yvonnetastic | Caudovirales | Siphoviridae | Yvonnevirus |  | Outlier |  |
| Haemophilus~phage~SuMu | Caudovirales | Myoviridae | Unassigned |  | Outlier |  |
| Halobacterium~phage~phiH | Caudovirales | Myoviridae | Myohalovirus |  | Singleton |  |
| Halocynthia~phage~JM-2012 | Caudovirales | Myoviridae | Unassigned |  | Outlier |  |
| Halogeometricum~pleomorphic~virus~1 | Unassigned | Pleolipoviridae | Betapleolipovirus |  | Singleton |  |
| Halorubrum~pleomorphic~virus~3 | Unassigned | Pleolipoviridae | Betapleolipovirus |  | Singleton |  |
| Halovirus~HGTV-1 | Unassigned | Unassigned | Unassigned |  | Outlier |  |
| Halovirus~HHTV-1 | Unassigned | Unassigned | Unassigned |  | Singleton |  |
| Halovirus~HRTV-4 | Unassigned | Unassigned | Unassigned |  | Singleton |  |
| Halovirus~HRTV-7 | Unassigned | Unassigned | Unassigned |  | Overlap (VC_293/VC_294) |  |
| Halovirus~HSTV-1 | Unassigned | Unassigned | Unassigned |  | Singleton |  |
| Halovirus~HSTV-2 | Unassigned | Unassigned | Unassigned |  | Overlap (VC_293/VC_294) |  |
| Halovirus~VNH-1 | Unassigned | Unassigned | Unassigned |  | Singleton |  |
| His~1~virus | Unassigned | Unassigned | Salterprovirus |  | Singleton |  |
| His2~virus | Unassigned | Pleolipoviridae | Gammapleolipovirus |  | Singleton |  |
| Hydrogenobaculum~phage~1 | Unassigned | Unassigned | Unassigned |  | Singleton |  |
| Hyperthermophilic~Archaeal~Virus~1 | Unassigned | Unassigned | Unassigned |  | Singleton |  |
| Hyperthermophilic~Archaeal~Virus~2 | Unassigned | Unassigned | Unassigned |  | Singleton |  |
| Idiomarinaceae~phage~1N2-2 | Caudovirales | Siphoviridae | Unassigned |  | Overlap (VC_45/VC_180) |  |
| Klebsiella~phage~F19 | Caudovirales | Podoviridae | Kp34virus |  | Overlap (VC_32/VC_33) |  |
| Klebsiella~phage~K64-1 | Caudovirales | Myoviridae | Alcyoneusvirus |  | Outlier |  |
| Klebsiella~phage~KP34 | Caudovirales | Podoviridae | Kp34virus |  | Overlap (VC_32/VC_33) |  |
| Klebsiella~phage~Kp2 | Caudovirales | Podoviridae | Kp34virus |  | Overlap (VC_32/VC_33) |  |
| Klebsiella~phage~KpV41 | Caudovirales | Podoviridae | Kp34virus |  | Overlap (VC_32/VC_33) |  |
| Klebsiella~phage~KpV475 | Caudovirales | Podoviridae | Kp34virus |  | Overlap (VC_32/VC_33) |  |
| Klebsiella~phage~KpV71 | Caudovirales | Podoviridae | Kp34virus |  | Overlap (VC_32/VC_33) |  |
| Klebsiella~phage~NTUH-K2044-K1-1 | Caudovirales | Podoviridae | Kp34virus |  | Overlap (VC_32/VC_33) |  |
| Klebsiella~phage~vB_KpnP_SU503 | Caudovirales | Podoviridae | Kp34virus |  | Overlap (VC_32/VC_33) |  |
| Klebsiella~phage~vB_KpnP_SU552A | Caudovirales | Podoviridae | Kp34virus |  | Overlap (VC_32/VC_33) |  |
| Lactobacillus~phage~CL1 | Caudovirales | Siphoviridae | Unassigned |  | Overlap (VC_150/VC_174) |  |
| Lactobacillus~phage~CL2 | Caudovirales | Siphoviridae | Unassigned |  | Overlap (VC_150/VC_174) |  |
| Lactobacillus~phage~LBR48 | Caudovirales | Myoviridae | Unassigned |  | Overlap (VC_133/VC_241) |  |
| Lactobacillus~phage~LfeInf | Caudovirales | Herelleviridae | Unassigned |  | Outlier |  |
| Lactobacillus~phage~LfeSau | Caudovirales | Siphoviridae | Unassigned |  | Outlier |  |
| Lactobacillus~phage~Lv-1 | Caudovirales | Siphoviridae | Unassigned |  | Outlier |  |
| Lactobacillus~phage~PLE3 | Caudovirales | Siphoviridae | Unassigned |  | Overlap (VC_150/VC_174) |  |
| Lactobacillus~phage~Sha1 | Caudovirales | Siphoviridae | Unassigned |  | Outlier |  |
| Lactobacillus~phage~iLp1308 | Caudovirales | Siphoviridae | Unassigned |  | Overlap (VC_150/VC_174) |  |
| Lactobacillus~phage~iLp84 | Caudovirales | Siphoviridae | Unassigned |  | Overlap (VC_150/VC_174) |  |
| Lactobacillus~phage~phiadh | Caudovirales | Siphoviridae | Unassigned |  | Outlier |  |
| Lactobacillus~phage~phig1e | Caudovirales | Siphoviridae | Unassigned |  | Outlier |  |
| Lactobacillus~prophage~Lj928 | Caudovirales | Siphoviridae | Unassigned |  | Outlier |  |
| Lactobacillus~prophage~Lj965 | Caudovirales | Siphoviridae | Unassigned |  | Outlier |  |
| Lactobacillus~virus~LP65 | Caudovirales | Myoviridae | Unassigned |  | Outlier |  |
| Lactobacillus~virus~Lb338-1 | Caudovirales | Myoviridae | Unassigned |  | Outlier |  |
| Lactoccocus~phage~WP-2 | Caudovirales | Podoviridae | Unassigned |  | Outlier |  |
| Lactococcus~phage~1706 | Caudovirales | Siphoviridae | Unassigned |  | Outlier |  |
| Lactococcus~phage~28201 | Caudovirales | Siphoviridae | Unassigned |  | Overlap (VC_159/VC_297) |  |
| Lactococcus~phage~50101 | Caudovirales | Siphoviridae | Unassigned |  | Overlap (VC_159/VC_257) |  |
| Lactococcus~phage~63301 | Caudovirales | Siphoviridae | Unassigned |  | Overlap (VC_159/VC_257) |  |
| Lactococcus~phage~98201 | Caudovirales | Siphoviridae | Unassigned |  | Overlap (VC_159/VC_297) |  |
| Lactococcus~phage~GE1 | Caudovirales | Siphoviridae | Unassigned |  | Outlier |  |
| Lactococcus~phage~P087 | Caudovirales | Siphoviridae | Unassigned |  | Singleton |  |
| Lactococcus~phage~PLgT-1 | Caudovirales | Siphoviridae | Unassigned |  | Outlier |  |
| Lactococcus~phage~Q54 | Caudovirales | Siphoviridae | Unassigned |  | Outlier |  |
| Lactococcus~phage~TP901-1 | Caudovirales | Siphoviridae | Unassigned |  | Overlap (VC_159/VC_175) |  |
| Lactococcus~phage~Tuc2009 | Caudovirales | Siphoviridae | Unassigned |  | Overlap (VC_159/VC_175) |  |
| Lactococcus~phage~asccphi28 | Caudovirales | Podoviridae | Unassigned |  | Outlier |  |
| Lactococcus~phage~bIL311 | Caudovirales | Siphoviridae | Unassigned |  | Singleton |  |
| Lactococcus~phage~phiLC3 | Caudovirales | Siphoviridae | Unassigned |  | Overlap (VC_159/VC_257) |  |
| Lactococcus~phage~r1t | Caudovirales | Siphoviridae | Unassigned |  | Overlap (VC_159/VC_257) |  |
| Lactococcus~phage~ul36 | Caudovirales | Siphoviridae | Unassigned |  | Overlap (VC_159/VC_175) |  |
| Lactococcus~virus~KSY1 | Caudovirales | Podoviridae | Unassigned |  | Singleton |  |
| Lepus~americanus~faeces~associated~microvirus~SHP1~6472 | Unassigned | Microviridae | Unassigned |  | Overlap (VC_75/VC_78) |  |
| Listeria~phage~A006 | Caudovirales | Siphoviridae | Unassigned |  | Outlier |  |
| Listeria~phage~B054 | Caudovirales | Siphoviridae | Unassigned |  | Overlap (VC_133/VC_241) |  |
| Listeria~phage~WIL-1 | Caudovirales | Herelleviridae | Pecentumvirus |  | Outlier |  |
| Listonella~phage~phiHSIC | Caudovirales | Siphoviridae | Unassigned |  | Overlap (VC_61/VC_181) |  |
| Loktanella~phage~pCB2051-A | Unassigned | Unassigned | Unassigned |  | Outlier |  |
| Mannheimia~phage~vB_MhM_3927AP2 | Caudovirales | Myoviridae | Unassigned |  | Outlier |  |
| Marinomonas~phage~P12026 | Caudovirales | Siphoviridae | Unassigned |  | Overlap (VC_45/VC_180) |  |
| Mesorhizobium~phage~vB_MloP_Lo5R7ANS | Caudovirales | Podoviridae | Unassigned |  | Outlier |  |
| Metallosphaera~turreted~icosahedral~virus | Unassigned | Unassigned | Unassigned |  | Singleton |  |
| Microbacterium~phage~Dismas | Caudovirales | Siphoviridae | Dismasvirus |  | Outlier |  |
| Microbacterium~phage~Min1 | Caudovirales | Siphoviridae | Minunavirus |  | Outlier |  |
| Microviridae~Bog1249_12 | Unassigned | Microviridae | Unassigned |  | Overlap (VC_75/VC_78) |  |
| Microviridae~Fen4707_41 | Unassigned | Microviridae | Unassigned |  | Overlap (VC_77/VC_78) |  |
| Microviridae~Fen7918_21 | Unassigned | Microviridae | Unassigned |  | Overlap (VC_77/VC_78) |  |
| Microviridae~Fen7940_21 | Unassigned | Microviridae | Unassigned |  | Outlier |  |
| Microviridae~IME-16 | Unassigned | Microviridae | Unassigned |  | Singleton |  |
| Microviridae~phi-CA82 | Unassigned | Microviridae | Unassigned |  | Outlier |  |
| Morganella~phage~vB_MmoM_MP1 | Caudovirales | Myoviridae | Unassigned |  | Outlier |  |
| Mycobacterium~phage~32HC | Caudovirales | Siphoviridae | Trigintaduovirus |  | Outlier |  |
| Mycobacterium~phage~39HC | Caudovirales | Siphoviridae | Unassigned |  | Overlap (VC_57/VC_59) |  |
| Mycobacterium~phage~Adawi | Caudovirales | Siphoviridae | Coopervirus |  | Overlap (VC_57/VC_59) |  |
| Mycobacterium~phage~Adler | Caudovirales | Siphoviridae | Unassigned |  | Singleton |  |
| Mycobacterium~phage~Akoma | Caudovirales | Siphoviridae | Pipefishvirus |  | Overlap (VC_55/VC_57/VC_58/VC_59) |  |
| Mycobacterium~phage~Athena | Caudovirales | Siphoviridae | Pipefishvirus |  | Overlap (VC_55/VC_57/VC_58/VC_59) |  |
| Mycobacterium~phage~Baee | Caudovirales | Siphoviridae | Acadianvirus |  | Overlap (VC_57/VC_59) |  |
| Mycobacterium~phage~Bane1 | Caudovirales | Siphoviridae | Coopervirus |  | Overlap (VC_57/VC_59) |  |
| Mycobacterium~phage~Bernardo | Caudovirales | Siphoviridae | Pipefishvirus |  | Overlap (VC_55/VC_57/VC_58/VC_59) |  |
| Mycobacterium~phage~Bipper | Caudovirales | Siphoviridae | Unassigned |  | Outlier |  |
| Mycobacterium~phage~BrownCNA | Caudovirales | Siphoviridae | Coopervirus |  | Overlap (VC_57/VC_59) |  |
| Mycobacterium~phage~Chadwick | Caudovirales | Siphoviridae | Fromanvirus |  | Overlap (VC_285/VC_286) |  |
| Mycobacterium~phage~ChrisnMich | Caudovirales | Siphoviridae | Coopervirus |  | Overlap (VC_57/VC_59) |  |
| Mycobacterium~phage~Conspiracy | Caudovirales | Siphoviridae | Fromanvirus |  | Overlap (VC_285/VC_286) |  |
| Mycobacterium~phage~Cooper | Caudovirales | Siphoviridae | Coopervirus |  | Overlap (VC_57/VC_59) |  |
| Mycobacterium~phage~DS6A | Caudovirales | Siphoviridae | Unassigned |  | Outlier |  |
| Mycobacterium~phage~Dori | Caudovirales | Siphoviridae | Unassigned |  | Outlier |  |
| Mycobacterium~phage~Fredward | Caudovirales | Siphoviridae | Fromanvirus |  | Overlap (VC_285/VC_286) |  |
| Mycobacterium~phage~Gadjet | Caudovirales | Siphoviridae | Pipefishvirus |  | Overlap (VC_55/VC_57/VC_58/VC_59) |  |
| Mycobacterium~phage~Gaia | Caudovirales | Siphoviridae | Gaiavirus |  | Outlier |  |
| Mycobacterium~phage~Giles | Caudovirales | Siphoviridae | Gilesvirus |  | Outlier |  |
| Mycobacterium~phage~Godines | Caudovirales | Siphoviridae | Rosebushvirus |  | Overlap (VC_55/VC_57/VC_59) |  |
| Mycobacterium~phage~Hosp | Caudovirales | Siphoviridae | Unassigned |  | Overlap (VC_57/VC_59) |  |
| Mycobacterium~phage~JAMaL | Caudovirales | Siphoviridae | Coopervirus |  | Overlap (VC_57/VC_59) |  |
| Mycobacterium~phage~Jolie1 | Caudovirales | Siphoviridae | Unassigned |  | Overlap (VC_57/VC_59) |  |
| Mycobacterium~phage~Jovo | Caudovirales | Siphoviridae | Fromanvirus |  | Overlap (VC_285/VC_286) |  |
| Mycobacterium~phage~KayaCho | Caudovirales | Siphoviridae | Unassigned |  | Overlap (VC_57/VC_59) |  |
| Mycobacterium~phage~LittleCherry | Caudovirales | Siphoviridae | Fromanvirus |  | Overlap (VC_285/VC_286) |  |
| Mycobacterium~phage~Myrna | Caudovirales | Myoviridae | Unassigned |  | Outlier |  |
| Mycobacterium~phage~Nigel | Caudovirales | Siphoviridae | Coopervirus |  | Overlap (VC_57/VC_59) |  |
| Mycobacterium~phage~Phaedrus | Caudovirales | Siphoviridae | Pipefishvirus |  | Overlap (VC_55/VC_57/VC_58/VC_59) |  |
| Mycobacterium~phage~Phelemich | Caudovirales | Siphoviridae | Acadianvirus |  | Overlap (VC_57/VC_59) |  |
| Mycobacterium~phage~Phlyer | Caudovirales | Siphoviridae | Pipefishvirus |  | Overlap (VC_55/VC_57/VC_58/VC_59) |  |
| Mycobacterium~phage~Pipefish | Caudovirales | Siphoviridae | Pipefishvirus |  | Overlap (VC_55/VC_57/VC_58/VC_59) |  |
| Mycobacterium~phage~Qyrzula | Caudovirales | Siphoviridae | Rosebushvirus |  | Overlap (VC_55/VC_57/VC_59) |  |
| Mycobacterium~phage~Reprobate | Caudovirales | Siphoviridae | Acadianvirus |  | Overlap (VC_57/VC_59) |  |
| Mycobacterium~phage~Rosebush | Caudovirales | Siphoviridae | Rosebushvirus |  | Overlap (VC_55/VC_57/VC_59) |  |
| Mycobacterium~phage~Sbash | Caudovirales | Siphoviridae | Che9cvirus |  | Outlier |  |
| Mycobacterium~phage~Smeadley | Caudovirales | Siphoviridae | Fromanvirus |  | Overlap (VC_285/VC_286) |  |
| Mycobacterium~phage~Stinger | Caudovirales | Siphoviridae | Coopervirus |  | Overlap (VC_57/VC_59) |  |
| Mycobacterium~phage~Swirley | Caudovirales | Siphoviridae | Fromanvirus |  | Overlap (VC_285/VC_286) |  |
| Mycobacterium~phage~Theia | Caudovirales | Siphoviridae | Fromanvirus |  | Overlap (VC_285/VC_286) |  |
| Mycobacterium~phage~Tiger | Caudovirales | Siphoviridae | L5virus |  | Overlap (VC_285/VC_286) |  |
| Mycobacterium~phage~Tortellini | Caudovirales | Siphoviridae | Tortellinivirus |  | Outlier |  |
| Mycobacterium~phage~UnionJack | Caudovirales | Siphoviridae | Fromanvirus |  | Overlap (VC_285/VC_286) |  |
| Mycobacterium~phage~Vincenzo | Caudovirales | Siphoviridae | Coopervirus |  | Overlap (VC_57/VC_59) |  |
| Mycobacterium~phage~Wildcat | Caudovirales | Siphoviridae | Wildcatvirus |  | Outlier |  |
| Mycobacterium~phage~Zemanar | Caudovirales | Siphoviridae | Coopervirus |  | Overlap (VC_57/VC_59) |  |
| Mycobacterium~virus~Acadian | Caudovirales | Siphoviridae | Acadianvirus |  | Overlap (VC_57/VC_59) |  |
| Mycobacterium~virus~Babsiella | Caudovirales | Siphoviridae | Brujitavirus |  | Outlier |  |
| Mycobacterium~virus~Brujita | Caudovirales | Siphoviridae | Brujitavirus |  | Outlier |  |
| Mycobacterium~virus~Che9c | Caudovirales | Siphoviridae | Che9cvirus |  | Outlier |  |
| Mycoplasma~virus~P1 | Caudovirales | Podoviridae | Unassigned |  | Singleton |  |
| Myxococcus~phage~Mx8 | Caudovirales | Podoviridae | Myxoctovirus |  | Outlier |  |
| NIFA_virome_10658 | Unassigned | Unassigned | Unassigned |  | Singleton |  |
| NIFA_virome_1141 | Unassigned | Unassigned | Unassigned |  | Singleton |  |
| NIFA_virome_1434 | Unassigned | Unassigned | Unassigned |  | Singleton |  |
| NIFA_virome_15867 | Unassigned | Unassigned | Unassigned |  | Singleton |  |
| NIFA_virome_16493 | Unassigned | Unassigned | Unassigned |  | Singleton |  |
| NIFA_virome_16612 | Unassigned | Unassigned | Unassigned |  | Outlier |  |
| NIFA_virome_18499 | Unassigned | Unassigned | Unassigned |  | Singleton |  |
| NIFA_virome_19520 | Unassigned | Unassigned | Unassigned |  | Singleton |  |
| NIFA_virome_20851 | Unassigned | Unassigned | Unassigned |  | Singleton |  |
| NIFA_virome_21056 | Unassigned | Unassigned | Unassigned |  | Singleton |  |
| NIFA_virome_23088 | Unassigned | Unassigned | Unassigned |  | Singleton |  |
| NIFA_virome_23092 | Unassigned | Unassigned | Unassigned |  | Singleton |  |
| NIFA_virome_23606 | Unassigned | Unassigned | Unassigned |  | Singleton |  |
| NIFA_virome_25568 | Unassigned | Unassigned | Unassigned |  | Singleton |  |
| NIFA_virome_27311 | Unassigned | Unassigned | Unassigned |  | Outlier |  |
| NIFA_virome_27825 | Unassigned | Unassigned | Unassigned |  | Singleton |  |
| NIFA_virome_28509 | Unassigned | Unassigned | Unassigned |  | Singleton |  |
| NIFA_virome_29885 | Unassigned | Unassigned | Unassigned |  | Singleton |  |
| NIFA_virome_29952 | Unassigned | Unassigned | Unassigned |  | Singleton |  |
| NIFA_virome_30307 | Unassigned | Unassigned | Unassigned |  | Singleton |  |
| NIFA_virome_31247 | Unassigned | Unassigned | Unassigned |  | Singleton |  |
| NIFA_virome_31962 | Unassigned | Unassigned | Unassigned |  | Singleton |  |
| NIFA_virome_34410 | Unassigned | Unassigned | Unassigned |  | Singleton |  |
| NIFA_virome_36239 | Unassigned | Unassigned | Unassigned |  | Singleton |  |
| NIFA_virome_36582 | Unassigned | Unassigned | Unassigned |  | Outlier |  |
| NIFA_virome_43994 | Unassigned | Unassigned | Unassigned |  | Singleton |  |
| NIFA_virome_4537 | Unassigned | Unassigned | Unassigned |  | Singleton |  |
| NIFA_virome_45440 | Unassigned | Unassigned | Unassigned |  | Singleton |  |
| NIFA_virome_45576 | Unassigned | Unassigned | Unassigned |  | Singleton |  |
| NIFA_virome_46485 | Unassigned | Unassigned | Unassigned |  | Singleton |  |
| NIFA_virome_47644 | Unassigned | Unassigned | Unassigned |  | Singleton |  |
| NIFA_virome_48106 | Unassigned | Unassigned | Unassigned |  | Singleton |  |
| NIFA_virome_48647 | Unassigned | Unassigned | Unassigned |  | Singleton |  |
| NIFA_virome_52407 | Unassigned | Unassigned | Unassigned |  | Singleton |  |
| NIFA_virome_53111 | Unassigned | Unassigned | Unassigned |  | Singleton |  |
| NIFA_virome_53182 | Unassigned | Unassigned | Unassigned |  | Singleton |  |
| NIFA_virome_54186 | Unassigned | Unassigned | Unassigned |  | Singleton |  |
| NIFA_virome_55897 | Unassigned | Unassigned | Unassigned |  | Singleton |  |
| NIFA_virome_56672 | Unassigned | Unassigned | Unassigned |  | Singleton |  |
| NIFA_virome_57183 | Unassigned | Unassigned | Unassigned |  | Singleton |  |
| NIFA_virome_57641 | Unassigned | Unassigned | Unassigned |  | Singleton |  |
| NIFA_virome_58985 | Unassigned | Unassigned | Unassigned |  | Singleton |  |
| NIFA_virome_59209 | Unassigned | Unassigned | Unassigned |  | Singleton |  |
| NIFA_virome_60249 | Unassigned | Unassigned | Unassigned |  | Singleton |  |
| NIFA_virome_60628 | Unassigned | Unassigned | Unassigned |  | Outlier |  |
| NIFA_virome_60681 | Unassigned | Unassigned | Unassigned |  | Singleton |  |
| NIFA_virome_60699 | Unassigned | Unassigned | Unassigned |  | Singleton |  |
| NIFA_virome_60702 | Unassigned | Unassigned | Unassigned |  | Singleton |  |
| NIFA_virome_60737 | Unassigned | Unassigned | Unassigned |  | Singleton |  |
| NIFA_virome_60749 | Unassigned | Unassigned | Unassigned |  | Singleton |  |
| NIFA_virome_60751 | Unassigned | Unassigned | Unassigned |  | Outlier |  |
| NIFA_virome_60765 | Unassigned | Unassigned | Unassigned |  | Singleton |  |
| NIFA_virome_60794 | Unassigned | Unassigned | Unassigned |  | Outlier |  |
| NIFA_virome_60798 | Unassigned | Unassigned | Unassigned |  | Singleton |  |
| NIFA_virome_60804 | Unassigned | Unassigned | Unassigned |  | Outlier |  |
| NIFA_virome_60810 | Unassigned | Unassigned | Unassigned |  | Singleton |  |
| NIFA_virome_60875 | Unassigned | Unassigned | Unassigned |  | Singleton |  |
| NIFA_virome_60886 | Unassigned | Unassigned | Unassigned |  | Singleton |  |
| NIFA_virome_60918 | Unassigned | Unassigned | Unassigned |  | Singleton |  |
| NIFA_virome_60924 | Unassigned | Unassigned | Unassigned |  | Singleton |  |
| NIFA_virome_61061 | Unassigned | Unassigned | Unassigned |  | Outlier |  |
| NIFA_virome_61091 | Unassigned | Unassigned | Unassigned |  | Singleton |  |
| NIFA_virome_61094 | Unassigned | Unassigned | Unassigned |  | Singleton |  |
| NIFA_virome_61183 | Unassigned | Unassigned | Unassigned |  | Singleton |  |
| NIFA_virome_61229 | Unassigned | Unassigned | Unassigned |  | Singleton |  |
| NIFA_virome_61242 | Unassigned | Unassigned | Unassigned |  | Outlier |  |
| NIFA_virome_61294 | Unassigned | Unassigned | Unassigned |  | Outlier |  |
| NIFA_virome_61295 | Unassigned | Unassigned | Unassigned |  | Outlier |  |
| NIFA_virome_61316 | Unassigned | Unassigned | Unassigned |  | Singleton |  |
| NIFA_virome_61363 | Unassigned | Unassigned | Unassigned |  | Singleton |  |
| NIFA_virome_61390 | Unassigned | Unassigned | Unassigned |  | Singleton |  |
| NIFA_virome_61419 | Unassigned | Unassigned | Unassigned |  | Singleton |  |
| NIFA_virome_61423 | Unassigned | Unassigned | Unassigned |  | Singleton |  |
| NIFA_virome_61428 | Unassigned | Unassigned | Unassigned |  | Singleton |  |
| NIFA_virome_61456 | Unassigned | Unassigned | Unassigned |  | Singleton |  |
| NIFA_virome_61517 | Unassigned | Unassigned | Unassigned |  | Singleton |  |
| NIFA_virome_61585 | Unassigned | Unassigned | Unassigned |  | Singleton |  |
| NIFA_virome_61618 | Unassigned | Unassigned | Unassigned |  | Singleton |  |
| NIFA_virome_61652 | Unassigned | Unassigned | Unassigned |  | Singleton |  |
| NIFA_virome_61678 | Unassigned | Unassigned | Unassigned |  | Outlier |  |
| NIFA_virome_61703 | Unassigned | Unassigned | Unassigned |  | Singleton |  |
| NIFA_virome_61704 | Unassigned | Unassigned | Unassigned |  | Singleton |  |
| NIFA_virome_61730 | Unassigned | Unassigned | Unassigned |  | Outlier |  |
| NIFA_virome_61744 | Unassigned | Unassigned | Unassigned |  | Singleton |  |
| NIFA_virome_61761 | Unassigned | Unassigned | Unassigned |  | Singleton |  |
| NIFA_virome_61766 | Unassigned | Unassigned | Unassigned |  | Singleton |  |
| NIFA_virome_61818 | Unassigned | Unassigned | Unassigned |  | Singleton |  |
| NIFA_virome_61860 | Unassigned | Unassigned | Unassigned |  | Singleton |  |
| NIFA_virome_61876 | Unassigned | Unassigned | Unassigned |  | Singleton |  |
| NIFA_virome_61900 | Unassigned | Unassigned | Unassigned |  | Singleton |  |
| NIFA_virome_61905 | Unassigned | Unassigned | Unassigned |  | Singleton |  |
| NIFA_virome_61933 | Unassigned | Unassigned | Unassigned |  | Singleton |  |
| NIFA_virome_61971 | Unassigned | Unassigned | Unassigned |  | Singleton |  |
| NIFA_virome_61996 | Unassigned | Unassigned | Unassigned |  | Singleton |  |
| NIFA_virome_62013 | Unassigned | Unassigned | Unassigned |  | Singleton |  |
| NIFA_virome_62056 | Unassigned | Unassigned | Unassigned |  | Singleton |  |
| NIFA_virome_62075 | Unassigned | Unassigned | Unassigned |  | Singleton |  |
| NIFA_virome_62103 | Unassigned | Unassigned | Unassigned |  | Outlier |  |
| NIFA_virome_62109 | Unassigned | Unassigned | Unassigned |  | Singleton |  |
| NIFA_virome_62116 | Unassigned | Unassigned | Unassigned |  | Singleton |  |
| NIFA_virome_62163 | Unassigned | Unassigned | Unassigned |  | Singleton |  |
| NIFA_virome_62173 | Unassigned | Unassigned | Unassigned |  | Singleton |  |
| NIFA_virome_62184 | Unassigned | Unassigned | Unassigned |  | Singleton |  |
| NIFA_virome_62188 | Unassigned | Unassigned | Unassigned |  | Singleton |  |
| NIFA_virome_62249 | Unassigned | Unassigned | Unassigned |  | Singleton |  |
| NIFA_virome_62276 | Unassigned | Unassigned | Unassigned |  | Outlier |  |
| NIFA_virome_62297 | Unassigned | Unassigned | Unassigned |  | Outlier |  |
| NIFA_virome_62311 | Unassigned | Unassigned | Unassigned |  | Outlier |  |
| NIFA_virome_62356 | Unassigned | Unassigned | Unassigned |  | Singleton |  |
| NIFA_virome_62365 | Unassigned | Unassigned | Unassigned |  | Singleton |  |
| NIFA_virome_62374 | Unassigned | Unassigned | Unassigned |  | Singleton |  |
| NIFA_virome_62400 | Unassigned | Unassigned | Unassigned |  | Singleton |  |
| NIFA_virome_62437 | Unassigned | Unassigned | Unassigned |  | Singleton |  |
| NIFA_virome_62477 | Unassigned | Unassigned | Unassigned |  | Outlier |  |
| NIFA_virome_62508 | Unassigned | Unassigned | Unassigned |  | Outlier |  |
| NIFA_virome_62558 | Unassigned | Unassigned | Unassigned |  | Singleton |  |
| NIFA_virome_62575 | Unassigned | Unassigned | Unassigned |  | Singleton |  |
| NIFA_virome_62612 | Unassigned | Unassigned | Unassigned |  | Singleton |  |
| NIFA_virome_62642 | Unassigned | Unassigned | Unassigned |  | Outlier |  |
| NIFA_virome_62649 | Unassigned | Unassigned | Unassigned |  | Singleton |  |
| NIFA_virome_62659 | Unassigned | Unassigned | Unassigned |  | Singleton |  |
| NIFA_virome_62692 | Unassigned | Unassigned | Unassigned |  | Singleton |  |
| NIFA_virome_62709 | Unassigned | Unassigned | Unassigned |  | Singleton |  |
| NIFA_virome_62749 | Unassigned | Unassigned | Unassigned |  | Singleton |  |
| NIFA_virome_62756 | Unassigned | Unassigned | Unassigned |  | Singleton |  |
| NIFA_virome_62801 | Unassigned | Unassigned | Unassigned |  | Singleton |  |
| NIFA_virome_62866 | Unassigned | Unassigned | Unassigned |  | Singleton |  |
| NIFA_virome_62887 | Unassigned | Unassigned | Unassigned |  | Singleton |  |
| NIFA_virome_62945 | Unassigned | Unassigned | Unassigned |  | Singleton |  |
| NIFA_virome_62949 | Unassigned | Unassigned | Unassigned |  | Singleton |  |
| NIFA_virome_62962 | Unassigned | Unassigned | Unassigned |  | Singleton |  |
| NIFA_virome_62986 | Unassigned | Unassigned | Unassigned |  | Singleton |  |
| NIFA_virome_63000 | Unassigned | Unassigned | Unassigned |  | Singleton |  |
| NIFA_virome_63001 | Unassigned | Unassigned | Unassigned |  | Singleton |  |
| NIFA_virome_63067 | Unassigned | Unassigned | Unassigned |  | Outlier |  |
| NIFA_virome_63070 | Unassigned | Unassigned | Unassigned |  | Outlier |  |
| NIFA_virome_63080 | Unassigned | Unassigned | Unassigned |  | Singleton |  |
| NIFA_virome_63095 | Unassigned | Unassigned | Unassigned |  | Singleton |  |
| NIFA_virome_63102 | Unassigned | Unassigned | Unassigned |  | Singleton |  |
| NIFA_virome_63128 | Unassigned | Unassigned | Unassigned |  | Outlier |  |
| NIFA_virome_63164 | Unassigned | Unassigned | Unassigned |  | Singleton |  |
| NIFA_virome_63176 | Unassigned | Unassigned | Unassigned |  | Singleton |  |
| NIFA_virome_63188 | Unassigned | Unassigned | Unassigned |  | Singleton |  |
| NIFA_virome_63221 | Unassigned | Unassigned | Unassigned |  | Singleton |  |
| NIFA_virome_63235 | Unassigned | Unassigned | Unassigned |  | Singleton |  |
| NIFA_virome_63236 | Unassigned | Unassigned | Unassigned |  | Singleton |  |
| NIFA_virome_63344 | Unassigned | Unassigned | Unassigned |  | Singleton |  |
| NIFA_virome_63381 | Unassigned | Unassigned | Unassigned |  | Singleton |  |
| NIFA_virome_63382 | Unassigned | Unassigned | Unassigned |  | Singleton |  |
| NIFA_virome_63492 | Unassigned | Unassigned | Unassigned |  | Singleton |  |
| NIFA_virome_63864 | Unassigned | Unassigned | Unassigned |  | Singleton |  |
| NIFA_virome_63897 | Unassigned | Unassigned | Unassigned |  | Outlier |  |
| NIFA_virome_63924 | Unassigned | Unassigned | Unassigned |  | Singleton |  |
| NIFA_virome_63939 | Unassigned | Unassigned | Unassigned |  | Singleton |  |
| NIFA_virome_63948 | Unassigned | Unassigned | Unassigned |  | Outlier |  |
| NIFA_virome_63951 | Unassigned | Unassigned | Unassigned |  | Singleton |  |
| NIFA_virome_63967 | Unassigned | Unassigned | Unassigned |  | Singleton |  |
| NIFA_virome_63974 | Unassigned | Unassigned | Unassigned |  | Singleton |  |
| NIFA_virome_64092 | Unassigned | Unassigned | Unassigned |  | Outlier |  |
| NIFA_virome_64122 | Unassigned | Unassigned | Unassigned |  | Singleton |  |
| NIFA_virome_64134 | Unassigned | Unassigned | Unassigned |  | Singleton |  |
| NIFA_virome_64183 | Unassigned | Unassigned | Unassigned |  | Singleton |  |
| NIFA_virome_64186 | Unassigned | Unassigned | Unassigned |  | Singleton |  |
| NIFA_virome_64218 | Unassigned | Unassigned | Unassigned |  | Singleton |  |
| NIFA_virome_64230 | Unassigned | Unassigned | Unassigned |  | Singleton |  |
| NIFA_virome_6490 | Unassigned | Unassigned | Unassigned |  | Singleton |  |
| NIFA_virome_7102 | Unassigned | Unassigned | Unassigned |  | Singleton |  |
| NIFA_virome_7105 | Unassigned | Unassigned | Unassigned |  | Singleton |  |
| NIFA_virome_7290 | Unassigned | Unassigned | Unassigned |  | Singleton |  |
| NIFA_virome_7731 | Unassigned | Unassigned | Unassigned |  | Singleton |  |
| NIFA_virome_7733 | Unassigned | Unassigned | Unassigned |  | Singleton |  |
| NIFA_virome_8381 | Unassigned | Unassigned | Unassigned |  | Singleton |  |
| NIFA_virome_8979 | Unassigned | Unassigned | Unassigned |  | Singleton |  |
| NIFA_virome_9514 | Unassigned | Unassigned | Unassigned |  | Singleton |  |
| NIFA_virome_9523 | Unassigned | Unassigned | Unassigned |  | Outlier |  |
| Natrialba~phage~PhiCh1 | Caudovirales | Myoviridae | Myohalovirus |  | Outlier |  |
| Nitrincola~phage~1M3-16 | Caudovirales | Unassigned | Unassigned |  | Outlier |  |
| Nocardia~phage~NBR1 | Caudovirales | Siphoviridae | Unassigned |  | Outlier |  |
| Paenibacillus~phage~Harrison | Caudovirales | Siphoviridae | Harrisonvirus |  | Outlier |  |
| Paenibacillus~phage~PG1 | Caudovirales | Siphoviridae | Unassigned |  | Singleton |  |
| Paenibacillus~phage~Vegas | Caudovirales | Siphoviridae | Vegasvirus |  | Outlier |  |
| Pantoea~phage~LIMEzero | Caudovirales | Podoviridae | Phikmvvirus |  | Outlier |  |
| Parabacteroides~phage~YZ-2015a | Unassigned | Microviridae | Unassigned |  | Overlap (VC_75/VC_78) |  |
| Parabacteroides~phage~YZ-2015b | Unassigned | Microviridae | Unassigned |  | Overlap (VC_75/VC_78) |  |
| Paracoccus~phage~Shpa | Caudovirales | Siphoviridae | Vhulanivirus |  | Outlier |  |
| Pectobacterium~phage~ZF40 | Caudovirales | Myoviridae | Unassigned |  | Outlier |  |
| Pelagibacter~phage~HTVC008M | Caudovirales | Myoviridae | Unassigned |  | Outlier |  |
| Pelagibacter~phage~HTVC010P | Caudovirales | Podoviridae | Unassigned |  | Outlier |  |
| Pelagibacter~phage~HTVC011P | Caudovirales | Podoviridae | Unassigned |  | Outlier |  |
| Pelagibacter~phage~HTVC019P | Caudovirales | Podoviridae | Unassigned |  | Outlier |  |
| Phage~Gifsy-1 | Unassigned | Unassigned | Unassigned |  | Overlap (VC_64/VC_184/VC_185/VC_233) |  |
| Phage~Gifsy-2 | Unassigned | Unassigned | Unassigned |  | Overlap (VC_64/VC_184/VC_185/VC_233) |  |
| Phormidium~virus~WMP4 | Caudovirales | Podoviridae | Unassigned |  | Outlier |  |
| Planktothrix~phage~PaV-LD | Caudovirales | Podoviridae | Unassigned |  | Singleton |  |
| Podovirus~Lau218 | Caudovirales | Podoviridae | Unassigned |  | Outlier |  |
| Propionibacterium~phage~B5 | Unassigned | Inoviridae | Unassigned |  | Singleton |  |
| Propionibacterium~phage~PFR1 | Caudovirales | Siphoviridae | Pfr1virus |  | Overlap (VC_89/VC_342) |  |
| Propionibacterium~phage~PFR2 | Caudovirales | Siphoviridae | Pulverervirus |  | Overlap (VC_89/VC_342) |  |
| Proteus~phage~vB_PmiM_Pm5461 | Caudovirales | Myoviridae | Unassigned |  | Outlier |  |
| Pseudoalteromonas~phage~H101 | Caudovirales | Myoviridae | Unassigned |  | Outlier |  |
| Pseudoalteromonas~phage~Pq0 | Caudovirales | Siphoviridae | Unassigned |  | Overlap (VC_45/VC_180) |  |
| Pseudoalteromonas~phage~RIO-1 | Caudovirales | Podoviridae | Unassigned |  | Outlier |  |
| Pseudoalteromonas~phage~pYD6-A | Caudovirales | Podoviridae | Unassigned |  | Overlap (VC_12/VC_13) |  |
| Pseudomonas~phage~CHA_P1 | Caudovirales | Myoviridae | Kpp10virus |  | Overlap (VC_47/VC_48) |  |
| Pseudomonas~phage~KPP10 | Caudovirales | Myoviridae | Kpp10virus |  | Overlap (VC_47/VC_48) |  |
| Pseudomonas~phage~LKA1 | Caudovirales | Podoviridae | Phikmvvirus |  | Outlier |  |
| Pseudomonas~phage~PAK_P3 | Caudovirales | Myoviridae | Kpp10virus |  | Overlap (VC_47/VC_48) |  |
| Pseudomonas~phage~PAK_P5 | Caudovirales | Myoviridae | Kpp10virus |  | Overlap (VC_47/VC_48) |  |
| Pseudomonas~phage~PS-1 | Caudovirales | Siphoviridae | Unassigned |  | Outlier |  |
| Pseudomonas~phage~PaMx11 | Caudovirales | Siphoviridae | Ab18virus |  | Overlap (VC_3/VC_79) |  |
| Pseudomonas~phage~PaMx28 | Caudovirales | Siphoviridae | Pamx74virus |  | Overlap (VC_5/VC_79) |  |
| Pseudomonas~phage~PaMx74 | Caudovirales | Siphoviridae | Pamx74virus |  | Overlap (VC_5/VC_79) |  |
| Pseudomonas~phage~Pf3 | Unassigned | Inoviridae | Unassigned |  | Singleton |  |
| Pseudomonas~phage~SM1 | Caudovirales | Siphoviridae | Samunavirus |  | Outlier |  |
| Pseudomonas~phage~VCM | Caudovirales | Myoviridae | Otagovirus |  | Overlap (VC_47/VC_49) |  |
| Pseudomonas~phage~pf16 | Caudovirales | Myoviridae | Chakrabartyvirus |  | Outlier |  |
| Pseudomonas~phage~phi-2 | Caudovirales | Podoviridae | Phikmvvirus |  | Outlier |  |
| Pseudomonas~phage~phi8 | Unassigned | Cystoviridae | Cystovirus |  | Singleton |  |
| Pseudomonas~phage~phiPsa374 | Caudovirales | Myoviridae | Otagovirus |  | Overlap (VC_47/VC_49) |  |
| Pseudomonas~phage~tabernarius | Caudovirales | Podoviridae | Tabernariusvirus |  | Outlier |  |
| Pseudomonas~phage~vB_PaeM_G1 | Caudovirales | Myoviridae | Nankokuvirus |  | Overlap (VC_47/VC_48) |  |
| Pseudomonas~phage~vB_PaeM_PAO1_Ab03 | Caudovirales | Myoviridae | Kpp10virus |  | Overlap (VC_47/VC_48) |  |
| Pseudomonas~phage~vB_PaeM_PS24 | Caudovirales | Myoviridae | Nankokuvirus |  | Overlap (VC_47/VC_48) |  |
| Pseudomonas~phage~vB_PaeS_PAO1_Ab18 | Caudovirales | Siphoviridae | Ab18virus |  | Overlap (VC_3/VC_79) |  |
| Pseudomonas~virus~F116 | Caudovirales | Podoviridae | F116virus |  | Outlier |  |
| Pseudomonas~virus~Pf1 | Unassigned | Inoviridae | Unassigned |  | Singleton |  |
| Psychrobacter~phage~Psymv2 | Caudovirales | Siphoviridae | Unassigned |  | Outlier |  |
| Psychrobacter~phage~pOW20-A | Caudovirales | Myoviridae | Unassigned |  | Outlier |  |
| Pyrobaculum~filamentous~virus~1 | Unassigned | Tristromaviridae | Alphatristromavirus |  | Singleton |  |
| Pyrococcus~abyssi~virus~1 | Unassigned | Unassigned | Unassigned |  | Singleton |  |
| Ralstonia~phage~1~NP-2014 | Unassigned | Inoviridae | Unassigned |  | Outlier |  |
| Ralstonia~phage~RP12 | Caudovirales | Myoviridae | Ripduovirus |  | Outlier |  |
| Ralstonia~phage~RS138 | Caudovirales | Siphoviridae | Unassigned |  | Outlier |  |
| Ralstonia~phage~RSB3 | Caudovirales | Podoviridae | Phikmvvirus |  | Outlier |  |
| Ralstonia~phage~RSF1 | Caudovirales | Myoviridae | Rsl2virus |  | Outlier |  |
| Ralstonia~phage~RSL2 | Caudovirales | Myoviridae | Rsl2virus |  | Outlier |  |
| Ralstonia~phage~RSP15 | Caudovirales | Myoviridae | Unassigned |  | Outlier |  |
| Ralstonia~phage~RSS30 | Unassigned | Inoviridae | Unassigned |  | Outlier |  |
| Ralstonia~phage~phiRSL1 | Caudovirales | Myoviridae | Rslunavirus |  | Outlier |  |
| Rhizobium~phage~RR1-B | Caudovirales | Myoviridae | Unassigned |  | Outlier |  |
| Rhodobacter~phage~RcapMu | Caudovirales | Siphoviridae | Unassigned |  | Outlier |  |
| Rhodobacter~phage~RcapNL | Unassigned | Unassigned | Unassigned |  | Outlier |  |
| Rhodococcus~phage~E3 | Caudovirales | Myoviridae | Unassigned |  | Outlier |  |
| Rhodococcus~phage~REQ1 | Caudovirales | Siphoviridae | Unassigned |  | Singleton |  |
| Rhodococcus~phage~REQ2 | Caudovirales | Siphoviridae | Unassigned |  | Outlier |  |
| Rhodococcus~phage~ReqiDocB7 | Caudovirales | Siphoviridae | Unassigned |  | Outlier |  |
| Rhodococcus~phage~ReqiPepy6 | Caudovirales | Siphoviridae | Pepy6virus |  | Overlap (VC_95/VC_97) |  |
| Rhodococcus~phage~ReqiPine5 | Caudovirales | Siphoviridae | Unassigned |  | Outlier |  |
| Rhodococcus~phage~ReqiPoco6 | Caudovirales | Siphoviridae | Pepy6virus |  | Overlap (VC_95/VC_97) |  |
| Rhodothermus~phage~RM378 | Caudovirales | Myoviridae | Unassigned |  | Singleton |  |
| Rhodovulum~phage~vB_RhkS_P1 | Caudovirales | Siphoviridae | Unassigned |  | Outlier |  |
| Riemerella~phage~RAP44 | Caudovirales | Siphoviridae | Unassigned |  | Singleton |  |
| Ruegeria~phage~DSS3-P1 | Caudovirales | Siphoviridae | Unassigned |  | Outlier |  |
| Salicola~phage~CGphi29 | Unassigned | Unassigned | Unassigned |  | Outlier |  |
| Salisaeta~icosahedral~phage~1 | Unassigned | Unassigned | Unassigned |  | Singleton |  |
| Salmonella~phage~19 | Caudovirales | Myoviridae | Unassigned |  | Outlier |  |
| Salmonella~phage~21 | Caudovirales | Myoviridae | Unassigned |  | Outlier |  |
| Salmonella~phage~BP63 | Caudovirales | Unassigned | Unassigned |  | Outlier |  |
| Salmonella~phage~FSL~SP-058 | Caudovirales | Podoviridae | Sp58virus |  | Overlap (VC_8/VC_12) |  |
| Salmonella~phage~FSL~SP-076 | Caudovirales | Podoviridae | Sp58virus |  | Overlap (VC_8/VC_12) |  |
| Salmonella~phage~Fels-1 | Unassigned | Unassigned | Unassigned |  | Overlap (VC_64/VC_184/VC_185/VC_233) |  |
| Salmonella~phage~L13 | Caudovirales | Siphoviridae | Jerseyvirus |  | Outlier |  |
| Salmonella~phage~SEN34 | Caudovirales | Myoviridae | Brunovirus |  | Outlier |  |
| Salmonella~phage~SPN3UB | Caudovirales | Siphoviridae | Unassigned |  | Outlier |  |
| Salmonella~phage~vB_SosS_Oslo | Caudovirales | Siphoviridae | Unassigned |  | Outlier |  |
| Serratia~phage~Eta | Caudovirales | Siphoviridae | Unassigned |  | Outlier |  |
| Serratia~phage~PS2 | Caudovirales | Myoviridae | Unassigned |  | Outlier |  |
| Shewanella~sp.~phage~1/41 | Caudovirales | Myoviridae | Unassigned |  | Outlier |  |
| Shewanella~sp.~phage~3/49 | Caudovirales | Myoviridae | Unassigned |  | Outlier |  |
| Silicibacter~phage~DSS3phi2 | Caudovirales | Podoviridae | Baltimorevirus |  | Overlap (VC_8/VC_11) |  |
| Sinorhizobium~phage~PBC5 | Caudovirales | Podoviridae | Unassigned |  | Outlier |  |
| Sodalis~phage~phiSG1 | Caudovirales | Podoviridae | Unassigned |  | Outlier |  |
| Sphingobium~phage~Lacusarx | Caudovirales | Siphoviridae | Lacusarxvirus |  | Outlier |  |
| Sphingomonas~phage~PAU | Caudovirales | Myoviridae | Unassigned |  | Outlier |  |
| Spiroplasma~phage~SVTS2 | Unassigned | Inoviridae | Unassigned |  | Singleton |  |
| Spiroplasma~virus~SpV4 | Unassigned | Microviridae | Spiromicrovirus |  | Outlier |  |
| Staphylococcus~phage~2638A | Caudovirales | Siphoviridae | Biseptimavirus |  | Outlier |  |
| Staphylococcus~phage~CNPx | Caudovirales | Siphoviridae | Phietavirus |  | Overlap (VC_148/VC_152) |  |
| Staphylococcus~phage~IME-SA4 | Caudovirales | Siphoviridae | Biseptimavirus |  | Outlier |  |
| Staphylococcus~phage~PT1028 | Unassigned | Unassigned | Unassigned |  | Outlier |  |
| Staphylococcus~phage~SpaA1 | Caudovirales | Siphoviridae | Unassigned |  | Overlap (VC_130/VC_131) |  |
| Staphylococcus~phage~StB12 | Caudovirales | Siphoviridae | Phietavirus |  | Outlier |  |
| Staphylococcus~phage~StB20 | Caudovirales | Siphoviridae | Unassigned |  | Outlier |  |
| Staphylococcus~phage~StB20-like | Caudovirales | Siphoviridae | Unassigned |  | Outlier |  |
| Staphylococcus~phage~StB27 | Caudovirales | Siphoviridae | Phietavirus |  | Outlier |  |
| Staphylococcus~phage~StauST398-2 | Caudovirales | Siphoviridae | Triavirus |  | Overlap (VC_146/VC_148) |  |
| Staphylococcus~phage~TEM123 | Caudovirales | Siphoviridae | Phietavirus |  | Outlier |  |
| Staphylococcus~phage~YMC/09/04/R1988 | Caudovirales | Siphoviridae | Triavirus |  | Overlap (VC_146/VC_148) |  |
| Staphylococcus~phage~phi2958PVL | Caudovirales | Siphoviridae | Triavirus |  | Overlap (VC_146/VC_148) |  |
| Staphylococcus~phage~phiRS7 | Caudovirales | Siphoviridae | Biseptimavirus |  | Outlier |  |
| Staphylococcus~phage~tp310-2 | Caudovirales | Siphoviridae | Triavirus |  | Overlap (VC_146/VC_148) |  |
| Staphylococcus~phage~vB_SauS_phi2 | Caudovirales | Siphoviridae | Triavirus |  | Overlap (VC_146/VC_148) |  |
| Staphylococcus~virus~37 | Caudovirales | Siphoviridae | Phietavirus |  | Overlap (VC_148/VC_152) |  |
| Staphylococcus~virus~3a | Caudovirales | Siphoviridae | Triavirus |  | Overlap (VC_146/VC_148) |  |
| Staphylococcus~virus~42e | Caudovirales | Siphoviridae | Triavirus |  | Overlap (VC_146/VC_148) |  |
| Staphylococcus~virus~47 | Caudovirales | Siphoviridae | Triavirus |  | Overlap (VC_146/VC_148) |  |
| Staphylococcus~virus~77 | Caudovirales | Siphoviridae | Biseptimavirus |  | Overlap (VC_145/VC_148) |  |
| Staphylococcus~virus~CNPH82 | Caudovirales | Siphoviridae | Phietavirus |  | Overlap (VC_148/VC_152) |  |
| Staphylococcus~virus~EW | Caudovirales | Siphoviridae | Phietavirus |  | Overlap (VC_148/VC_152) |  |
| Staphylococcus~virus~IPLA35 | Caudovirales | Siphoviridae | Triavirus |  | Overlap (VC_146/VC_148) |  |
| Staphylococcus~virus~IPLA5 | Caudovirales | Siphoviridae | Phietavirus |  | Overlap (VC_148/VC_152) |  |
| Staphylococcus~virus~IPLA7 | Caudovirales | Siphoviridae | Phietavirus |  | Overlap (VC_148/VC_152) |  |
| Staphylococcus~virus~PH15 | Caudovirales | Siphoviridae | Phietavirus |  | Overlap (VC_148/VC_152) |  |
| Staphylococcus~virus~phi12 | Caudovirales | Siphoviridae | Triavirus |  | Overlap (VC_146/VC_148) |  |
| Staphylococcus~virus~phiSLT | Caudovirales | Siphoviridae | Triavirus |  | Overlap (VC_146/VC_148) |  |
| Streptococcus~phage~315.2 | Caudovirales | Siphoviridae | Unassigned |  | Outlier |  |
| Streptococcus~phage~315.4 | Caudovirales | Siphoviridae | Unassigned |  | Overlap (VC_124/VC_126) |  |
| Streptococcus~phage~315.5 | Caudovirales | Unassigned | Unassigned |  | Outlier |  |
| Streptococcus~phage~5093 | Caudovirales | Siphoviridae | Unassigned |  | Outlier |  |
| Streptococcus~phage~EJ-1 | Caudovirales | Myoviridae | Unassigned |  | Outlier |  |
| Streptococcus~phage~PH15 | Caudovirales | Siphoviridae | Unassigned |  | Outlier |  |
| Streptococcus~phage~SMP | Caudovirales | Siphoviridae | Unassigned |  | Outlier |  |
| Streptococcus~phage~T12 | Caudovirales | Siphoviridae | Unassigned |  | Outlier |  |
| Streptococcus~phage~YMC-2011 | Caudovirales | Siphoviridae | Moineauvirus |  | Overlap (VC_125/VC_298) |  |
| Streptococcus~virus~7201 | Caudovirales | Siphoviridae | Sfi21dt1virus |  | Overlap (VC_125/VC_298) |  |
| Streptococcus~virus~9871 | Caudovirales | Siphoviridae | Unassigned |  | Overlap (VC_125/VC_175) |  |
| Streptococcus~virus~9872 | Caudovirales | Siphoviridae | Unassigned |  | Overlap (VC_125/VC_175) |  |
| Streptococcus~virus~9874 | Caudovirales | Siphoviridae | Unassigned |  | Overlap (VC_125/VC_175) |  |
| Streptococcus~virus~C1 | Caudovirales | Podoviridae | P68virus |  | Outlier |  |
| Streptococcus~virus~DT1 | Caudovirales | Siphoviridae | Sfi21dt1virus |  | Overlap (VC_125/VC_298) |  |
| Streptococcus~virus~Sfi19 | Caudovirales | Siphoviridae | Sfi21dt1virus |  | Overlap (VC_125/VC_298) |  |
| Streptococcus~virus~Sfi21 | Caudovirales | Siphoviridae | Sfi21dt1virus |  | Overlap (VC_125/VC_298) |  |
| Streptococcus~virus~phiAbc2 | Caudovirales | Siphoviridae | Sfi21dt1virus |  | Overlap (VC_125/VC_298) |  |
| Streptomyces~phage~mu1/6 | Caudovirales | Siphoviridae | Unassigned |  | Outlier |  |
| Streptomyces~phage~phiSASD1 | Caudovirales | Siphoviridae | Lomovskayavirus |  | Outlier |  |
| Stx2-converting~phage~1717 | Caudovirales | Siphoviridae | Unassigned |  | Outlier |  |
| Sulfitobacter~phage~EE36phi1 | Caudovirales | Podoviridae | Baltimorevirus |  | Overlap (VC_8/VC_11) |  |
| Sulfitobacter~phage~phiCB2047-B | Caudovirales | Podoviridae | Baltimorevirus |  | Overlap (VC_8/VC_11) |  |
| Sulfolobales~Mexican~fusellovirus~1 | Unassigned | Fuselloviridae | Unassigned |  | Outlier |  |
| Sulfolobales~Mexican~rudivirus~1 | Ligamenvirales | Rudiviridae | Rudivirus |  | Outlier |  |
| Sulfolobus~polyhedral~virus~1 | Unassigned | Portogloboviridae | Alphaportoglobovirus |  | Singleton |  |
| Synechococcus~phage~S-CBS4 | Caudovirales | Siphoviridae | Unassigned |  | Outlier |  |
| Synechococcus~phage~S-CRM01 | Caudovirales | Myoviridae | Unassigned |  | Outlier |  |
| Temperate~phage~phiNIH1.1 | Caudovirales | Siphoviridae | Unassigned |  | Overlap (VC_124/VC_126) |  |
| Tetrasphaera~phage~TJE1 | Caudovirales | Myoviridae | Tijeunavirus |  | Singleton |  |
| Thalassomonas~phage~BA3 | Caudovirales | Podoviridae | Unassigned |  | Overlap (VC_163/VC_166) |  |
| Thermococcus~prieurii~virus~1 | Unassigned | Unassigned | Unassigned |  | Singleton |  |
| Thermoproteus~tenax~virus~1 | Unassigned | Tristromaviridae | Alphatristromavirus |  | Singleton |  |
| Thermus~phage~phi~OH2 | Caudovirales | Unassigned | Unassigned |  | Outlier |  |
| Tsukamurella~phage~TPA2 | Caudovirales | Siphoviridae | Unassigned |  | Outlier |  |
| Tsukamurella~phage~TPA4 | Caudovirales | Siphoviridae | Unassigned |  | Outlier |  |
| Verrucomicrobia~phage~P8625 | Caudovirales | Siphoviridae | Unassigned |  | Singleton |  |
| Vibrio~phage~JA-1 | Caudovirales | Podoviridae | Unassigned |  | Overlap (VC_10/VC_12) |  |
| Vibrio~phage~KSF1 | Unassigned | Inoviridae | Unassigned |  | Overlap (VC_368/VC_369) |  |
| Vibrio~phage~KVP40 | Caudovirales | Myoviridae | Schizot4virus |  | Overlap (VC_37/VC_40) |  |
| Vibrio~phage~PVA1 | Caudovirales | Podoviridae | Unassigned |  | Outlier |  |
| Vibrio~phage~VBM1 | Unassigned | Unassigned | Unassigned |  | Outlier |  |
| Vibrio~phage~VBP32 | Caudovirales | Podoviridae | Unassigned |  | Overlap (VC_12/VC_13) |  |
| Vibrio~phage~VBP47 | Caudovirales | Podoviridae | Unassigned |  | Overlap (VC_12/VC_13) |  |
| Vibrio~phage~VCY-phi | Unassigned | Inoviridae | Unassigned |  | Overlap (VC_368/VC_369) |  |
| Vibrio~phage~VH7D | Caudovirales | Myoviridae | Schizotequatrovirus |  | Overlap (VC_37/VC_40) |  |
| Vibrio~phage~ValKK3 | Caudovirales | Myoviridae | Schizot4virus |  | Overlap (VC_37/VC_40) |  |
| Vibrio~phage~VpKK5 | Caudovirales | Siphoviridae | Unassigned |  | Outlier |  |
| Vibrio~phage~VvAW1 | Caudovirales | Podoviridae | Unassigned |  | Overlap (VC_163/VC_166) |  |
| Vibrio~phage~douglas~12A4 | Unassigned | Unassigned | Unassigned |  | Outlier |  |
| Vibrio~phage~henriette~12B8 | Unassigned | Unassigned | Unassigned |  | Outlier |  |
| Vibrio~phage~martha~12B12 | Caudovirales | Myoviridae | Unassigned |  | Outlier |  |
| Vibrio~phage~nt-1 | Caudovirales | Myoviridae | Schizot4virus |  | Overlap (VC_37/VC_40) |  |
| Vibrio~phage~pYD38-B | Caudovirales | Siphoviridae | Unassigned |  | Overlap (VC_61/VC_181) |  |
| Vibrio~phage~phi~1 | Caudovirales | Podoviridae | Unassigned |  | Overlap (VC_10/VC_12) |  |
| Vibrio~virus~CTXphi | Unassigned | Inoviridae | Unassigned |  | Singleton |  |
| Vibrio~virus~Vf33 | Unassigned | Inoviridae | Unassigned |  | Outlier |  |
| Vibrio~virus~VpV262 | Caudovirales | Podoviridae | Unassigned |  | Outlier |  |
| Weissella~phage~WCP30 | Caudovirales | Siphoviridae | Unassigned |  | Outlier |  |
| Weissella~phage~phiYS61 | Caudovirales | Podoviridae | Unassigned |  | Outlier |  |
| Xanthomonas~phage~OP2 | Caudovirales | Myoviridae | Bcep78virus |  | Outlier |  |
| Xanthomonas~phage~Xp15 | Caudovirales | Siphoviridae | Unassigned |  | Outlier |  |
| Xanthomonas~virus~Cf1c | Unassigned | Inoviridae | Unassigned |  | Outlier |  |
| Xylella~phage~Xfas53 | Caudovirales | Podoviridae | Unassigned |  | Outlier |  |
| alaska_puertorico_778 | Unassigned | Unassigned | Unassigned |  | Singleton |  |
| alaska_puertorico_785 | Unassigned | Unassigned | Unassigned |  | Singleton |  |
| alaska_puertorico_938 | Unassigned | Unassigned | Unassigned |  | Singleton |  |
| alaska_puertorico_961 | Unassigned | Unassigned | Unassigned |  | Singleton |  |
| gary_all20_11916 | Unassigned | Unassigned | Unassigned |  | Outlier |  |
| gary_all20_16094 | Unassigned | Unassigned | Unassigned |  | Outlier |  |
| gary_all20_20258 | Unassigned | Unassigned | Unassigned |  | Singleton |  |
| uncultured~crAssphage | Unassigned | Unassigned | Unassigned |  | Singleton |  |

**Table S11**

| BankIt | vOTUs | Accession number |
| --- | --- | --- |
| BankIt2579032 | NIFA_virome_10658 | ON448394 |
| BankIt2579032 | NIFA_virome_10698 | ON448395 |
| BankIt2579032 | NIFA_virome_10923 | ON448396 |
| BankIt2579032 | NIFA_virome_1141 | ON448397 |
| BankIt2579032 | NIFA_virome_12105 | ON448398 |
| BankIt2579032 | NIFA_virome_1434 | ON448399 |
| BankIt2579032 | NIFA_virome_15867 | ON448400 |
| BankIt2579032 | NIFA_virome_16073 | ON448401 |
| BankIt2579032 | NIFA_virome_1612 | ON448402 |
| BankIt2579032 | NIFA_virome_16493 | ON448403 |
| BankIt2579032 | NIFA_virome_16612 | ON448404 |
| BankIt2579032 | NIFA_virome_17346 | ON448405 |
| BankIt2579032 | NIFA_virome_18499 | ON448406 |
| BankIt2579032 | NIFA_virome_19520 | ON448407 |
| BankIt2579032 | NIFA_virome_20851 | ON448408 |
| BankIt2579032 | NIFA_virome_21056 | ON448409 |
| BankIt2579032 | NIFA_virome_23088 | ON448410 |
| BankIt2579032 | NIFA_virome_23092 | ON448411 |
| BankIt2579032 | NIFA_virome_23606 | ON448412 |
| BankIt2579032 | NIFA_virome_25568 | ON448413 |
| BankIt2579032 | NIFA_virome_27311 | ON448414 |
| BankIt2579032 | NIFA_virome_27825 | ON448415 |
| BankIt2579032 | NIFA_virome_28509 | ON448416 |
| BankIt2579032 | NIFA_virome_29885 | ON448417 |
| BankIt2579032 | NIFA_virome_29952 | ON448418 |
| BankIt2579032 | NIFA_virome_30030 | ON448419 |
| BankIt2579032 | NIFA_virome_30307 | ON448420 |
| BankIt2579032 | NIFA_virome_31247 | ON448421 |
| BankIt2579032 | NIFA_virome_31962 | ON448422 |
| BankIt2579032 | NIFA_virome_34410 | ON448423 |
| BankIt2579032 | NIFA_virome_357 | ON448424 |
| BankIt2579032 | NIFA_virome_36239 | ON448425 |
| BankIt2579032 | NIFA_virome_36582 | ON448426 |
| BankIt2579032 | NIFA_virome_43994 | ON448427 |
| BankIt2579032 | NIFA_virome_4537 | ON448428 |
| BankIt2579032 | NIFA_virome_45440 | ON448429 |
| BankIt2579032 | NIFA_virome_45576 | ON448430 |
| BankIt2579032 | NIFA_virome_46485 | ON448431 |
| BankIt2579032 | NIFA_virome_47580 | ON448432 |
| BankIt2579032 | NIFA_virome_47644 | ON448433 |
| BankIt2579032 | NIFA_virome_48106 | ON448434 |
| BankIt2579032 | NIFA_virome_48647 | ON448435 |
| BankIt2579032 | NIFA_virome_49231 | ON448436 |
| BankIt2579032 | NIFA_virome_51733 | ON448437 |
| BankIt2579032 | NIFA_virome_51951 | ON448438 |
| BankIt2579032 | NIFA_virome_52407 | ON448439 |
| BankIt2579032 | NIFA_virome_52668 | ON448440 |
| BankIt2579032 | NIFA_virome_53111 | ON448441 |
| BankIt2579032 | NIFA_virome_53182 | ON448442 |
| BankIt2579032 | NIFA_virome_54186 | ON448443 |
| BankIt2579032 | NIFA_virome_55160 | ON448444 |
| BankIt2579032 | NIFA_virome_55897 | ON448445 |
| BankIt2579032 | NIFA_virome_56672 | ON448446 |
| BankIt2579032 | NIFA_virome_57183 | ON448447 |
| BankIt2579032 | NIFA_virome_57519 | ON448448 |
| BankIt2579032 | NIFA_virome_57641 | ON448449 |
| BankIt2579032 | NIFA_virome_58985 | ON448450 |
| BankIt2579032 | NIFA_virome_59209 | ON448451 |
| BankIt2579032 | NIFA_virome_60249 | ON448452 |
| BankIt2579032 | NIFA_virome_60613 | ON448453 |
| BankIt2579032 | NIFA_virome_60628 | ON448454 |
| BankIt2579032 | NIFA_virome_60681 | ON448455 |
| BankIt2579032 | NIFA_virome_60699 | ON448456 |
| BankIt2579032 | NIFA_virome_60702 | ON448457 |
| BankIt2579032 | NIFA_virome_60737 | ON448458 |
| BankIt2579032 | NIFA_virome_60749 | ON448459 |
| BankIt2579032 | NIFA_virome_60751 | ON448460 |
| BankIt2579032 | NIFA_virome_60765 | ON448461 |
| BankIt2579032 | NIFA_virome_60794 | ON448462 |
| BankIt2579032 | NIFA_virome_60798 | ON448463 |
| BankIt2579032 | NIFA_virome_60804 | ON448464 |
| BankIt2579032 | NIFA_virome_60810 | ON448465 |
| BankIt2579032 | NIFA_virome_60823 | ON448466 |
| BankIt2579032 | NIFA_virome_60831 | ON448467 |
| BankIt2579032 | NIFA_virome_60872 | ON448468 |
| BankIt2579032 | NIFA_virome_60875 | ON448469 |
| BankIt2579032 | NIFA_virome_60886 | ON448470 |
| BankIt2579032 | NIFA_virome_60918 | ON448471 |
| BankIt2579032 | NIFA_virome_60924 | ON448472 |
| BankIt2579032 | NIFA_virome_61061 | ON448473 |
| BankIt2579032 | NIFA_virome_61091 | ON448474 |
| BankIt2579032 | NIFA_virome_61094 | ON448475 |
| BankIt2579032 | NIFA_virome_61183 | ON448476 |
| BankIt2579032 | NIFA_virome_61188 | ON448477 |
| BankIt2579032 | NIFA_virome_61229 | ON448478 |
| BankIt2579032 | NIFA_virome_61242 | ON448479 |
| BankIt2579032 | NIFA_virome_61249 | ON448480 |
| BankIt2579032 | NIFA_virome_61294 | ON448481 |
| BankIt2579032 | NIFA_virome_61295 | ON448482 |
| BankIt2579032 | NIFA_virome_61313 | ON448483 |
| BankIt2579032 | NIFA_virome_61316 | ON448484 |
| BankIt2579032 | NIFA_virome_61321 | ON448485 |
| BankIt2579032 | NIFA_virome_61322 | ON448486 |
| BankIt2579032 | NIFA_virome_61363 | ON448487 |
| BankIt2579032 | NIFA_virome_61390 | ON448488 |
| BankIt2579032 | NIFA_virome_61419 | ON448489 |
| BankIt2579032 | NIFA_virome_61423 | ON448490 |
| BankIt2579032 | NIFA_virome_61428 | ON448491 |
| BankIt2579032 | NIFA_virome_61451 | ON448492 |
| BankIt2579032 | NIFA_virome_61456 | ON448493 |
| BankIt2579032 | NIFA_virome_61517 | ON448494 |
| BankIt2579032 | NIFA_virome_61533 | ON448495 |
| BankIt2579032 | NIFA_virome_61585 | ON448496 |
| BankIt2579032 | NIFA_virome_61618 | ON448497 |
| BankIt2579032 | NIFA_virome_61652 | ON448498 |
| BankIt2579032 | NIFA_virome_61678 | ON448499 |
| BankIt2579032 | NIFA_virome_61686 | ON448500 |
| BankIt2579032 | NIFA_virome_61703 | ON448501 |
| BankIt2579032 | NIFA_virome_61704 | ON448502 |
| BankIt2579032 | NIFA_virome_61730 | ON448503 |
| BankIt2579032 | NIFA_virome_61744 | ON448504 |
| BankIt2579032 | NIFA_virome_61761 | ON448505 |
| BankIt2579032 | NIFA_virome_61766 | ON448506 |
| BankIt2579032 | NIFA_virome_61802 | ON448507 |
| BankIt2579032 | NIFA_virome_61818 | ON448508 |
| BankIt2579032 | NIFA_virome_61844 | ON448509 |
| BankIt2579032 | NIFA_virome_61860 | ON448510 |
| BankIt2579032 | NIFA_virome_61872 | ON448511 |
| BankIt2579032 | NIFA_virome_61876 | ON448512 |
| BankIt2579032 | NIFA_virome_61900 | ON448513 |
| BankIt2579032 | NIFA_virome_61905 | ON448514 |
| BankIt2579032 | NIFA_virome_61933 | ON448515 |
| BankIt2579032 | NIFA_virome_61971 | ON448516 |
| BankIt2579032 | NIFA_virome_61978 | ON448517 |
| BankIt2579032 | NIFA_virome_61996 | ON448518 |
| BankIt2579032 | NIFA_virome_62013 | ON448519 |
| BankIt2579032 | NIFA_virome_62056 | ON448520 |
| BankIt2579032 | NIFA_virome_62075 | ON448521 |
| BankIt2579032 | NIFA_virome_62084 | ON448522 |
| BankIt2579032 | NIFA_virome_62103 | ON448523 |
| BankIt2579032 | NIFA_virome_62106 | ON448524 |
| BankIt2579032 | NIFA_virome_62109 | ON448525 |
| BankIt2579032 | NIFA_virome_62116 | ON448526 |
| BankIt2579032 | NIFA_virome_62163 | ON448527 |
| BankIt2579032 | NIFA_virome_62169 | ON448528 |
| BankIt2579032 | NIFA_virome_62173 | ON448529 |
| BankIt2579032 | NIFA_virome_62184 | ON448530 |
| BankIt2579032 | NIFA_virome_62188 | ON448531 |
| BankIt2579032 | NIFA_virome_62211 | ON448532 |
| BankIt2579032 | NIFA_virome_62249 | ON448533 |
| BankIt2579032 | NIFA_virome_62276 | ON448534 |
| BankIt2579032 | NIFA_virome_62297 | ON448535 |
| BankIt2579032 | NIFA_virome_62311 | ON448536 |
| BankIt2579032 | NIFA_virome_62356 | ON448537 |
| BankIt2579032 | NIFA_virome_62365 | ON448538 |
| BankIt2579032 | NIFA_virome_62374 | ON448539 |
| BankIt2579032 | NIFA_virome_62399 | ON448540 |
| BankIt2579032 | NIFA_virome_62400 | ON448541 |
| BankIt2579032 | NIFA_virome_62409 | ON448542 |
| BankIt2579032 | NIFA_virome_62437 | ON448543 |
| BankIt2579032 | NIFA_virome_62477 | ON448544 |
| BankIt2579032 | NIFA_virome_62479 | ON448545 |
| BankIt2579032 | NIFA_virome_62508 | ON448546 |
| BankIt2579032 | NIFA_virome_62558 | ON448547 |
| BankIt2579032 | NIFA_virome_62575 | ON448548 |
| BankIt2579032 | NIFA_virome_62612 | ON448549 |
| BankIt2579032 | NIFA_virome_62642 | ON448550 |
| BankIt2579032 | NIFA_virome_62649 | ON448551 |
| BankIt2579032 | NIFA_virome_62659 | ON448552 |
| BankIt2579032 | NIFA_virome_62692 | ON448553 |
| BankIt2579032 | NIFA_virome_62709 | ON448554 |
| BankIt2579032 | NIFA_virome_62749 | ON448555 |
| BankIt2579032 | NIFA_virome_62756 | ON448556 |
| BankIt2579032 | NIFA_virome_62801 | ON448557 |
| BankIt2579032 | NIFA_virome_62866 | ON448558 |
| BankIt2579032 | NIFA_virome_62887 | ON448559 |
| BankIt2579032 | NIFA_virome_62939 | ON448560 |
| BankIt2579032 | NIFA_virome_62945 | ON448561 |
| BankIt2579032 | NIFA_virome_62949 | ON448562 |
| BankIt2579032 | NIFA_virome_62962 | ON448563 |
| BankIt2579032 | NIFA_virome_62964 | ON448564 |
| BankIt2579032 | NIFA_virome_62967 | ON448565 |
| BankIt2579032 | NIFA_virome_62986 | ON448566 |
| BankIt2579032 | NIFA_virome_62993 | ON448567 |
| BankIt2579032 | NIFA_virome_63000 | ON448568 |
| BankIt2579032 | NIFA_virome_63001 | ON448569 |
| BankIt2579032 | NIFA_virome_63029 | ON448570 |
| BankIt2579032 | NIFA_virome_63067 | ON448571 |
| BankIt2579032 | NIFA_virome_63070 | ON448572 |
| BankIt2579032 | NIFA_virome_63080 | ON448573 |
| BankIt2579032 | NIFA_virome_63095 | ON448574 |
| BankIt2579032 | NIFA_virome_63102 | ON448575 |
| BankIt2579032 | NIFA_virome_63105 | ON448576 |
| BankIt2579032 | NIFA_virome_63121 | ON448577 |
| BankIt2579032 | NIFA_virome_63128 | ON448578 |
| BankIt2579032 | NIFA_virome_63164 | ON448579 |
| BankIt2579032 | NIFA_virome_63176 | ON448580 |
| BankIt2579032 | NIFA_virome_63181 | ON448581 |
| BankIt2579032 | NIFA_virome_63188 | ON448582 |
| BankIt2579032 | NIFA_virome_63221 | ON448583 |
| BankIt2579032 | NIFA_virome_63235 | ON448584 |
| BankIt2579032 | NIFA_virome_63236 | ON448585 |
| BankIt2579032 | NIFA_virome_63344 | ON448586 |
| BankIt2579032 | NIFA_virome_63381 | ON448587 |
| BankIt2579032 | NIFA_virome_63382 | ON448588 |
| BankIt2579032 | NIFA_virome_63492 | ON448589 |
| BankIt2579032 | NIFA_virome_63864 | ON448590 |
| BankIt2579032 | NIFA_virome_63897 | ON448591 |
| BankIt2579032 | NIFA_virome_63901 | ON448592 |
| BankIt2579032 | NIFA_virome_63920 | ON448593 |
| BankIt2579032 | NIFA_virome_63924 | ON448594 |
| BankIt2579032 | NIFA_virome_63939 | ON448595 |
| BankIt2579032 | NIFA_virome_63943 | ON448596 |
| BankIt2579032 | NIFA_virome_63944 | ON448597 |
| BankIt2579032 | NIFA_virome_63948 | ON448598 |
| BankIt2579032 | NIFA_virome_63951 | ON448599 |
| BankIt2579032 | NIFA_virome_63967 | ON448600 |
| BankIt2579032 | NIFA_virome_63974 | ON448601 |
| BankIt2579032 | NIFA_virome_64073 | ON448602 |
| BankIt2579032 | NIFA_virome_64080 | ON448603 |
| BankIt2579032 | NIFA_virome_64091 | ON448604 |
| BankIt2579032 | NIFA_virome_64092 | ON448605 |
| BankIt2579032 | NIFA_virome_64122 | ON448606 |
| BankIt2579032 | NIFA_virome_64134 | ON448607 |
| BankIt2579032 | NIFA_virome_64136 | ON448608 |
| BankIt2579032 | NIFA_virome_64179 | ON448609 |
| BankIt2579032 | NIFA_virome_64183 | ON448610 |
| BankIt2579032 | NIFA_virome_64186 | ON448611 |
| BankIt2579032 | NIFA_virome_64218 | ON448612 |
| BankIt2579032 | NIFA_virome_64230 | ON448613 |
| BankIt2579032 | NIFA_virome_6490 | ON448614 |
| BankIt2579032 | NIFA_virome_7102 | ON448615 |
| BankIt2579032 | NIFA_virome_7105 | ON448616 |
| BankIt2579032 | NIFA_virome_7290 | ON448617 |
| BankIt2579032 | NIFA_virome_7731 | ON448618 |
| BankIt2579032 | NIFA_virome_7733 | ON448619 |
| BankIt2579032 | NIFA_virome_8381 | ON448620 |
| BankIt2579032 | NIFA_virome_8907 | ON448621 |
| BankIt2579032 | NIFA_virome_8979 | ON448622 |
| BankIt2579032 | NIFA_virome_9264 | ON448623 |
| BankIt2579032 | NIFA_virome_9514 | ON448624 |
| BankIt2579032 | NIFA_virome_9523 | ON448625 |
