# Supplementary material for: Identification of Novel Viruses and Their Microbial Hosts from Soils with Long-Term Nitrogen Fertilization and Cover Cropping Management
Source: mSystems. 2022 Nov 29;7(6):e00571-22. doi: 10.1128/msystems.00571-22 (PMC9765229; doi:10.1128/msystems.00571-22)
Supplement: TABLE S4 [file msystems.00571-22-s0004.docx]

**Table S4**

| #contig | NCNTN0 | NCNTN60 | VNTN0 | VNTN60 | vOTU |
| --- | --- | --- | --- | --- | --- |
| gary_all20_3439 | 1 | 0 | 0 | 0 | vOTU_1 |
| gary_all20_7195 | 1 | 0 | 0 | 0 | vOTU_3 |
| gary_all20_10349 | 1 | 0 | 0 | 0 | vOTU_5 |
| gary_all20_11343 | 1 | 0 | 0 | 0 | vOTU_6 |
| virsorter_curated_4650 | 1 | 0 | 0 | 0 | vOTU_9 |
| NIFA_virome_16612 | 1 | 0 | 0 | 0 | vOTU_29 |
| NIFA_virome_61188 | 1 | 0 | 0 | 0 | vOTU_30 |
| NIFA_virome_61321 | 1 | 0 | 0 | 0 | vOTU_31 |
| NIFA_virome_61423 | 1 | 0 | 0 | 0 | vOTU_34 |
| NIFA_virome_63029 | 1 | 0 | 0 | 0 | vOTU_40 |
| NIFA_virome_61295 | 1 | 0 | 0 | 0 | vOTU_47 |
| NIFA_virome_63105 | 1 | 0 | 0 | 0 | vOTU_53 |
| NIFA_virome_60751 | 1 | 0 | 0 | 0 | vOTU_62 |
| NIFA_virome_61313 | 1 | 0 | 0 | 0 | vOTU_63 |
| NIFA_virome_57183 | 1 | 0 | 0 | 0 | vOTU_73 |
| NIFA_virome_60794 | 1 | 0 | 0 | 0 | vOTU_75 |
| NIFA_virome_60702 | 1 | 0 | 0 | 0 | vOTU_78 |
| NIFA_virome_61242 | 1 | 0 | 0 | 0 | vOTU_82 |
| NIFA_virome_61294 | 1 | 0 | 0 | 0 | vOTU_91 |
| NIFA_virome_10698 | 1 | 0 | 0 | 0 | vOTU_93 |
| NIFA_virome_60804 | 1 | 0 | 0 | 0 | vOTU_94 |
| NIFA_virome_60699 | 1 | 0 | 0 | 0 | vOTU_97 |
| NIFA_virome_63102 | 1 | 0 | 0 | 0 | vOTU_98 |
| NIFA_virome_57519 | 1 | 0 | 0 | 0 | vOTU_105 |
| NIFA_virome_16493 | 1 | 0 | 0 | 0 | vOTU_106 |
| NIFA_virome_61322 | 1 | 0 | 0 | 0 | vOTU_113 |
| NIFA_virome_61428 | 1 | 0 | 0 | 0 | vOTU_116 |
| NIFA_virome_61451 | 1 | 0 | 0 | 0 | vOTU_123 |
| NIFA_virome_16073 | 1 | 0 | 0 | 0 | vOTU_125 |
| NIFA_virome_59209 | 1 | 0 | 0 | 0 | vOTU_133 |
| NIFA_virome_10923 | 1 | 0 | 0 | 0 | vOTU_134 |
| NIFA_virome_60628 | 1 | 0 | 0 | 0 | vOTU_136 |
| NIFA_virome_63067 | 1 | 0 | 0 | 0 | vOTU_141 |
| NIFA_virome_63095 | 1 | 0 | 0 | 0 | vOTU_146 |
| NIFA_virome_15867 | 1 | 0 | 0 | 0 | vOTU_148 |
| NIFA_virome_61249 | 1 | 0 | 0 | 0 | vOTU_149 |
| NIFA_virome_60749 | 1 | 0 | 0 | 0 | vOTU_150 |
| NIFA_virome_63070 | 1 | 0 | 0 | 0 | vOTU_167 |
| NIFA_virome_60798 | 1 | 0 | 0 | 0 | vOTU_173 |
| NIFA_virome_60681 | 1 | 0 | 0 | 0 | vOTU_187 |
| NIFA_virome_63001 | 1 | 0 | 0 | 0 | vOTU_205 |
| NIFA_virome_61363 | 1 | 0 | 0 | 0 | vOTU_211 |
| NIFA_virome_61183 | 1 | 0 | 0 | 0 | vOTU_216 |
| NIFA_virome_57641 | 1 | 0 | 0 | 0 | vOTU_223 |
| NIFA_virome_60765 | 1 | 0 | 0 | 0 | vOTU_225 |
| NIFA_virome_60737 | 1 | 0 | 0 | 0 | vOTU_226 |
| NIFA_virome_61390 | 1 | 0 | 0 | 0 | vOTU_242 |
| NIFA_virome_1612 | 1 | 0 | 0 | 0 | vOTU_254 |
| NIFA_virome_60810 | 1 | 0 | 0 | 0 | vOTU_256 |
| NIFA_virome_61316 | 1 | 0 | 0 | 0 | vOTU_258 |
| NIFA_virome_63920 | 0 | 1 | 0 | 0 | vOTU_46 |
| NIFA_virome_63967 | 0 | 1 | 0 | 0 | vOTU_74 |
| NIFA_virome_64091 | 0 | 1 | 0 | 0 | vOTU_145 |
| NIFA_virome_64179 | 0 | 1 | 0 | 0 | vOTU_103 |
| NIFA_virome_63901 | 0 | 1 | 0 | 0 | vOTU_39 |
| NIFA_virome_63128 | 0 | 1 | 0 | 0 | vOTU_43 |
| NIFA_virome_64080 | 0 | 1 | 0 | 0 | vOTU_45 |
| NIFA_virome_63164 | 0 | 1 | 0 | 0 | vOTU_50 |
| NIFA_virome_63121 | 0 | 1 | 0 | 0 | vOTU_67 |
| NIFA_virome_64186 | 0 | 1 | 0 | 0 | vOTU_83 |
| NIFA_virome_63939 | 0 | 1 | 0 | 0 | vOTU_89 |
| NIFA_virome_64092 | 0 | 1 | 0 | 0 | vOTU_104 |
| NIFA_virome_62709 | 0 | 1 | 0 | 0 | vOTU_118 |
| NIFA_virome_64183 | 0 | 1 | 0 | 0 | vOTU_143 |
| NIFA_virome_62642 | 0 | 1 | 0 | 0 | vOTU_144 |
| NIFA_virome_63188 | 0 | 1 | 0 | 0 | vOTU_158 |
| NIFA_virome_62575 | 0 | 1 | 0 | 0 | vOTU_165 |
| NIFA_virome_64122 | 0 | 1 | 0 | 0 | vOTU_168 |
| NIFA_virome_63236 | 0 | 1 | 0 | 0 | vOTU_178 |
| NIFA_virome_64134 | 0 | 1 | 0 | 0 | vOTU_183 |
| NIFA_virome_63176 | 0 | 1 | 0 | 0 | vOTU_192 |
| NIFA_virome_55897 | 0 | 1 | 0 | 0 | vOTU_198 |
| NIFA_virome_62659 | 0 | 1 | 0 | 0 | vOTU_202 |
| NIFA_virome_45440 | 0 | 1 | 0 | 0 | vOTU_217 |
| NIFA_virome_63974 | 0 | 1 | 0 | 0 | vOTU_227 |
| NIFA_virome_62692 | 0 | 1 | 0 | 0 | vOTU_228 |
| NIFA_virome_63864 | 0 | 1 | 0 | 0 | vOTU_240 |
| NIFA_virome_62558 | 0 | 1 | 0 | 0 | vOTU_248 |
| NIFA_virome_61533 | 0 | 0 | 1 | 0 | vOTU_52 |
| NIFA_virome_62749 | 0 | 0 | 1 | 0 | vOTU_214 |
| NIFA_virome_62967 | 0 | 0 | 1 | 0 | vOTU_58 |
| gary_all20_6694 | 0 | 0 | 1 | 0 | vOTU_2 |
| gary_all20_16094 | 0 | 0 | 1 | 0 | vOTU_14 |
| gary_all20_23251 | 0 | 0 | 1 | 0 | vOTU_27 |
| NIFA_virome_60872 | 0 | 0 | 1 | 0 | vOTU_32 |
| NIFA_virome_62993 | 0 | 0 | 1 | 0 | vOTU_37 |
| NIFA_virome_61585 | 0 | 0 | 1 | 0 | vOTU_42 |
| NIFA_virome_61730 | 0 | 0 | 1 | 0 | vOTU_48 |
| NIFA_virome_60875 | 0 | 0 | 1 | 0 | vOTU_59 |
| NIFA_virome_61678 | 0 | 0 | 1 | 0 | vOTU_60 |
| NIFA_virome_62964 | 0 | 0 | 1 | 0 | vOTU_69 |
| NIFA_virome_4537 | 0 | 0 | 1 | 0 | vOTU_70 |
| NIFA_virome_62801 | 0 | 0 | 1 | 0 | vOTU_84 |
| NIFA_virome_62756 | 0 | 0 | 1 | 0 | vOTU_88 |
| NIFA_virome_20851 | 0 | 0 | 1 | 0 | vOTU_90 |
| NIFA_virome_62949 | 0 | 0 | 1 | 0 | vOTU_109 |
| NIFA_virome_62887 | 0 | 0 | 1 | 0 | vOTU_122 |
| NIFA_virome_60918 | 0 | 0 | 1 | 0 | vOTU_124 |
| NIFA_virome_61456 | 0 | 0 | 1 | 0 | vOTU_152 |
| NIFA_virome_60886 | 0 | 0 | 1 | 0 | vOTU_157 |
| NIFA_virome_6490 | 0 | 0 | 1 | 0 | vOTU_174 |
| NIFA_virome_61652 | 0 | 0 | 1 | 0 | vOTU_180 |
| NIFA_virome_34410 | 0 | 0 | 1 | 0 | vOTU_208 |
| NIFA_virome_61766 | 0 | 0 | 1 | 0 | vOTU_212 |
| NIFA_virome_21056 | 0 | 0 | 1 | 0 | vOTU_218 |
| NIFA_virome_62962 | 0 | 0 | 1 | 0 | vOTU_219 |
| NIFA_virome_61704 | 0 | 0 | 1 | 0 | vOTU_233 |
| NIFA_virome_61761 | 0 | 0 | 1 | 0 | vOTU_249 |
| NIFA_virome_62184 | 0 | 0 | 0 | 1 | vOTU_87 |
| NIFA_virome_62188 | 0 | 0 | 0 | 1 | vOTU_80 |
| NIFA_virome_62276 | 0 | 0 | 0 | 1 | vOTU_49 |
| gary_all20_20798 | 0 | 0 | 0 | 1 | vOTU_17 |
| alaska_puertorico_961 | 0 | 0 | 0 | 1 | vOTU_28 |
| NIFA_virome_61978 | 0 | 0 | 0 | 1 | vOTU_35 |
| NIFA_virome_62409 | 0 | 0 | 0 | 1 | vOTU_36 |
| NIFA_virome_62106 | 0 | 0 | 0 | 1 | vOTU_38 |
| NIFA_virome_62399 | 0 | 0 | 0 | 1 | vOTU_44 |
| NIFA_virome_51733 | 0 | 0 | 0 | 1 | vOTU_51 |
| NIFA_virome_62311 | 0 | 0 | 0 | 1 | vOTU_54 |
| NIFA_virome_61802 | 0 | 0 | 0 | 1 | vOTU_55 |
| NIFA_virome_62297 | 0 | 0 | 0 | 1 | vOTU_57 |
| NIFA_virome_7102 | 0 | 0 | 0 | 1 | vOTU_61 |
| NIFA_virome_7290 | 0 | 0 | 0 | 1 | vOTU_64 |
| NIFA_virome_62477 | 0 | 0 | 0 | 1 | vOTU_65 |
| NIFA_virome_61876 | 0 | 0 | 0 | 1 | vOTU_66 |
| NIFA_virome_61061 | 0 | 0 | 0 | 1 | vOTU_68 |
| NIFA_virome_63382 | 0 | 0 | 0 | 1 | vOTU_76 |
| NIFA_virome_62374 | 0 | 0 | 0 | 1 | vOTU_79 |
| NIFA_virome_62084 | 0 | 0 | 0 | 1 | vOTU_92 |
| NIFA_virome_7731 | 0 | 0 | 0 | 1 | vOTU_99 |
| NIFA_virome_61872 | 0 | 0 | 0 | 1 | vOTU_100 |
| NIFA_virome_62249 | 0 | 0 | 0 | 1 | vOTU_102 |
| NIFA_virome_27311 | 0 | 0 | 0 | 1 | vOTU_107 |
| NIFA_virome_60924 | 0 | 0 | 0 | 1 | vOTU_114 |
| NIFA_virome_62163 | 0 | 0 | 0 | 1 | vOTU_121 |
| NIFA_virome_61933 | 0 | 0 | 0 | 1 | vOTU_135 |
| NIFA_virome_9514 | 0 | 0 | 0 | 1 | vOTU_140 |
| NIFA_virome_62056 | 0 | 0 | 0 | 1 | vOTU_156 |
| NIFA_virome_29885 | 0 | 0 | 0 | 1 | vOTU_164 |
| NIFA_virome_62437 | 0 | 0 | 0 | 1 | vOTU_169 |
| NIFA_virome_61860 | 0 | 0 | 0 | 1 | vOTU_170 |
| NIFA_virome_8381 | 0 | 0 | 0 | 1 | vOTU_171 |
| NIFA_virome_61905 | 0 | 0 | 0 | 1 | vOTU_172 |
| NIFA_virome_61844 | 0 | 0 | 0 | 1 | vOTU_175 |
| NIFA_virome_61900 | 0 | 0 | 0 | 1 | vOTU_176 |
| NIFA_virome_62075 | 0 | 0 | 0 | 1 | vOTU_177 |
| NIFA_virome_63381 | 0 | 0 | 0 | 1 | vOTU_182 |
| NIFA_virome_62400 | 0 | 0 | 0 | 1 | vOTU_185 |
| NIFA_virome_63492 | 0 | 0 | 0 | 1 | vOTU_186 |
| NIFA_virome_62013 | 0 | 0 | 0 | 1 | vOTU_188 |
| NIFA_virome_62173 | 0 | 0 | 0 | 1 | vOTU_190 |
| NIFA_virome_28509 | 0 | 0 | 0 | 1 | vOTU_194 |
| NIFA_virome_23092 | 0 | 0 | 0 | 1 | vOTU_195 |
| NIFA_virome_62103 | 0 | 0 | 0 | 1 | vOTU_207 |
| NIFA_virome_61091 | 0 | 0 | 0 | 1 | vOTU_209 |
| NIFA_virome_62479 | 0 | 0 | 0 | 1 | vOTU_229 |
| NIFA_virome_62356 | 0 | 0 | 0 | 1 | vOTU_230 |
| NIFA_virome_25568 | 0 | 0 | 0 | 1 | vOTU_238 |
| NIFA_virome_62211 | 0 | 0 | 0 | 1 | vOTU_250 |
| NIFA_virome_62169 | 0 | 0 | 0 | 1 | vOTU_253 |
| NIFA_virome_27825 | 0 | 0 | 0 | 1 | vOTU_257 |
