# Supplementary figures and images for: Identification of Novel Viruses and Their Microbial Hosts from Soils with Long-Term Nitrogen Fertilization and Cover Cropping Management
Source: mSystems. 2022 Nov 29;7(6):e00571-22. doi: 10.1128/msystems.00571-22 (PMC9765229; doi:10.1128/msystems.00571-22)

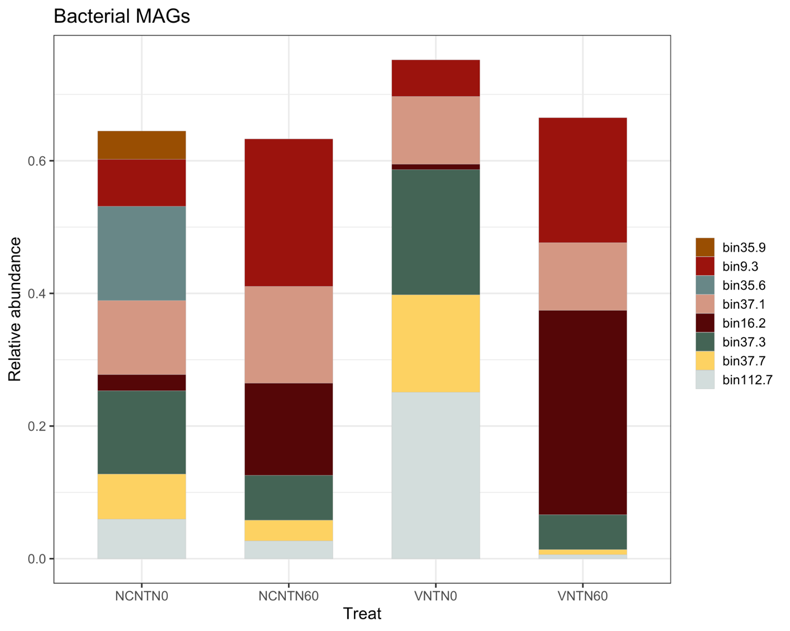


**Figure S1**

Supplement: FIG S1 [file msystems.00571-22-s0009.docx]

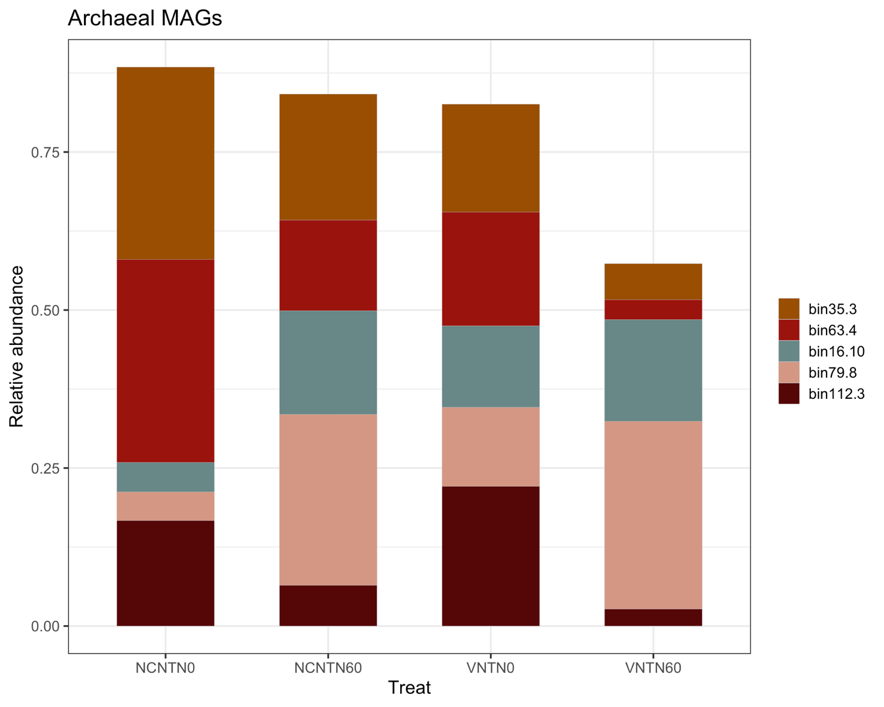


**Figure S2**

Supplement: FIG S2 [file msystems.00571-22-s0010.docx]
