# Supplementary material for: Identification of Novel Viruses and Their Microbial Hosts from Soils with Long-Term Nitrogen Fertilization and Cover Cropping Management
Source: mSystems. 2022 Nov 29;7(6):e00571-22. doi: 10.1128/msystems.00571-22 (PMC9765229; doi:10.1128/msystems.00571-22)
Supplement: TABLE S8 [file msystems.00571-22-s0008.docx]

**Table S8**

| BankIt | vOTUs | Accession number | type |
| --- | --- | --- | --- |
| BankIt2579032 | NIFA_virome_10658 | ON448394 | viral contig |
| BankIt2579032 | NIFA_virome_10698 | ON448395 | viral contig |
| BankIt2579032 | NIFA_virome_10923 | ON448396 | viral contig |
| BankIt2579032 | NIFA_virome_1141 | ON448397 | viral contig |
| BankIt2579032 | NIFA_virome_12105 | ON448398 | viral contig |
| BankIt2579032 | NIFA_virome_1434 | ON448399 | viral contig |
| BankIt2579032 | NIFA_virome_15867 | ON448400 | viral contig |
| BankIt2579032 | NIFA_virome_16073 | ON448401 | viral contig |
| BankIt2579032 | NIFA_virome_1612 | ON448402 | viral contig |
| BankIt2579032 | NIFA_virome_16493 | ON448403 | viral contig |
| BankIt2579032 | NIFA_virome_16612 | ON448404 | viral contig |
| BankIt2579032 | NIFA_virome_17346 | ON448405 | viral contig |
| BankIt2579032 | NIFA_virome_18499 | ON448406 | viral contig |
| BankIt2579032 | NIFA_virome_19520 | ON448407 | viral contig |
| BankIt2579032 | NIFA_virome_20851 | ON448408 | viral contig |
| BankIt2579032 | NIFA_virome_21056 | ON448409 | viral contig |
| BankIt2579032 | NIFA_virome_23088 | ON448410 | viral contig |
| BankIt2579032 | NIFA_virome_23092 | ON448411 | viral contig |
| BankIt2579032 | NIFA_virome_23606 | ON448412 | viral contig |
| BankIt2579032 | NIFA_virome_25568 | ON448413 | viral contig |
| BankIt2579032 | NIFA_virome_27311 | ON448414 | viral contig |
| BankIt2579032 | NIFA_virome_27825 | ON448415 | viral contig |
| BankIt2579032 | NIFA_virome_28509 | ON448416 | viral contig |
| BankIt2579032 | NIFA_virome_29885 | ON448417 | viral contig |
| BankIt2579032 | NIFA_virome_29952 | ON448418 | viral contig |
| BankIt2579032 | NIFA_virome_30030 | ON448419 | viral contig |
| BankIt2579032 | NIFA_virome_30307 | ON448420 | viral contig |
| BankIt2579032 | NIFA_virome_31247 | ON448421 | viral contig |
| BankIt2579032 | NIFA_virome_31962 | ON448422 | viral contig |
| BankIt2579032 | NIFA_virome_34410 | ON448423 | viral contig |
| BankIt2579032 | NIFA_virome_357 | ON448424 | viral contig |
| BankIt2579032 | NIFA_virome_36239 | ON448425 | viral contig |
| BankIt2579032 | NIFA_virome_36582 | ON448426 | viral contig |
| BankIt2579032 | NIFA_virome_43994 | ON448427 | viral contig |
| BankIt2579032 | NIFA_virome_4537 | ON448428 | viral contig |
| BankIt2579032 | NIFA_virome_45440 | ON448429 | viral contig |
| BankIt2579032 | NIFA_virome_45576 | ON448430 | viral contig |
| BankIt2579032 | NIFA_virome_46485 | ON448431 | viral contig |
| BankIt2579032 | NIFA_virome_47580 | ON448432 | viral contig |
| BankIt2579032 | NIFA_virome_47644 | ON448433 | viral contig |
| BankIt2579032 | NIFA_virome_48106 | ON448434 | viral contig |
| BankIt2579032 | NIFA_virome_48647 | ON448435 | viral contig |
| BankIt2579032 | NIFA_virome_49231 | ON448436 | viral contig |
| BankIt2579032 | NIFA_virome_51733 | ON448437 | viral contig |
| BankIt2579032 | NIFA_virome_51951 | ON448438 | viral contig |
| BankIt2579032 | NIFA_virome_52407 | ON448439 | viral contig |
| BankIt2579032 | NIFA_virome_52668 | ON448440 | viral contig |
| BankIt2579032 | NIFA_virome_53111 | ON448441 | viral contig |
| BankIt2579032 | NIFA_virome_53182 | ON448442 | viral contig |
| BankIt2579032 | NIFA_virome_54186 | ON448443 | viral contig |
| BankIt2579032 | NIFA_virome_55160 | ON448444 | viral contig |
| BankIt2579032 | NIFA_virome_55897 | ON448445 | viral contig |
| BankIt2579032 | NIFA_virome_56672 | ON448446 | viral contig |
| BankIt2579032 | NIFA_virome_57183 | ON448447 | viral contig |
| BankIt2579032 | NIFA_virome_57519 | ON448448 | viral contig |
| BankIt2579032 | NIFA_virome_57641 | ON448449 | viral contig |
| BankIt2579032 | NIFA_virome_58985 | ON448450 | viral contig |
| BankIt2579032 | NIFA_virome_59209 | ON448451 | viral contig |
| BankIt2579032 | NIFA_virome_60249 | ON448452 | viral contig |
| BankIt2579032 | NIFA_virome_60613 | ON448453 | viral contig |
| BankIt2579032 | NIFA_virome_60628 | ON448454 | viral contig |
| BankIt2579032 | NIFA_virome_60681 | ON448455 | viral contig |
| BankIt2579032 | NIFA_virome_60699 | ON448456 | viral contig |
| BankIt2579032 | NIFA_virome_60702 | ON448457 | viral contig |
| BankIt2579032 | NIFA_virome_60737 | ON448458 | viral contig |
| BankIt2579032 | NIFA_virome_60749 | ON448459 | viral contig |
| BankIt2579032 | NIFA_virome_60751 | ON448460 | viral contig |
| BankIt2579032 | NIFA_virome_60765 | ON448461 | viral contig |
| BankIt2579032 | NIFA_virome_60794 | ON448462 | viral contig |
| BankIt2579032 | NIFA_virome_60798 | ON448463 | viral contig |
| BankIt2579032 | NIFA_virome_60804 | ON448464 | viral contig |
| BankIt2579032 | NIFA_virome_60810 | ON448465 | viral contig |
| BankIt2579032 | NIFA_virome_60823 | ON448466 | viral contig |
| BankIt2579032 | NIFA_virome_60831 | ON448467 | viral contig |
| BankIt2579032 | NIFA_virome_60872 | ON448468 | viral contig |
| BankIt2579032 | NIFA_virome_60875 | ON448469 | viral contig |
| BankIt2579032 | NIFA_virome_60886 | ON448470 | viral contig |
| BankIt2579032 | NIFA_virome_60918 | ON448471 | viral contig |
| BankIt2579032 | NIFA_virome_60924 | ON448472 | viral contig |
| BankIt2579032 | NIFA_virome_61061 | ON448473 | viral contig |
| BankIt2579032 | NIFA_virome_61091 | ON448474 | viral contig |
| BankIt2579032 | NIFA_virome_61094 | ON448475 | viral contig |
| BankIt2579032 | NIFA_virome_61183 | ON448476 | viral contig |
| BankIt2579032 | NIFA_virome_61188 | ON448477 | viral contig |
| BankIt2579032 | NIFA_virome_61229 | ON448478 | viral contig |
| BankIt2579032 | NIFA_virome_61242 | ON448479 | viral contig |
| BankIt2579032 | NIFA_virome_61249 | ON448480 | viral contig |
| BankIt2579032 | NIFA_virome_61294 | ON448481 | viral contig |
| BankIt2579032 | NIFA_virome_61295 | ON448482 | viral contig |
| BankIt2579032 | NIFA_virome_61313 | ON448483 | viral contig |
| BankIt2579032 | NIFA_virome_61316 | ON448484 | viral contig |
| BankIt2579032 | NIFA_virome_61321 | ON448485 | viral contig |
| BankIt2579032 | NIFA_virome_61322 | ON448486 | viral contig |
| BankIt2579032 | NIFA_virome_61363 | ON448487 | viral contig |
| BankIt2579032 | NIFA_virome_61390 | ON448488 | viral contig |
| BankIt2579032 | NIFA_virome_61419 | ON448489 | viral contig |
| BankIt2579032 | NIFA_virome_61423 | ON448490 | viral contig |
| BankIt2579032 | NIFA_virome_61428 | ON448491 | viral contig |
| BankIt2579032 | NIFA_virome_61451 | ON448492 | viral contig |
| BankIt2579032 | NIFA_virome_61456 | ON448493 | viral contig |
| BankIt2579032 | NIFA_virome_61517 | ON448494 | viral contig |
| BankIt2579032 | NIFA_virome_61533 | ON448495 | viral contig |
| BankIt2579032 | NIFA_virome_61585 | ON448496 | viral contig |
| BankIt2579032 | NIFA_virome_61618 | ON448497 | viral contig |
| BankIt2579032 | NIFA_virome_61652 | ON448498 | viral contig |
| BankIt2579032 | NIFA_virome_61678 | ON448499 | viral contig |
| BankIt2579032 | NIFA_virome_61686 | ON448500 | viral contig |
| BankIt2579032 | NIFA_virome_61703 | ON448501 | viral contig |
| BankIt2579032 | NIFA_virome_61704 | ON448502 | viral contig |
| BankIt2579032 | NIFA_virome_61730 | ON448503 | viral contig |
| BankIt2579032 | NIFA_virome_61744 | ON448504 | viral contig |
| BankIt2579032 | NIFA_virome_61761 | ON448505 | viral contig |
| BankIt2579032 | NIFA_virome_61766 | ON448506 | viral contig |
| BankIt2579032 | NIFA_virome_61802 | ON448507 | viral contig |
| BankIt2579032 | NIFA_virome_61818 | ON448508 | viral contig |
| BankIt2579032 | NIFA_virome_61844 | ON448509 | viral contig |
| BankIt2579032 | NIFA_virome_61860 | ON448510 | viral contig |
| BankIt2579032 | NIFA_virome_61872 | ON448511 | viral contig |
| BankIt2579032 | NIFA_virome_61876 | ON448512 | viral contig |
| BankIt2579032 | NIFA_virome_61900 | ON448513 | viral contig |
| BankIt2579032 | NIFA_virome_61905 | ON448514 | viral contig |
| BankIt2579032 | NIFA_virome_61933 | ON448515 | viral contig |
| BankIt2579032 | NIFA_virome_61971 | ON448516 | viral contig |
| BankIt2579032 | NIFA_virome_61978 | ON448517 | viral contig |
| BankIt2579032 | NIFA_virome_61996 | ON448518 | viral contig |
| BankIt2579032 | NIFA_virome_62013 | ON448519 | viral contig |
| BankIt2579032 | NIFA_virome_62056 | ON448520 | viral contig |
| BankIt2579032 | NIFA_virome_62075 | ON448521 | viral contig |
| BankIt2579032 | NIFA_virome_62084 | ON448522 | viral contig |
| BankIt2579032 | NIFA_virome_62103 | ON448523 | viral contig |
| BankIt2579032 | NIFA_virome_62106 | ON448524 | viral contig |
| BankIt2579032 | NIFA_virome_62109 | ON448525 | viral contig |
| BankIt2579032 | NIFA_virome_62116 | ON448526 | viral contig |
| BankIt2579032 | NIFA_virome_62163 | ON448527 | viral contig |
| BankIt2579032 | NIFA_virome_62169 | ON448528 | viral contig |
| BankIt2579032 | NIFA_virome_62173 | ON448529 | viral contig |
| BankIt2579032 | NIFA_virome_62184 | ON448530 | viral contig |
| BankIt2579032 | NIFA_virome_62188 | ON448531 | viral contig |
| BankIt2579032 | NIFA_virome_62211 | ON448532 | viral contig |
| BankIt2579032 | NIFA_virome_62249 | ON448533 | viral contig |
| BankIt2579032 | NIFA_virome_62276 | ON448534 | viral contig |
| BankIt2579032 | NIFA_virome_62297 | ON448535 | viral contig |
| BankIt2579032 | NIFA_virome_62311 | ON448536 | viral contig |
| BankIt2579032 | NIFA_virome_62356 | ON448537 | viral contig |
| BankIt2579032 | NIFA_virome_62365 | ON448538 | viral contig |
| BankIt2579032 | NIFA_virome_62374 | ON448539 | viral contig |
| BankIt2579032 | NIFA_virome_62399 | ON448540 | viral contig |
| BankIt2579032 | NIFA_virome_62400 | ON448541 | viral contig |
| BankIt2579032 | NIFA_virome_62409 | ON448542 | viral contig |
| BankIt2579032 | NIFA_virome_62437 | ON448543 | viral contig |
| BankIt2579032 | NIFA_virome_62477 | ON448544 | viral contig |
| BankIt2579032 | NIFA_virome_62479 | ON448545 | viral contig |
| BankIt2579032 | NIFA_virome_62508 | ON448546 | viral contig |
| BankIt2579032 | NIFA_virome_62558 | ON448547 | viral contig |
| BankIt2579032 | NIFA_virome_62575 | ON448548 | viral contig |
| BankIt2579032 | NIFA_virome_62612 | ON448549 | viral contig |
| BankIt2579032 | NIFA_virome_62642 | ON448550 | viral contig |
| BankIt2579032 | NIFA_virome_62649 | ON448551 | viral contig |
| BankIt2579032 | NIFA_virome_62659 | ON448552 | viral contig |
| BankIt2579032 | NIFA_virome_62692 | ON448553 | viral contig |
| BankIt2579032 | NIFA_virome_62709 | ON448554 | viral contig |
| BankIt2579032 | NIFA_virome_62749 | ON448555 | viral contig |
| BankIt2579032 | NIFA_virome_62756 | ON448556 | viral contig |
| BankIt2579032 | NIFA_virome_62801 | ON448557 | viral contig |
| BankIt2579032 | NIFA_virome_62866 | ON448558 | viral contig |
| BankIt2579032 | NIFA_virome_62887 | ON448559 | viral contig |
| BankIt2579032 | NIFA_virome_62939 | ON448560 | viral contig |
| BankIt2579032 | NIFA_virome_62945 | ON448561 | viral contig |
| BankIt2579032 | NIFA_virome_62949 | ON448562 | viral contig |
| BankIt2579032 | NIFA_virome_62962 | ON448563 | viral contig |
| BankIt2579032 | NIFA_virome_62964 | ON448564 | viral contig |
| BankIt2579032 | NIFA_virome_62967 | ON448565 | viral contig |
| BankIt2579032 | NIFA_virome_62986 | ON448566 | viral contig |
| BankIt2579032 | NIFA_virome_62993 | ON448567 | viral contig |
| BankIt2579032 | NIFA_virome_63000 | ON448568 | viral contig |
| BankIt2579032 | NIFA_virome_63001 | ON448569 | viral contig |
| BankIt2579032 | NIFA_virome_63029 | ON448570 | viral contig |
| BankIt2579032 | NIFA_virome_63067 | ON448571 | viral contig |
| BankIt2579032 | NIFA_virome_63070 | ON448572 | viral contig |
| BankIt2579032 | NIFA_virome_63080 | ON448573 | viral contig |
| BankIt2579032 | NIFA_virome_63095 | ON448574 | viral contig |
| BankIt2579032 | NIFA_virome_63102 | ON448575 | viral contig |
| BankIt2579032 | NIFA_virome_63105 | ON448576 | viral contig |
| BankIt2579032 | NIFA_virome_63121 | ON448577 | viral contig |
| BankIt2579032 | NIFA_virome_63128 | ON448578 | viral contig |
| BankIt2579032 | NIFA_virome_63164 | ON448579 | viral contig |
| BankIt2579032 | NIFA_virome_63176 | ON448580 | viral contig |
| BankIt2579032 | NIFA_virome_63181 | ON448581 | viral contig |
| BankIt2579032 | NIFA_virome_63188 | ON448582 | viral contig |
| BankIt2579032 | NIFA_virome_63221 | ON448583 | viral contig |
| BankIt2579032 | NIFA_virome_63235 | ON448584 | viral contig |
| BankIt2579032 | NIFA_virome_63236 | ON448585 | viral contig |
| BankIt2579032 | NIFA_virome_63344 | ON448586 | viral contig |
| BankIt2579032 | NIFA_virome_63381 | ON448587 | viral contig |
| BankIt2579032 | NIFA_virome_63382 | ON448588 | viral contig |
| BankIt2579032 | NIFA_virome_63492 | ON448589 | viral contig |
| BankIt2579032 | NIFA_virome_63864 | ON448590 | viral contig |
| BankIt2579032 | NIFA_virome_63897 | ON448591 | viral contig |
| BankIt2579032 | NIFA_virome_63901 | ON448592 | viral contig |
| BankIt2579032 | NIFA_virome_63920 | ON448593 | viral contig |
| BankIt2579032 | NIFA_virome_63924 | ON448594 | viral contig |
| BankIt2579032 | NIFA_virome_63939 | ON448595 | viral contig |
| BankIt2579032 | NIFA_virome_63943 | ON448596 | viral contig |
| BankIt2579032 | NIFA_virome_63944 | ON448597 | viral contig |
| BankIt2579032 | NIFA_virome_63948 | ON448598 | viral contig |
| BankIt2579032 | NIFA_virome_63951 | ON448599 | viral contig |
| BankIt2579032 | NIFA_virome_63967 | ON448600 | viral contig |
| BankIt2579032 | NIFA_virome_63974 | ON448601 | viral contig |
| BankIt2579032 | NIFA_virome_64073 | ON448602 | viral contig |
| BankIt2579032 | NIFA_virome_64080 | ON448603 | viral contig |
| BankIt2579032 | NIFA_virome_64091 | ON448604 | viral contig |
| BankIt2579032 | NIFA_virome_64092 | ON448605 | viral contig |
| BankIt2579032 | NIFA_virome_64122 | ON448606 | viral contig |
| BankIt2579032 | NIFA_virome_64134 | ON448607 | viral contig |
| BankIt2579032 | NIFA_virome_64136 | ON448608 | viral contig |
| BankIt2579032 | NIFA_virome_64179 | ON448609 | viral contig |
| BankIt2579032 | NIFA_virome_64183 | ON448610 | viral contig |
| BankIt2579032 | NIFA_virome_64186 | ON448611 | viral contig |
| BankIt2579032 | NIFA_virome_64218 | ON448612 | viral contig |
| BankIt2579032 | NIFA_virome_64230 | ON448613 | viral contig |
| BankIt2579032 | NIFA_virome_6490 | ON448614 | viral contig |
| BankIt2579032 | NIFA_virome_7102 | ON448615 | viral contig |
| BankIt2579032 | NIFA_virome_7105 | ON448616 | viral contig |
| BankIt2579032 | NIFA_virome_7290 | ON448617 | viral contig |
| BankIt2579032 | NIFA_virome_7731 | ON448618 | viral contig |
| BankIt2579032 | NIFA_virome_7733 | ON448619 | viral contig |
| BankIt2579032 | NIFA_virome_8381 | ON448620 | viral contig |
| BankIt2579032 | NIFA_virome_8907 | ON448621 | viral contig |
| BankIt2579032 | NIFA_virome_8979 | ON448622 | viral contig |
| BankIt2579032 | NIFA_virome_9264 | ON448623 | viral contig |
| BankIt2579032 | NIFA_virome_9514 | ON448624 | viral contig |
| BankIt2579032 | NIFA_virome_9523 | ON448625 | viral contig |
|  | NCN0_1_bin6 | SAMN27157275 | draft genome |
|  | NCN0_1_bin9 | SAMN27157276 | draft genome |
|  | NCN0_1_bin3 | SAMN27157277 | draft genome |
|  | NCN0_2_bin4 | SAMN27157278 | draft genome |
|  | NCN0_4_bin3 | SAMN27157279 | draft genome |
|  | NCN60_2_bin3 | SAMN27157280 | draft genome |
|  | VN0_1_bin3 | SAMN27157281 | draft genome |
|  | VN0_1_bin7 | SAMN27157282 | draft genome |
|  | VN0_1_bin1 | SAMN27157283 | draft genome |
|  | VN0_2_bin1 | SAMN27157284 | draft genome |
|  | VN0_2_bin2 | SAMN27157285 | draft genome |
|  | VN0_4_bin3 | SAMN27157286 | draft genome |
|  | VN0_4_bin7 | SAMN27157287 | draft genome |
|  | VN60_1_bin3 | SAMN27157288 | draft genome |
|  | VN60_2_bin1 | SAMN27157289 | draft genome |
|  | VN60_2_bin2 | SAMN27157290 | draft genome |
|  | VN60_2_bin4 | SAMN27157291 | draft genome |
|  | VN60_2_bin5 | SAMN27157292 | draft genome |
|  | VN60_2_bin10 | SAMN27157293 | draft genome |
|  | VN60_4_bin8 | SAMN27157294 | draft genome |
